# Supplementary figures and images for: Ubiquitin ligase ITCH regulates life cycle of SARS-CoV-2 virus (part 1 of 4)
Source: eLife. 2026 May 29;14:RP105105. doi: 10.7554/eLife.105105 (PMC13221179; doi:10.7554/eLife.105105)

1G

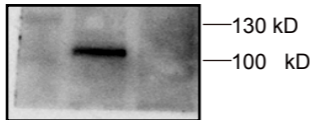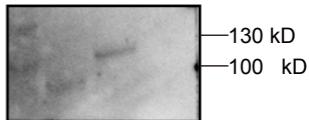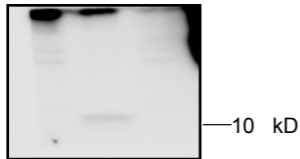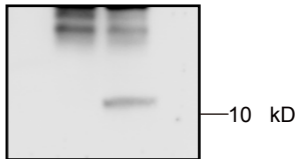

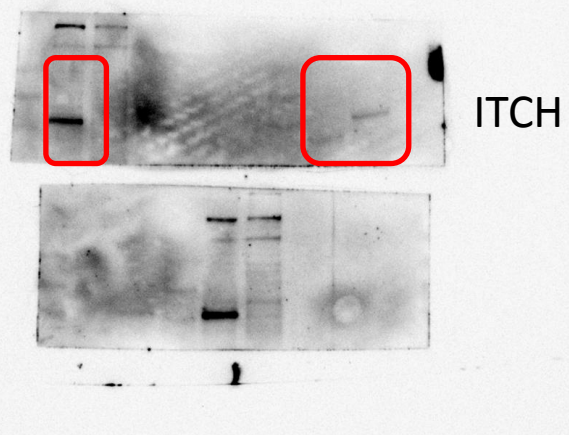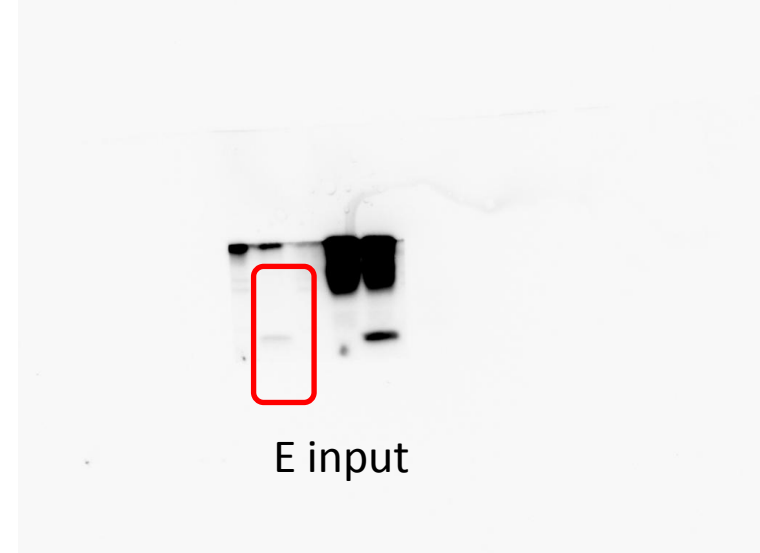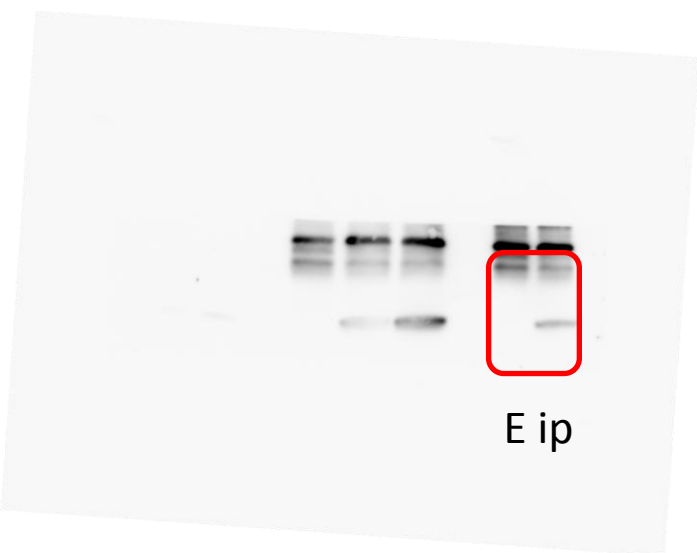

Supplement: Figure 1—source data 1. [file elife-105105-fig1-data1.zip › Figure 1G.pdf]

1H

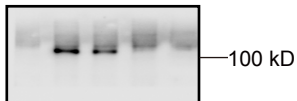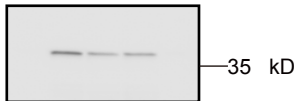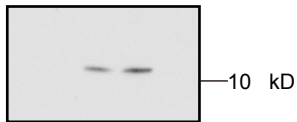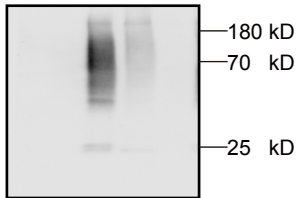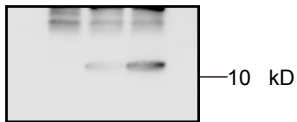

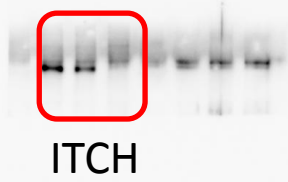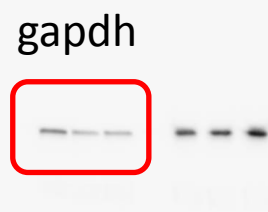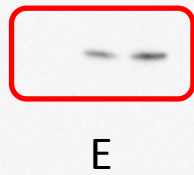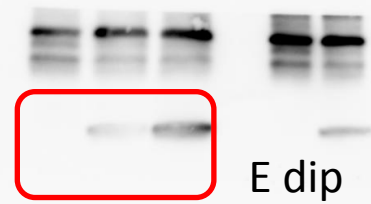

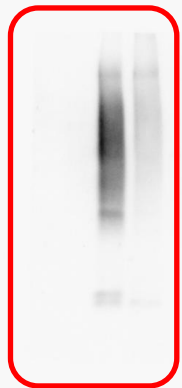

E ubi

Supplement: Figure 1—source data 1. [file elife-105105-fig1-data1.zip › Figure 1H.pdf]

1A

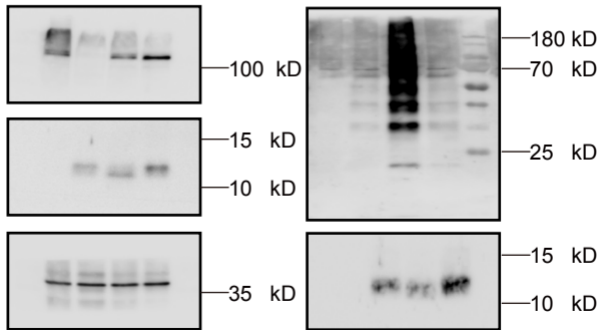

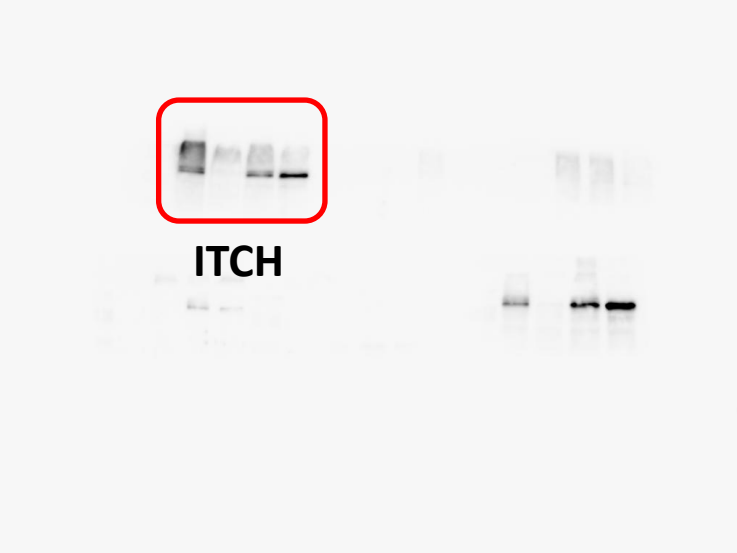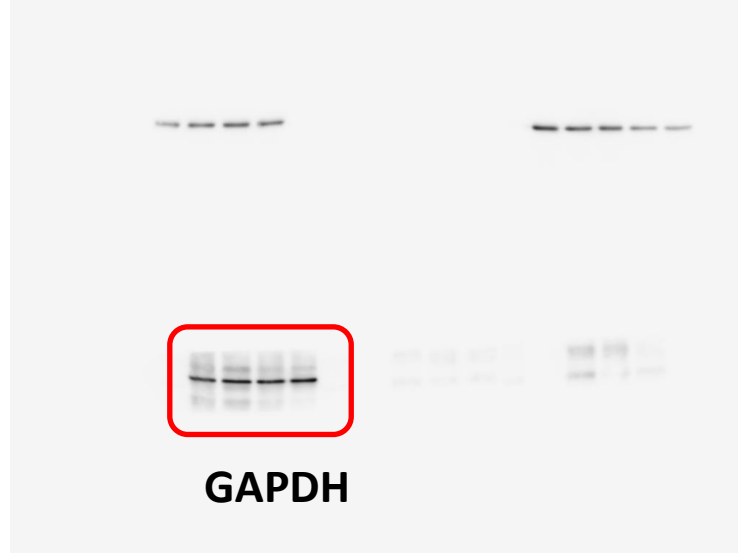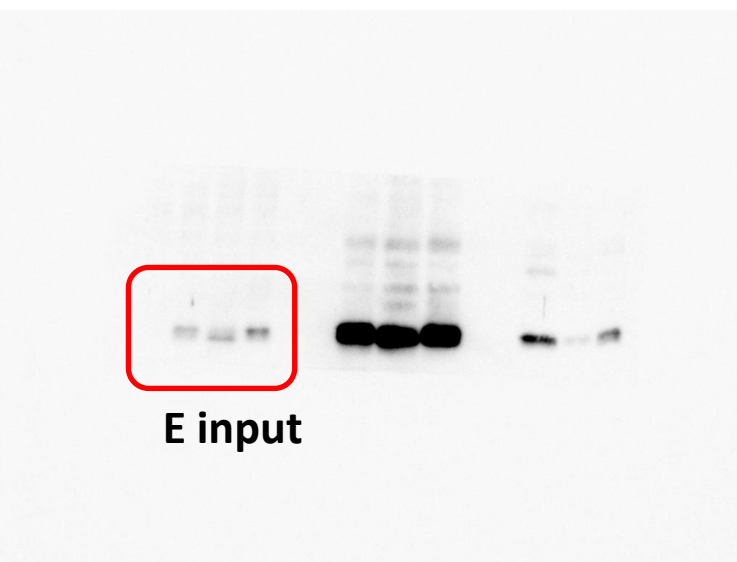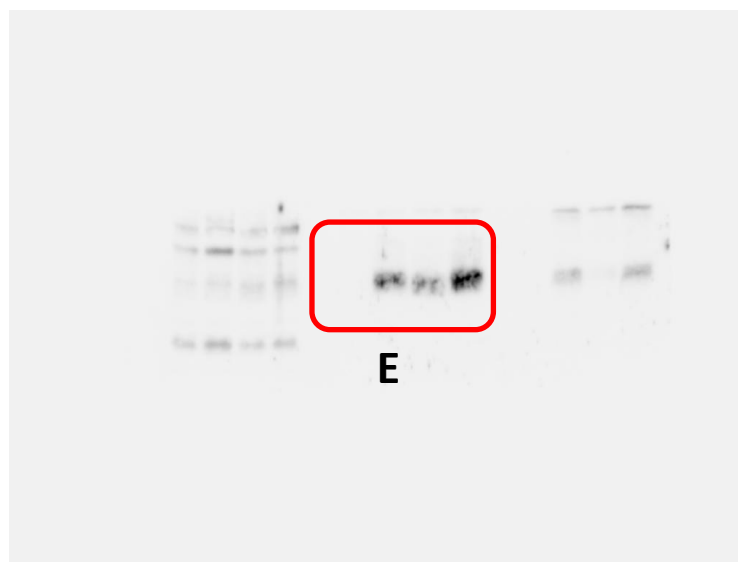

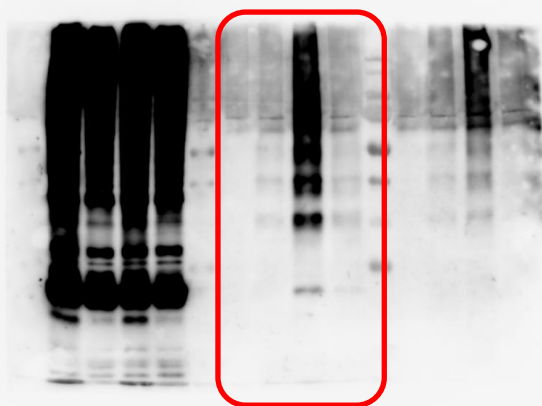

ubi

Supplement: Figure 1—source data 1. [file elife-105105-fig1-data1.zip › Figure 1A.pdf]

1B

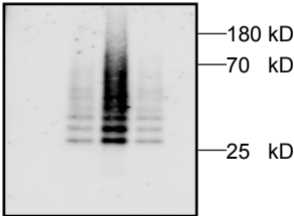

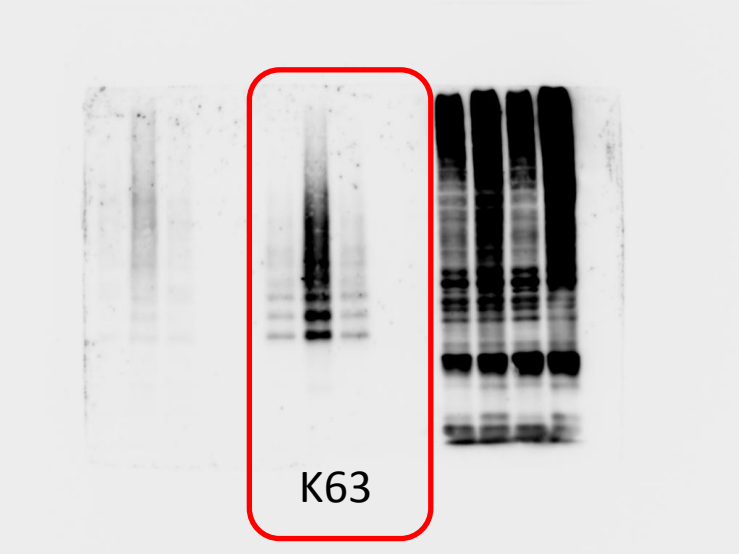

Supplement: Figure 1—source data 1. [file elife-105105-fig1-data1.zip › Figure 1B.pdf]

1C

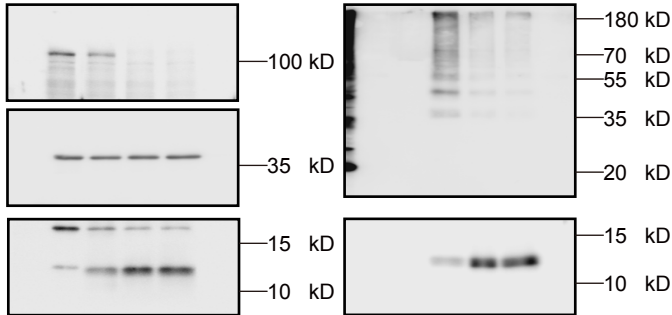

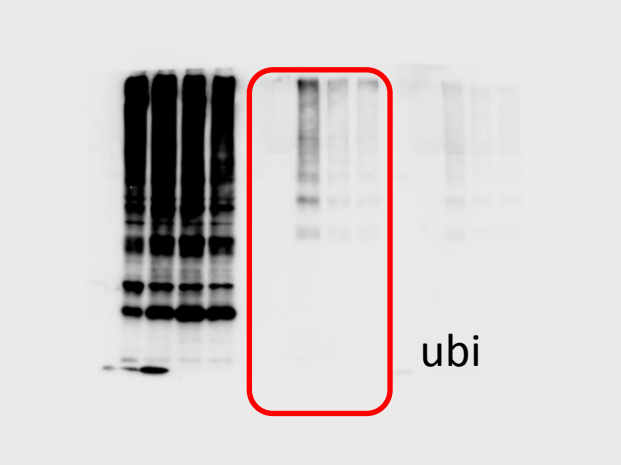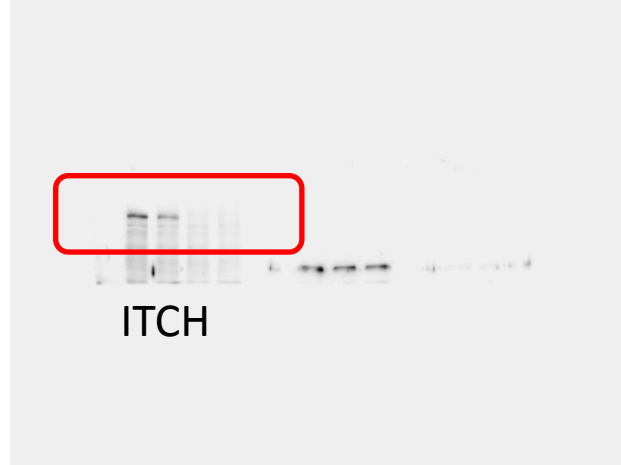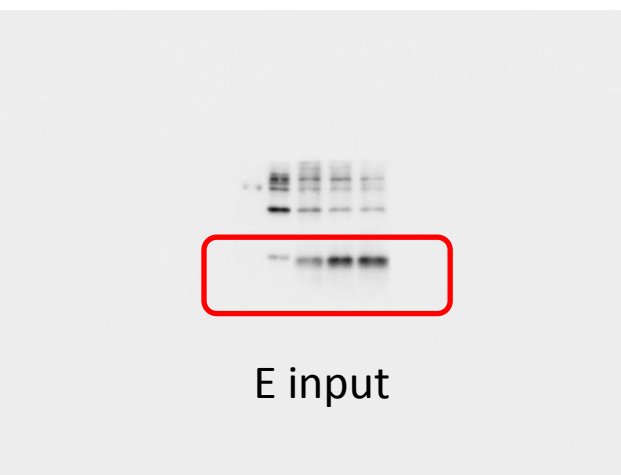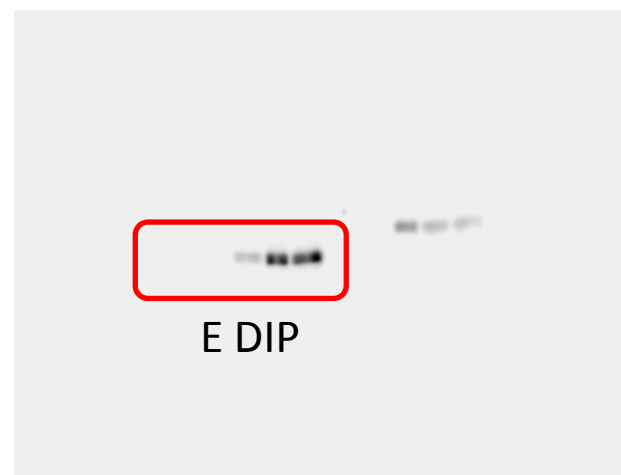

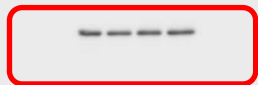

GADPH

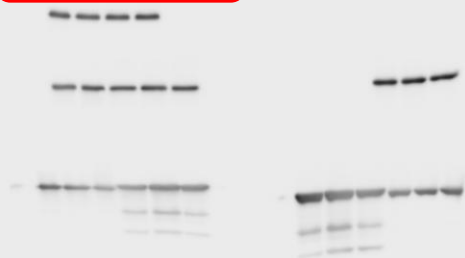

Supplement: Figure 1—source data 1. [file elife-105105-fig1-data1.zip › Figure 1C.pdf]

1D

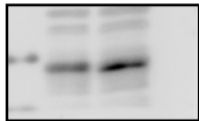

—15 kD

—10 kD

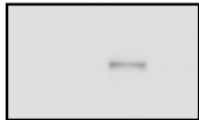

—100 kD

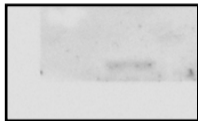

—15 kD

—10 kD

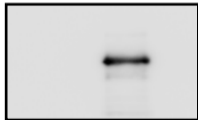

—100 kD

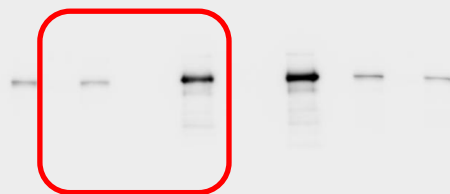

**ITCH**

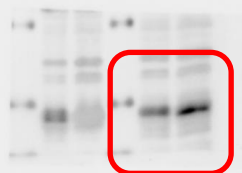

**E**

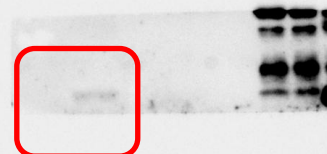

**E**

Supplement: Figure 1—source data 1. [file elife-105105-fig1-data1.zip › Figure 1D.pdf]

1E

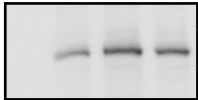

—180 kD

—100 kD

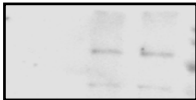

—180 kD

—100 kD

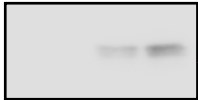

—10 kD

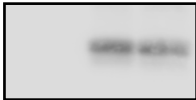

—10 kD

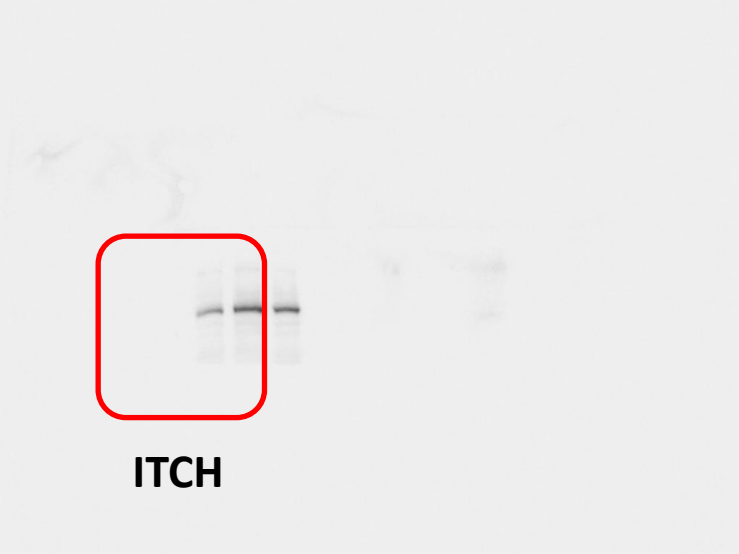

**ITCH**

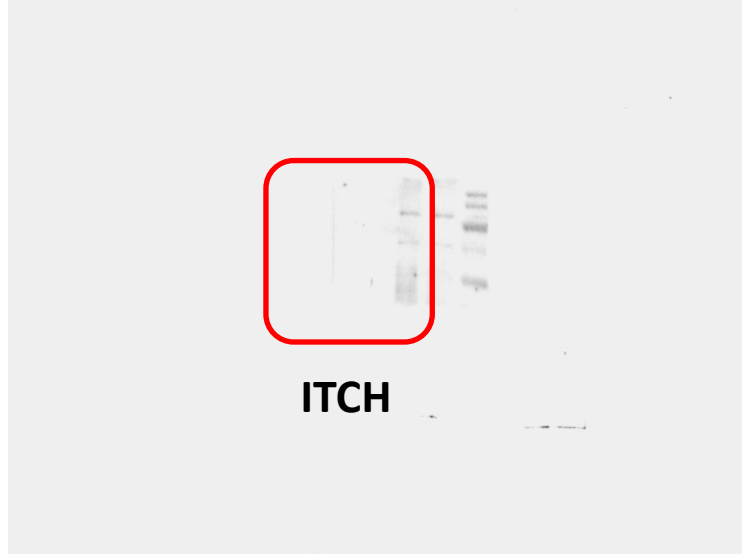

**ITCH**

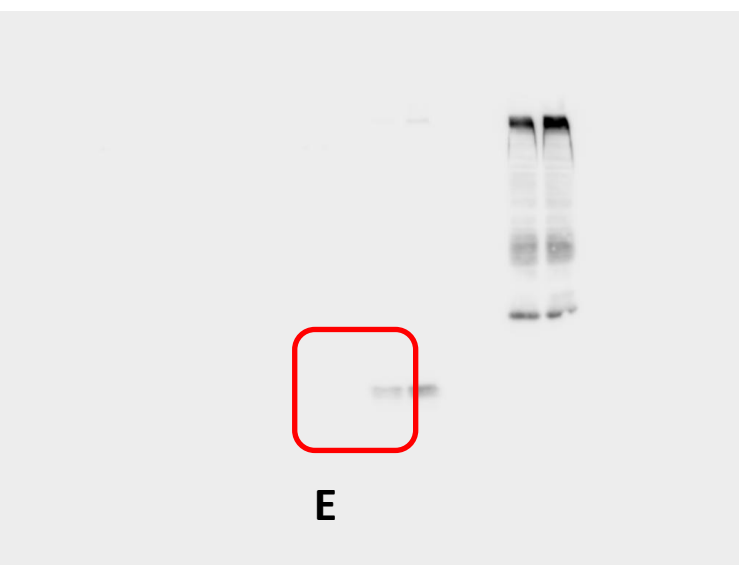

**E**

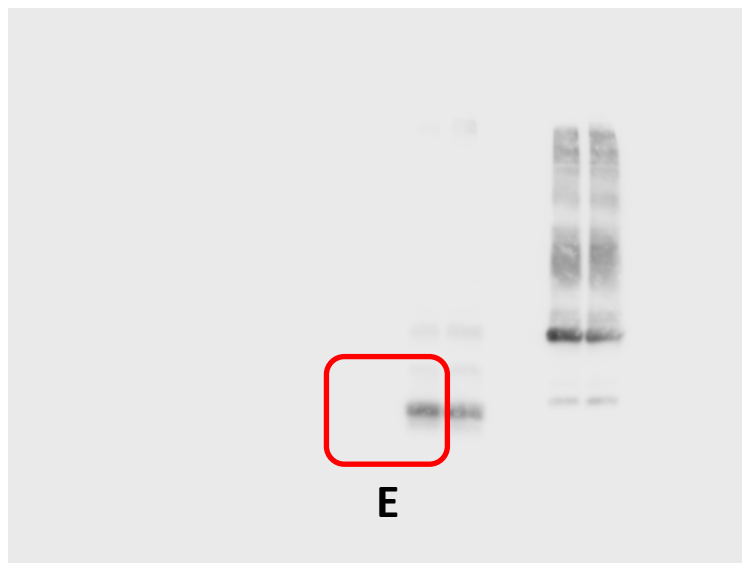

**E**

Supplement: Figure 1—source data 1. [file elife-105105-fig1-data1.zip › Figure 1E.pdf]

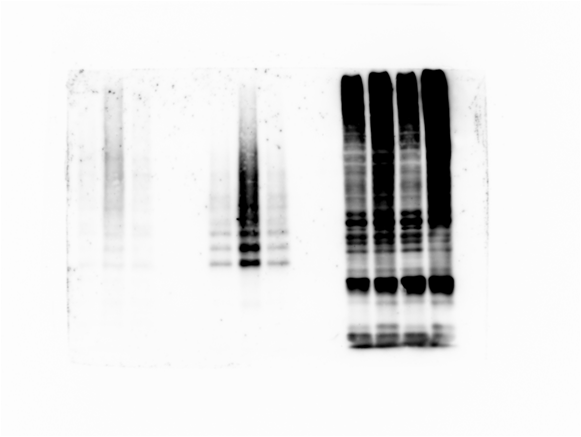

Supplement: Figure 1—source data 2. [file elife-105105-fig1-data2.zip › Figure 1B/1B.tif]

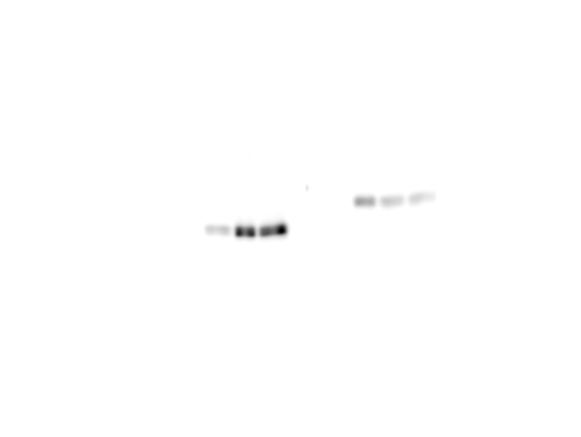

Supplement: Figure 1—source data 2. [file elife-105105-fig1-data2.zip › Figure 1C/E DIP.tif]

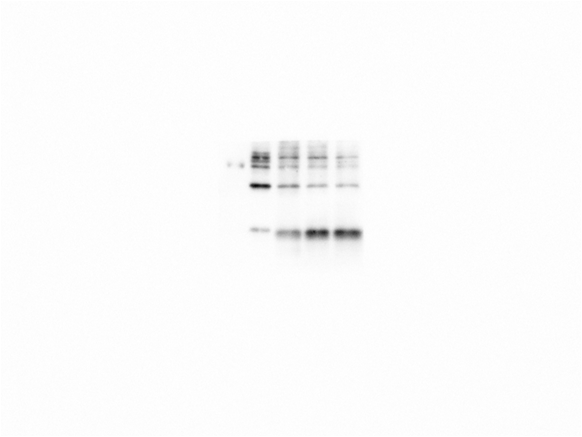

Supplement: Figure 1—source data 2. [file elife-105105-fig1-data2.zip › Figure 1C/E input.tif]

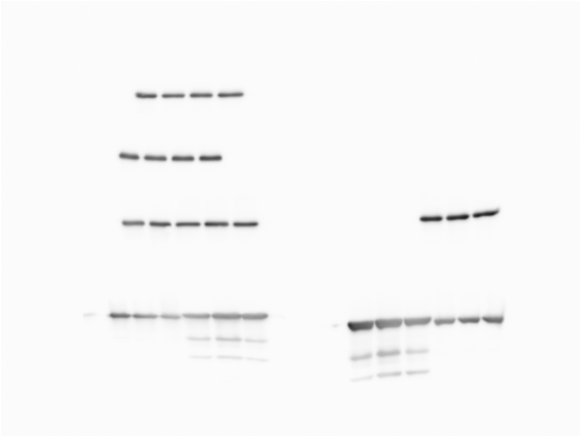

Supplement: Figure 1—source data 2. [file elife-105105-fig1-data2.zip › Figure 1C/gapdh.tif]

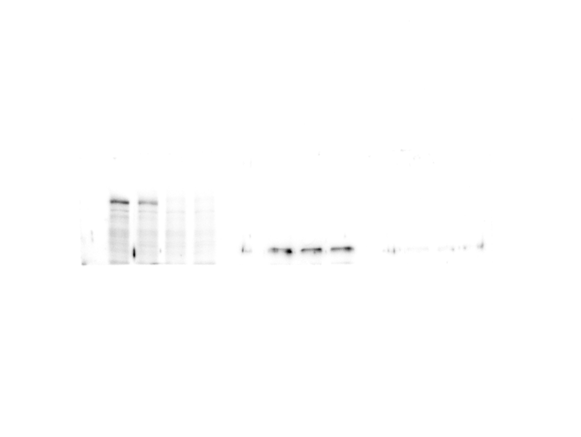

Supplement: Figure 1—source data 2. [file elife-105105-fig1-data2.zip › Figure 1C/itch.tif]

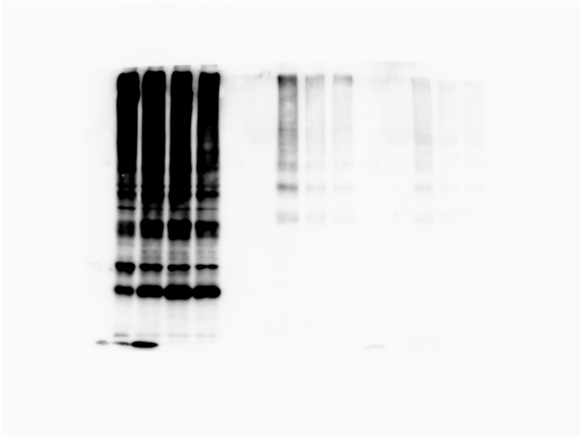

Supplement: Figure 1—source data 2. [file elife-105105-fig1-data2.zip › Figure 1C/Ubi E.tif]

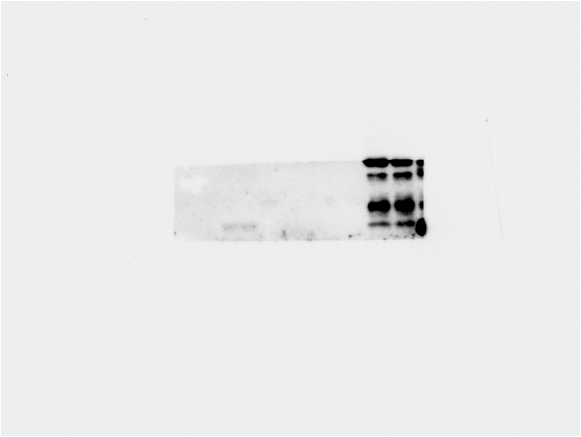

Supplement: Figure 1—source data 2. [file elife-105105-fig1-data2.zip › Figure 1D/E ip.tif]

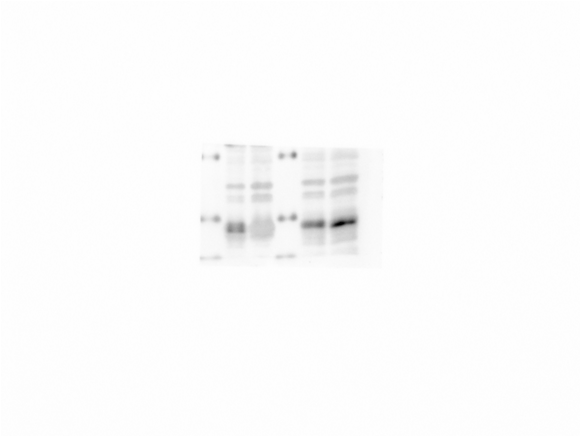

Supplement: Figure 1—source data 2. [file elife-105105-fig1-data2.zip › Figure 1D/E.tif]

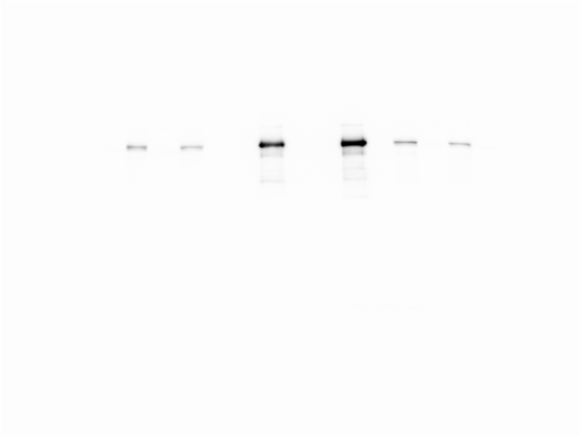

Supplement: Figure 1—source data 2. [file elife-105105-fig1-data2.zip › Figure 1D/itch.tif]

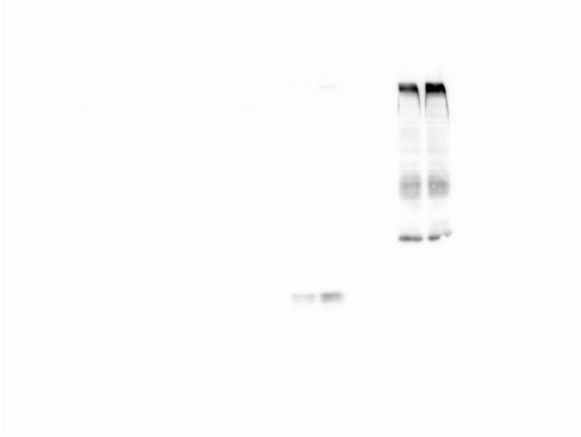

Supplement: Figure 1—source data 2. [file elife-105105-fig1-data2.zip › Figure 1E/e input.tif]

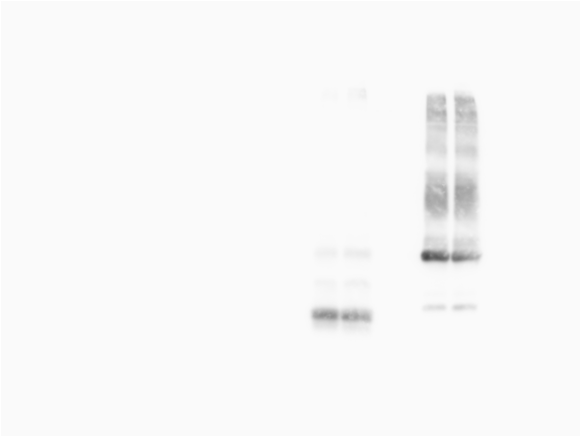

Supplement: Figure 1—source data 2. [file elife-105105-fig1-data2.zip › Figure 1E/e ip.tif]

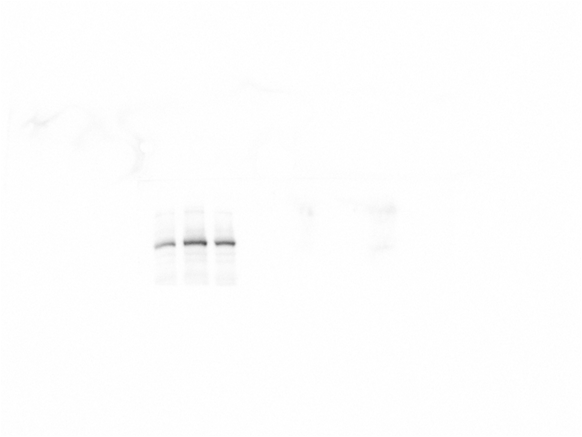

Supplement: Figure 1—source data 2. [file elife-105105-fig1-data2.zip › Figure 1E/ITCH 1E.tif]

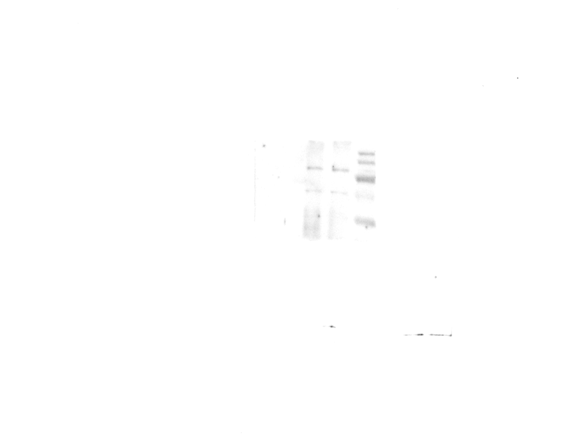

Supplement: Figure 1—source data 2. [file elife-105105-fig1-data2.zip › Figure 1E/itch ip.tif]

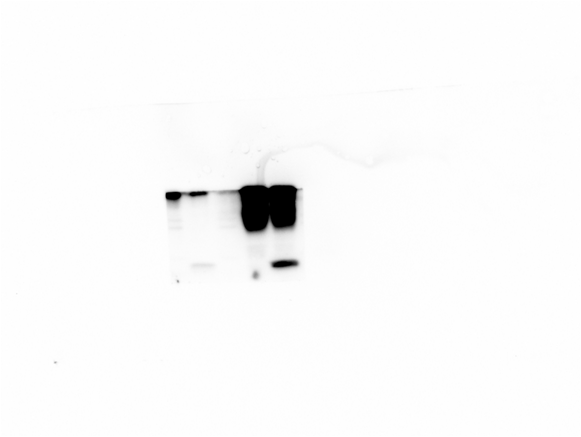

Supplement: Figure 1—source data 2. [file elife-105105-fig1-data2.zip › Figure 1G/e input.tif]

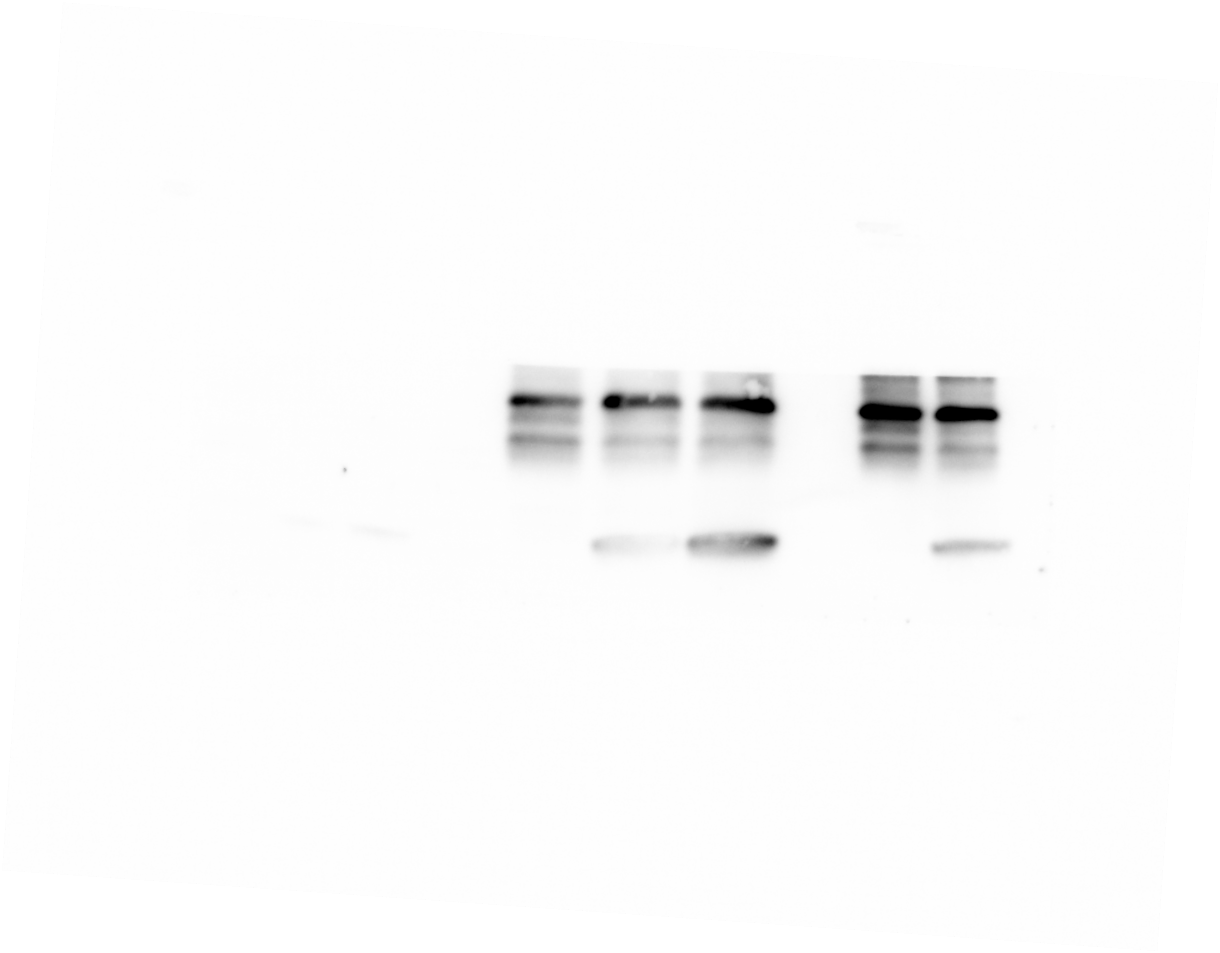

Supplement: Figure 1—source data 2. [file elife-105105-fig1-data2.zip › Figure 1G/e ip.tif]

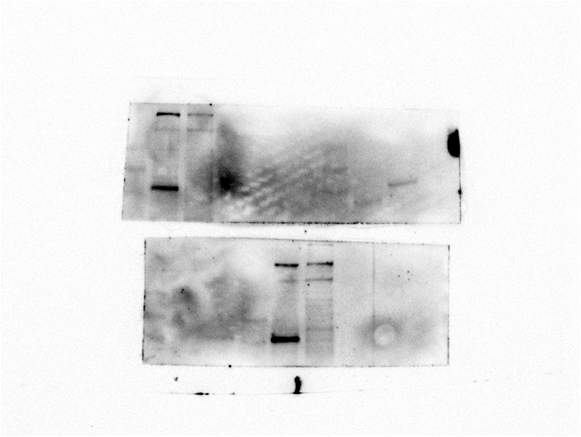

Supplement: Figure 1—source data 2. [file elife-105105-fig1-data2.zip › Figure 1G/itch.tif]

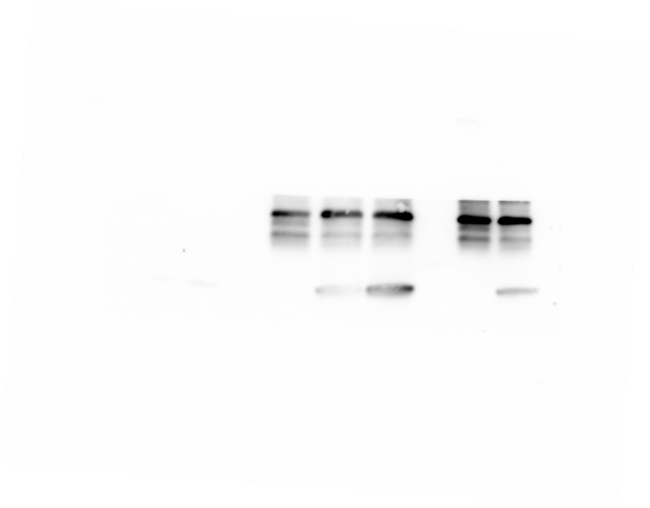

Supplement: Figure 1—source data 2. [file elife-105105-fig1-data2.zip › Figure 1H/E dip.tif]

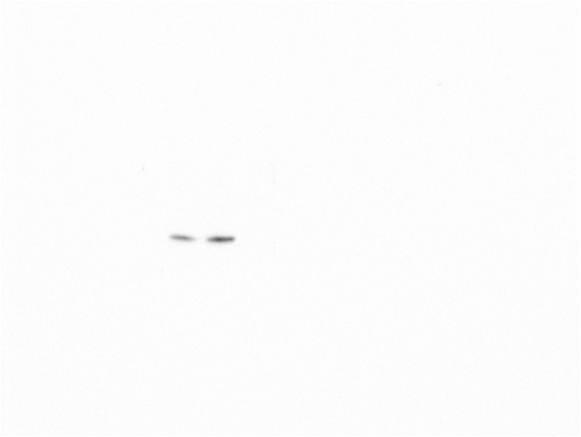

Supplement: Figure 1—source data 2. [file elife-105105-fig1-data2.zip › Figure 1H/E input.tif]

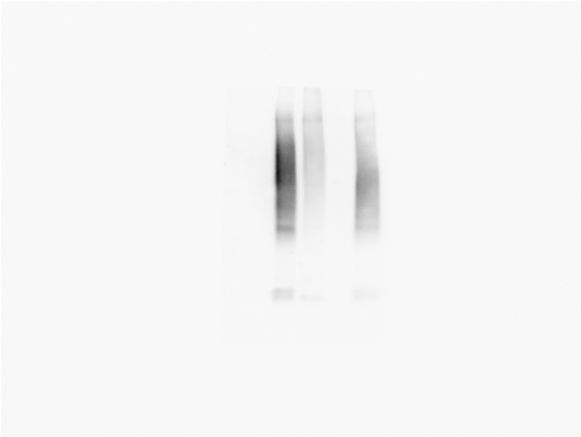

Supplement: Figure 1—source data 2. [file elife-105105-fig1-data2.zip › Figure 1H/e ubi.tif]

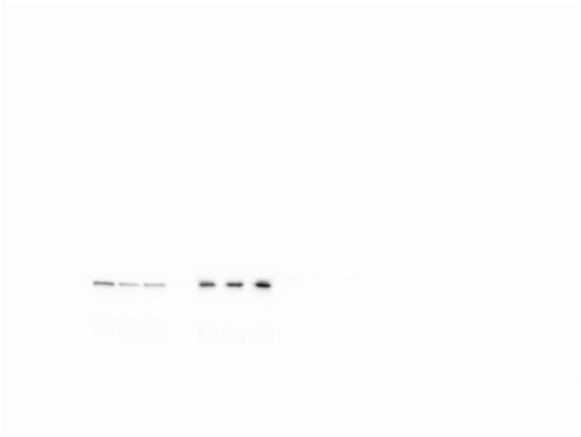

Supplement: Figure 1—source data 2. [file elife-105105-fig1-data2.zip › Figure 1H/gapdh.tif]

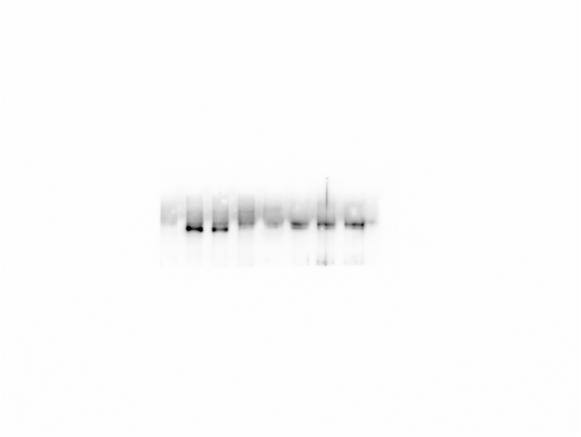

Supplement: Figure 1—source data 2. [file elife-105105-fig1-data2.zip › Figure 1H/itch.tif]

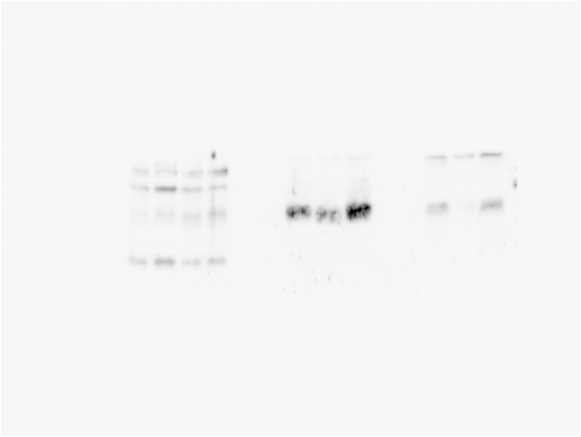

Supplement: Figure 1—source data 2. [file elife-105105-fig1-data2.zip › Figure 1A/e dip.tif]

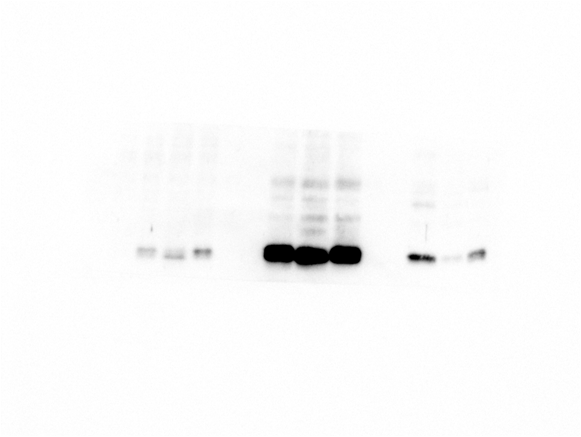

Supplement: Figure 1—source data 2. [file elife-105105-fig1-data2.zip › Figure 1A/E input.tif]

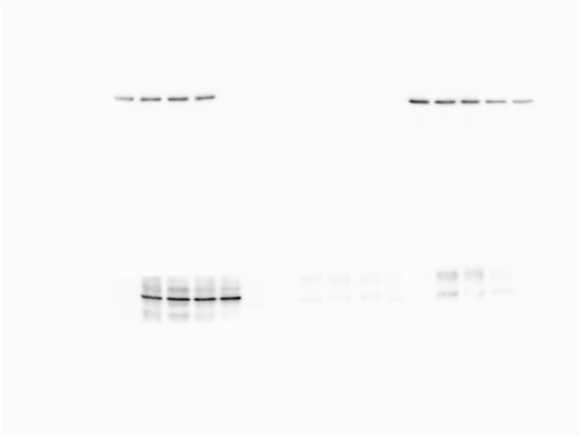

Supplement: Figure 1—source data 2. [file elife-105105-fig1-data2.zip › Figure 1A/gapdh.tif]

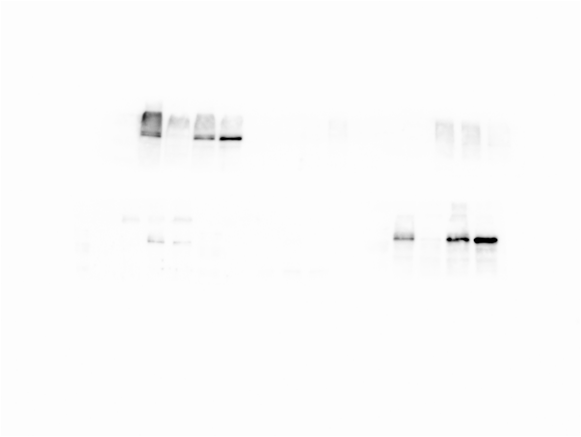

Supplement: Figure 1—source data 2. [file elife-105105-fig1-data2.zip › Figure 1A/ITCH.tif]

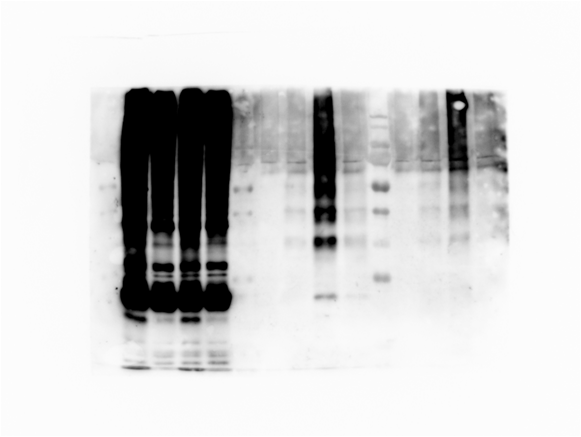

Supplement: Figure 1—source data 2. [file elife-105105-fig1-data2.zip › Figure 1A/ubi 1A.tif]

S1I

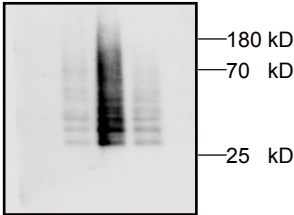

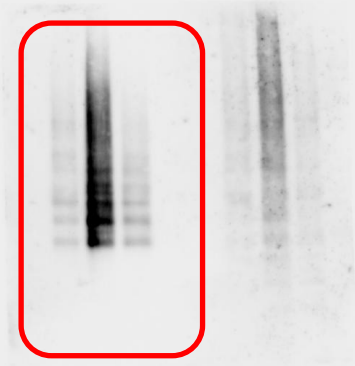

K63

Supplement: Figure 1—figure supplement 1—source data 1. [file elife-105105-fig1-figsupp1-data1.zip › Figure 1-Figure supplement 1I.pdf]

S1J

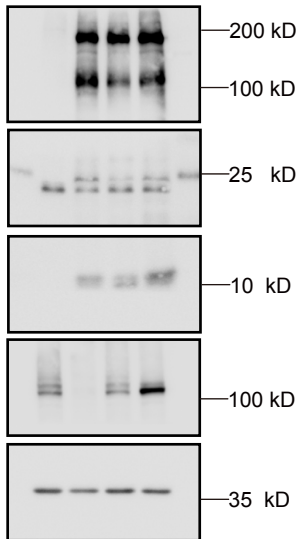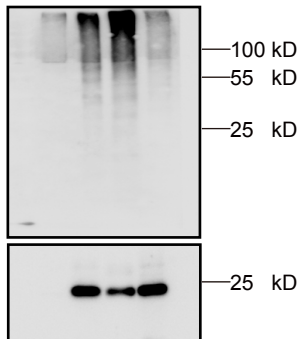

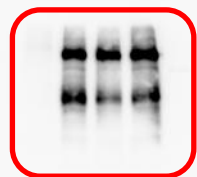

spike

M

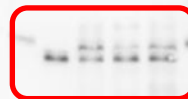

E

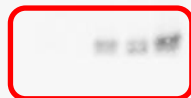

ITCH

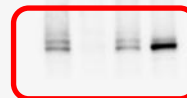

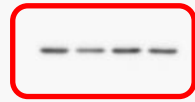

gapdh

M

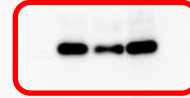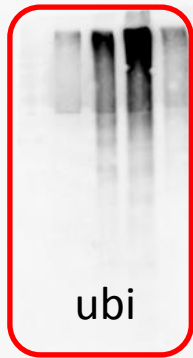

ubi

Supplement: Figure 1—figure supplement 1—source data 1. [file elife-105105-fig1-figsupp1-data1.zip › Figure 1-Figure supplement 1J.pdf]

# S1K

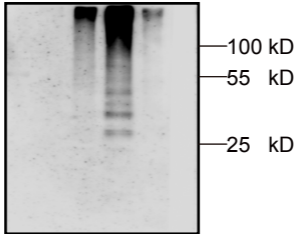

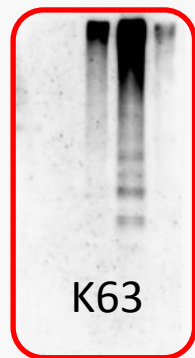

Supplement: Figure 1—figure supplement 1—source data 1. [file elife-105105-fig1-figsupp1-data1.zip › Figure 1-Figure supplement 1K.pdf]

S1D

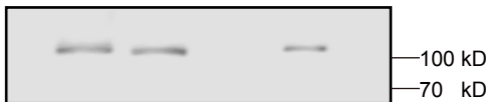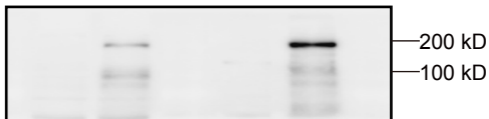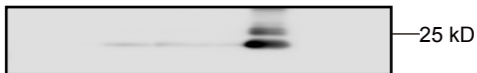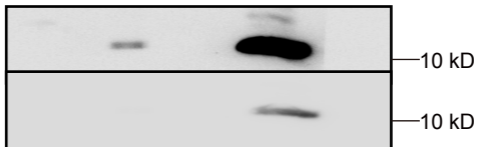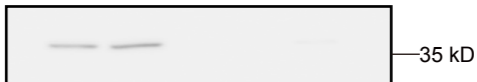

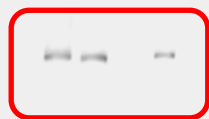

ITCH

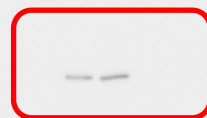

GAPDH

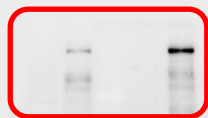

Spike

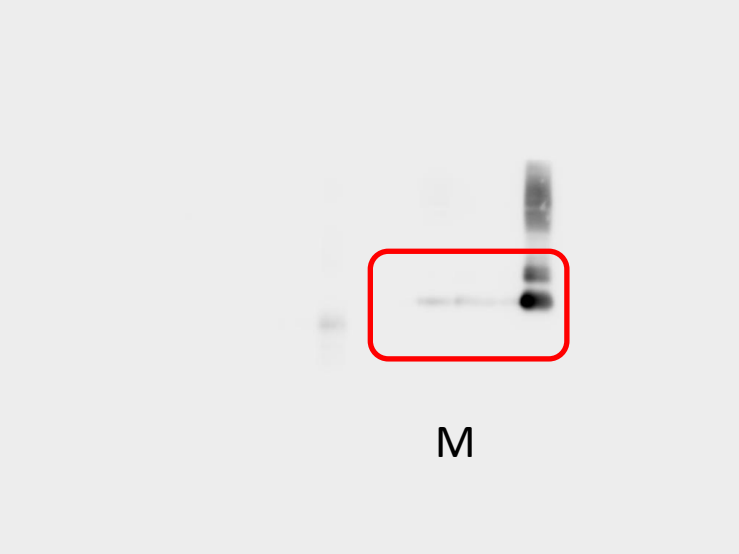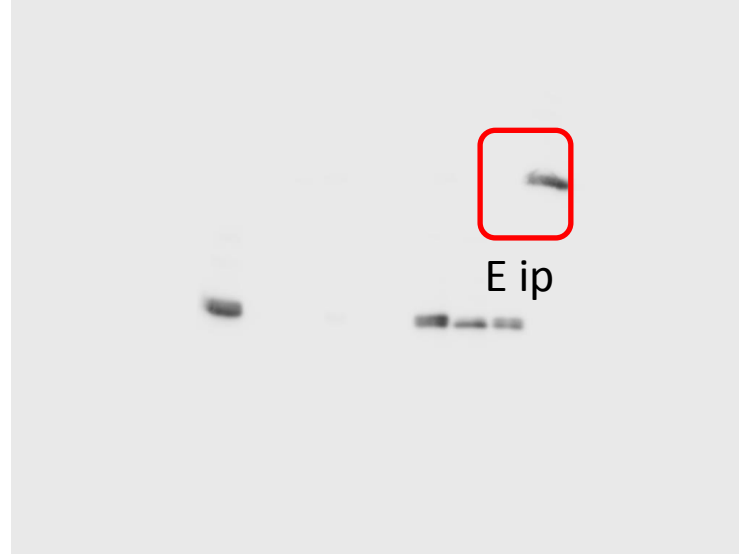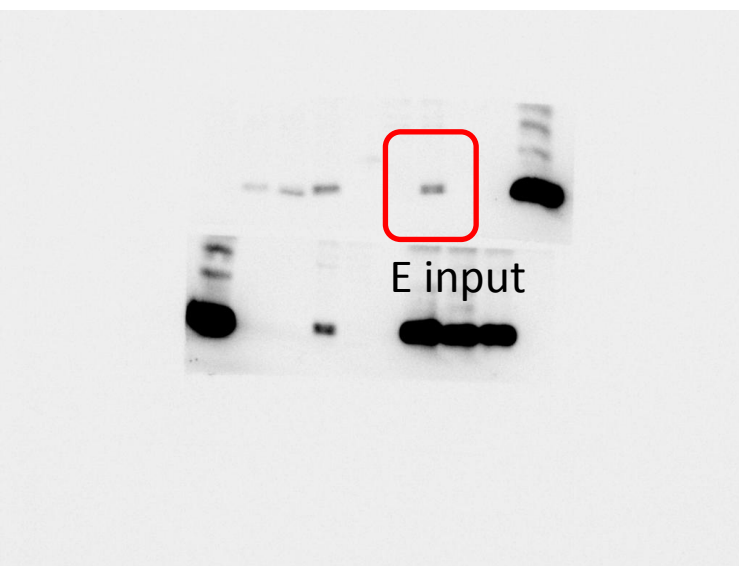

Supplement: Figure 1—figure supplement 1—source data 1. [file elife-105105-fig1-figsupp1-data1.zip › Figure 1-Figure supplement 1D.pdf]

# S1E

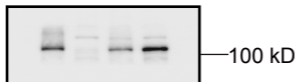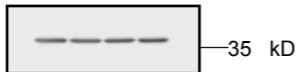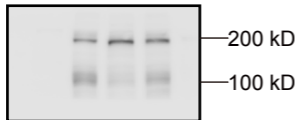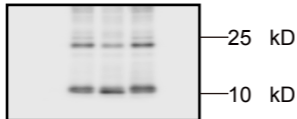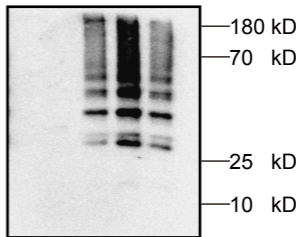

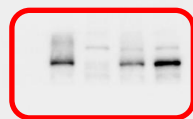

ITCH

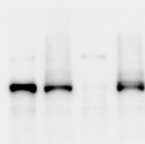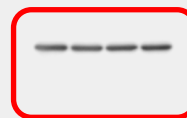

GAPDH

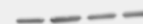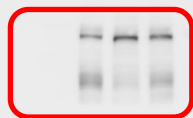

Spike

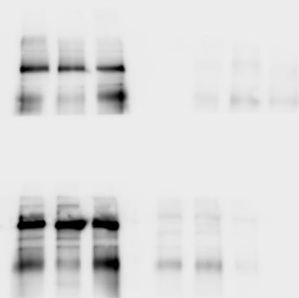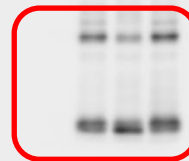

E+M

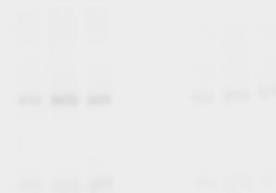

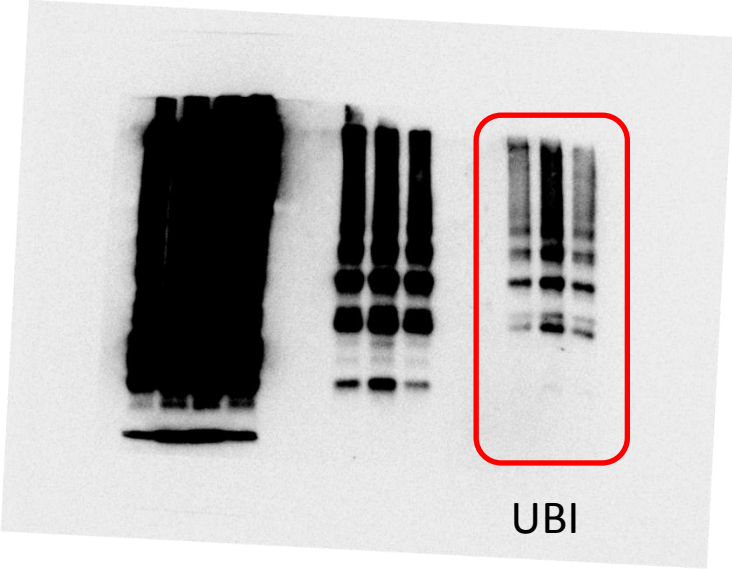

Supplement: Figure 1—figure supplement 1—source data 1. [file elife-105105-fig1-figsupp1-data1.zip › Figure 1-Figure supplement 1E.pdf]

S1F

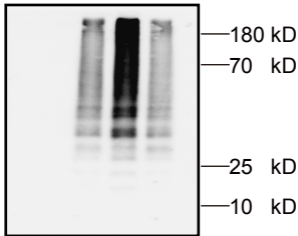

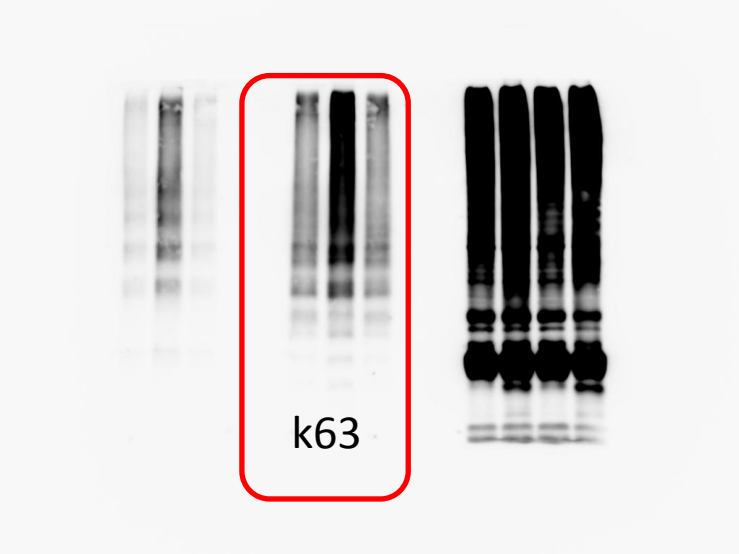

Supplement: Figure 1—figure supplement 1—source data 1. [file elife-105105-fig1-figsupp1-data1.zip › Figure 1-Figure supplement 1F.pdf]

# S1G

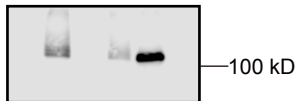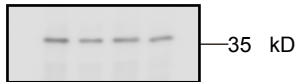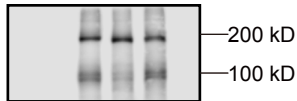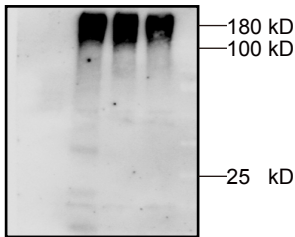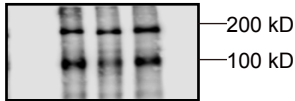

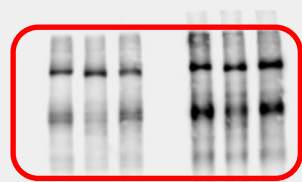

Spike

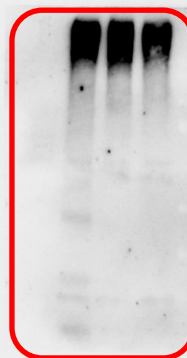

Ubi

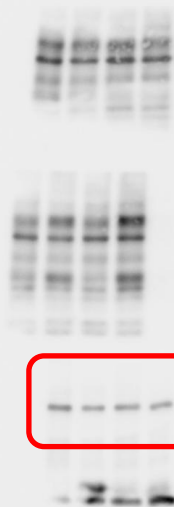

gapdh

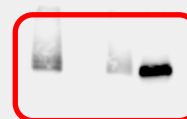

ITCH

Supplement: Figure 1—figure supplement 1—source data 1. [file elife-105105-fig1-figsupp1-data1.zip › Figure 1-Figure supplement 1G.pdf]

S1H

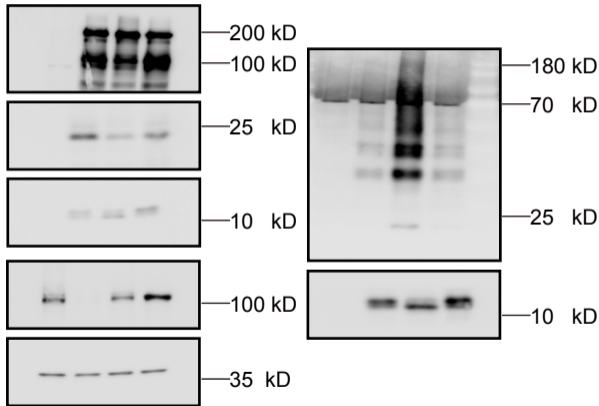

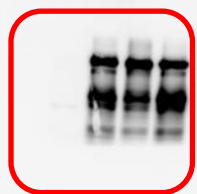

Spike

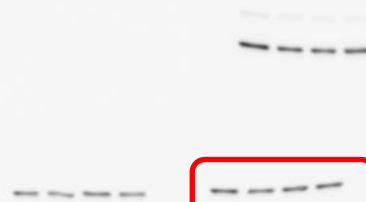

GAPDH

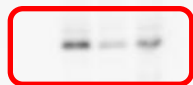

M

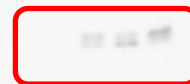

E

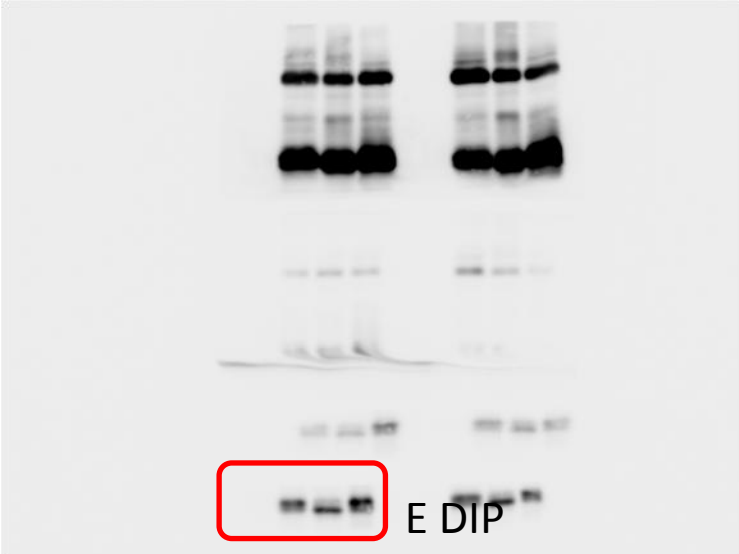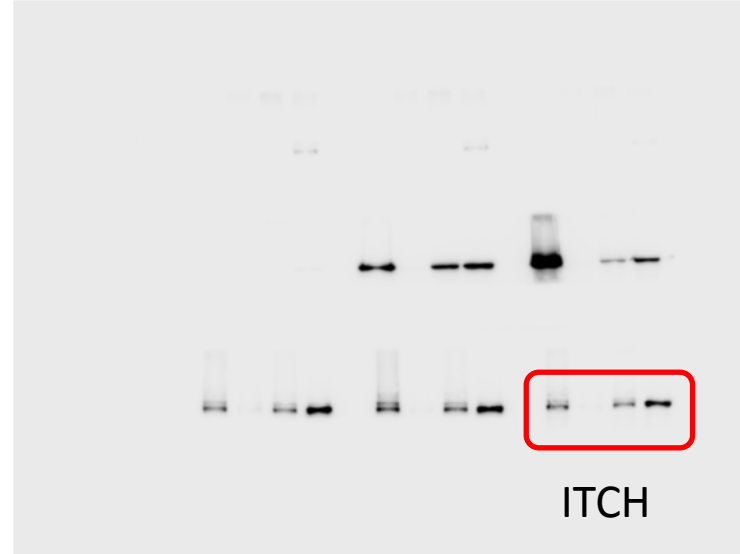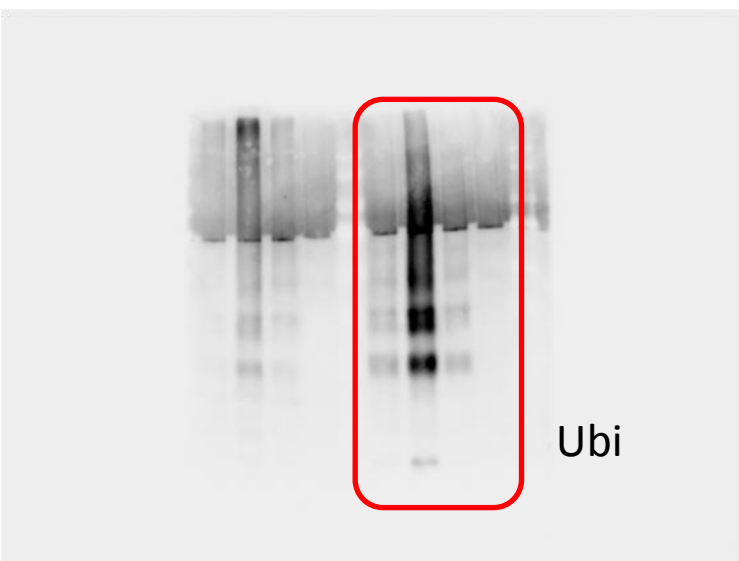

Supplement: Figure 1—figure supplement 1—source data 1. [file elife-105105-fig1-figsupp1-data1.zip › Figure 1-Figure supplement 1H.pdf]

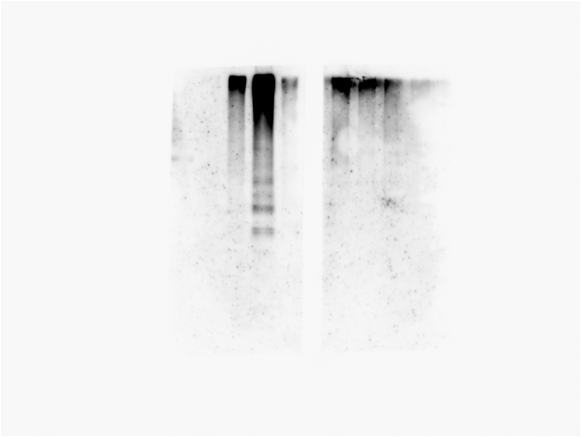

Supplement: Figure 1—figure supplement 1—source data 2. [file elife-105105-fig1-figsupp1-data2.zip › Figure 1-Figure supplement 1K/M k63.tif]

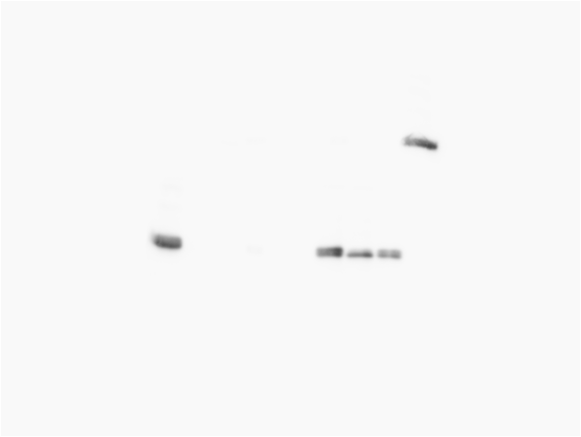

Supplement: Figure 1—figure supplement 1—source data 2. [file elife-105105-fig1-figsupp1-data2.zip › Figure 1-Figure supplement 1D/E IP.tif]

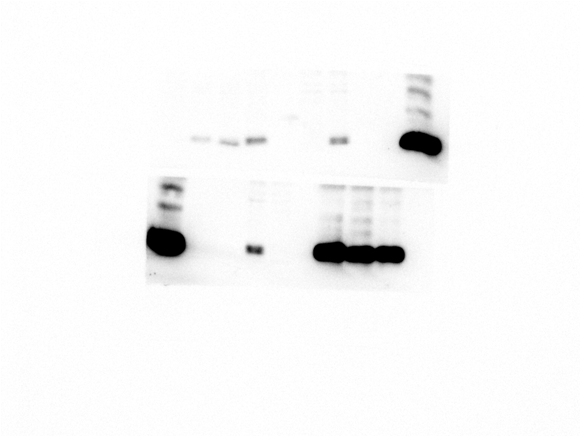

Supplement: Figure 1—figure supplement 1—source data 2. [file elife-105105-fig1-figsupp1-data2.zip › Figure 1-Figure supplement 1D/E.tif]

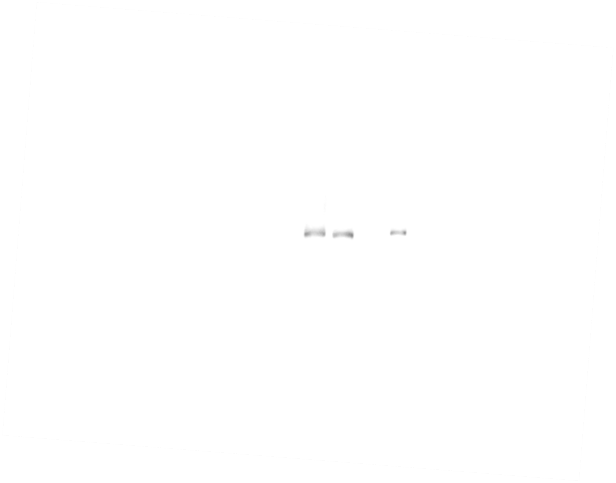

Supplement: Figure 1—figure supplement 1—source data 2. [file elife-105105-fig1-figsupp1-data2.zip › Figure 1-Figure supplement 1D/ITCH.tif]

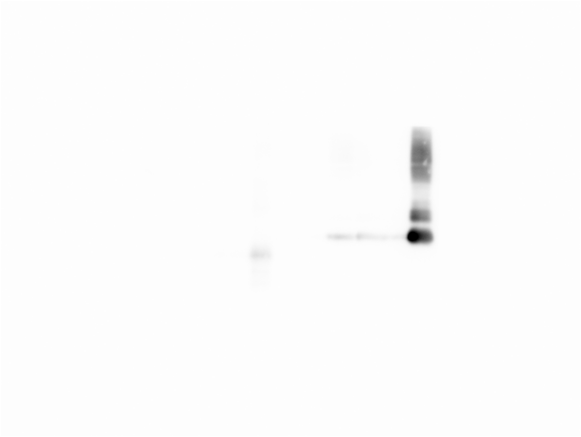

Supplement: Figure 1—figure supplement 1—source data 2. [file elife-105105-fig1-figsupp1-data2.zip › Figure 1-Figure supplement 1D/M.tif]

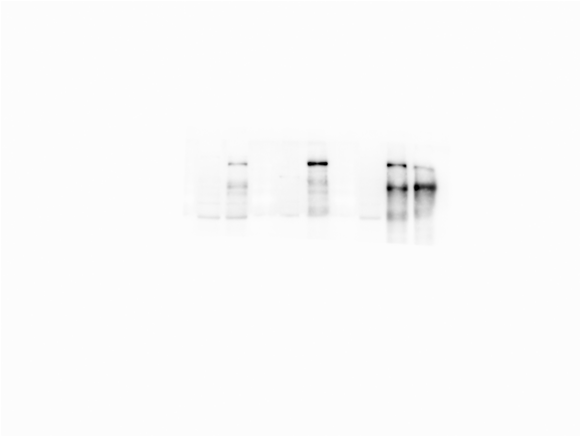

Supplement: Figure 1—figure supplement 1—source data 2. [file elife-105105-fig1-figsupp1-data2.zip › Figure 1-Figure supplement 1D/spike.tif]

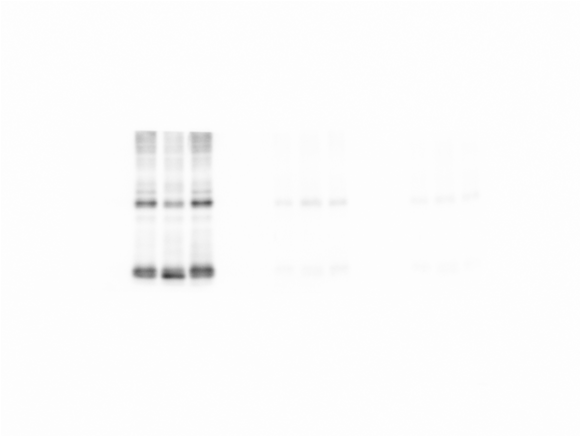

Supplement: Figure 1—figure supplement 1—source data 2. [file elife-105105-fig1-figsupp1-data2.zip › Figure 1-Figure supplement 1E/E M.tif]

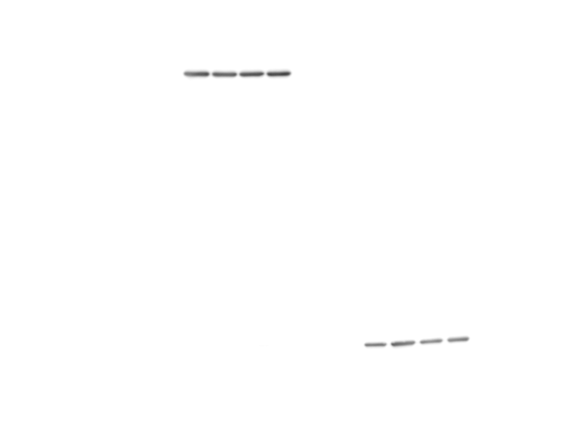

Supplement: Figure 1—figure supplement 1—source data 2. [file elife-105105-fig1-figsupp1-data2.zip › Figure 1-Figure supplement 1E/gapdh.tif]

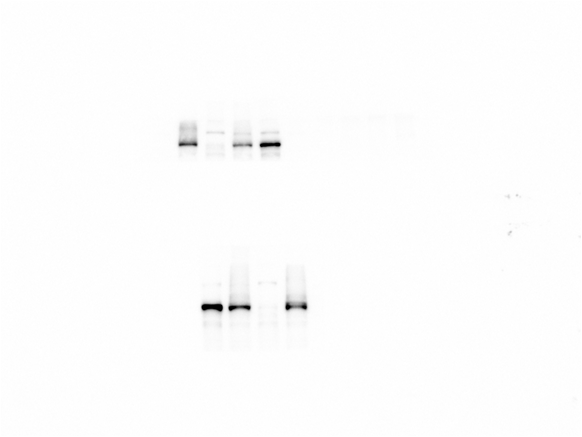

Supplement: Figure 1—figure supplement 1—source data 2. [file elife-105105-fig1-figsupp1-data2.zip › Figure 1-Figure supplement 1E/ITCH.tif]

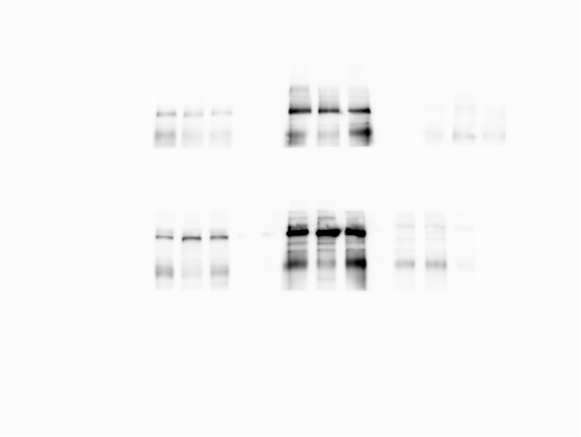

Supplement: Figure 1—figure supplement 1—source data 2. [file elife-105105-fig1-figsupp1-data2.zip › Figure 1-Figure supplement 1E/spike.tif]

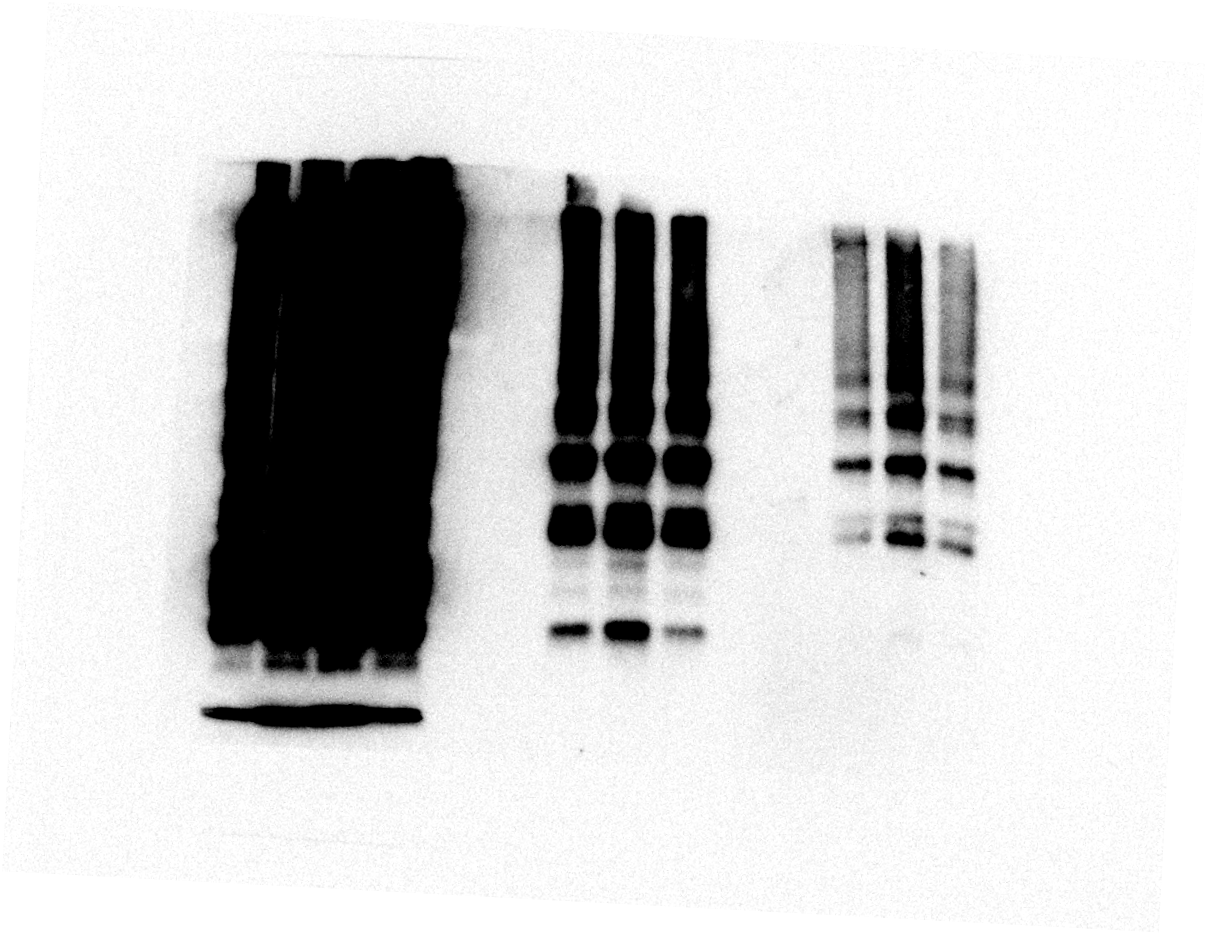

Supplement: Figure 1—figure supplement 1—source data 2. [file elife-105105-fig1-figsupp1-data2.zip › Figure 1-Figure supplement 1E/UBI.tif]

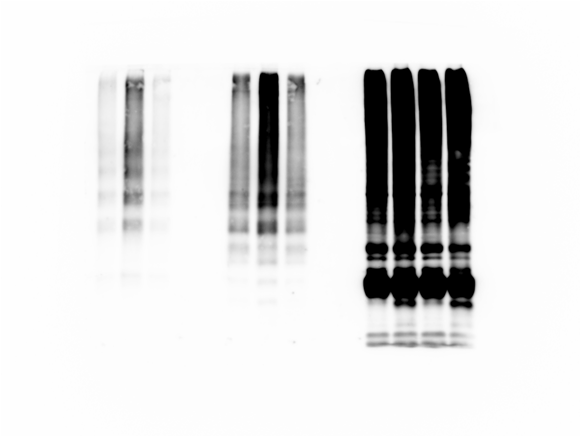

Supplement: Figure 1—figure supplement 1—source data 2. [file elife-105105-fig1-figsupp1-data2.zip › Figure 1-Figure supplement 1F/k63.tif]

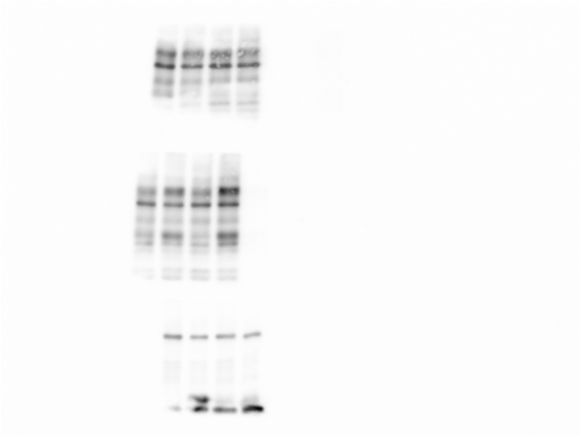

Supplement: Figure 1—figure supplement 1—source data 2. [file elife-105105-fig1-figsupp1-data2.zip › Figure 1-Figure supplement 1G/gapdh.tif]

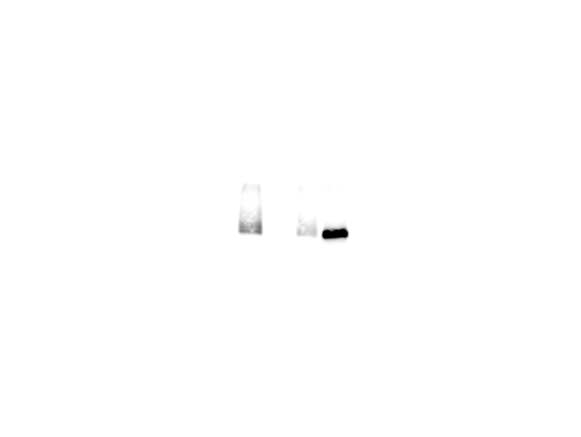

Supplement: Figure 1—figure supplement 1—source data 2. [file elife-105105-fig1-figsupp1-data2.zip › Figure 1-Figure supplement 1G/itch.tif]

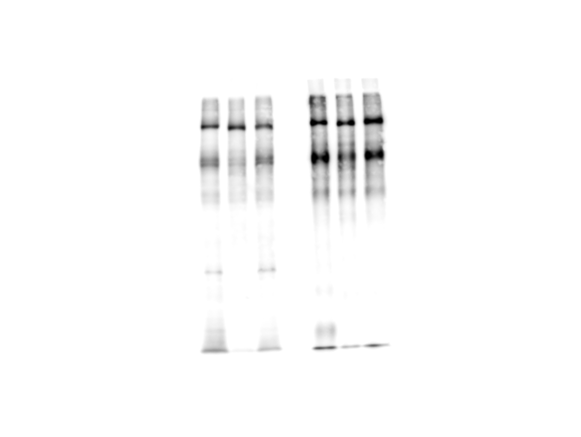

Supplement: Figure 1—figure supplement 1—source data 2. [file elife-105105-fig1-figsupp1-data2.zip › Figure 1-Figure supplement 1G/spike.tif]

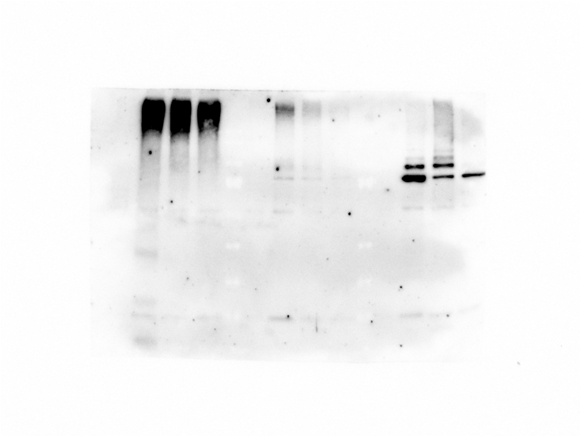

Supplement: Figure 1—figure supplement 1—source data 2. [file elife-105105-fig1-figsupp1-data2.zip › Figure 1-Figure supplement 1G/ubi.tif]

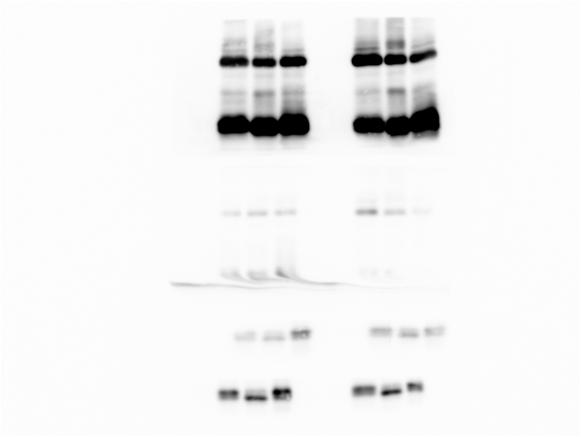

Supplement: Figure 1—figure supplement 1—source data 2. [file elife-105105-fig1-figsupp1-data2.zip › Figure 1-Figure supplement 1H/E ip.tif]

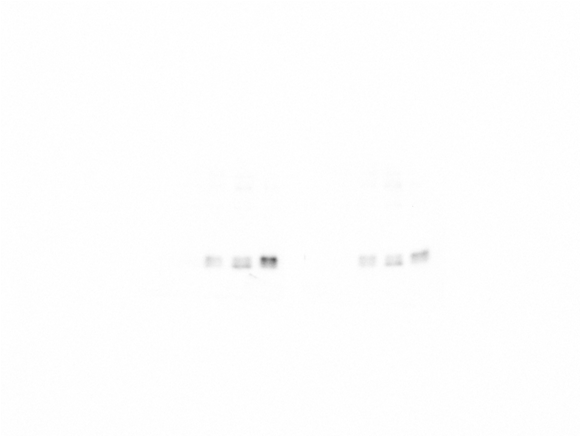

Supplement: Figure 1—figure supplement 1—source data 2. [file elife-105105-fig1-figsupp1-data2.zip › Figure 1-Figure supplement 1H/E.tif]

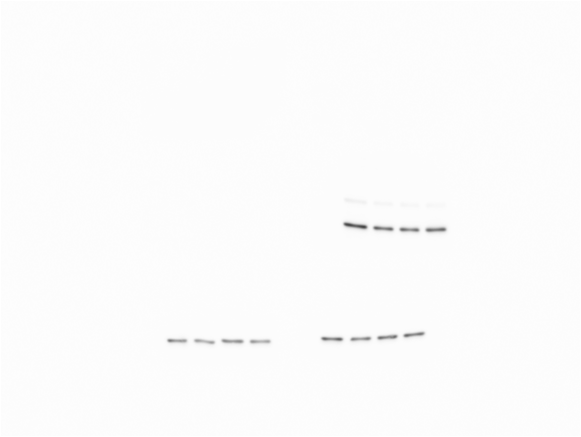

Supplement: Figure 1—figure supplement 1—source data 2. [file elife-105105-fig1-figsupp1-data2.zip › Figure 1-Figure supplement 1H/GAPDH.tif]

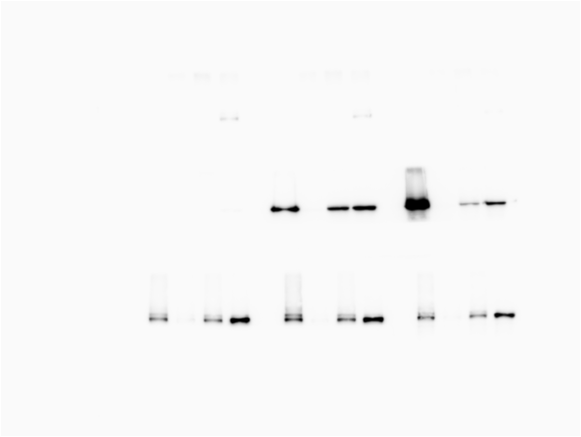

Supplement: Figure 1—figure supplement 1—source data 2. [file elife-105105-fig1-figsupp1-data2.zip › Figure 1-Figure supplement 1H/ITCH.tif]

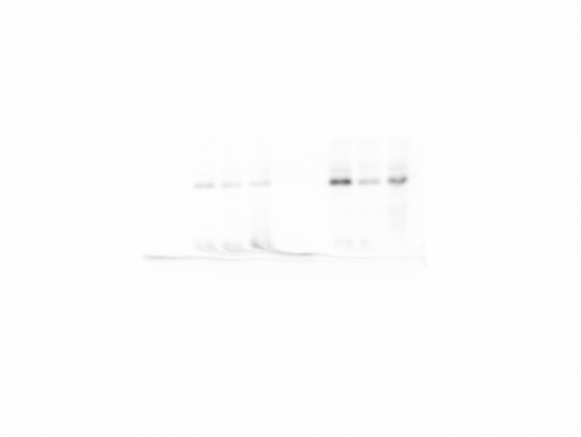

Supplement: Figure 1—figure supplement 1—source data 2. [file elife-105105-fig1-figsupp1-data2.zip › Figure 1-Figure supplement 1H/M.tif]

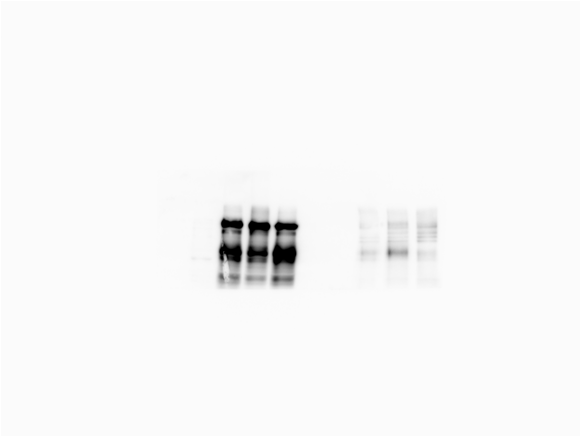

Supplement: Figure 1—figure supplement 1—source data 2. [file elife-105105-fig1-figsupp1-data2.zip › Figure 1-Figure supplement 1H/spike.tif]

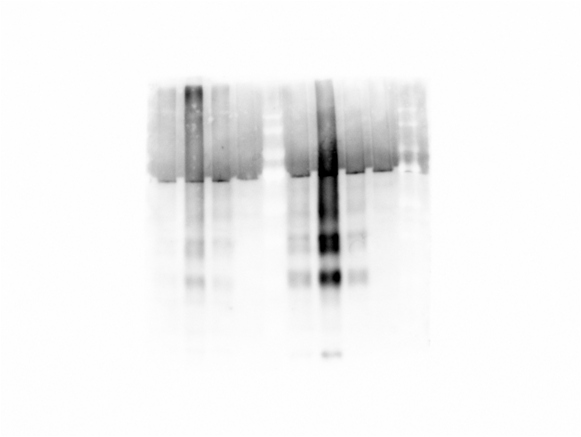

Supplement: Figure 1—figure supplement 1—source data 2. [file elife-105105-fig1-figsupp1-data2.zip › Figure 1-Figure supplement 1H/ubi S1H.tif]

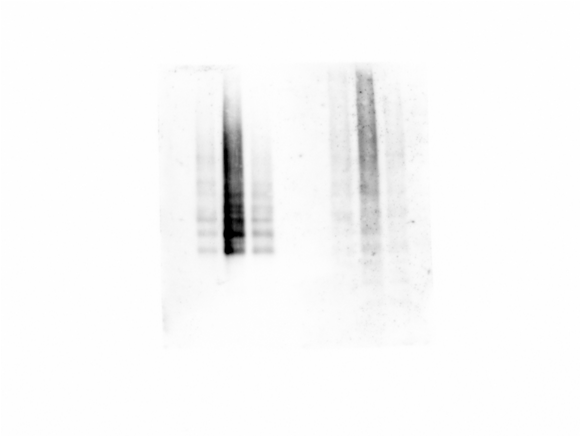

Supplement: Figure 1—figure supplement 1—source data 2. [file elife-105105-fig1-figsupp1-data2.zip › Figure 1-Figure supplement 1I/E k63.tif]

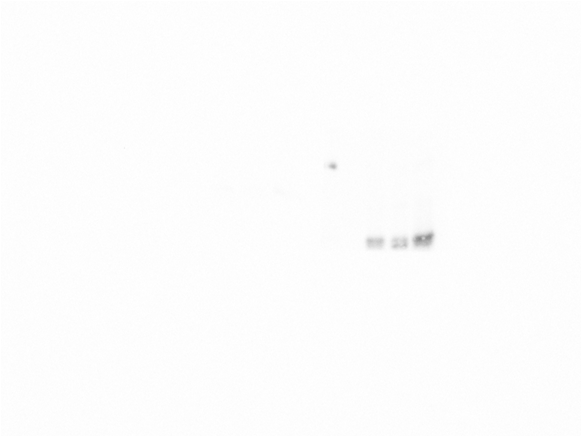

Supplement: Figure 1—figure supplement 1—source data 2. [file elife-105105-fig1-figsupp1-data2.zip › Figure 1-Figure supplement 1J/E.tif]

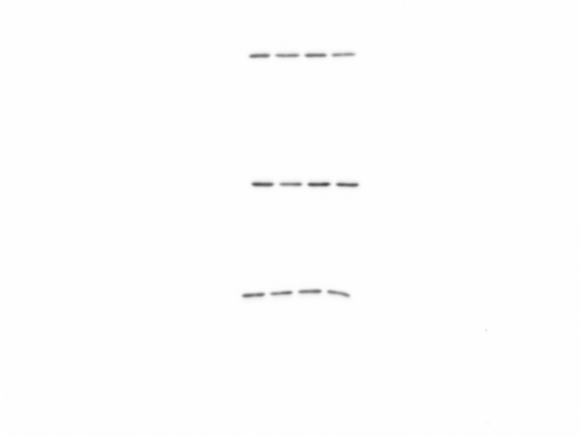

Supplement: Figure 1—figure supplement 1—source data 2. [file elife-105105-fig1-figsupp1-data2.zip › Figure 1-Figure supplement 1J/GAPDH.png]

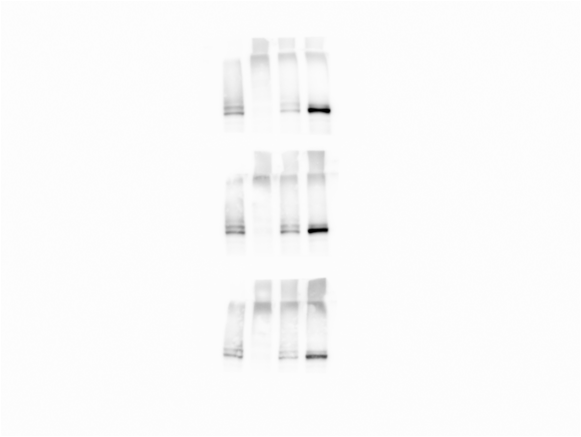

Supplement: Figure 1—figure supplement 1—source data 2. [file elife-105105-fig1-figsupp1-data2.zip › Figure 1-Figure supplement 1J/ITCH.tif]

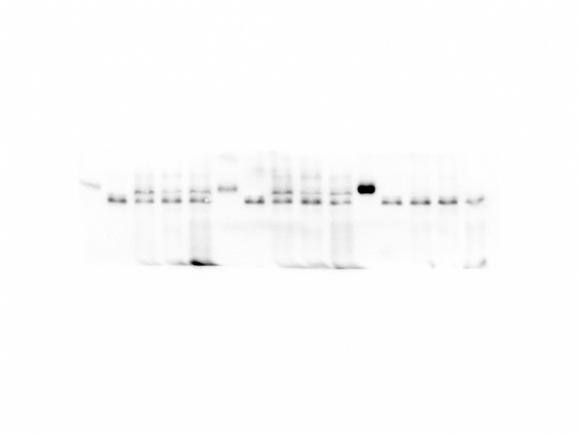

Supplement: Figure 1—figure supplement 1—source data 2. [file elife-105105-fig1-figsupp1-data2.zip › Figure 1-Figure supplement 1J/M.tif]

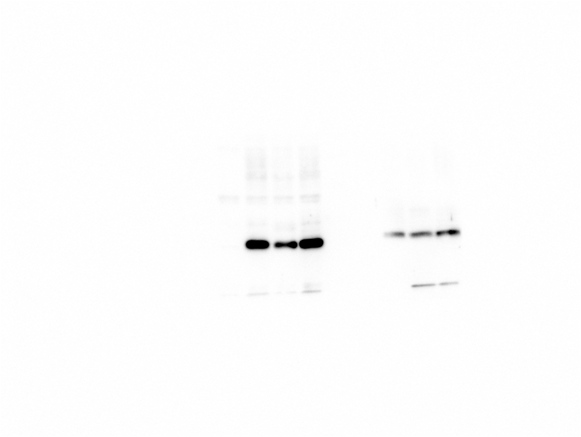

Supplement: Figure 1—figure supplement 1—source data 2. [file elife-105105-fig1-figsupp1-data2.zip › Figure 1-Figure supplement 1J/S tag M.tif]

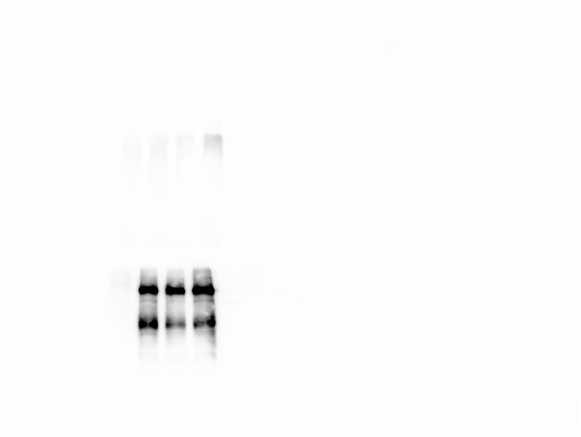

Supplement: Figure 1—figure supplement 1—source data 2. [file elife-105105-fig1-figsupp1-data2.zip › Figure 1-Figure supplement 1J/Spike.tif]

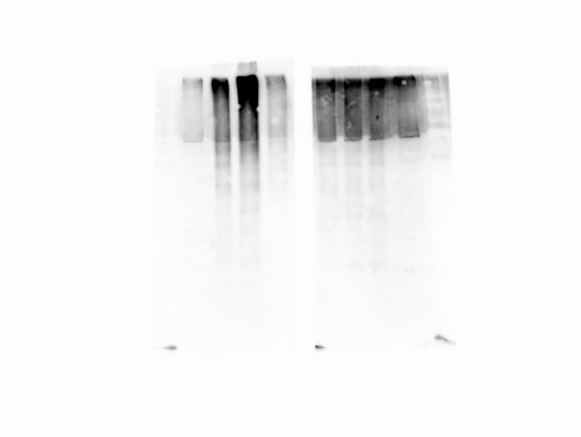

Supplement: Figure 1—figure supplement 1—source data 2. [file elife-105105-fig1-figsupp1-data2.zip › Figure 1-Figure supplement 1J/ubi M.tif]

# S2C

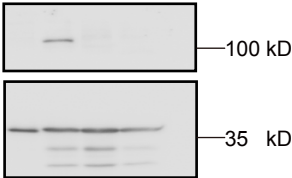

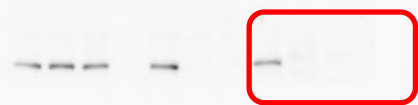

ITCH

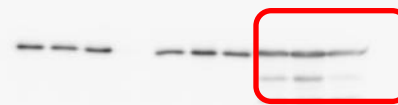

GAPDH

Supplement: Figure 1—figure supplement 2—source data 1. [file elife-105105-fig1-figsupp2-data1.zip › Figure 1-figure supplement 2C.pdf]

# S2D

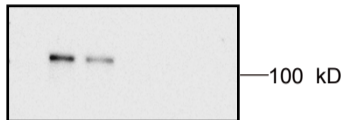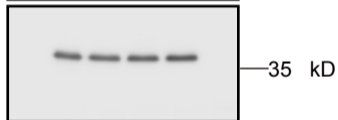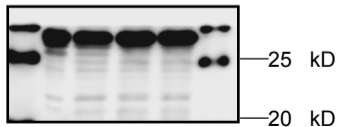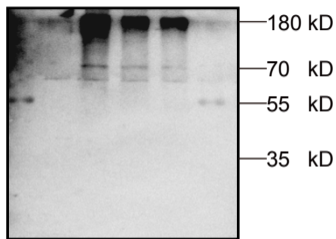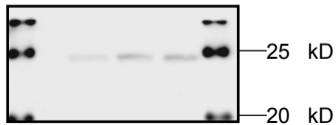

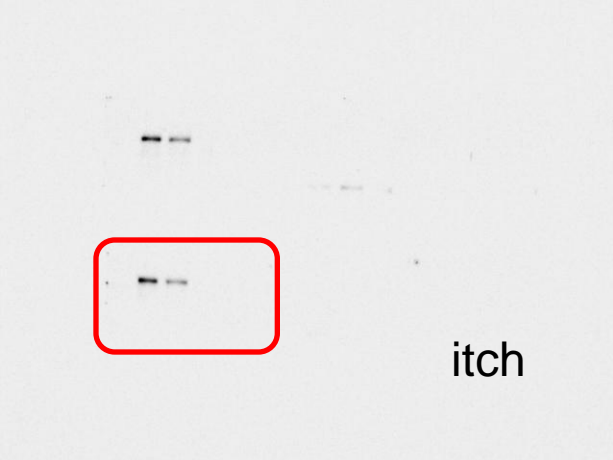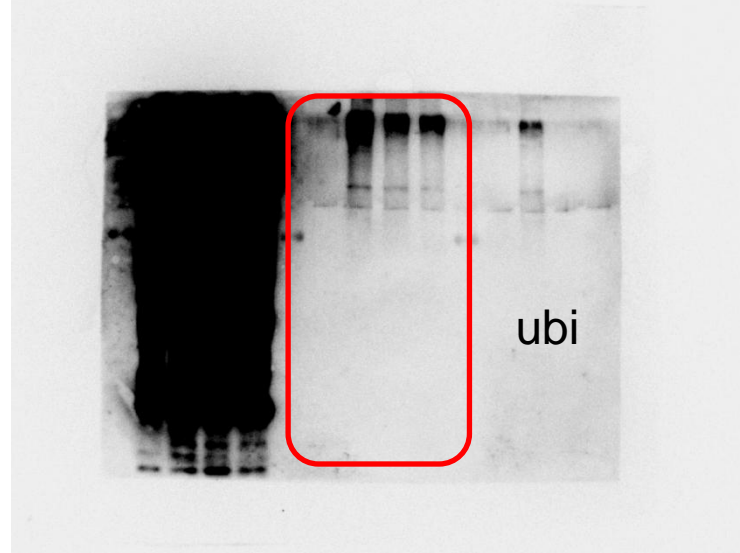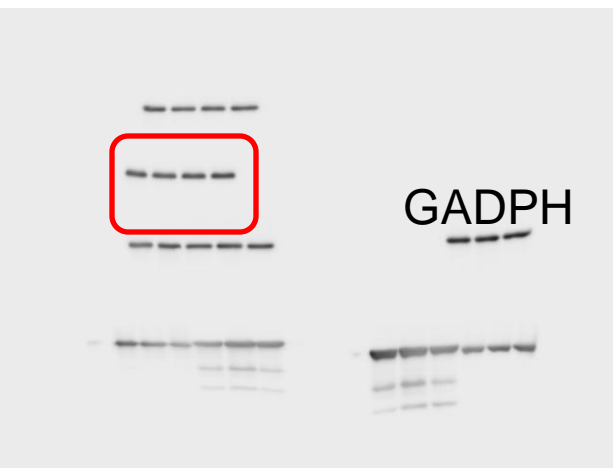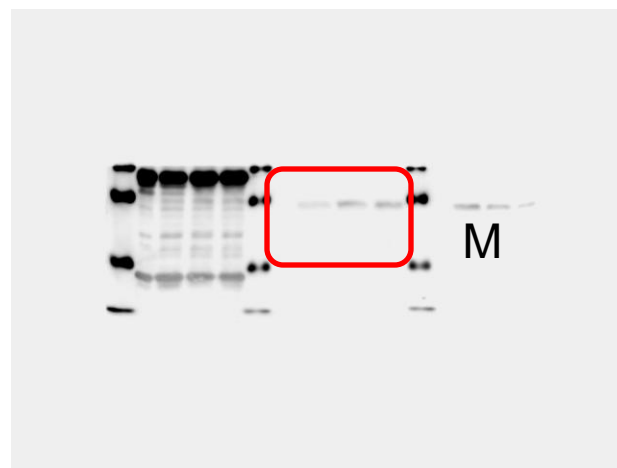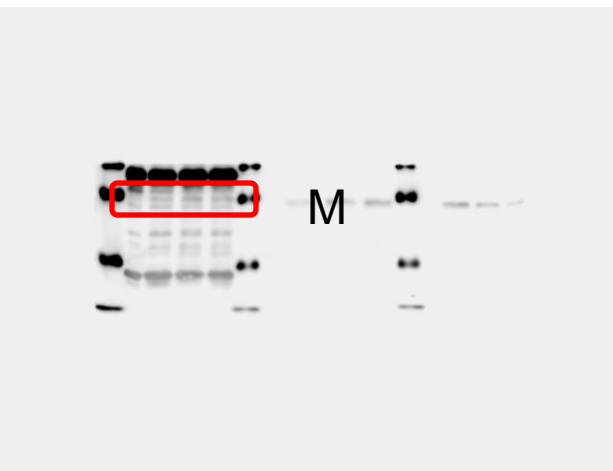

Supplement: Figure 1—figure supplement 2—source data 1. [file elife-105105-fig1-figsupp2-data1.zip › Figure 1-figure supplement 2D.pdf]

S2E

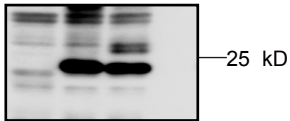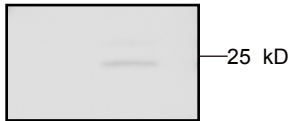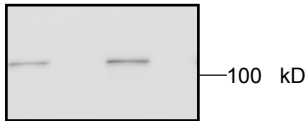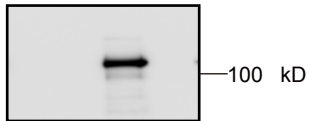

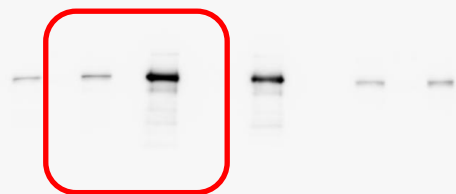

ITCH

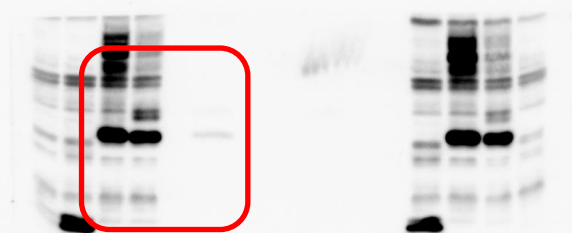

M

Supplement: Figure 1—figure supplement 2—source data 1. [file elife-105105-fig1-figsupp2-data1.zip › Figure 1-figure supplement 2E.pdf]

S2F

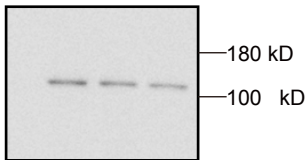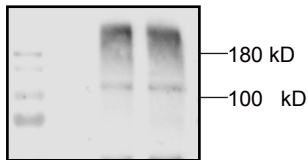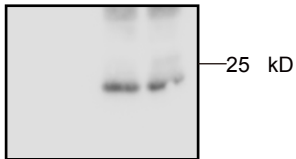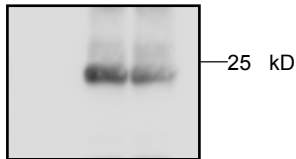

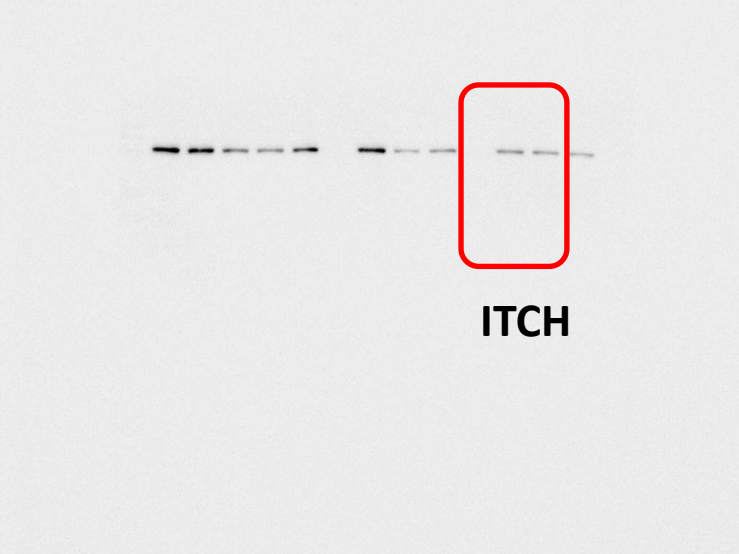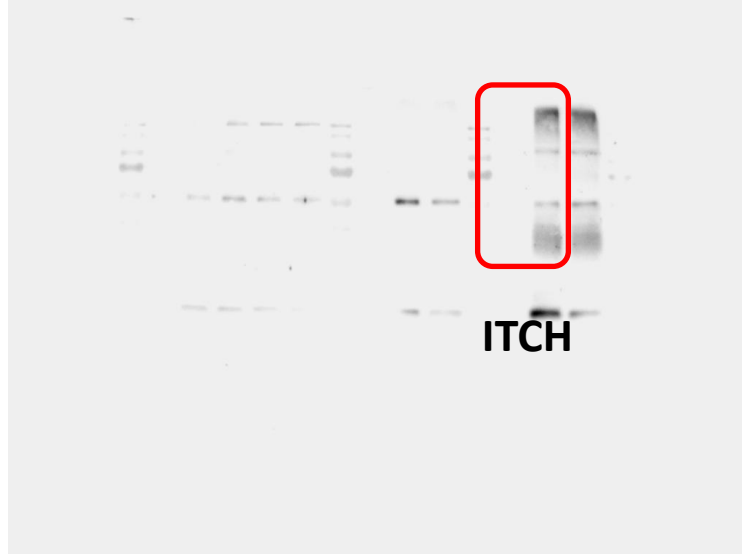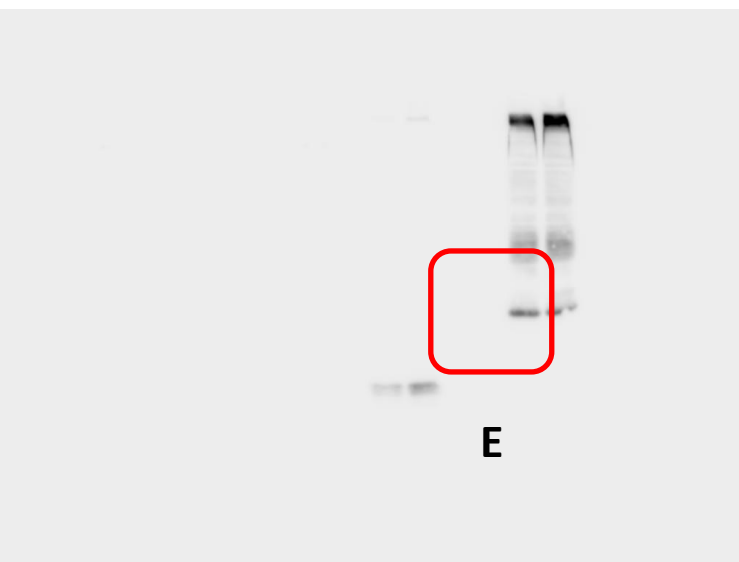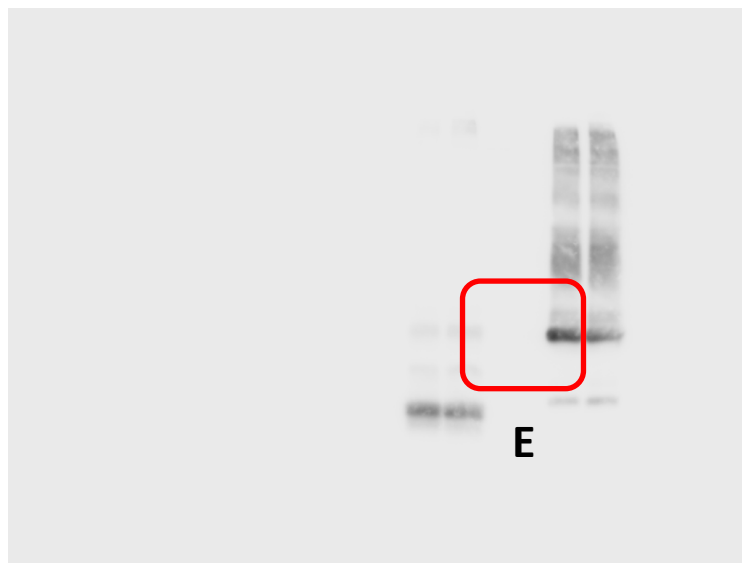

Supplement: Figure 1—figure supplement 2—source data 1. [file elife-105105-fig1-figsupp2-data1.zip › Figure 1-figure supplement 2F.pdf]

# S2H

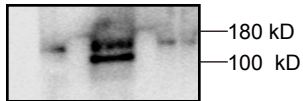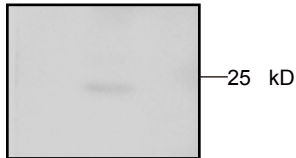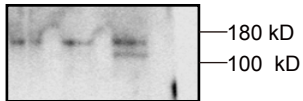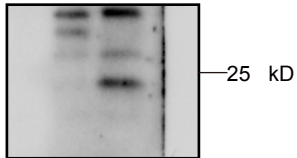

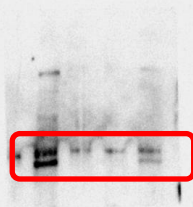

ITCH

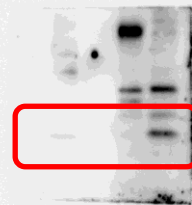

M

Supplement: Figure 1—figure supplement 2—source data 1. [file elife-105105-fig1-figsupp2-data1.zip › Figure 1-figure supplement 2H.pdf]

# S2I

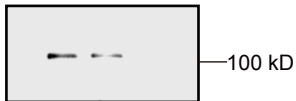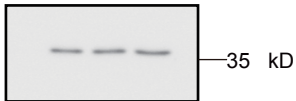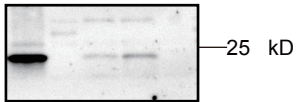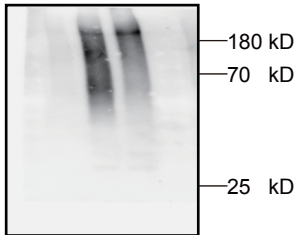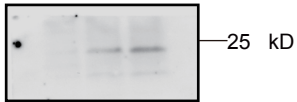

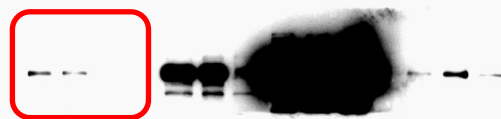

ITCH

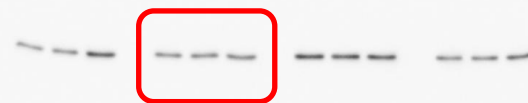

gapdh

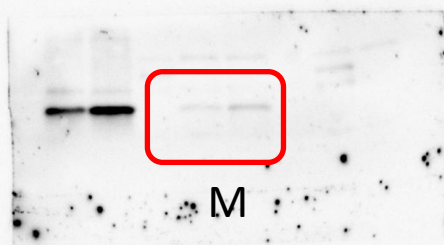

M

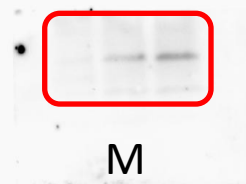

M

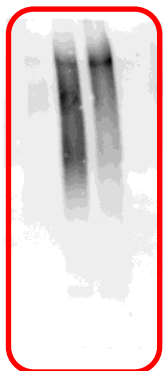

M ubi

Supplement: Figure 1—figure supplement 2—source data 1. [file elife-105105-fig1-figsupp2-data1.zip › Figure 1-figure supplement 2I.pdf]

# S2A

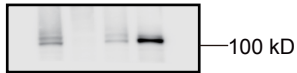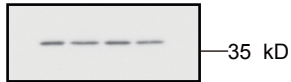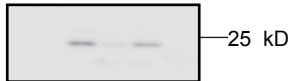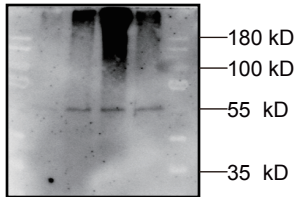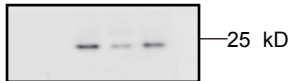

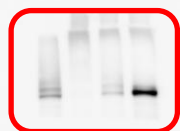

**ITCH**

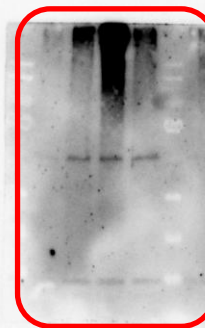

**ubi**

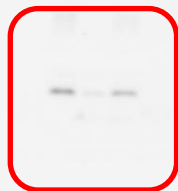

**Flag M**

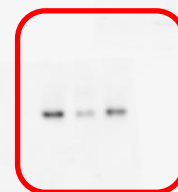

**Flag M**

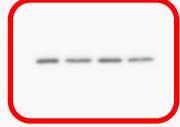

**GADPH**

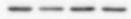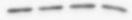

Supplement: Figure 1—figure supplement 2—source data 1. [file elife-105105-fig1-figsupp2-data1.zip › Figure 1-figure supplement 2A.pdf]

# S2B

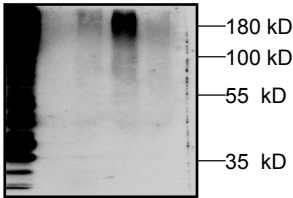

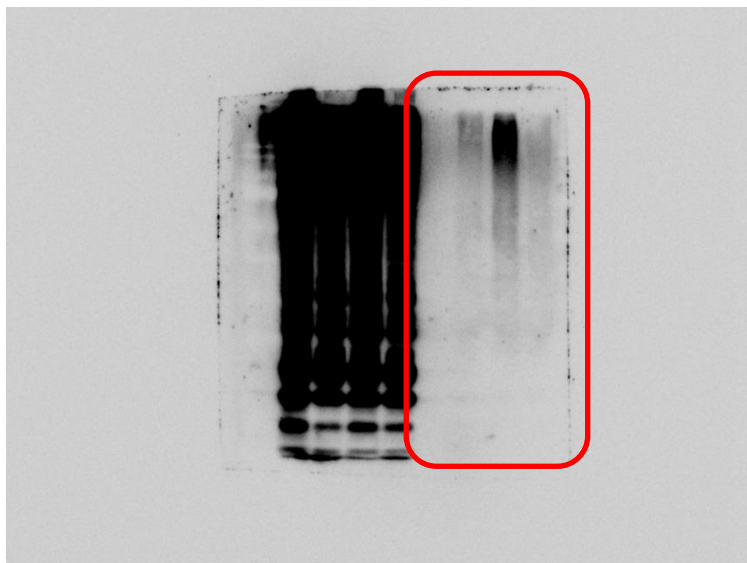

Supplement: Figure 1—figure supplement 2—source data 1. [file elife-105105-fig1-figsupp2-data1.zip › Figure 1-figure supplement 2B.pdf]

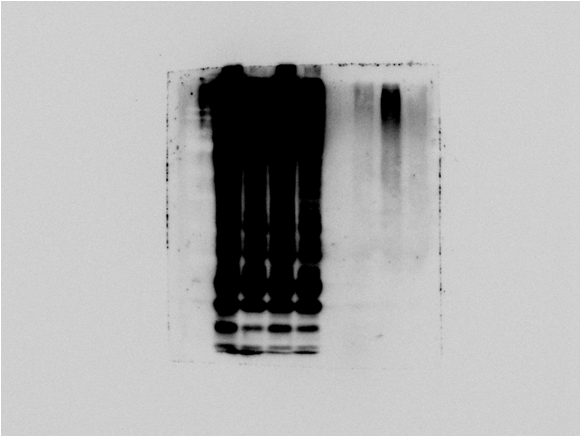

Supplement: Figure 1—figure supplement 2—source data 2. [file elife-105105-fig1-figsupp2-data2.zip › Figure 1-figure supplement 2B/k63.tif]

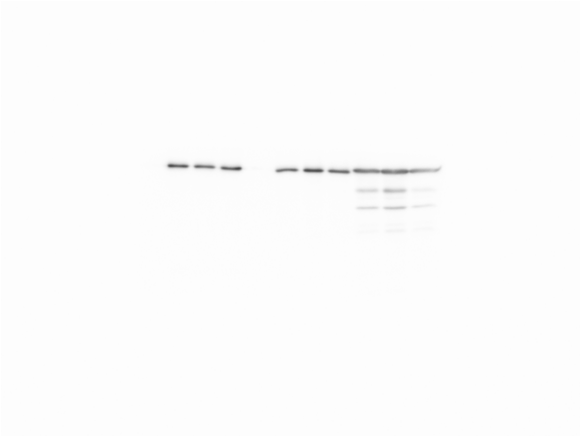

Supplement: Figure 1—figure supplement 2—source data 2. [file elife-105105-fig1-figsupp2-data2.zip › Figure 1-figure supplement 2C/gapdh.tif]

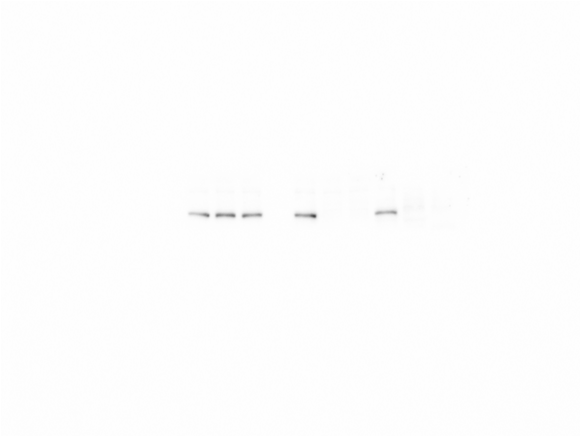

Supplement: Figure 1—figure supplement 2—source data 2. [file elife-105105-fig1-figsupp2-data2.zip › Figure 1-figure supplement 2C/ITCH S2C.tif]

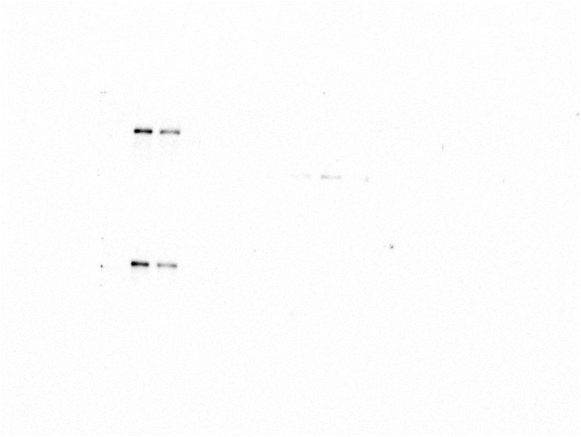

Supplement: Figure 1—figure supplement 2—source data 2. [file elife-105105-fig1-figsupp2-data2.zip › Figure 1-figure supplement 2D/ITCH.tif]

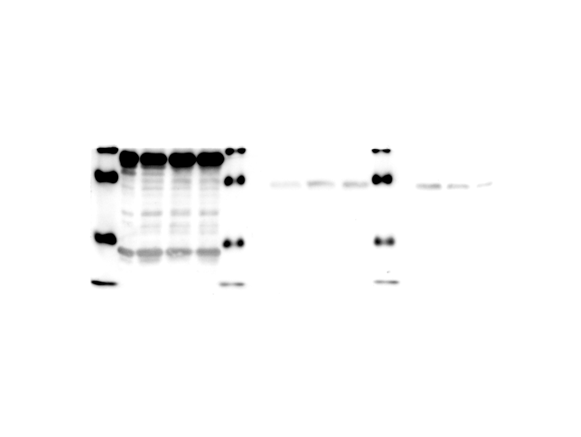

Supplement: Figure 1—figure supplement 2—source data 2. [file elife-105105-fig1-figsupp2-data2.zip › Figure 1-figure supplement 2D/M DIP.tif]

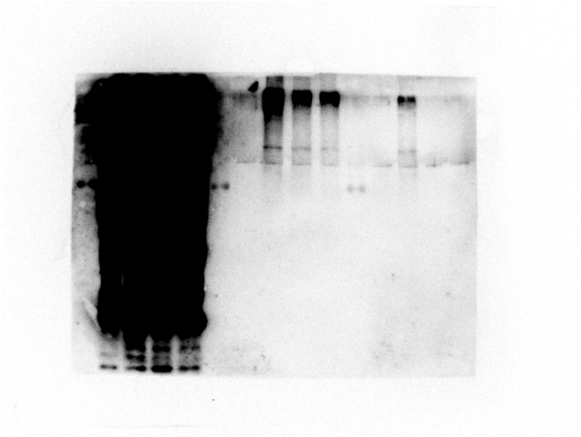

Supplement: Figure 1—figure supplement 2—source data 2. [file elife-105105-fig1-figsupp2-data2.zip › Figure 1-figure supplement 2D/UBI.tif]

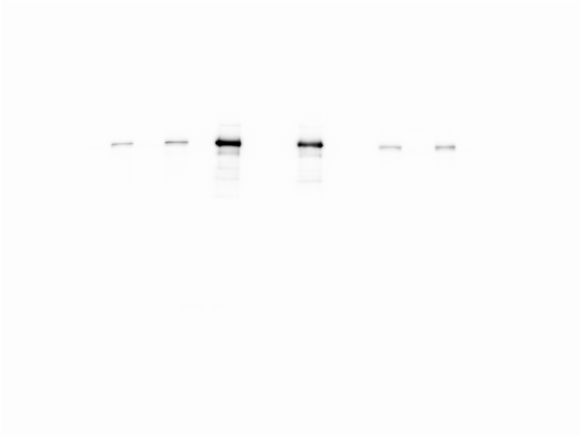

Supplement: Figure 1—figure supplement 2—source data 2. [file elife-105105-fig1-figsupp2-data2.zip › Figure 1-figure supplement 2E/ITCH.tif]

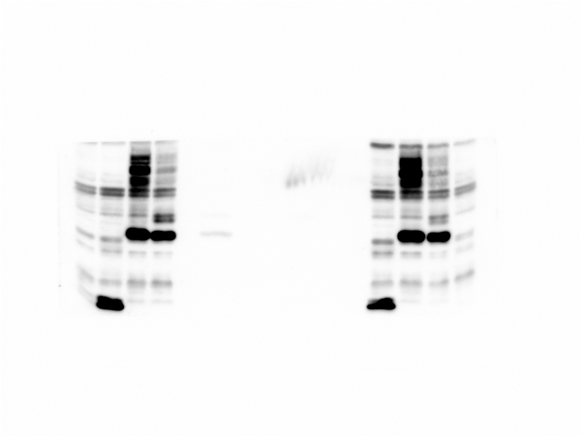

Supplement: Figure 1—figure supplement 2—source data 2. [file elife-105105-fig1-figsupp2-data2.zip › Figure 1-figure supplement 2E/M.tif]

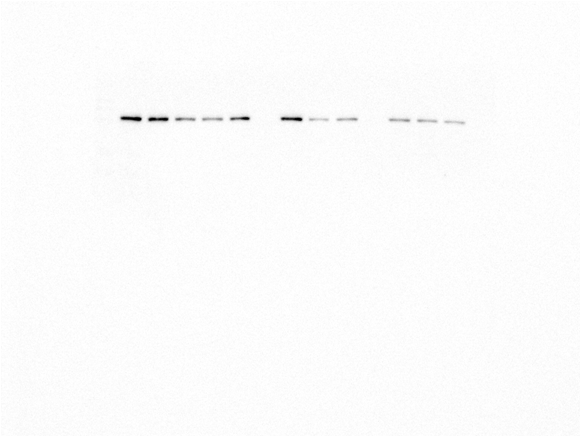

Supplement: Figure 1—figure supplement 2—source data 2. [file elife-105105-fig1-figsupp2-data2.zip › Figure 1-figure supplement 2F/ITCH input.tif]

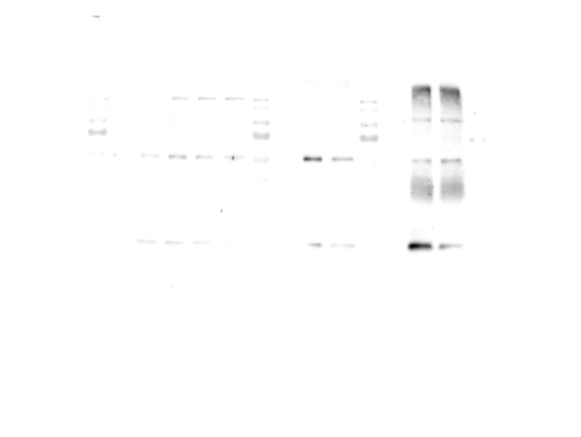

Supplement: Figure 1—figure supplement 2—source data 2. [file elife-105105-fig1-figsupp2-data2.zip › Figure 1-figure supplement 2F/itch ip.tif]

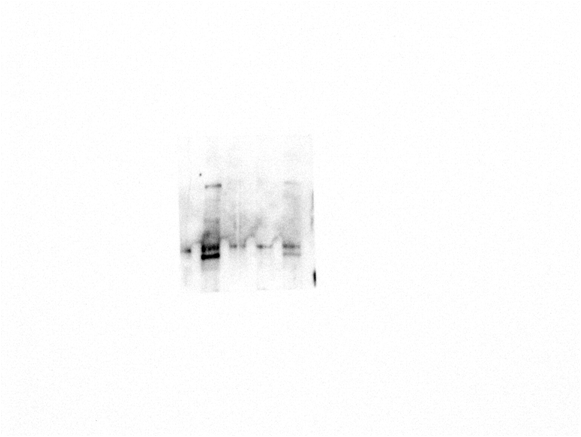

Supplement: Figure 1—figure supplement 2—source data 2. [file elife-105105-fig1-figsupp2-data2.zip › Figure 1-figure supplement 2H/itch.tif]

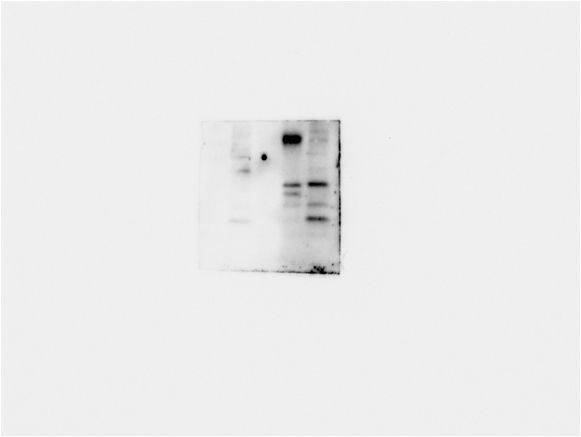

Supplement: Figure 1—figure supplement 2—source data 2. [file elife-105105-fig1-figsupp2-data2.zip › Figure 1-figure supplement 2H/M.tif]

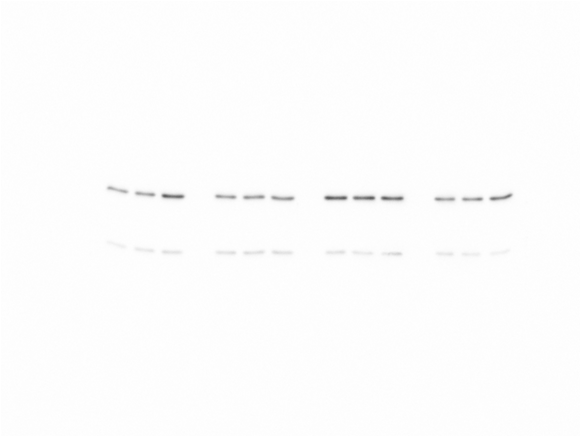

Supplement: Figure 1—figure supplement 2—source data 2. [file elife-105105-fig1-figsupp2-data2.zip › Figure 1-figure supplement 2I/gapdh.tif]

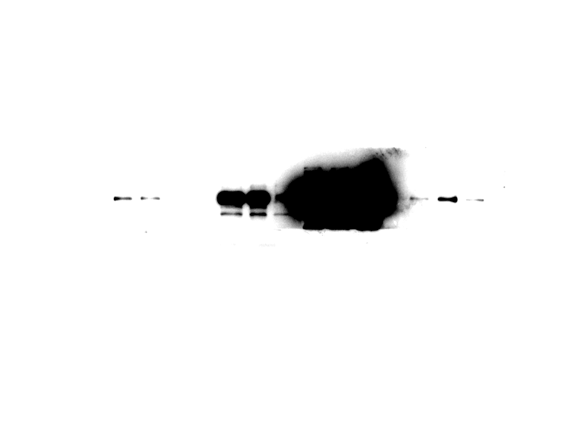

Supplement: Figure 1—figure supplement 2—source data 2. [file elife-105105-fig1-figsupp2-data2.zip › Figure 1-figure supplement 2I/itch.tif]

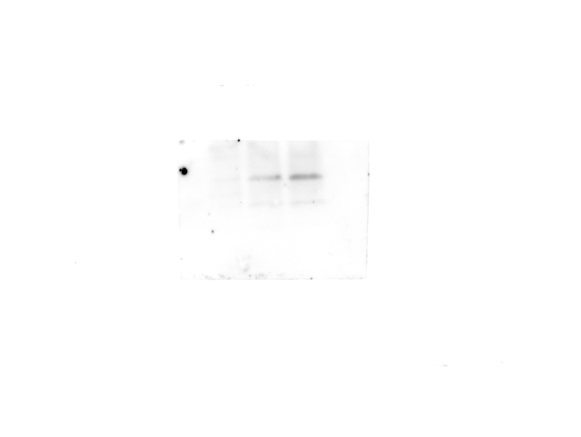

Supplement: Figure 1—figure supplement 2—source data 2. [file elife-105105-fig1-figsupp2-data2.zip › Figure 1-figure supplement 2I/m dip.tif]

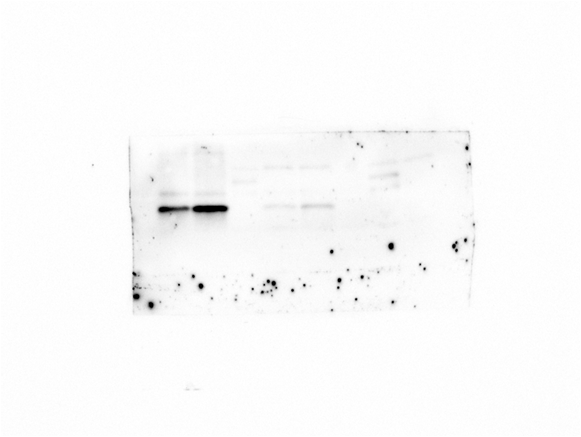

Supplement: Figure 1—figure supplement 2—source data 2. [file elife-105105-fig1-figsupp2-data2.zip › Figure 1-figure supplement 2I/m input.tif]

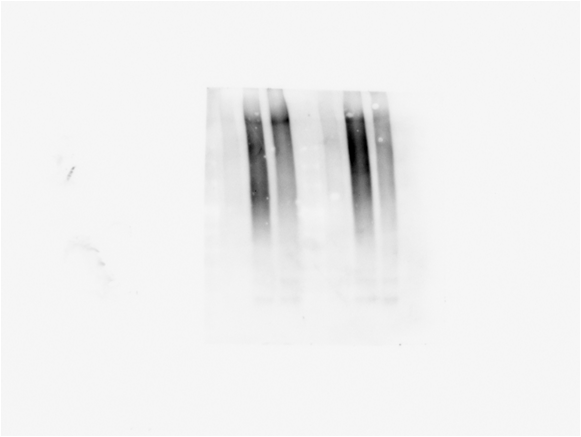

Supplement: Figure 1—figure supplement 2—source data 2. [file elife-105105-fig1-figsupp2-data2.zip › Figure 1-figure supplement 2I/m ubi.tif]

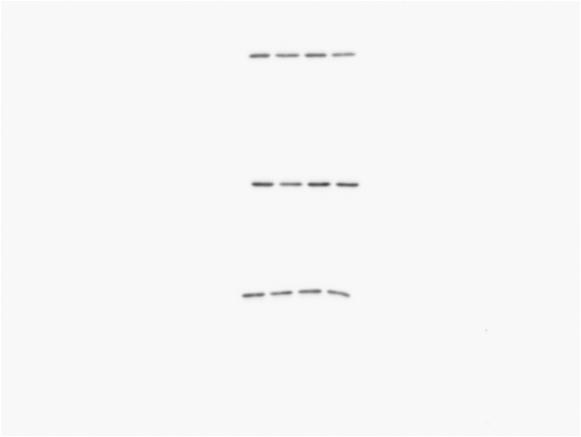

Supplement: Figure 1—figure supplement 2—source data 2. [file elife-105105-fig1-figsupp2-data2.zip › Figure 1-figure supplement 2A/gapdh.tif]

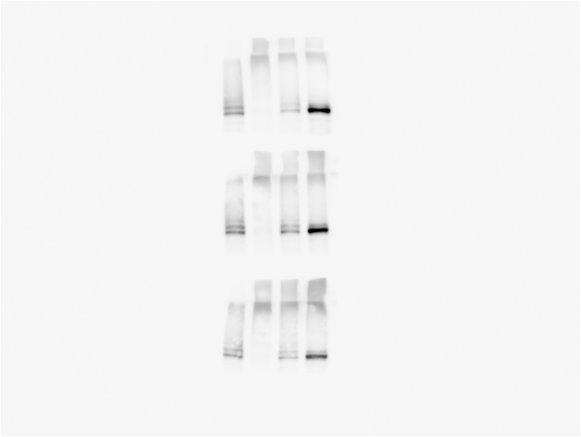

Supplement: Figure 1—figure supplement 2—source data 2. [file elife-105105-fig1-figsupp2-data2.zip › Figure 1-figure supplement 2A/itch.tif]

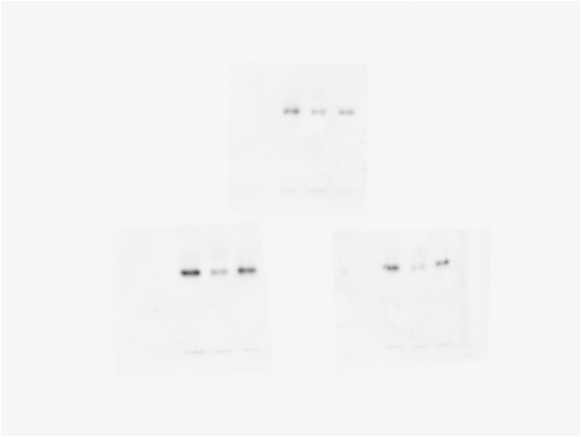

Supplement: Figure 1—figure supplement 2—source data 2. [file elife-105105-fig1-figsupp2-data2.zip › Figure 1-figure supplement 2A/m dip.tif]
