# Supplementary figures and images for: Ubiquitin ligase ITCH regulates life cycle of SARS-CoV-2 virus (part 2 of 4)
Source: eLife. 2026 May 29;14:RP105105. doi: 10.7554/eLife.105105 (PMC13221179; doi:10.7554/eLife.105105)

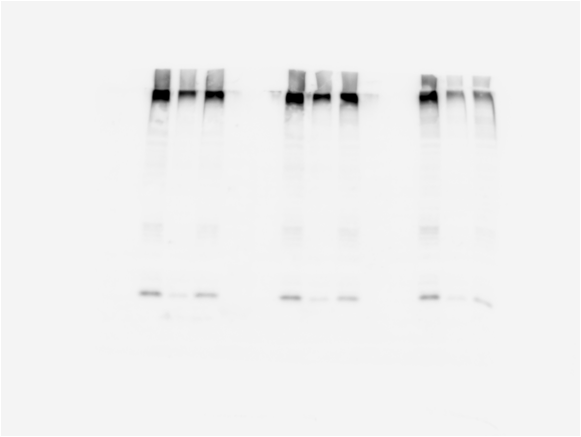

Supplement: Figure 1—figure supplement 2—source data 2. [file elife-105105-fig1-figsupp2-data2.zip › Figure 1-figure supplement 2A/m.tif]

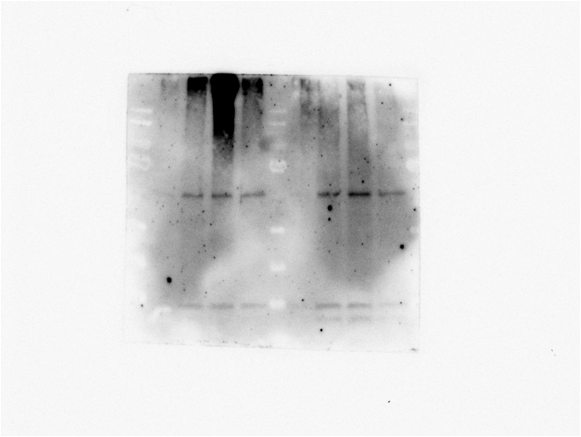

Supplement: Figure 1—figure supplement 2—source data 2. [file elife-105105-fig1-figsupp2-data2.zip › Figure 1-figure supplement 2A/ubi.tif]

2B

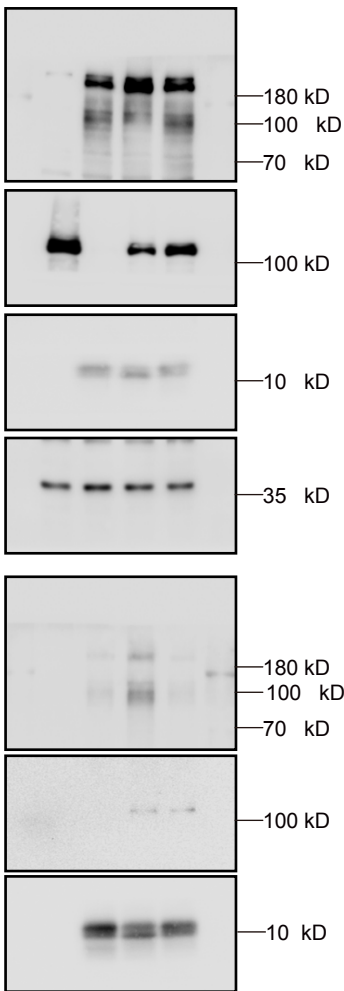

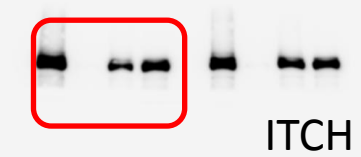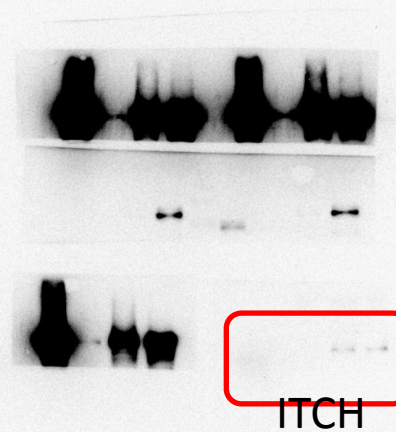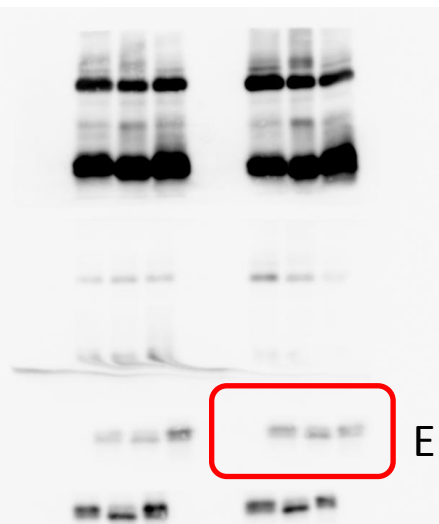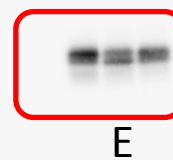

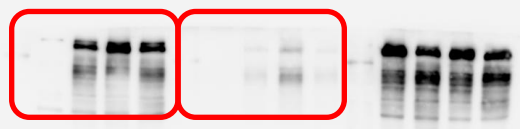

spike

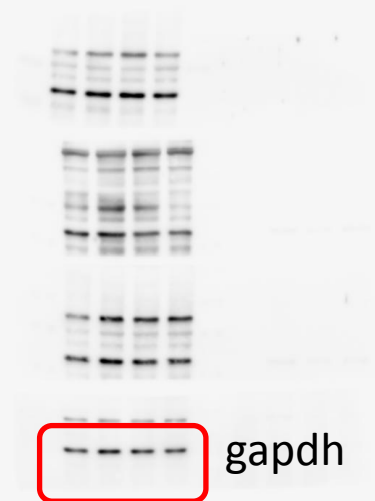

Supplement: Figure 2—source data 1. [file elife-105105-fig2-data1.zip › Figure 2B.pdf]

2C

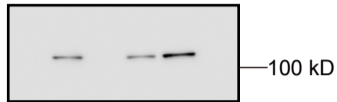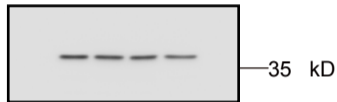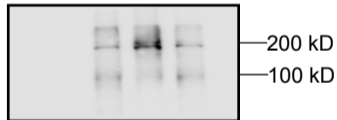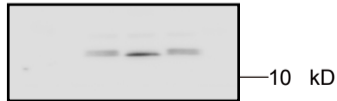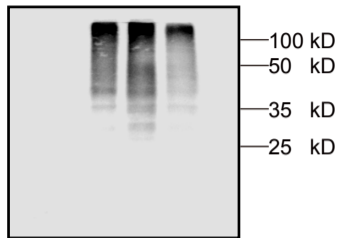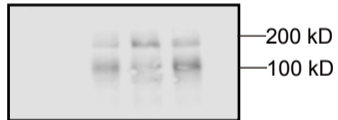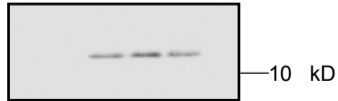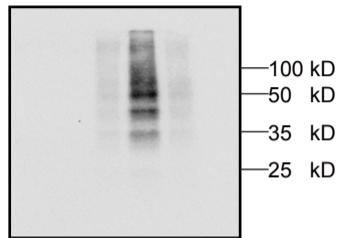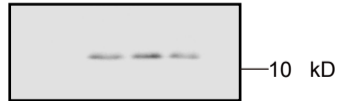

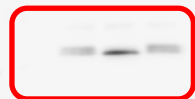

E

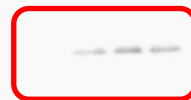

E 1IP

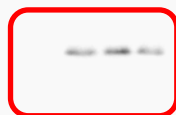

E 2IP

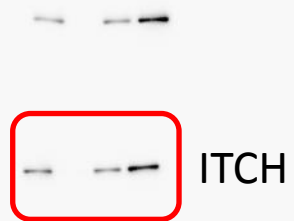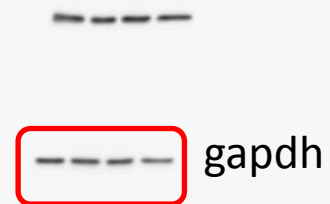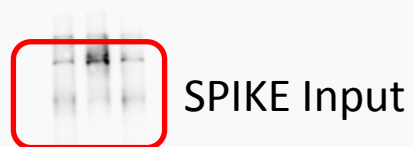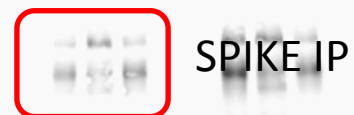

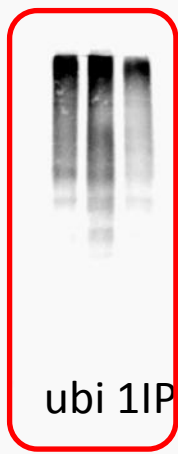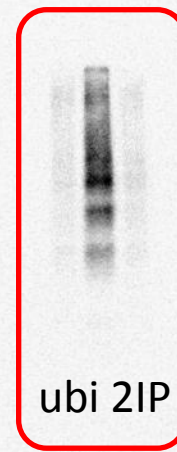

Supplement: Figure 2—source data 1. [file elife-105105-fig2-data1.zip › Figure 2C.pdf]

2D

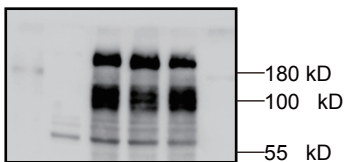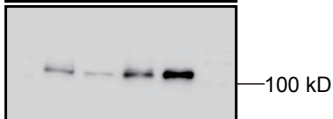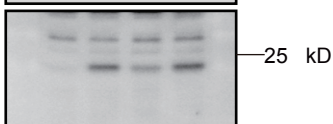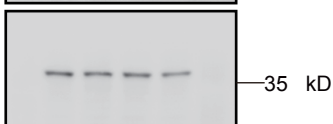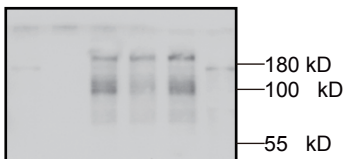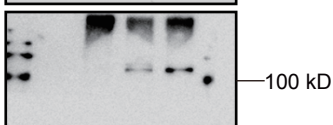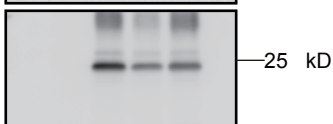

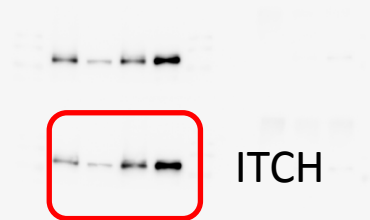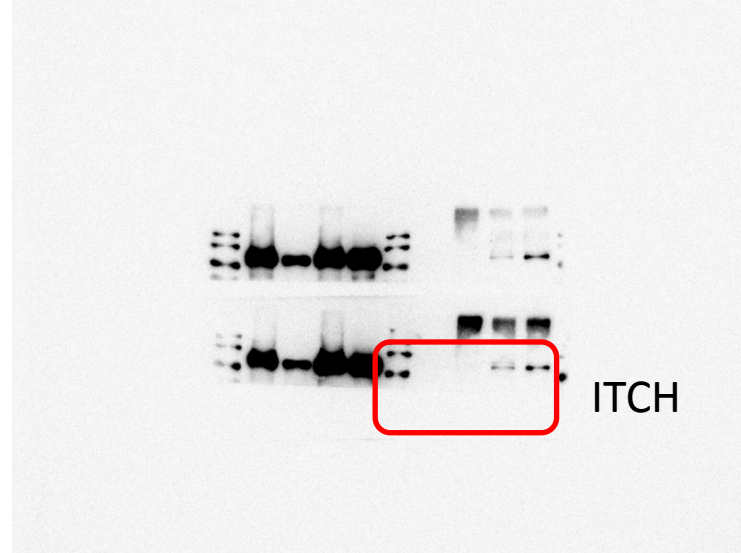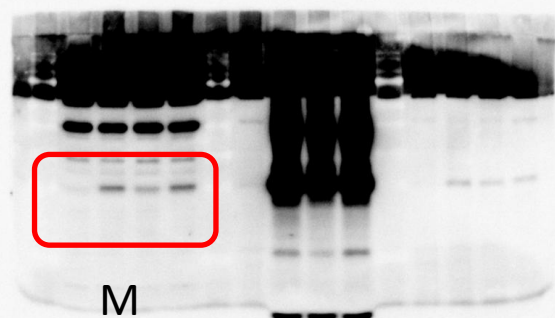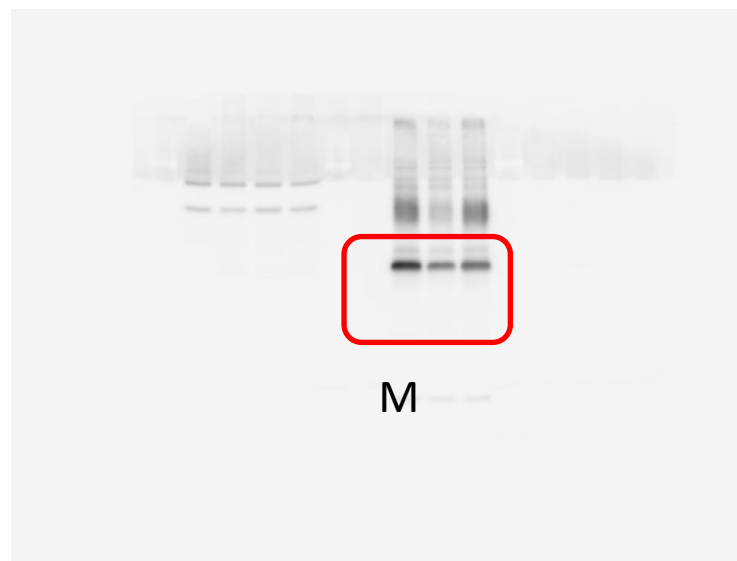

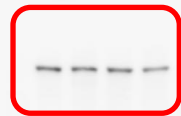

gapdh

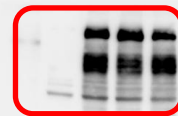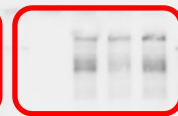

spike

Supplement: Figure 2—source data 1. [file elife-105105-fig2-data1.zip › Figure 2D.pdf]

2E

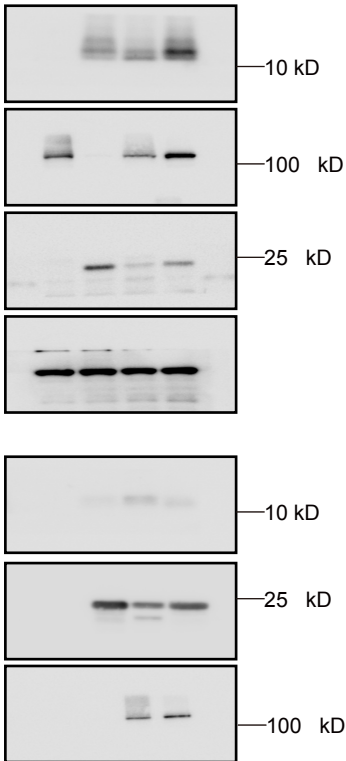

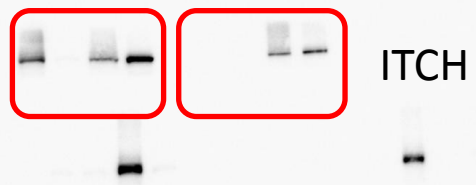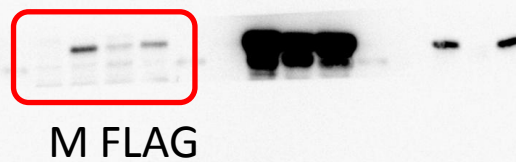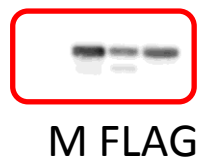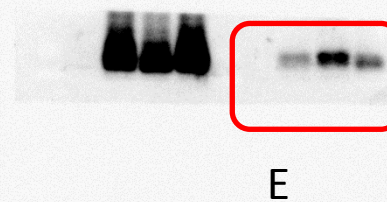

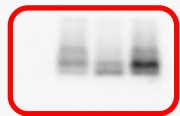

E

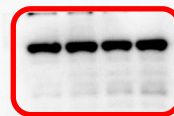

gapdh

Supplement: Figure 2—source data 1. [file elife-105105-fig2-data1.zip › Figure 2E.pdf]

2F

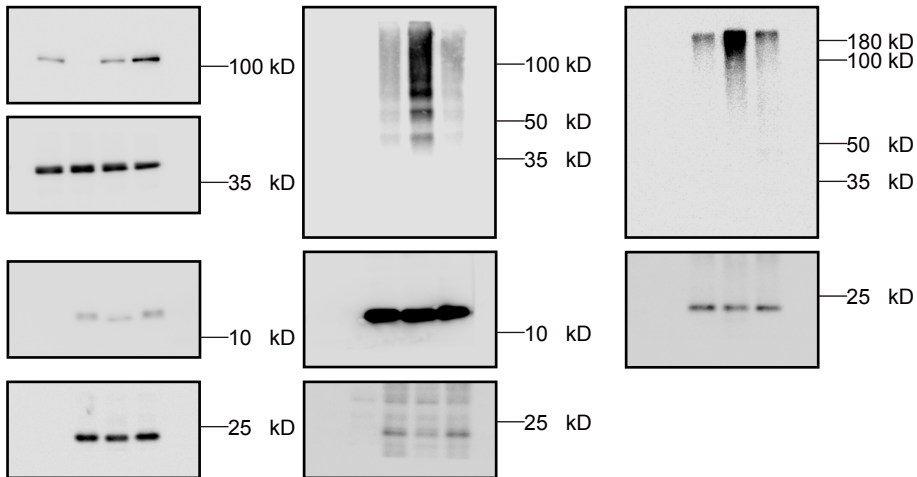

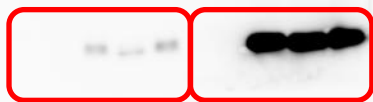

E

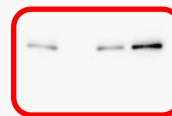

ITCH

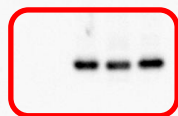

M input

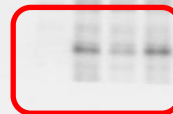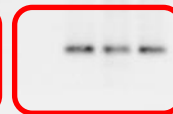

M 1 2 IP

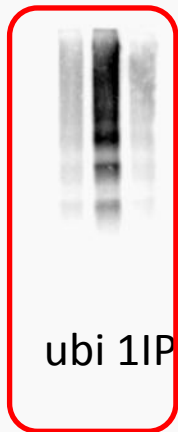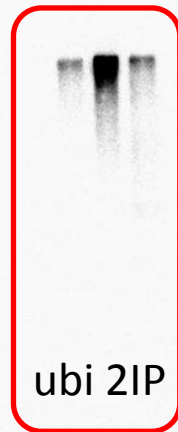

Supplement: Figure 2—source data 1. [file elife-105105-fig2-data1.zip › Figure 2F.pdf]

Fig. 2A

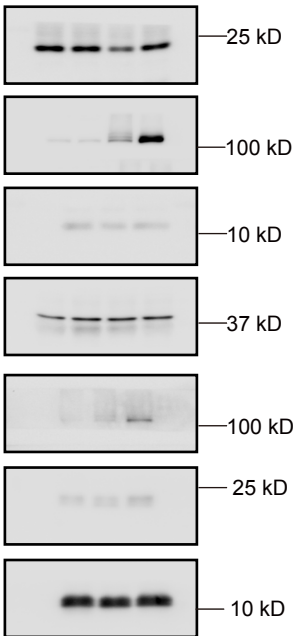

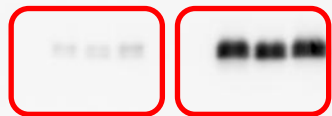

E

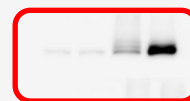

ITCH input

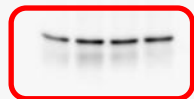

GAPDH

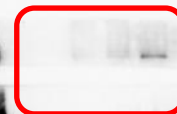

ITCH ip

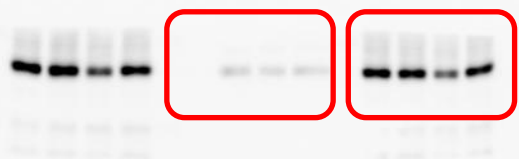

M

Supplement: Figure 2—source data 1. [file elife-105105-fig2-data1.zip › Figure 2A.pdf]

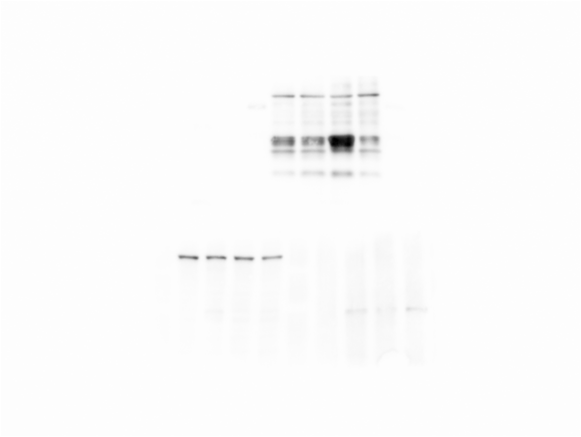

Supplement: Figure 2—source data 2. [file elife-105105-fig2-data2.zip › Figure 2D/gapdh.tif]

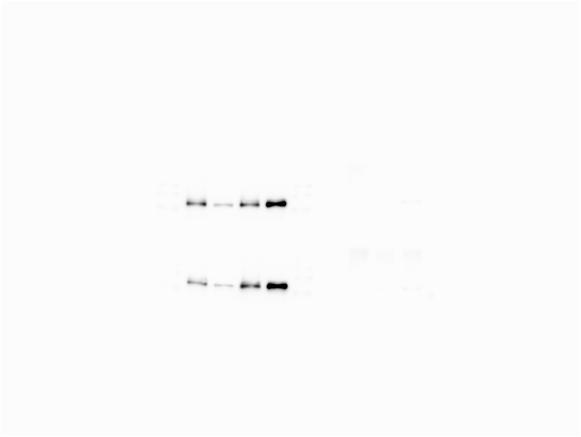

Supplement: Figure 2—source data 2. [file elife-105105-fig2-data2.zip › Figure 2D/itch input.tif]

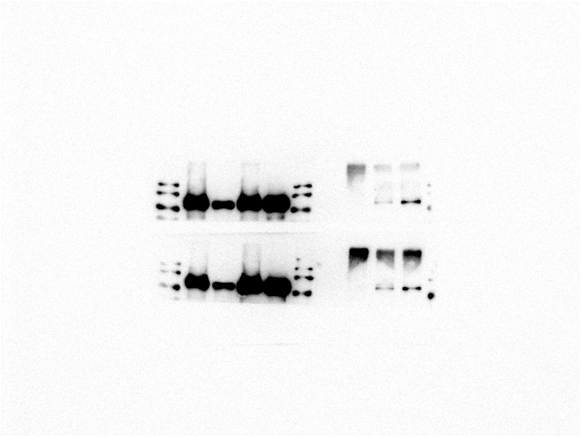

Supplement: Figure 2—source data 2. [file elife-105105-fig2-data2.zip › Figure 2D/itch ip.tif]

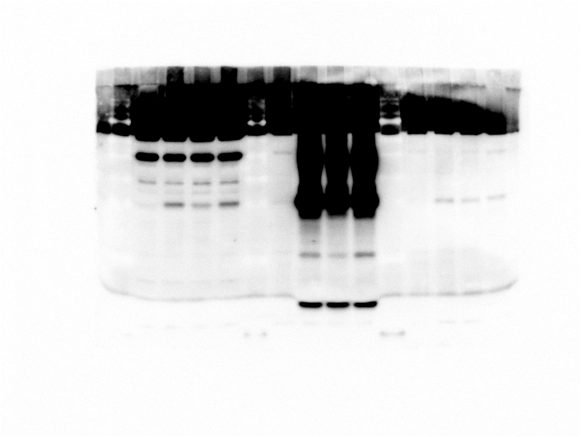

Supplement: Figure 2—source data 2. [file elife-105105-fig2-data2.zip › Figure 2D/m input.tif]

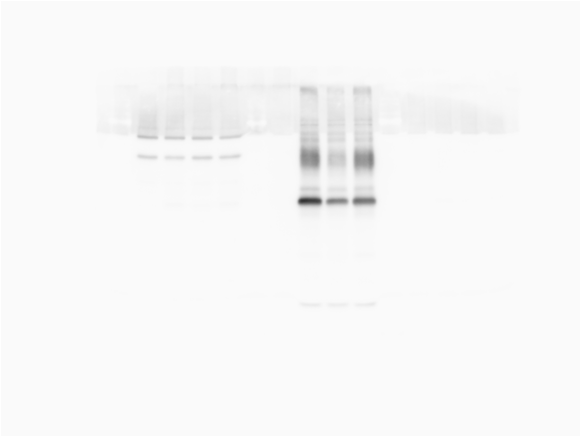

Supplement: Figure 2—source data 2. [file elife-105105-fig2-data2.zip › Figure 2D/m ip.tif]

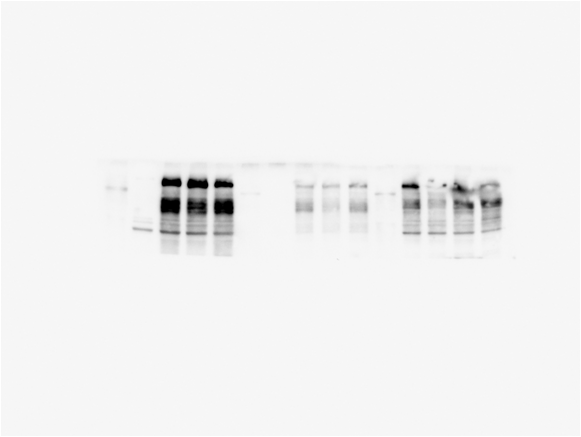

Supplement: Figure 2—source data 2. [file elife-105105-fig2-data2.zip › Figure 2D/spike.tif]

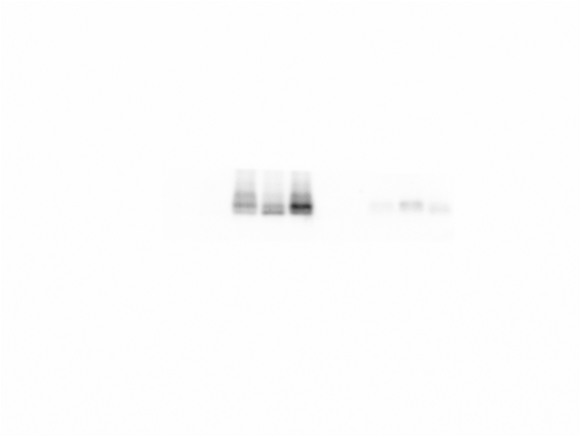

Supplement: Figure 2—source data 2. [file elife-105105-fig2-data2.zip › Figure 2E/E input.tif]

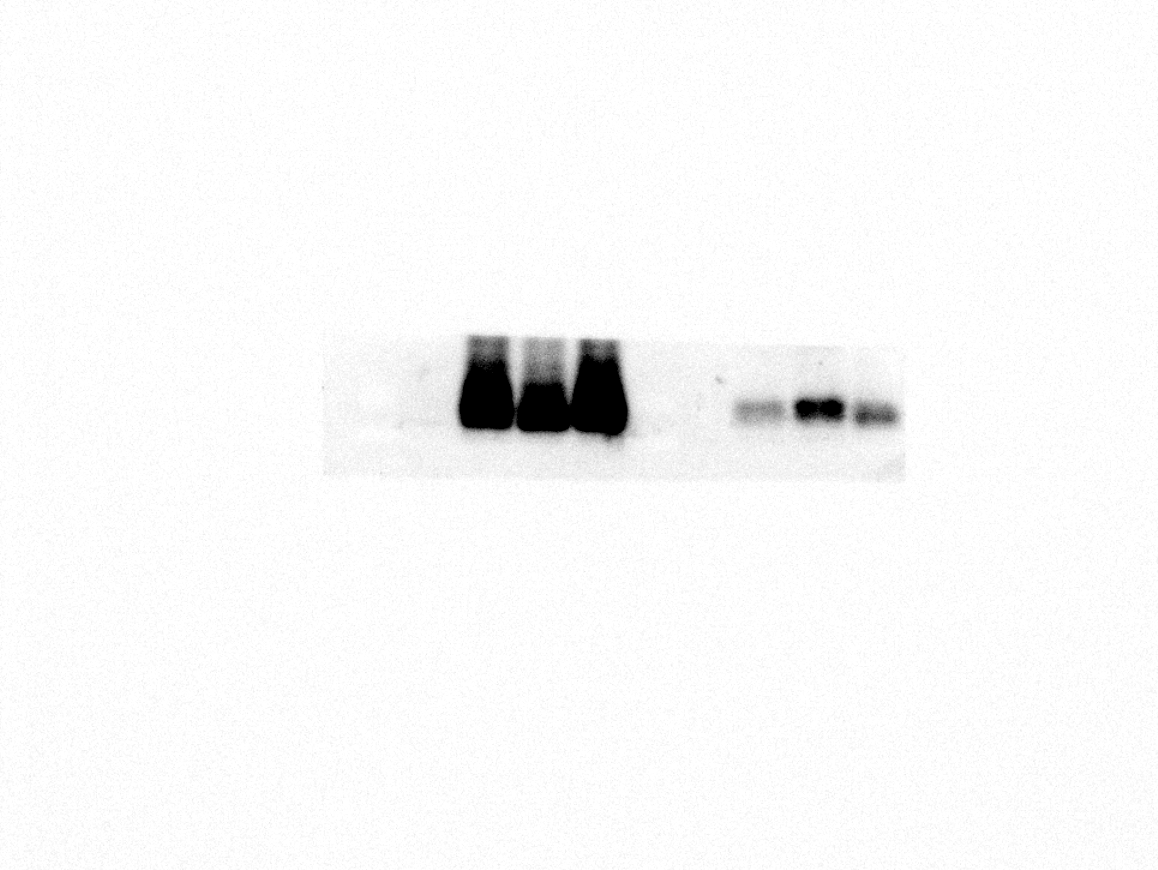

Supplement: Figure 2—source data 2. [file elife-105105-fig2-data2.zip › Figure 2E/E.tif]

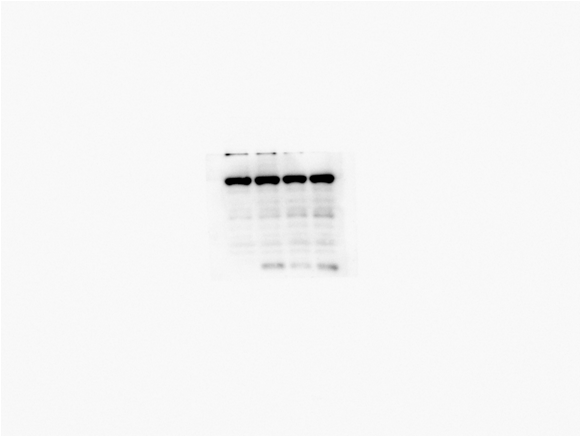

Supplement: Figure 2—source data 2. [file elife-105105-fig2-data2.zip › Figure 2E/gapdh.tif]

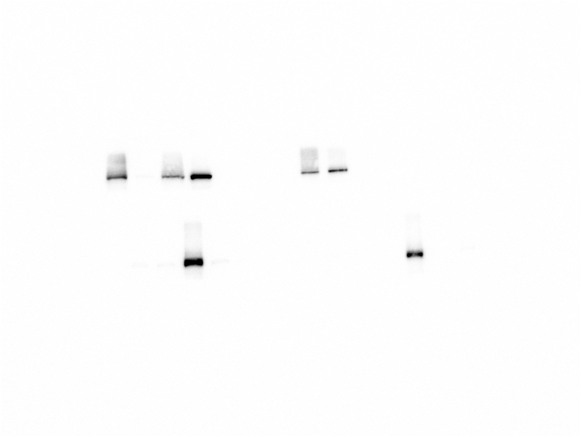

Supplement: Figure 2—source data 2. [file elife-105105-fig2-data2.zip › Figure 2E/ITCH.tif]

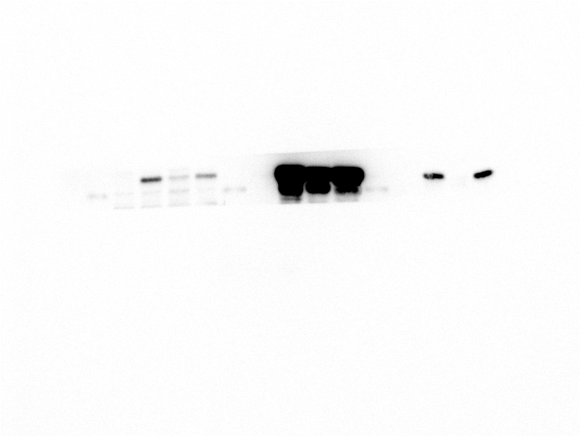

Supplement: Figure 2—source data 2. [file elife-105105-fig2-data2.zip › Figure 2E/M INPUT.tif]

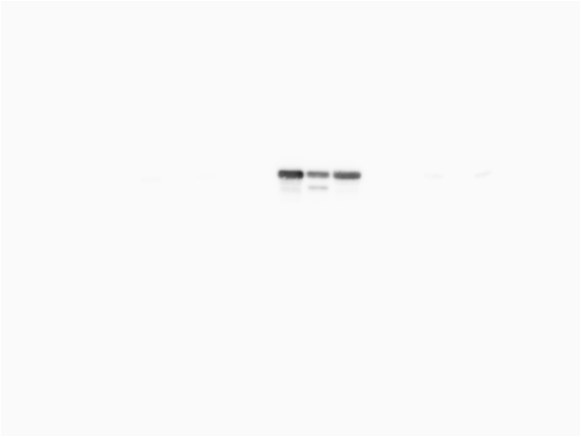

Supplement: Figure 2—source data 2. [file elife-105105-fig2-data2.zip › Figure 2E/M IP.tif]

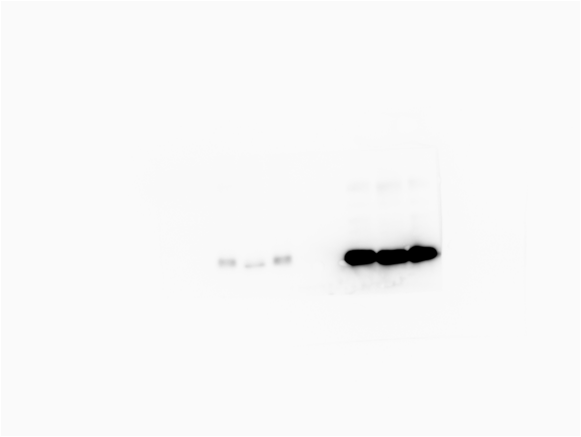

Supplement: Figure 2—source data 2. [file elife-105105-fig2-data2.zip › Figure 2F/FLAG E.tif]

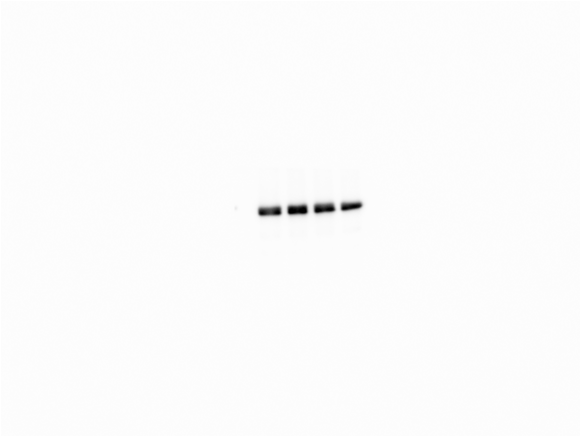

Supplement: Figure 2—source data 2. [file elife-105105-fig2-data2.zip › Figure 2F/GAPDH.tif]

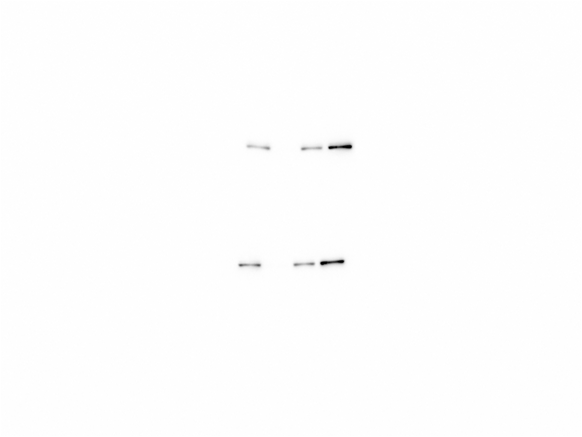

Supplement: Figure 2—source data 2. [file elife-105105-fig2-data2.zip › Figure 2F/ITCH.tif]

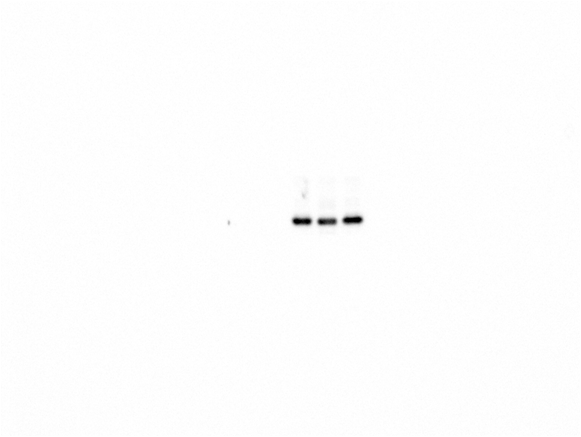

Supplement: Figure 2—source data 2. [file elife-105105-fig2-data2.zip › Figure 2F/m input.tif]

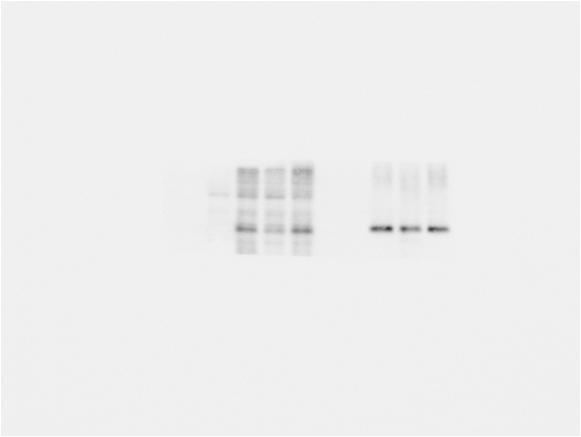

Supplement: Figure 2—source data 2. [file elife-105105-fig2-data2.zip › Figure 2F/S tag M 2.tif]

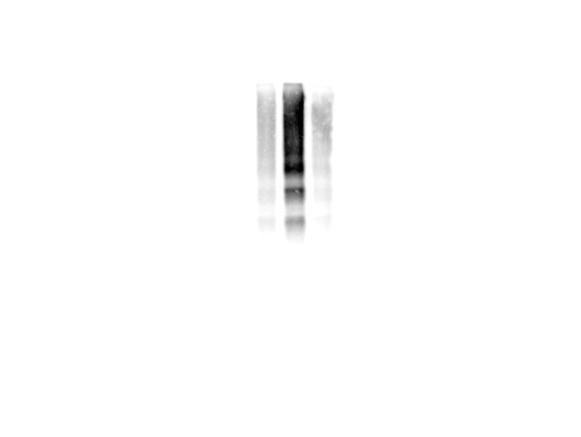

Supplement: Figure 2—source data 2. [file elife-105105-fig2-data2.zip › Figure 2F/ubi E.tif]

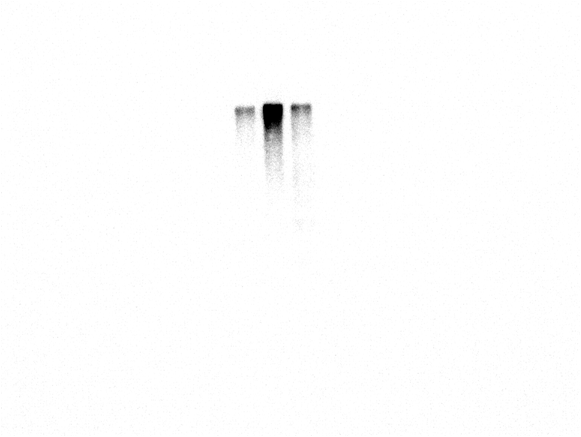

Supplement: Figure 2—source data 2. [file elife-105105-fig2-data2.zip › Figure 2F/ubi M.tif]

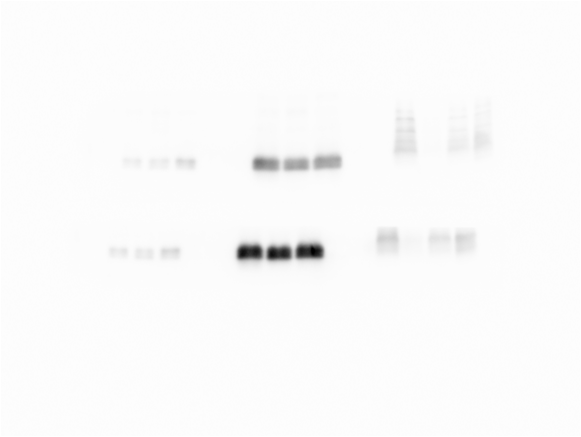

Supplement: Figure 2—source data 2. [file elife-105105-fig2-data2.zip › Figure 2A/Flag E.tif]

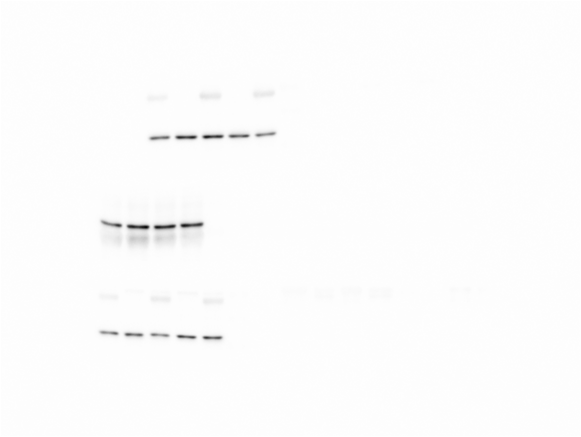

Supplement: Figure 2—source data 2. [file elife-105105-fig2-data2.zip › Figure 2A/GAPDH.tif]

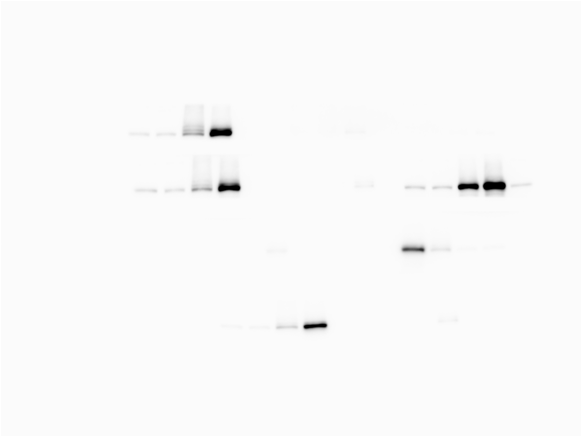

Supplement: Figure 2—source data 2. [file elife-105105-fig2-data2.zip › Figure 2A/ITCH-1.tif]

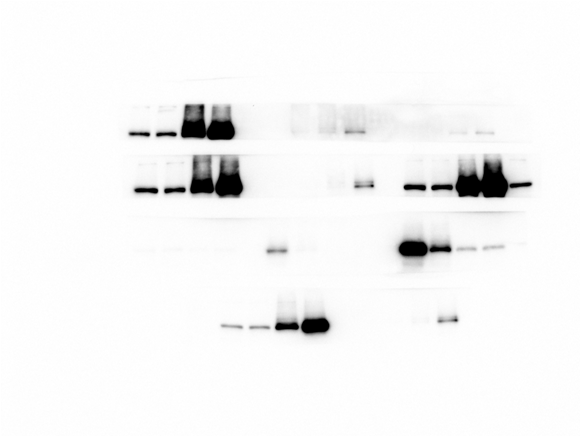

Supplement: Figure 2—source data 2. [file elife-105105-fig2-data2.zip › Figure 2A/ITCH-2.tif]

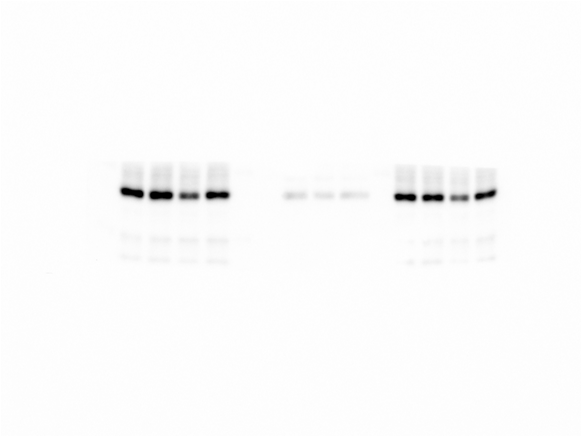

Supplement: Figure 2—source data 2. [file elife-105105-fig2-data2.zip › Figure 2A/S tag M.tif]

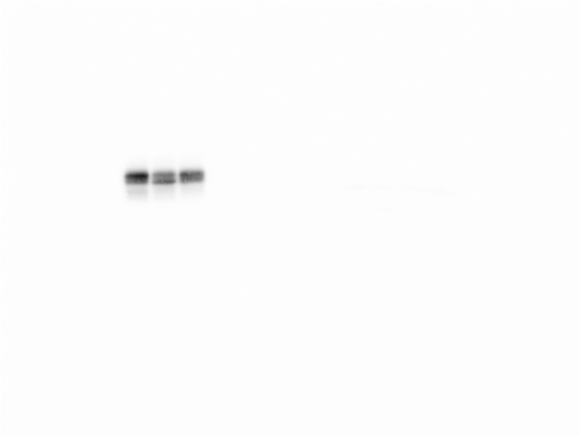

Supplement: Figure 2—source data 2. [file elife-105105-fig2-data2.zip › Figure 2B/e ip.tif]

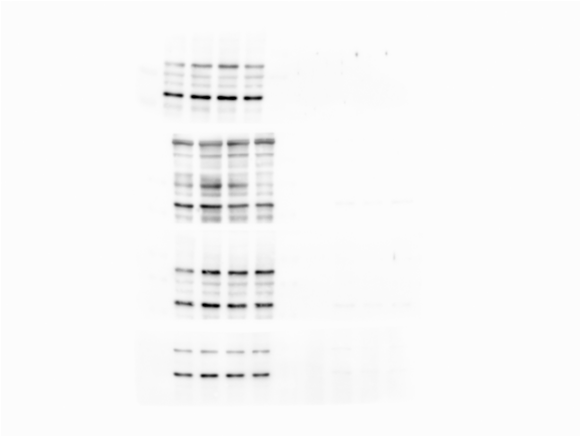

Supplement: Figure 2—source data 2. [file elife-105105-fig2-data2.zip › Figure 2B/gapdh.tif]

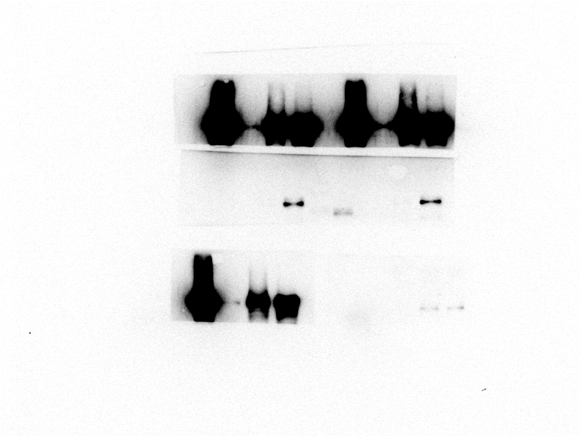

Supplement: Figure 2—source data 2. [file elife-105105-fig2-data2.zip › Figure 2B/itch ip.tif]

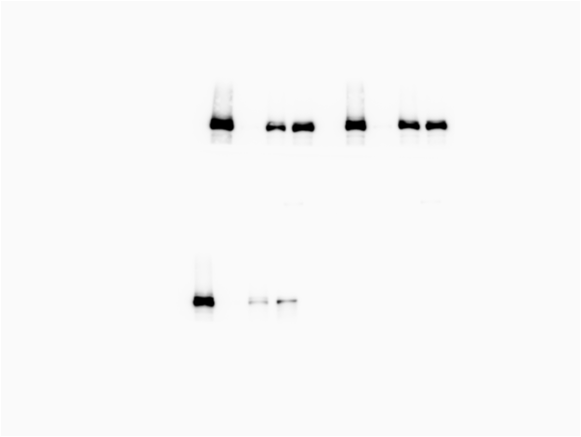

Supplement: Figure 2—source data 2. [file elife-105105-fig2-data2.zip › Figure 2B/itch.tif]

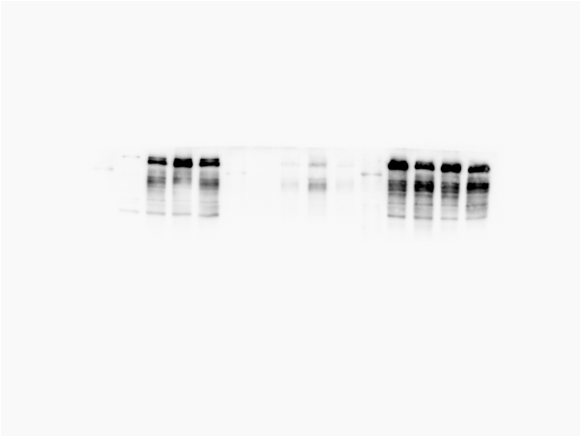

Supplement: Figure 2—source data 2. [file elife-105105-fig2-data2.zip › Figure 2B/spike.tif]

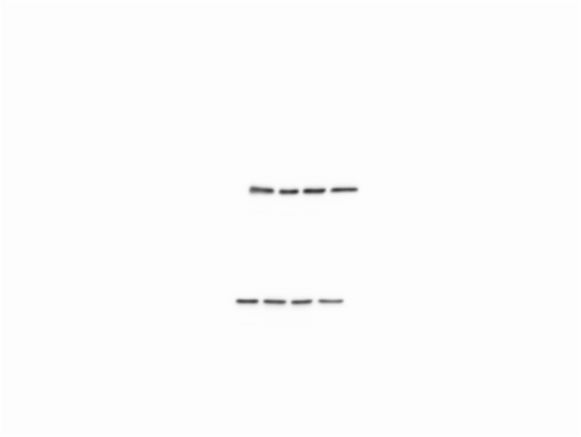

Supplement: Figure 2—source data 2. [file elife-105105-fig2-data2.zip › Figure 2C/GAPDH.tif]

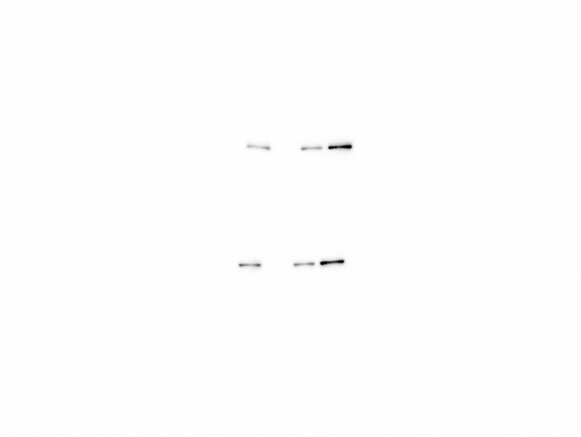

Supplement: Figure 2—source data 2. [file elife-105105-fig2-data2.zip › Figure 2C/ITCH.tif]

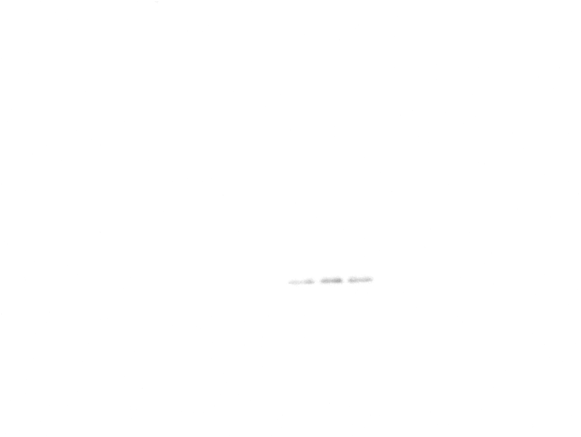

Supplement: Figure 2—source data 2. [file elife-105105-fig2-data2.zip › Figure 2C/S tag E 2.tif]

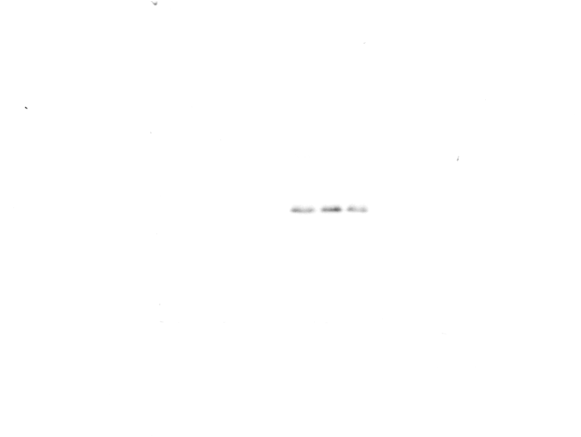

Supplement: Figure 2—source data 2. [file elife-105105-fig2-data2.zip › Figure 2C/S tag E 3.tif]

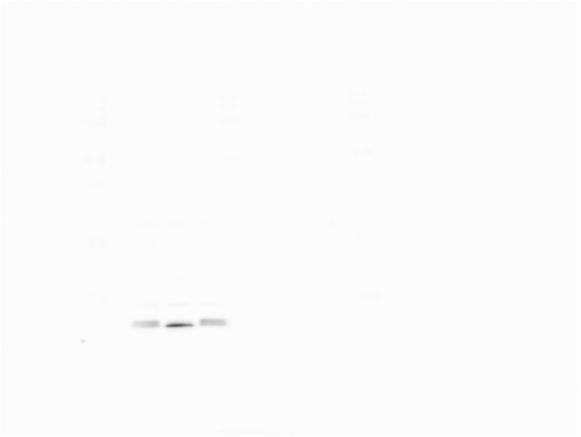

Supplement: Figure 2—source data 2. [file elife-105105-fig2-data2.zip › Figure 2C/S tag E.tif]

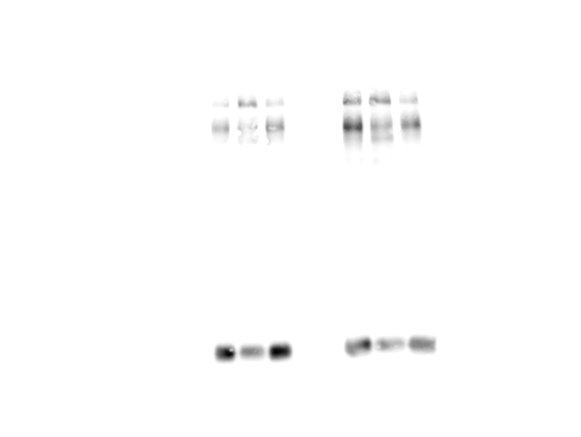

Supplement: Figure 2—source data 2. [file elife-105105-fig2-data2.zip › Figure 2C/SPIKE 2.tif]

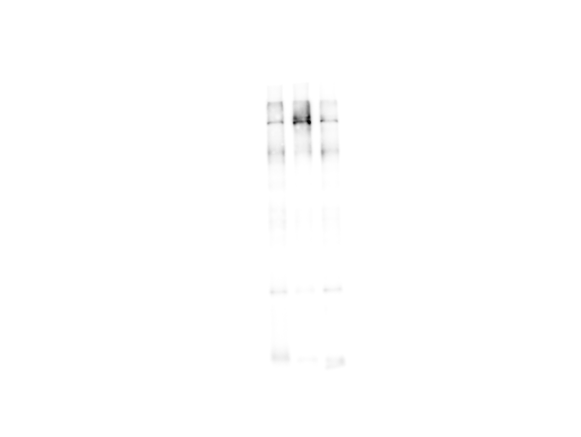

Supplement: Figure 2—source data 2. [file elife-105105-fig2-data2.zip › Figure 2C/SPIKE.tif]

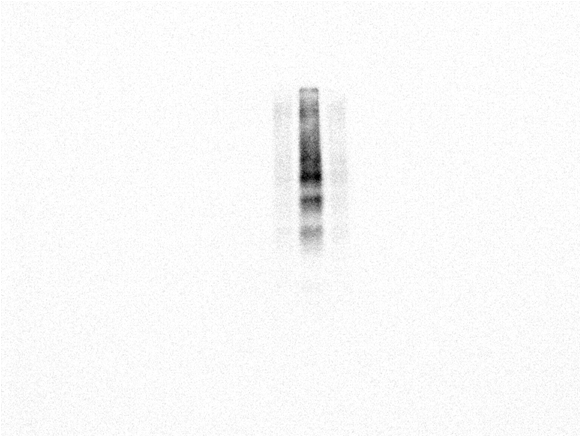

Supplement: Figure 2—source data 2. [file elife-105105-fig2-data2.zip › Figure 2C/ubi E.tif]

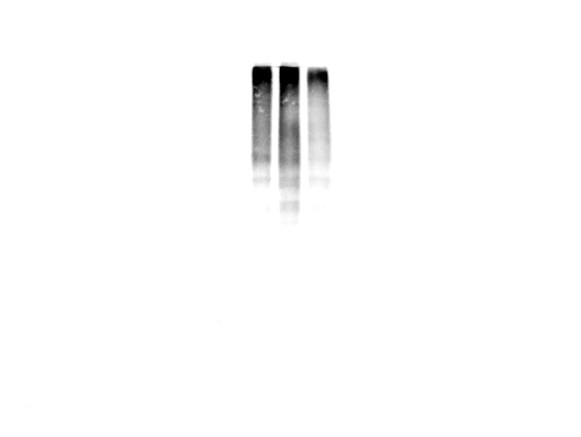

Supplement: Figure 2—source data 2. [file elife-105105-fig2-data2.zip › Figure 2C/ubi S.tif]

3H

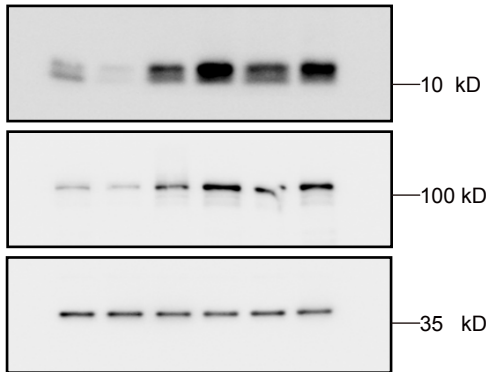

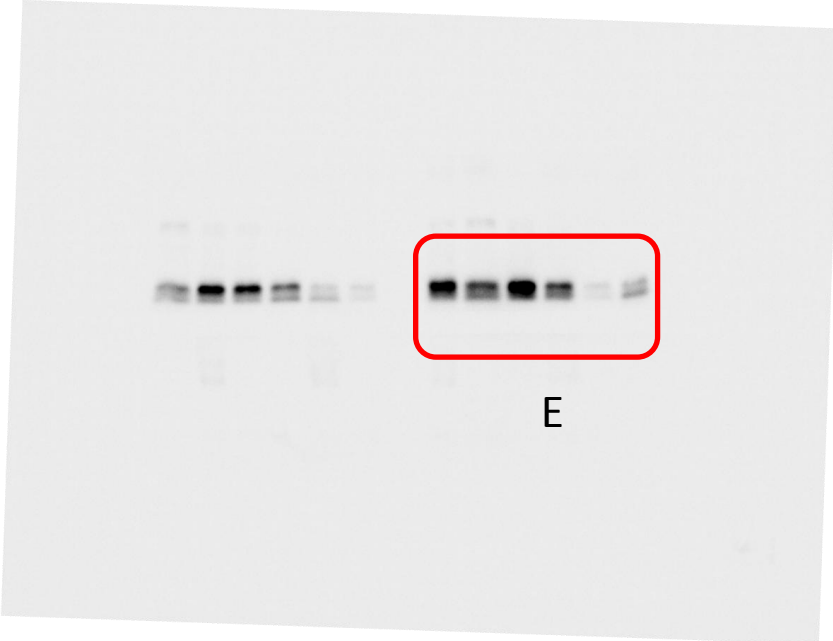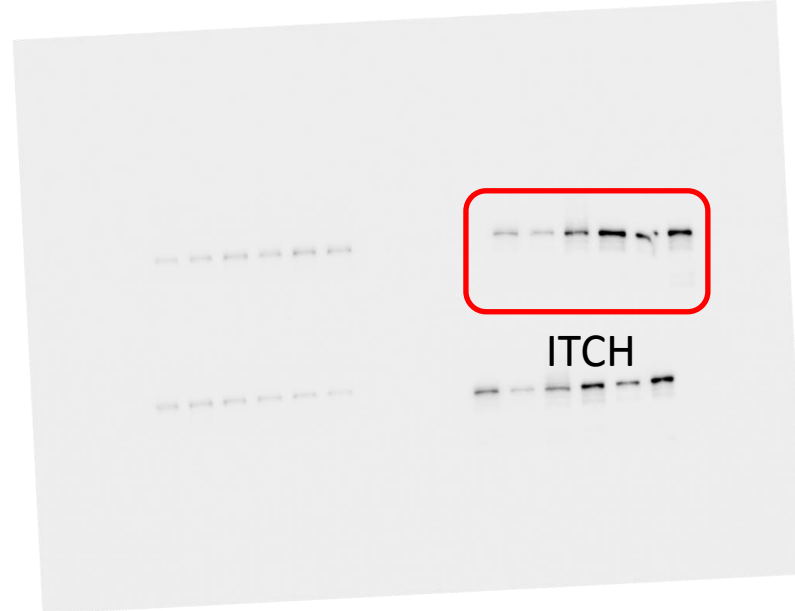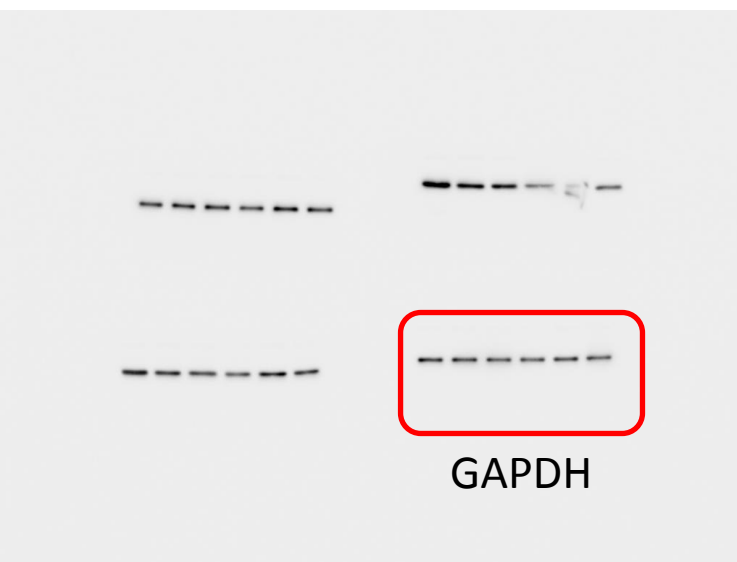

Supplement: Figure 3—source data 1. [file elife-105105-fig3-data1.zip › Figure 3H.pdf]

3C

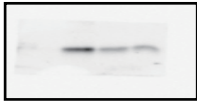

10 kD

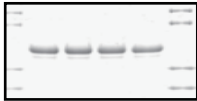

100 kD

55 kD

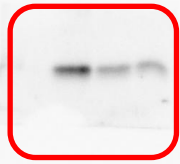

E

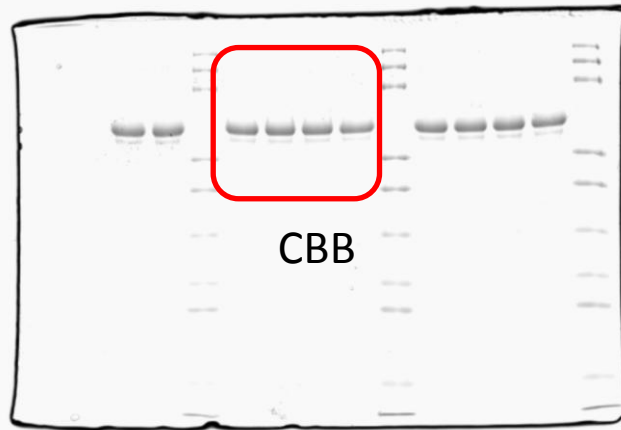

CBB

Supplement: Figure 3—source data 1. [file elife-105105-fig3-data1.zip › Figure 3A.pdf]

3B

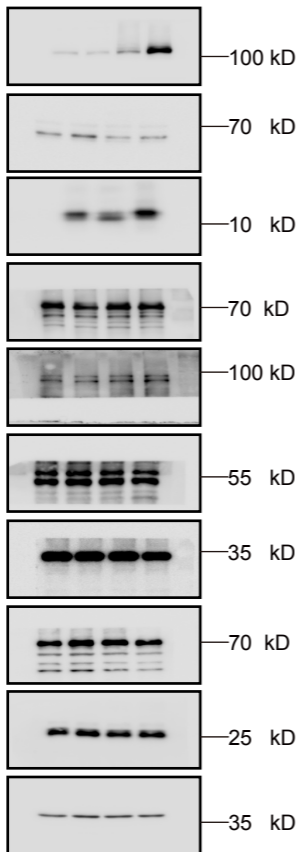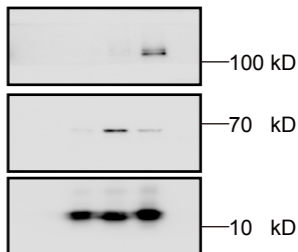

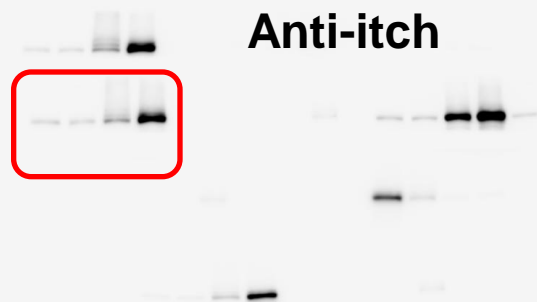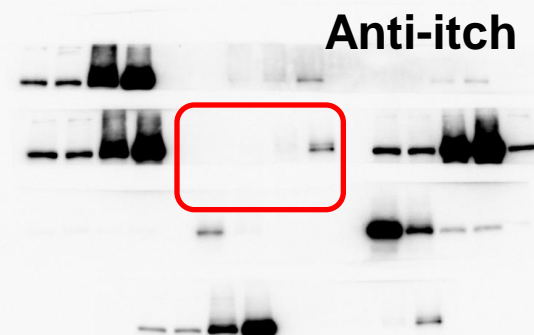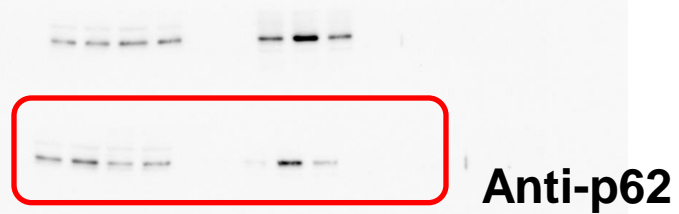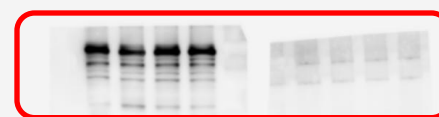

**Anti-FAM134B**

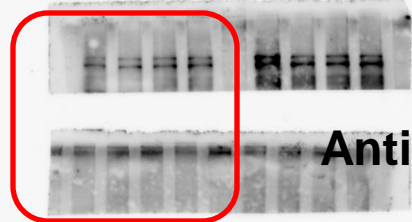

**Anti-NBR1**

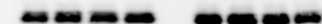

**Anti-RTN3**

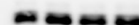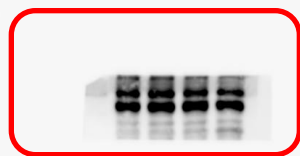

**Anti-NDP52**

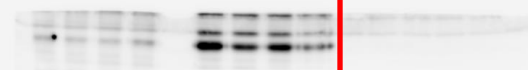

**Anti-NDP52**

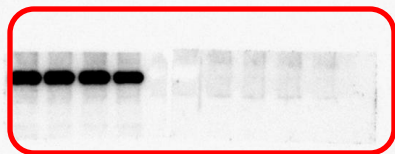

**Anti-NIX**

**Anti-Flag**

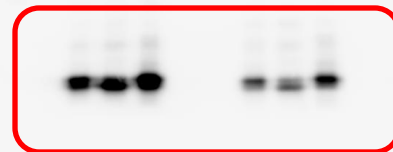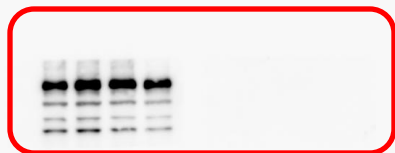

**Anti-OPTN**

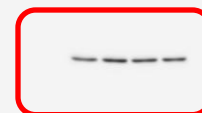

**Anti-GADPH**

Supplement: Figure 3—source data 1. [file elife-105105-fig3-data1.zip › Figure 3B.pdf]

3C

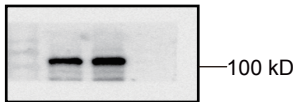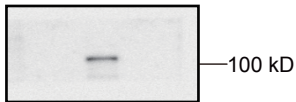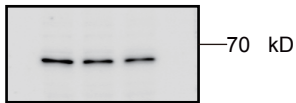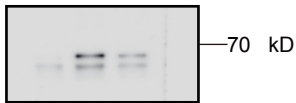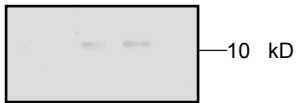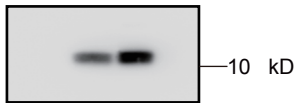

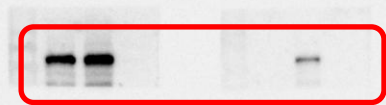

ITCH

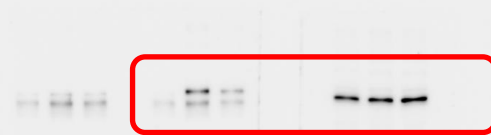

p62

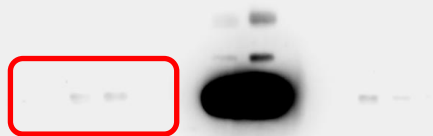

E

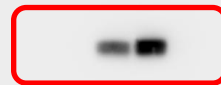

E

Supplement: Figure 3—source data 1. [file elife-105105-fig3-data1.zip › Figure 3C.pdf]

3F

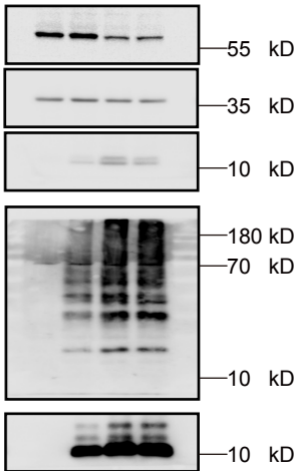

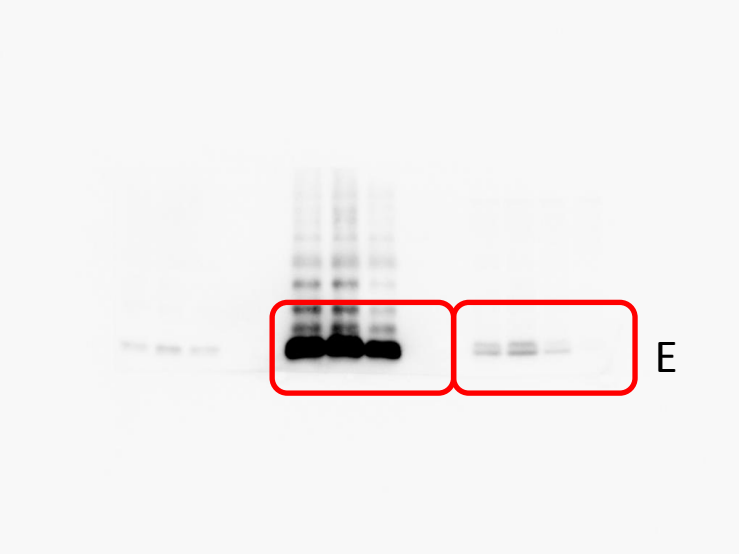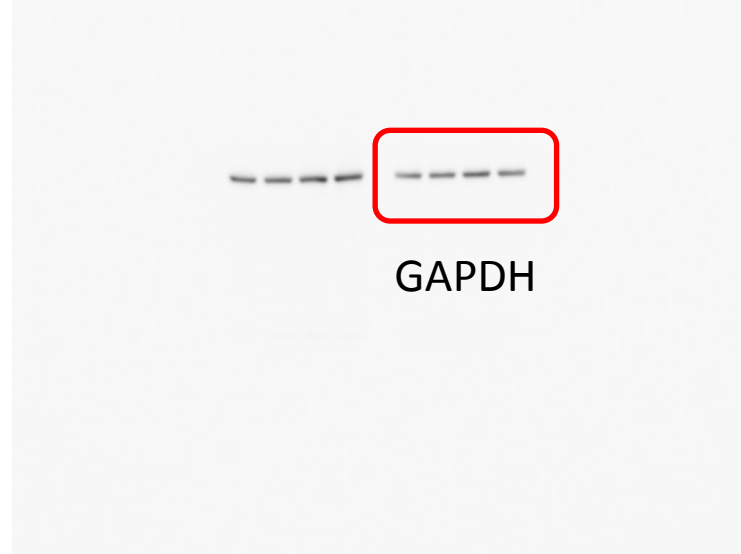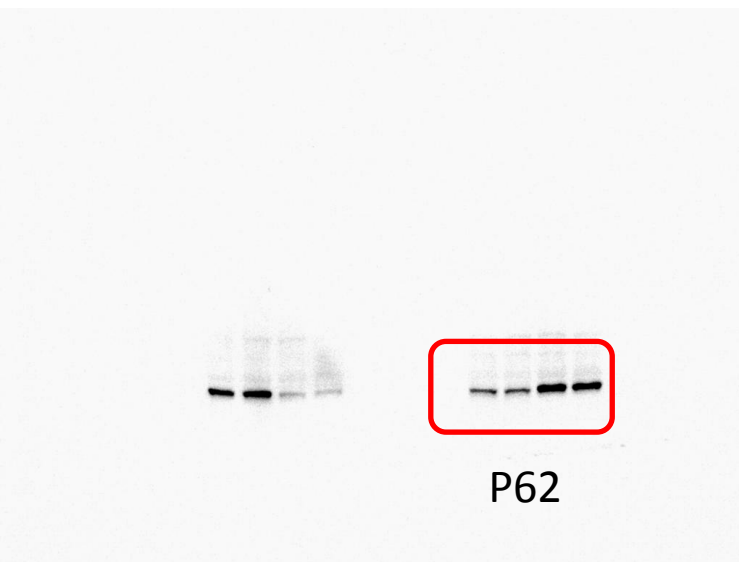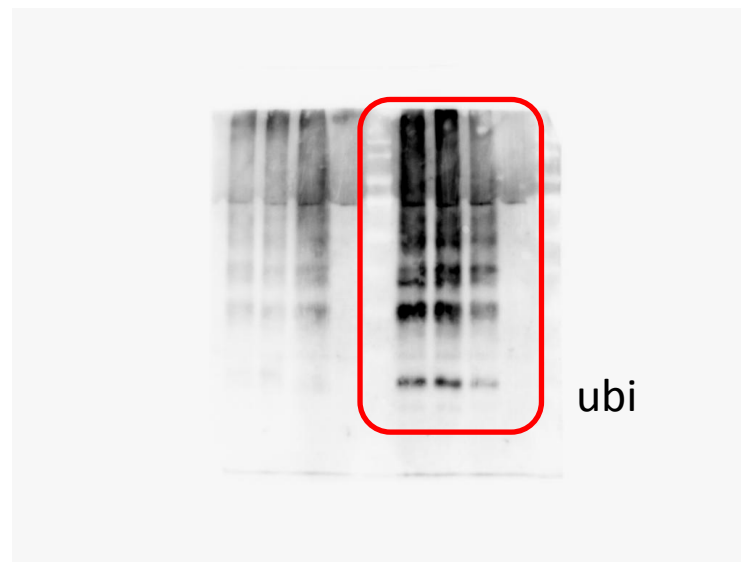

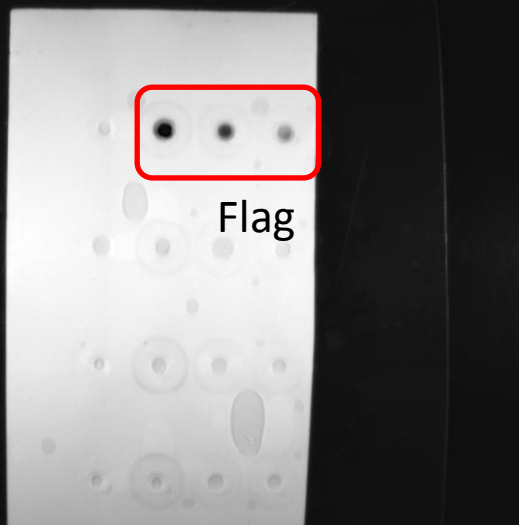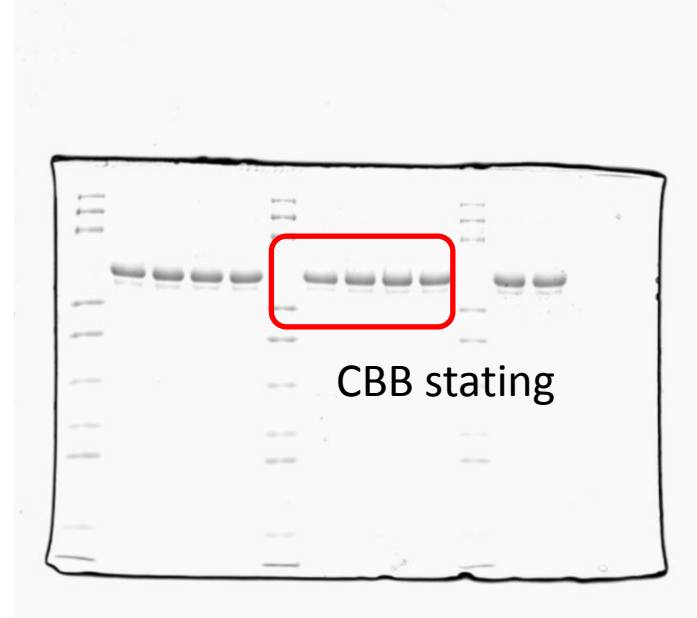

Supplement: Figure 3—source data 1. [file elife-105105-fig3-data1.zip › Figure 3F.pdf]

3G

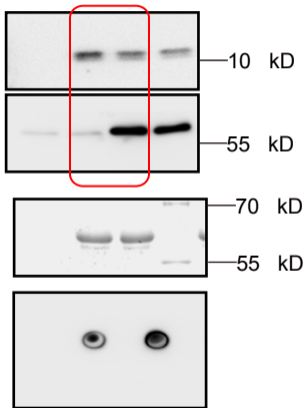

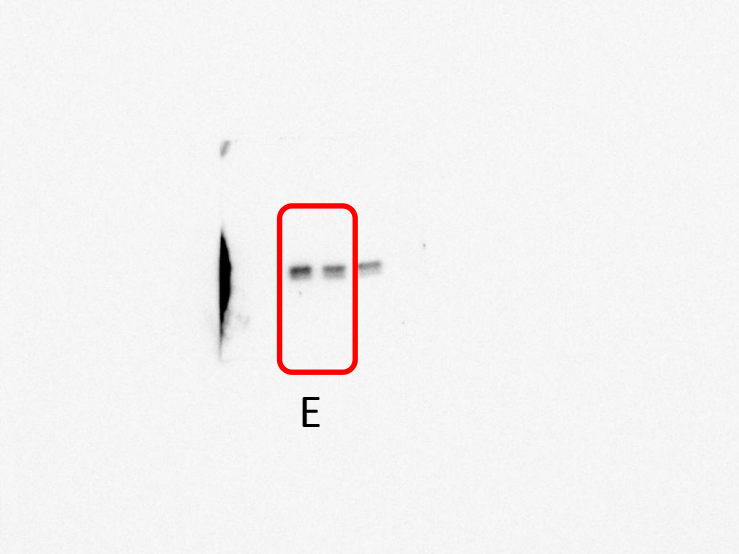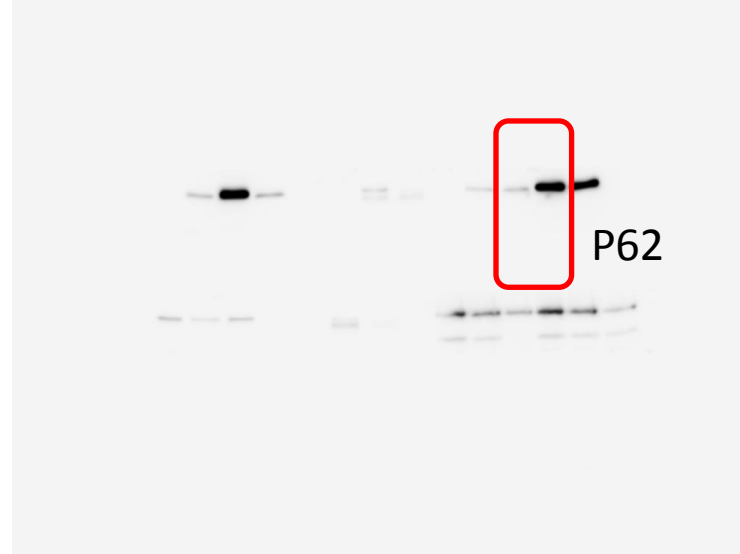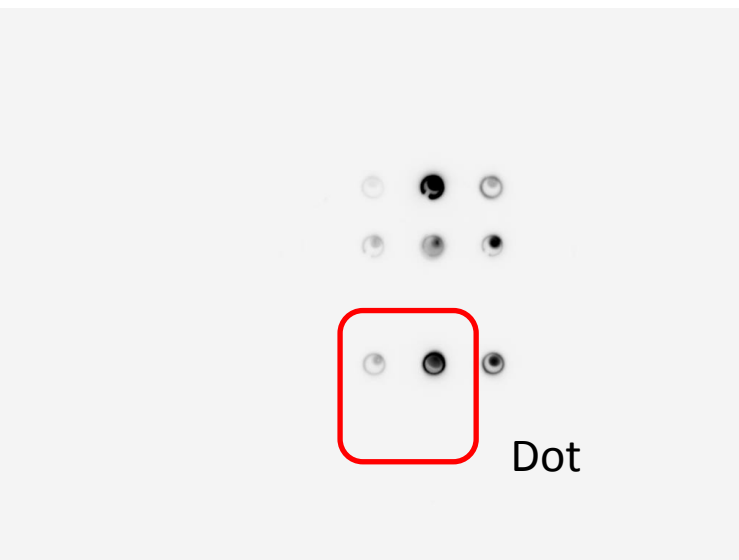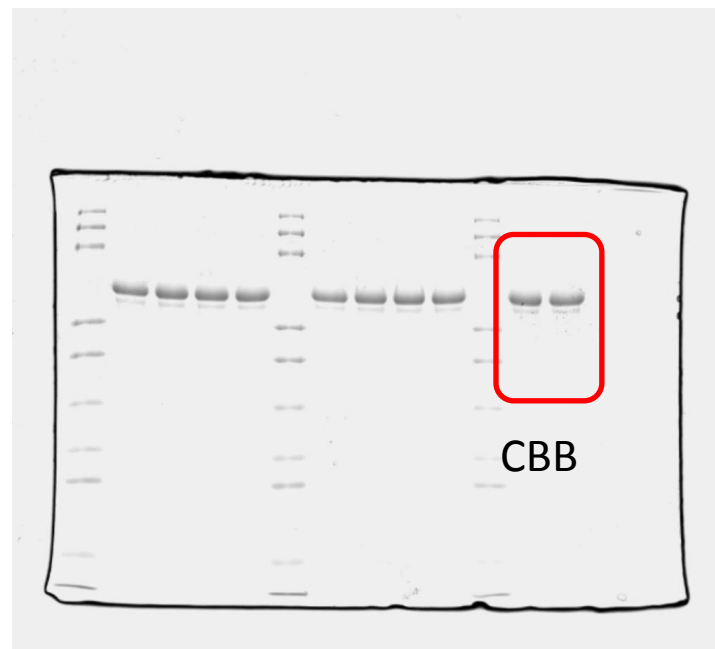

Supplement: Figure 3—source data 1. [file elife-105105-fig3-data1.zip › Figure 3G.pdf]

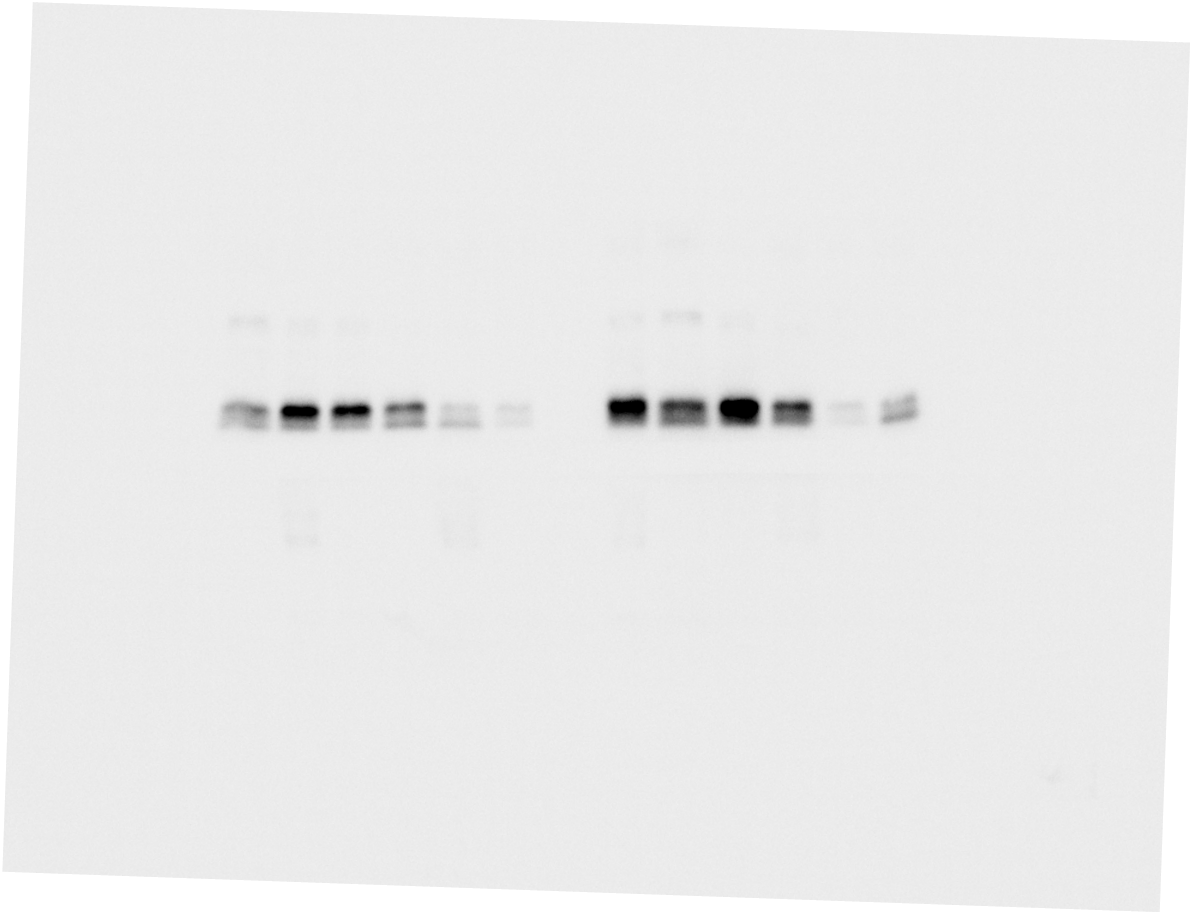

Supplement: Figure 3—source data 2. [file elife-105105-fig3-data2.zip › Figure 3H/3H E.tif]

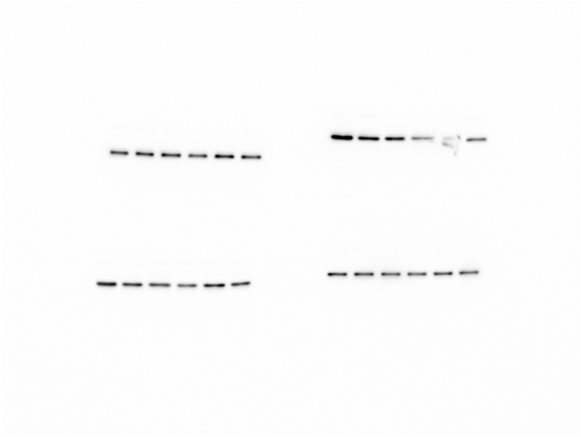

Supplement: Figure 3—source data 2. [file elife-105105-fig3-data2.zip › Figure 3H/3H gapdh.tif]

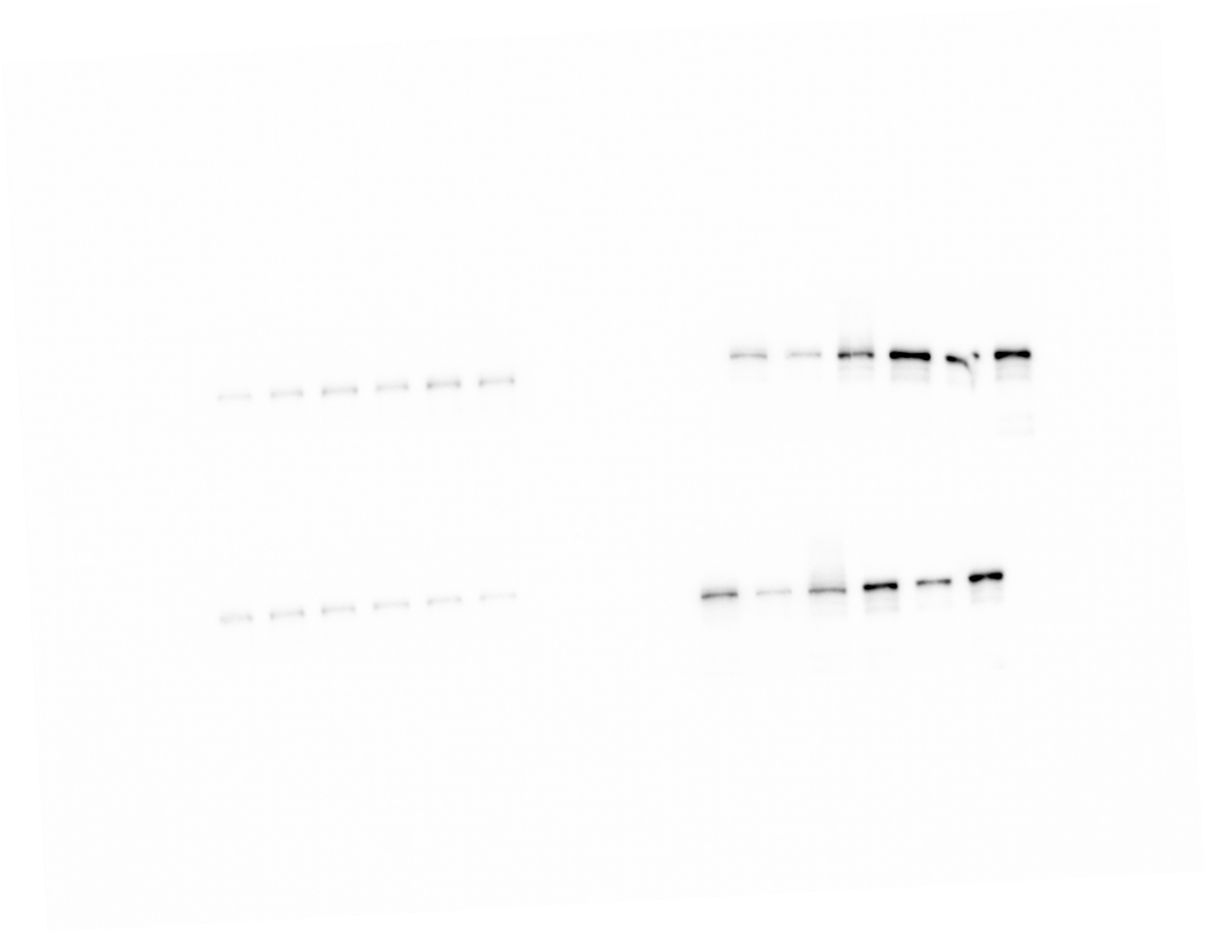

Supplement: Figure 3—source data 2. [file elife-105105-fig3-data2.zip › Figure 3H/3H ITCH.tif]

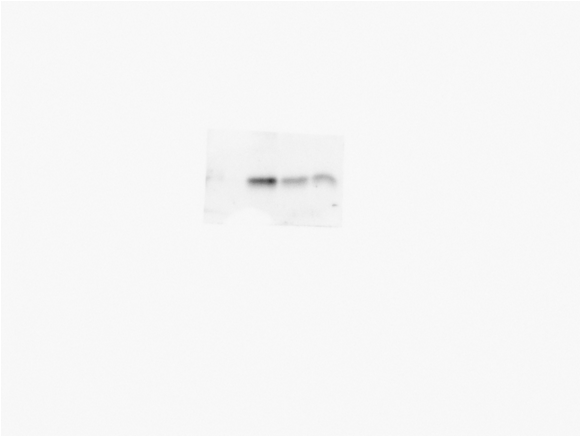

Supplement: Figure 3—source data 2. [file elife-105105-fig3-data2.zip › Figure 3A/3A E.tif]

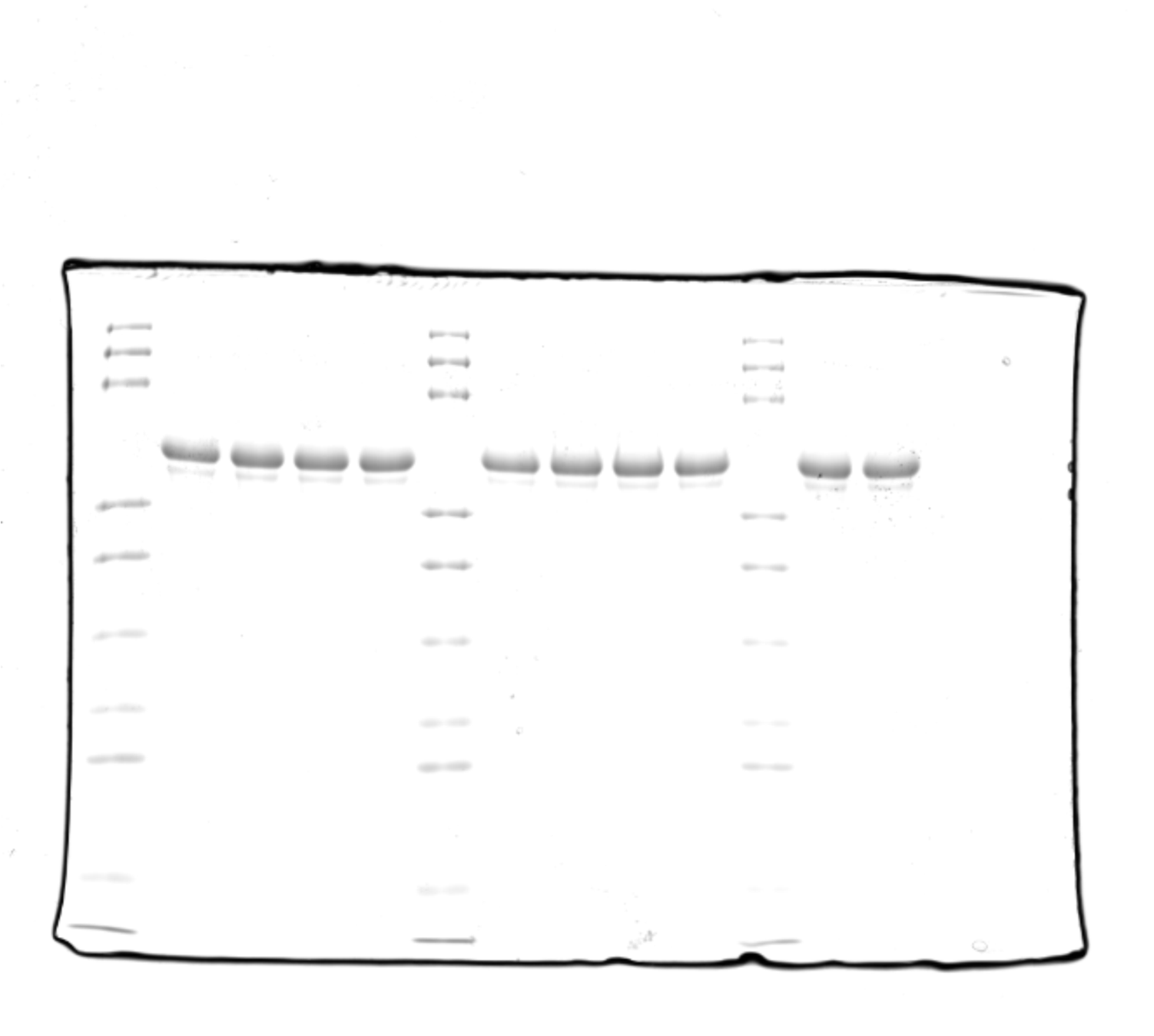

Supplement: Figure 3—source data 2. [file elife-105105-fig3-data2.zip › Figure 3A/3A.tif]

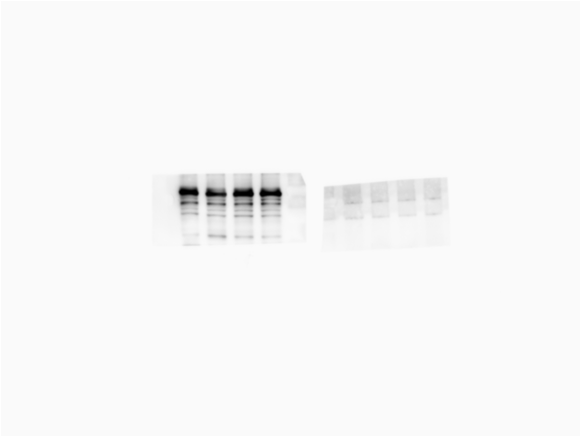

Supplement: Figure 3—source data 2. [file elife-105105-fig3-data2.zip › Figure 3B/FAM134B.tif]

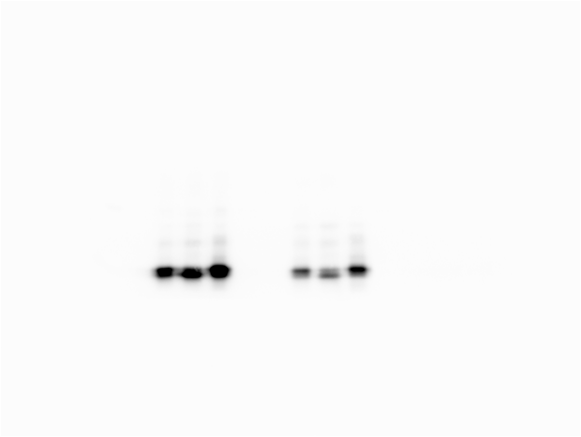

Supplement: Figure 3—source data 2. [file elife-105105-fig3-data2.zip › Figure 3B/FLAG E.tif]

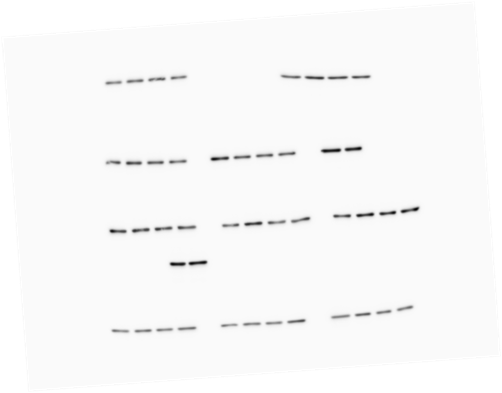

Supplement: Figure 3—source data 2. [file elife-105105-fig3-data2.zip › Figure 3B/GAPDH.tif]

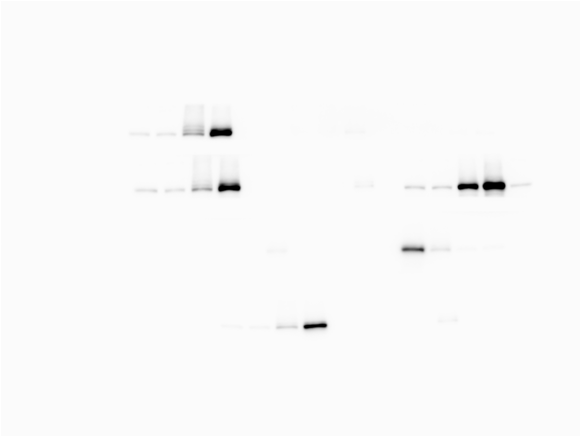

Supplement: Figure 3—source data 2. [file elife-105105-fig3-data2.zip › Figure 3B/ITCH input.tif]

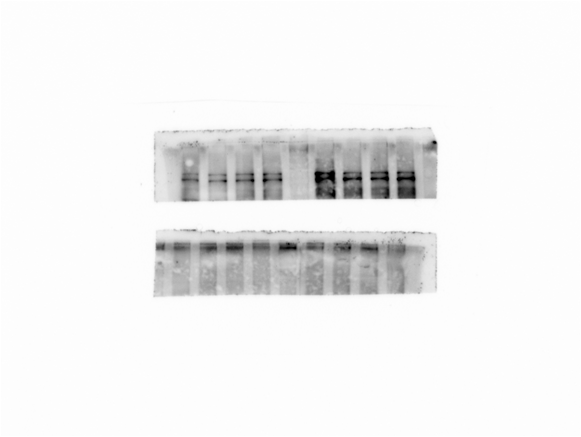

Supplement: Figure 3—source data 2. [file elife-105105-fig3-data2.zip › Figure 3B/NBR1.tif]

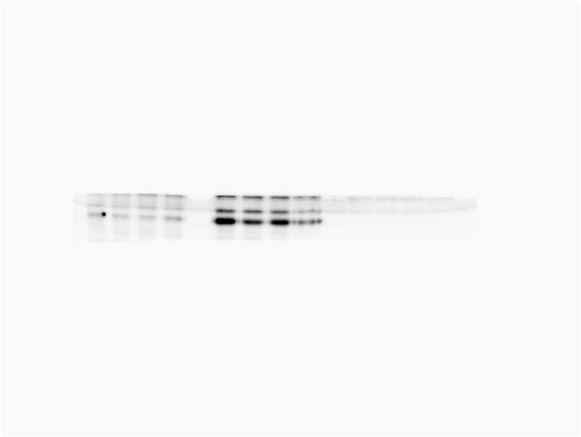

Supplement: Figure 3—source data 2. [file elife-105105-fig3-data2.zip › Figure 3B/NDP52 IP.tif]

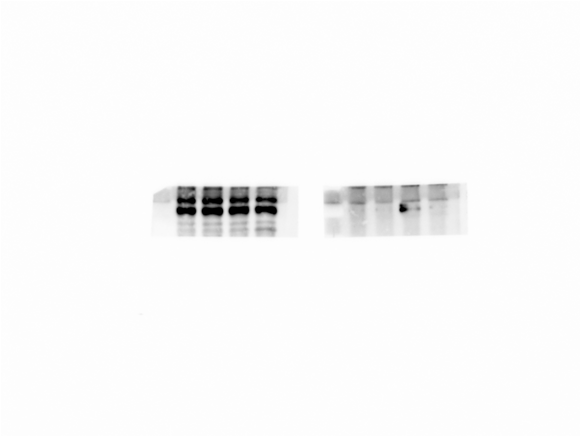

Supplement: Figure 3—source data 2. [file elife-105105-fig3-data2.zip › Figure 3B/NDP52.tif]

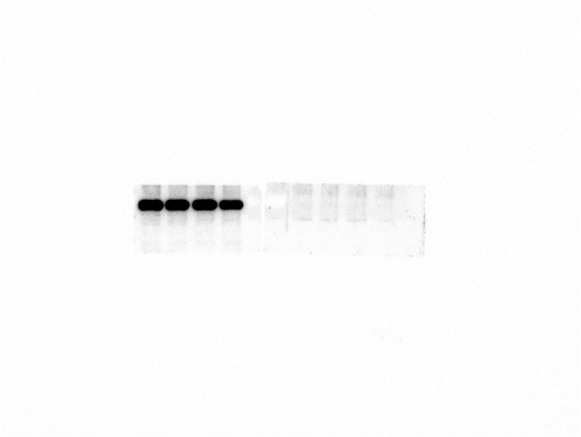

Supplement: Figure 3—source data 2. [file elife-105105-fig3-data2.zip › Figure 3B/NIX.tif]

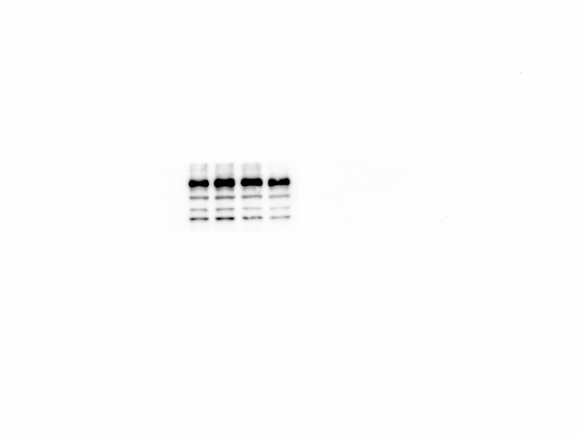

Supplement: Figure 3—source data 2. [file elife-105105-fig3-data2.zip › Figure 3B/OPTN.tif]

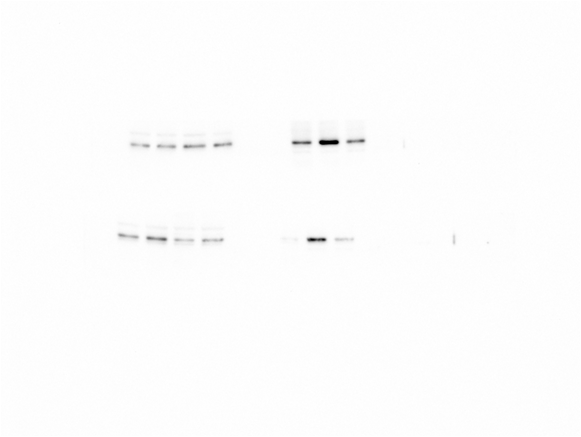

Supplement: Figure 3—source data 2. [file elife-105105-fig3-data2.zip › Figure 3B/p62.tif]

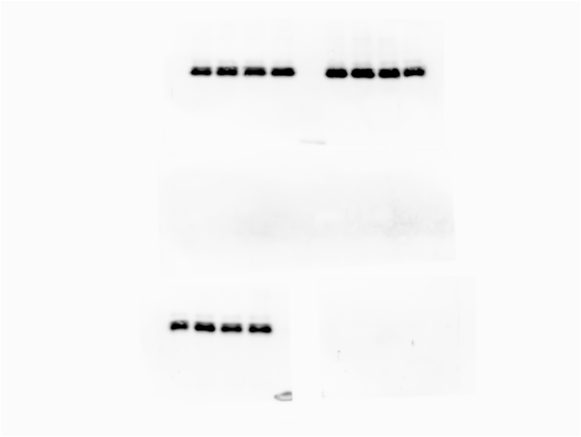

Supplement: Figure 3—source data 2. [file elife-105105-fig3-data2.zip › Figure 3B/RTN3.tif]

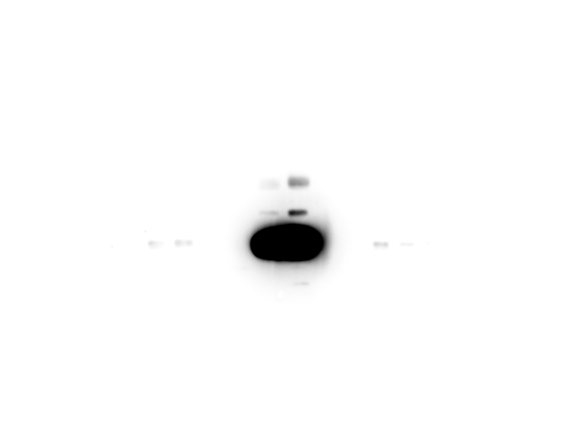

Supplement: Figure 3—source data 2. [file elife-105105-fig3-data2.zip › Figure 3C/E input.tif]

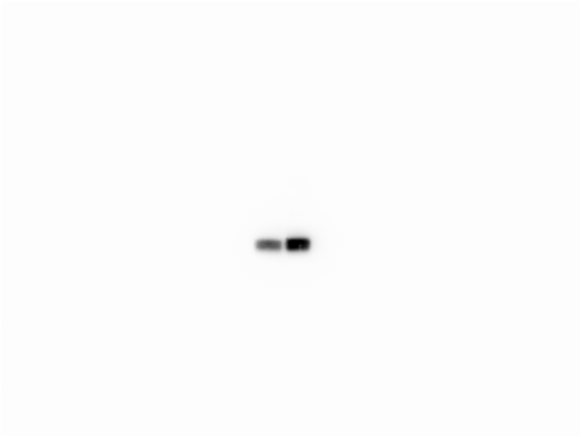

Supplement: Figure 3—source data 2. [file elife-105105-fig3-data2.zip › Figure 3C/E IP.tif]

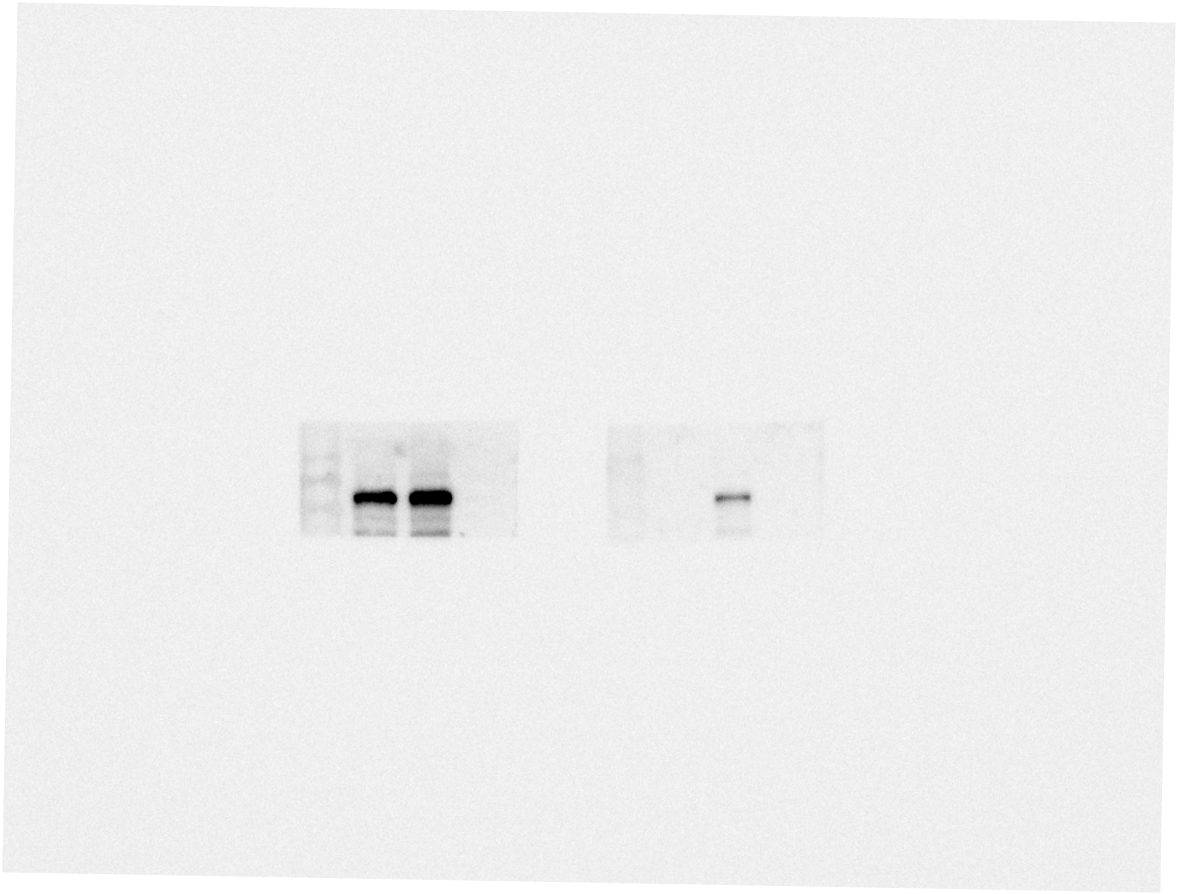

Supplement: Figure 3—source data 2. [file elife-105105-fig3-data2.zip › Figure 3C/ITCH.tif]

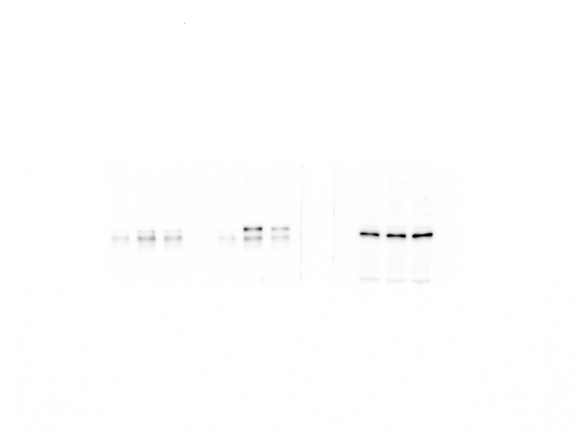

Supplement: Figure 3—source data 2. [file elife-105105-fig3-data2.zip › Figure 3C/p62.tif]

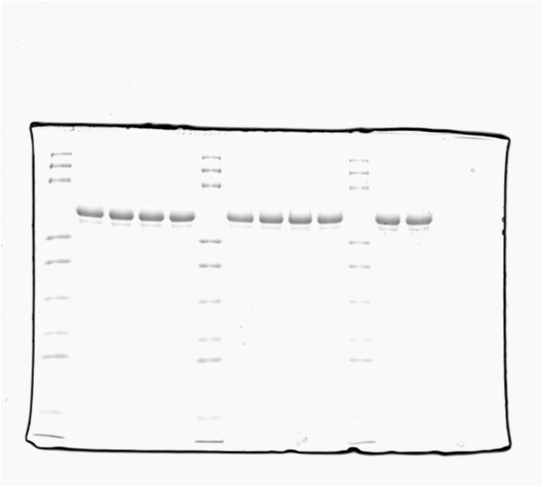

Supplement: Figure 3—source data 2. [file elife-105105-fig3-data2.zip › Figure 3F/CBB.tif]

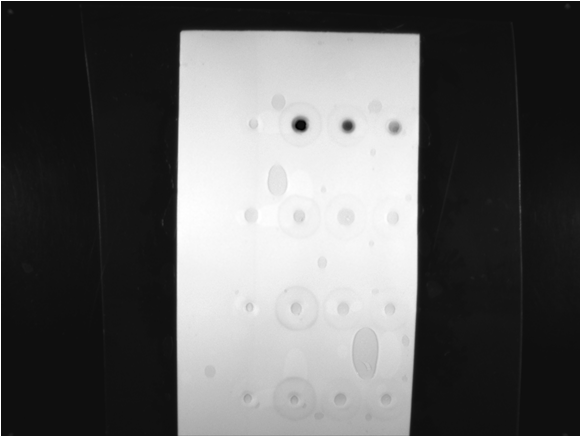

Supplement: Figure 3—source data 2. [file elife-105105-fig3-data2.zip › Figure 3F/E dot.tif]

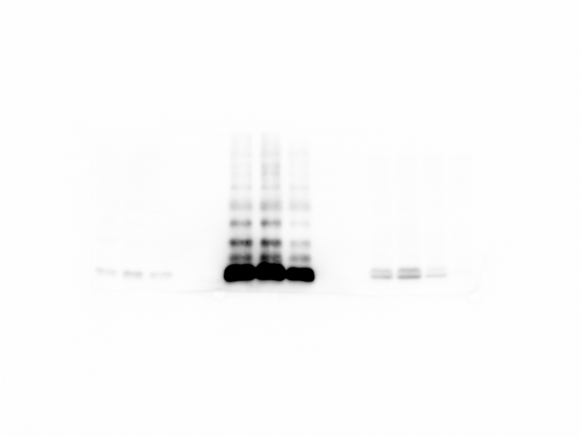

Supplement: Figure 3—source data 2. [file elife-105105-fig3-data2.zip › Figure 3F/E.tif]

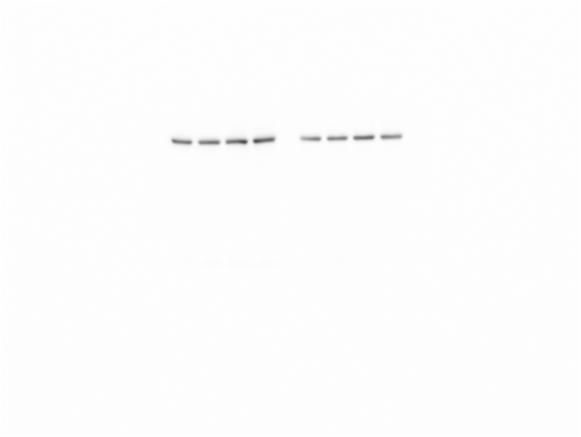

Supplement: Figure 3—source data 2. [file elife-105105-fig3-data2.zip › Figure 3F/gapdh.tif]

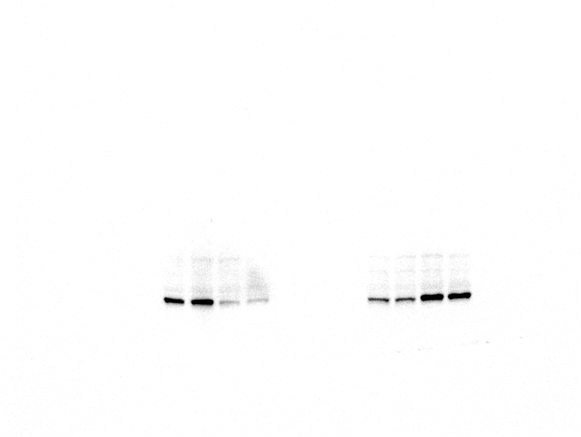

Supplement: Figure 3—source data 2. [file elife-105105-fig3-data2.zip › Figure 3F/P62.tif]

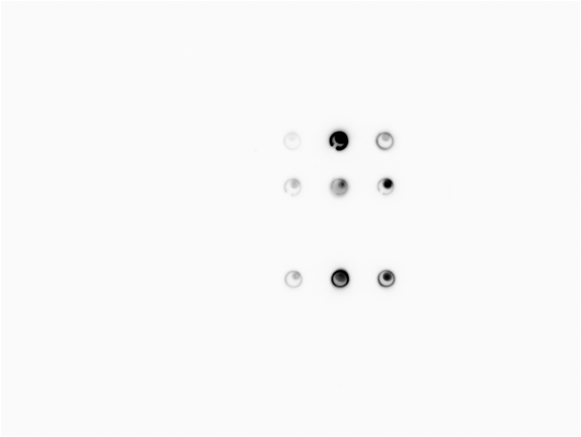

Supplement: Figure 3—source data 2. [file elife-105105-fig3-data2.zip › Figure 3G/e medium.tif]

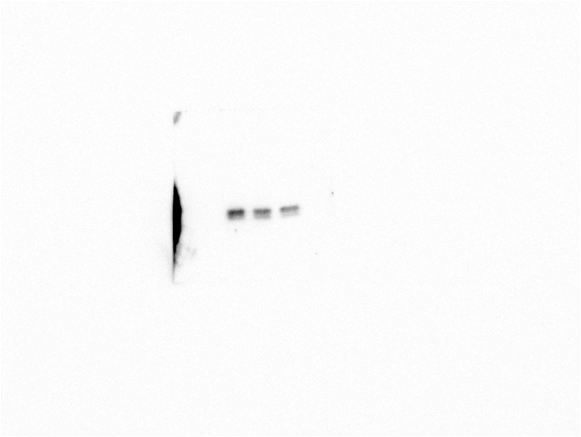

Supplement: Figure 3—source data 2. [file elife-105105-fig3-data2.zip › Figure 3G/e.tif]

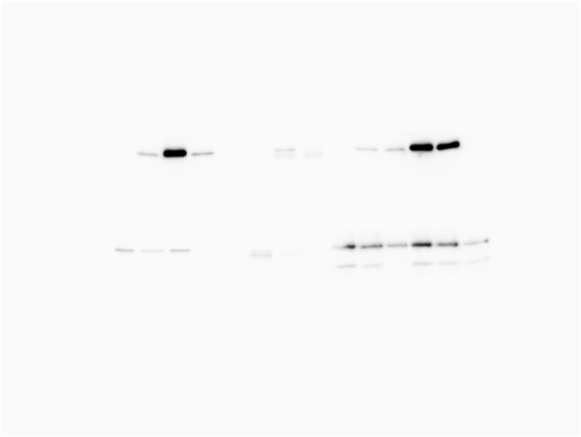

Supplement: Figure 3—source data 2. [file elife-105105-fig3-data2.zip › Figure 3G/p62.tif]

# S3H

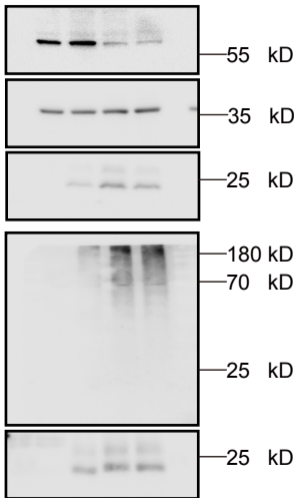

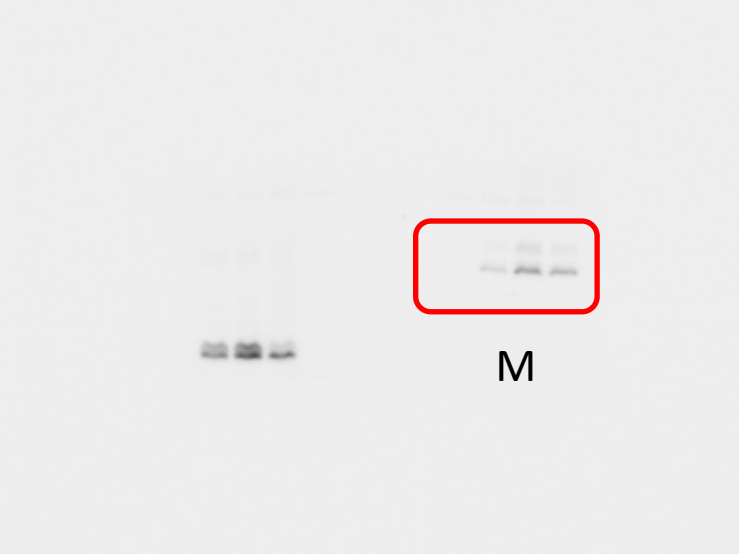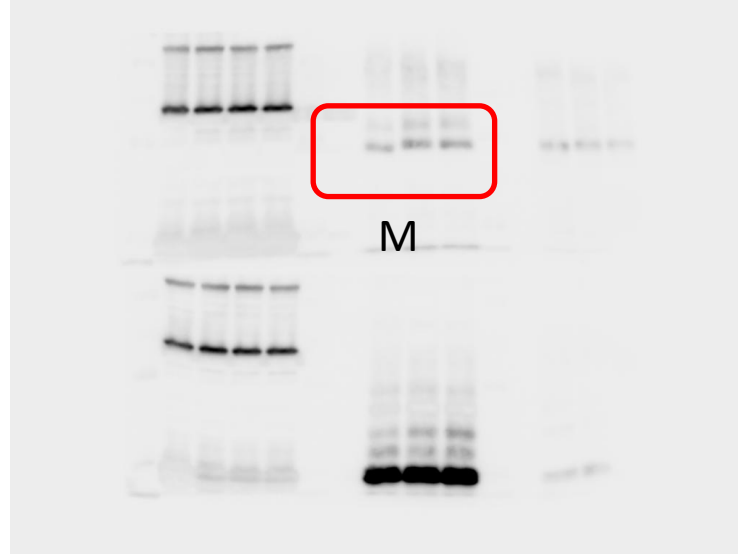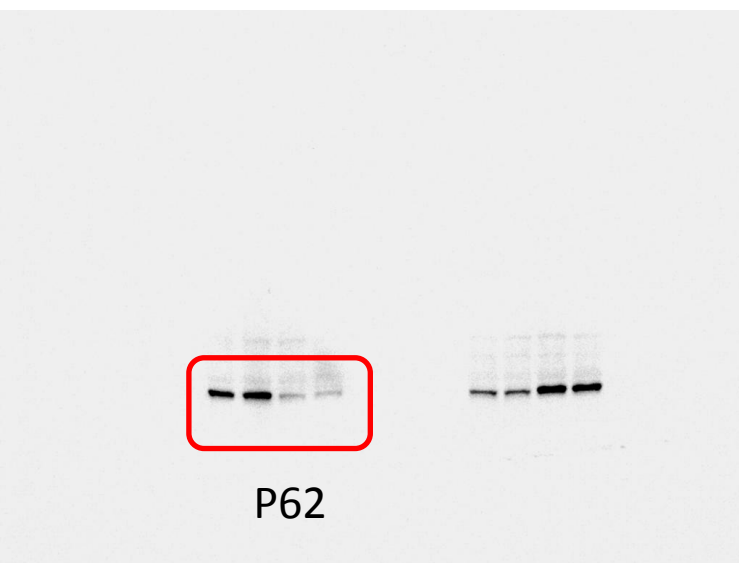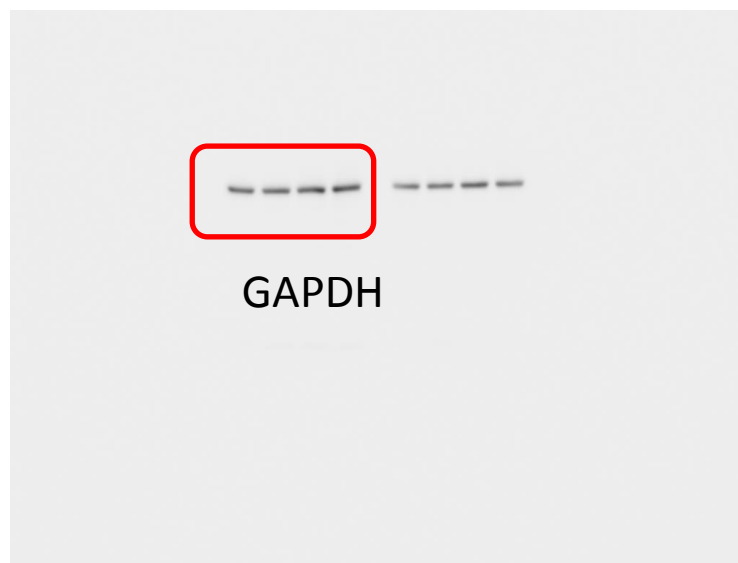

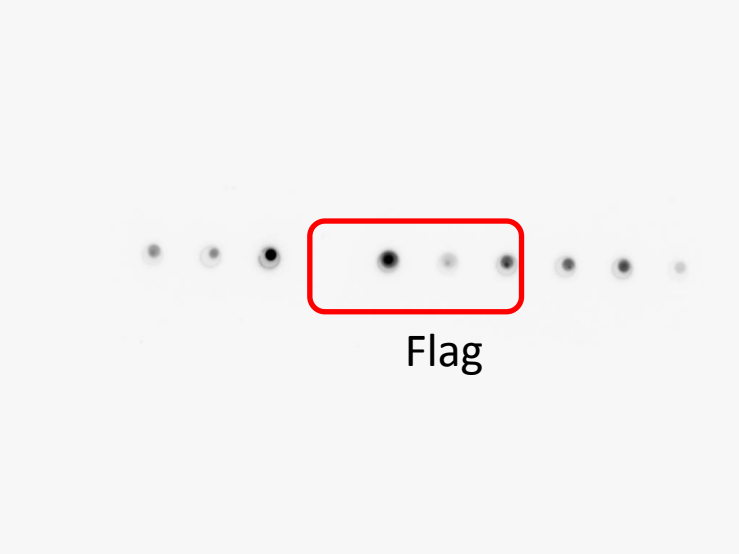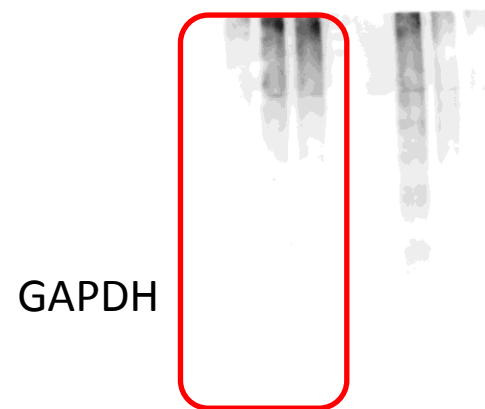

Supplement: Figure 3—figure supplement 1—source data 1. [file elife-105105-fig3-figsupp1-data1.zip › Figure 3-figure supplement 1H.pdf]

S3I

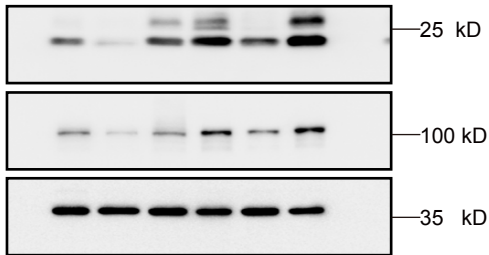

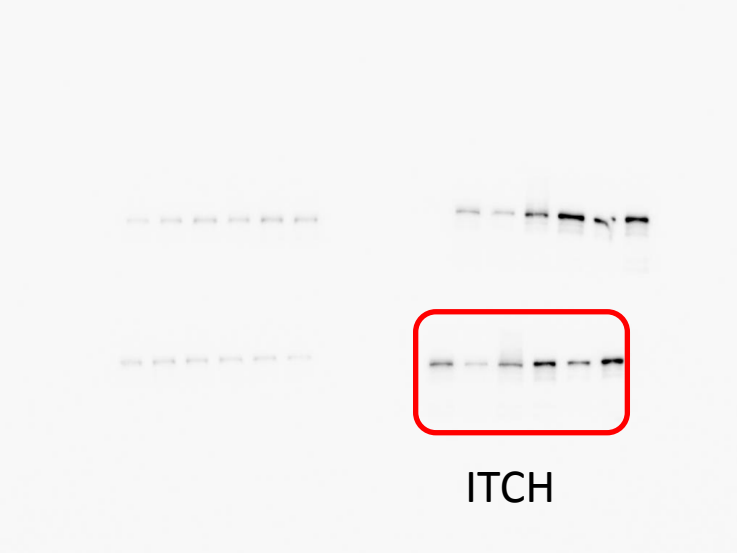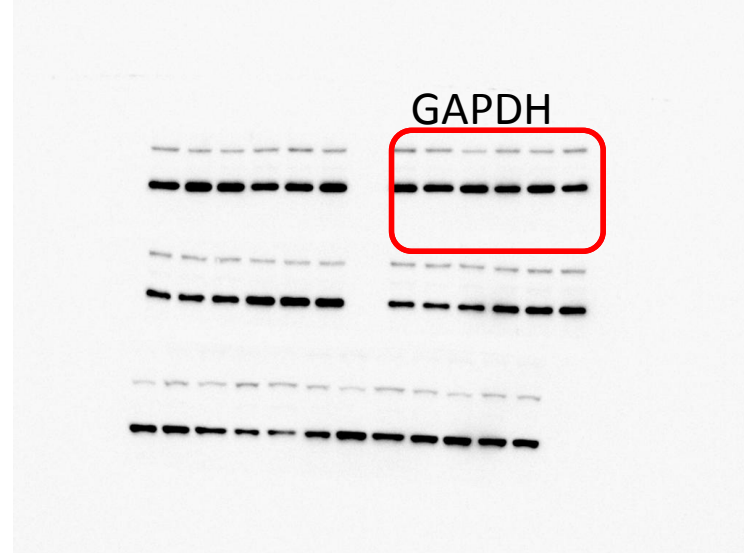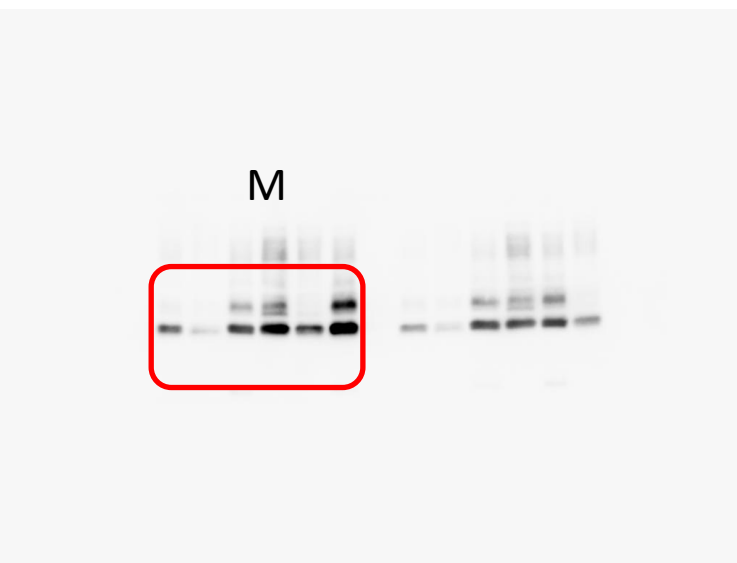

Supplement: Figure 3—figure supplement 1—source data 1. [file elife-105105-fig3-figsupp1-data1.zip › Figure 3-figure supplement 1I.pdf]

# S3A

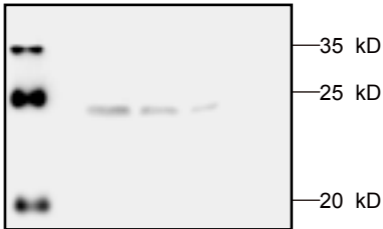

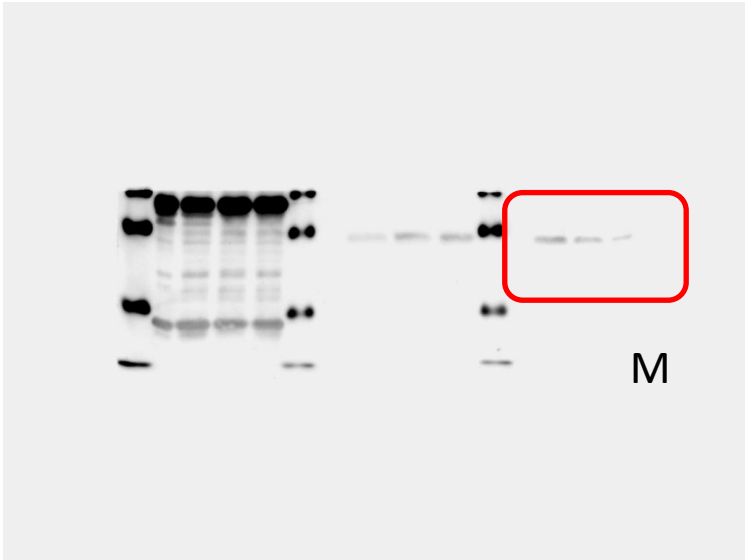

Supplement: Figure 3—figure supplement 1—source data 1. [file elife-105105-fig3-figsupp1-data1.zip › Figure 3-figure supplement 1A.pdf]

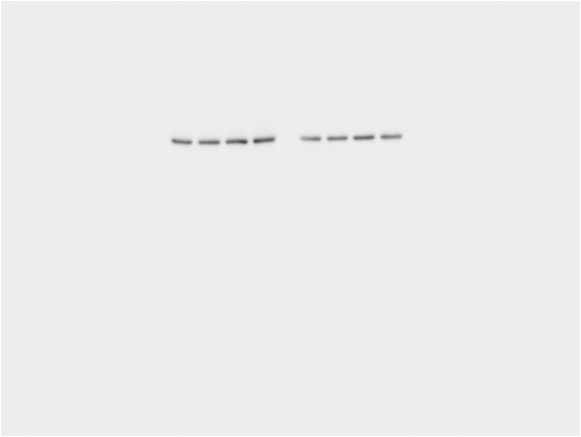

Supplement: Figure 3—figure supplement 1—source data 2. [file elife-105105-fig3-figsupp1-data2.zip › Figure 3-figure supplement 1H/gapdh.tif]

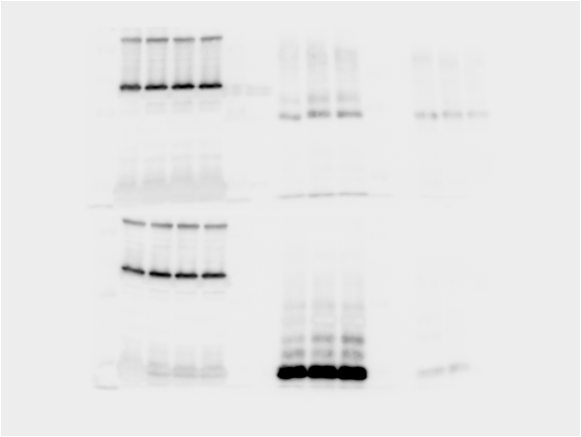

Supplement: Figure 3—figure supplement 1—source data 2. [file elife-105105-fig3-figsupp1-data2.zip › Figure 3-figure supplement 1H/M dip.tif]

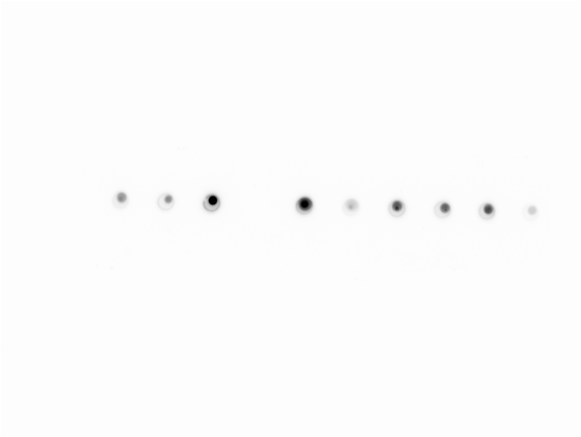

Supplement: Figure 3—figure supplement 1—source data 2. [file elife-105105-fig3-figsupp1-data2.zip › Figure 3-figure supplement 1H/M dot.tif]

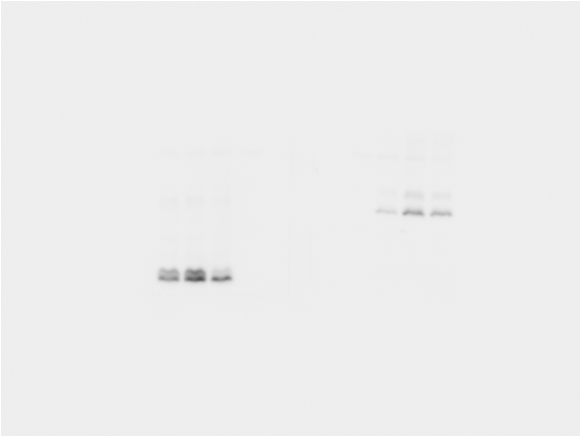

Supplement: Figure 3—figure supplement 1—source data 2. [file elife-105105-fig3-figsupp1-data2.zip › Figure 3-figure supplement 1H/M input.tif]

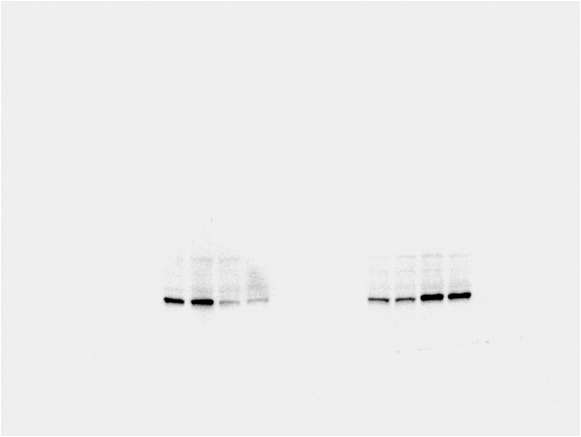

Supplement: Figure 3—figure supplement 1—source data 2. [file elife-105105-fig3-figsupp1-data2.zip › Figure 3-figure supplement 1H/p62.tif]

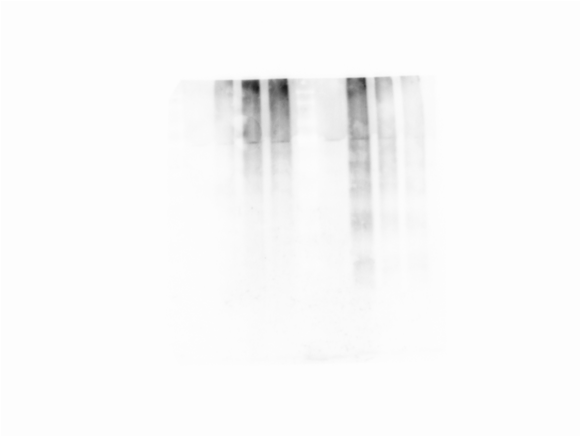

Supplement: Figure 3—figure supplement 1—source data 2. [file elife-105105-fig3-figsupp1-data2.zip › Figure 3-figure supplement 1H/ubi.tif]

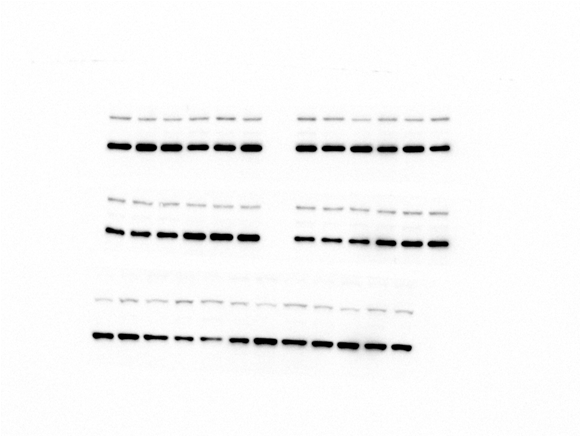

Supplement: Figure 3—figure supplement 1—source data 2. [file elife-105105-fig3-figsupp1-data2.zip › Figure 3-figure supplement 1I/GAPDH.tif]

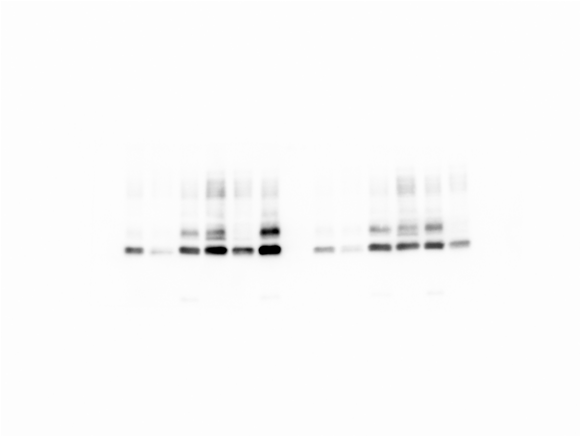

Supplement: Figure 3—figure supplement 1—source data 2. [file elife-105105-fig3-figsupp1-data2.zip › Figure 3-figure supplement 1I/M.tif]

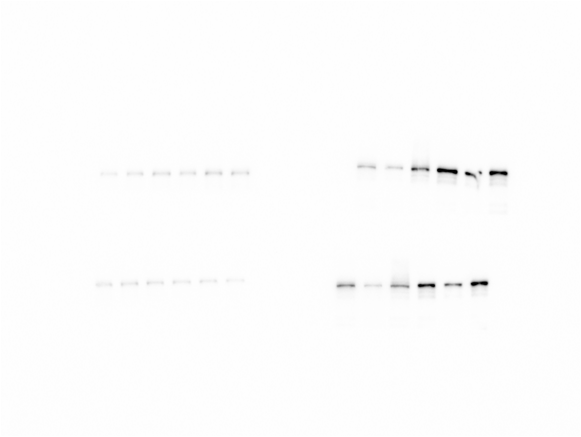

Supplement: Figure 3—figure supplement 1—source data 2. [file elife-105105-fig3-figsupp1-data2.zip › Figure 3-figure supplement 1I/S3I.tif]

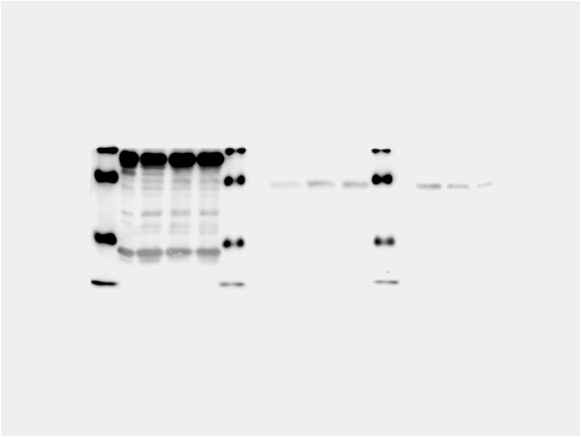

Supplement: Figure 3—figure supplement 1—source data 2. [file elife-105105-fig3-figsupp1-data2.zip › Figure 3-figure supplement 1A/S3A M.tif]

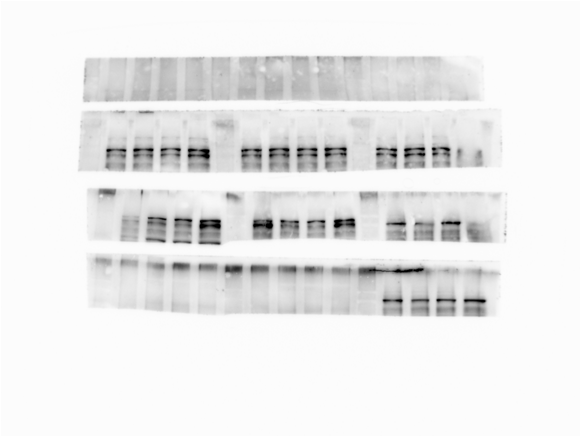

Supplement: Figure 3—figure supplement 1—source data 2. [file elife-105105-fig3-figsupp1-data2.zip › Figure 3-figure supplement 1C/fam134b.tif]

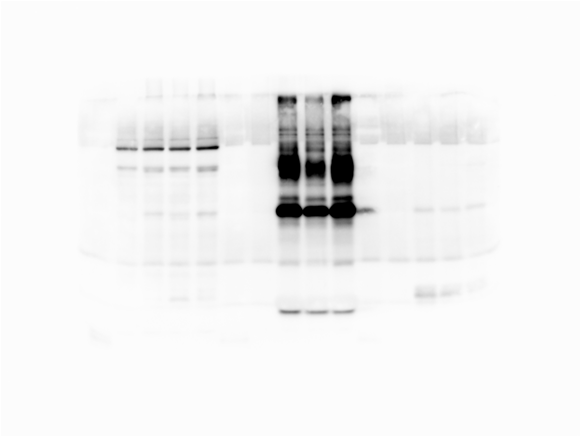

Supplement: Figure 3—figure supplement 1—source data 2. [file elife-105105-fig3-figsupp1-data2.zip › Figure 3-figure supplement 1C/flag.tif]

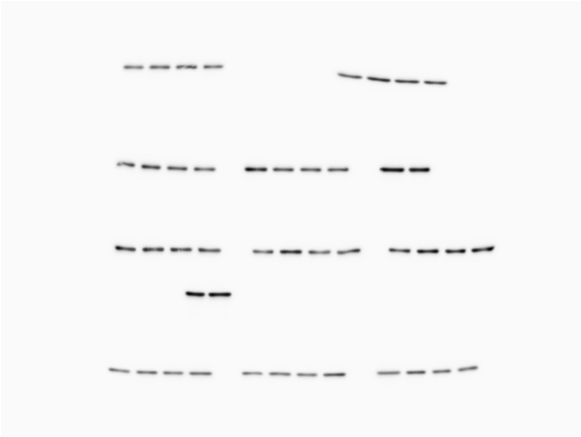

Supplement: Figure 3—figure supplement 1—source data 2. [file elife-105105-fig3-figsupp1-data2.zip › Figure 3-figure supplement 1C/gapdh.tif]

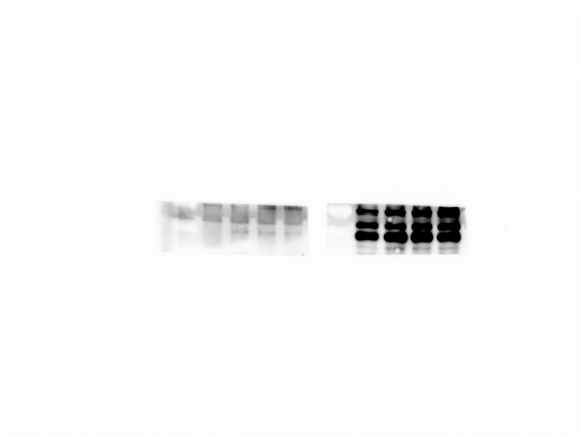

Supplement: Figure 3—figure supplement 1—source data 2. [file elife-105105-fig3-figsupp1-data2.zip › Figure 3-figure supplement 1C/ndp52.tif]

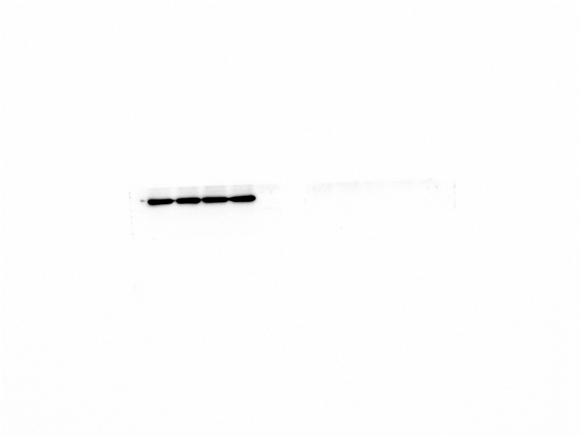

Supplement: Figure 3—figure supplement 1—source data 2. [file elife-105105-fig3-figsupp1-data2.zip › Figure 3-figure supplement 1C/NIX.tif]

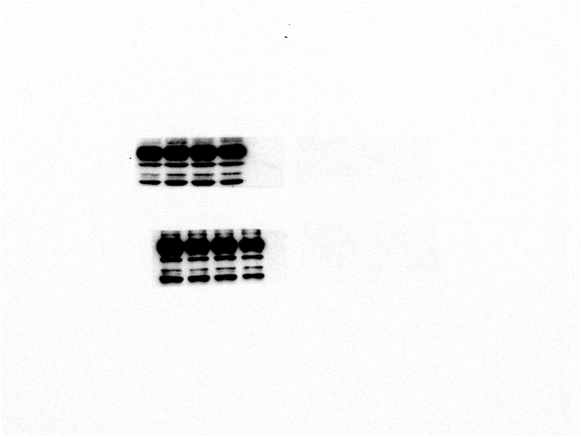

Supplement: Figure 3—figure supplement 1—source data 2. [file elife-105105-fig3-figsupp1-data2.zip › Figure 3-figure supplement 1C/optn.tif]

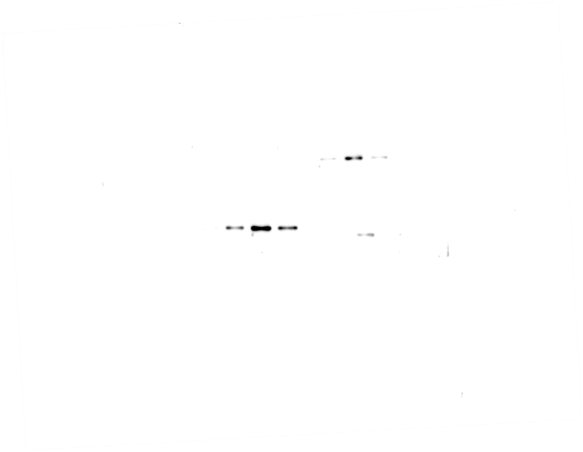

Supplement: Figure 3—figure supplement 1—source data 2. [file elife-105105-fig3-figsupp1-data2.zip › Figure 3-figure supplement 1C/p62 ip.tif]
