# Supplementary material for: Ubiquitin ligase ITCH regulates life cycle of SARS-CoV-2 virus
Source: eLife. 2026 May 29;14:RP105105. doi: 10.7554/eLife.105105 (PMC13221179; doi:10.7554/eLife.105105)
Supplement: Figure 3—figure supplement 1—source data 1. [file elife-105105-fig3-figsupp1-data1.zip › Figure 3-figure supplement 1C.pdf]

S3C

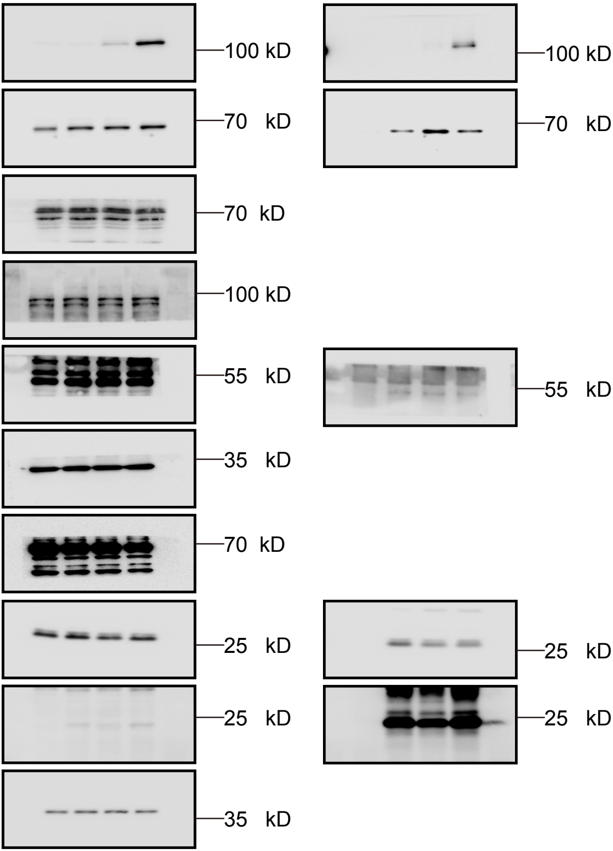
100 kD
100 kD
70 kD
70 kD
70 kD
100 kD
55 kD
55 kD
35 kD
70 kD
25 kD
25 kD
25 kD
25 kD
35 kD

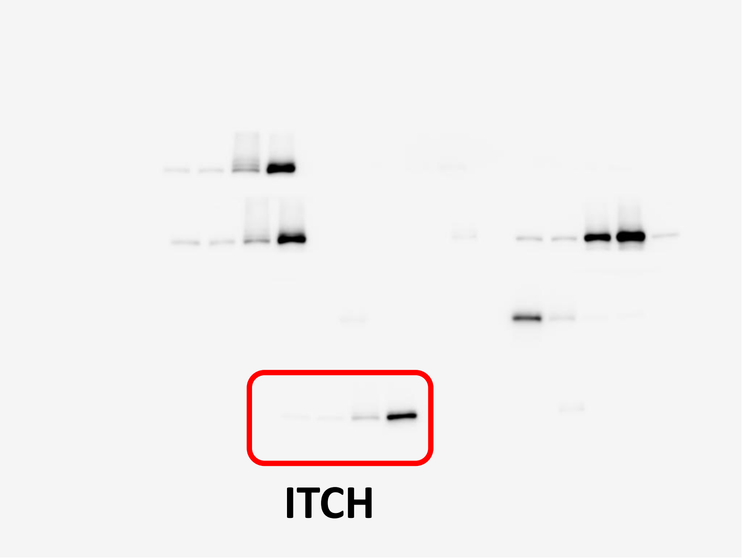
ITCH
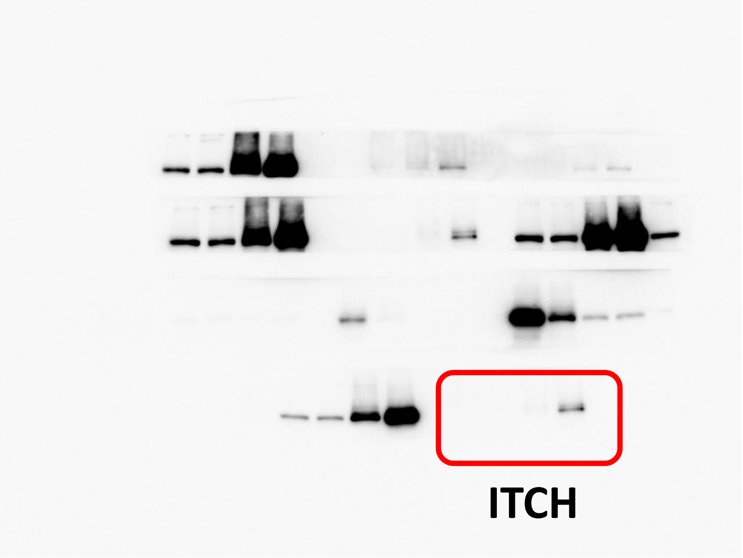
ITCH
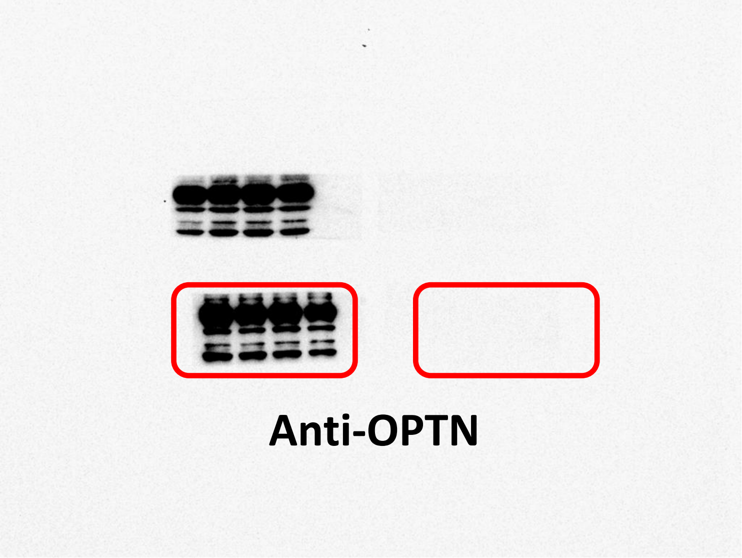
Anti-OPTN
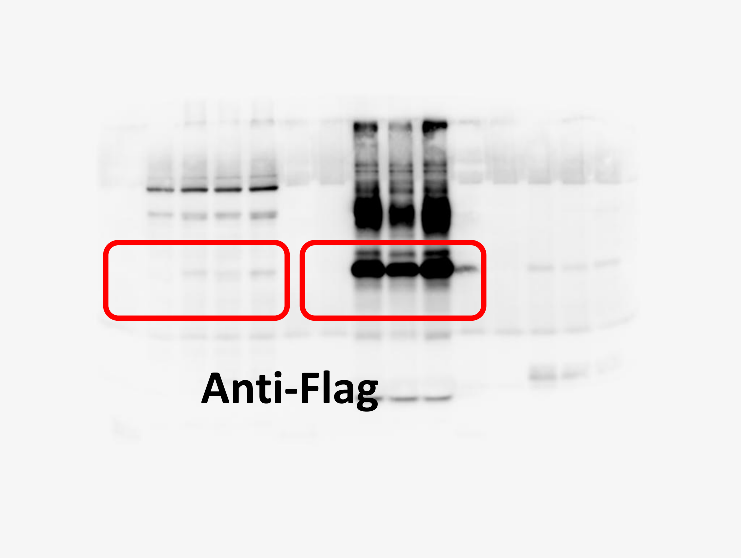
Anti-Flag

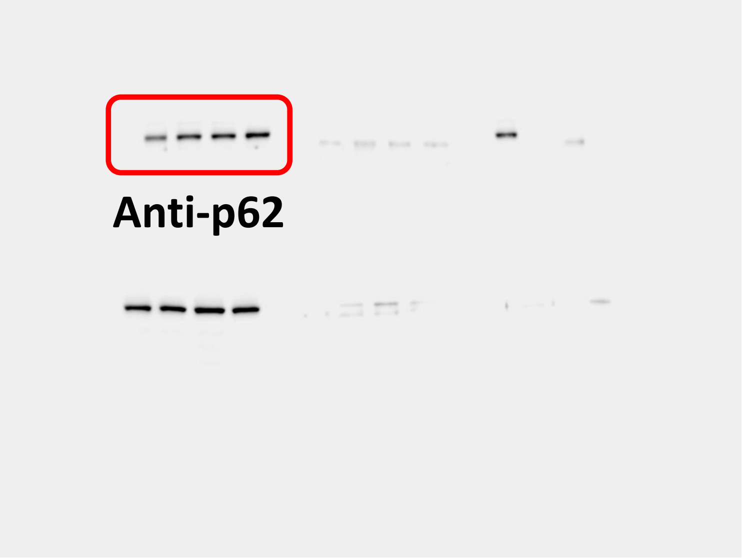
Anti-p62

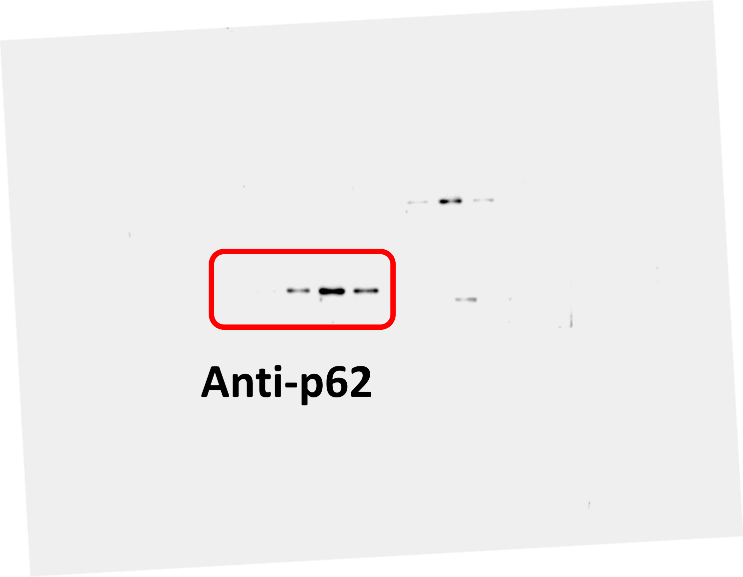
Anti-p62

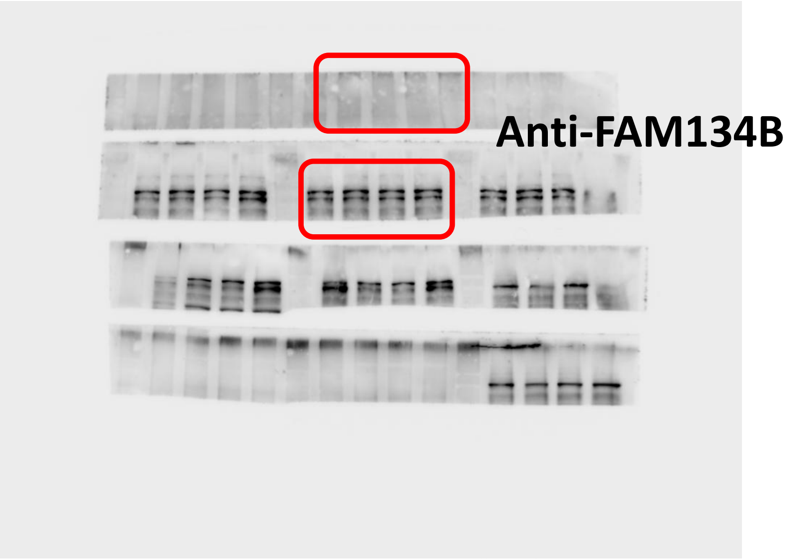
Anti-FAM134B

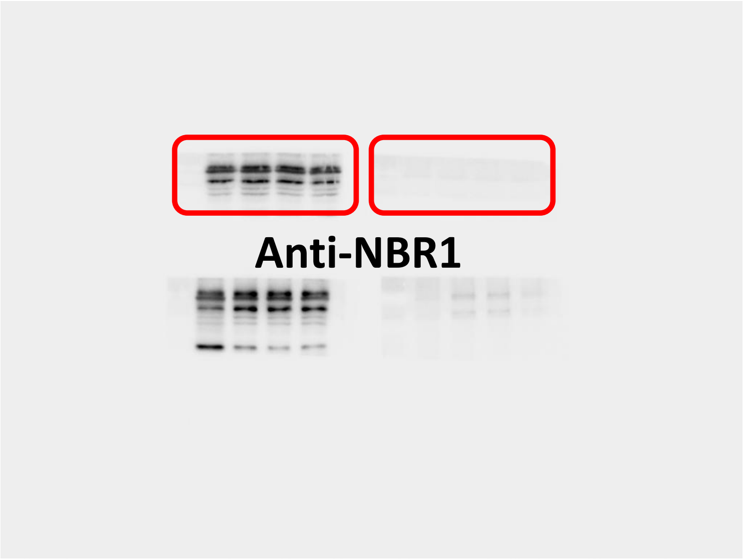
Anti-NBR1

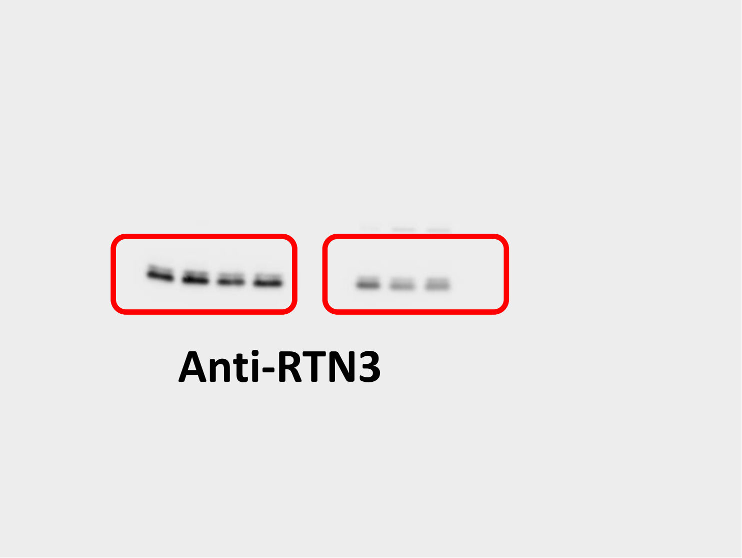
Anti-RTN3

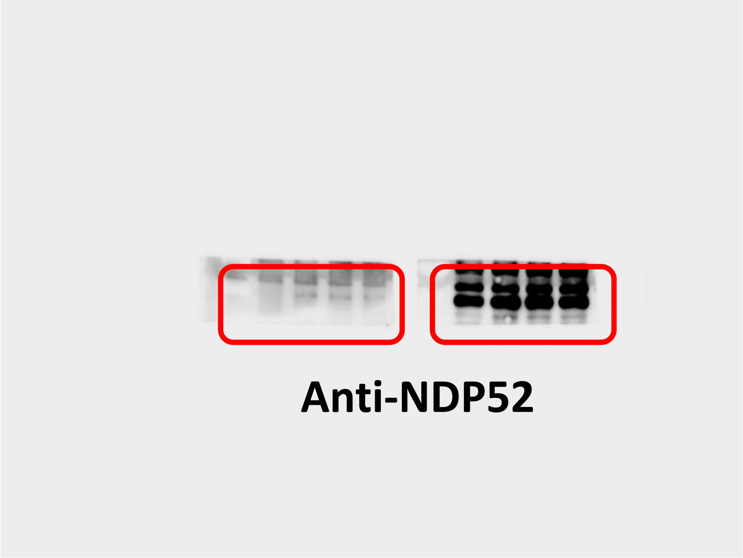
Anti-NDP52

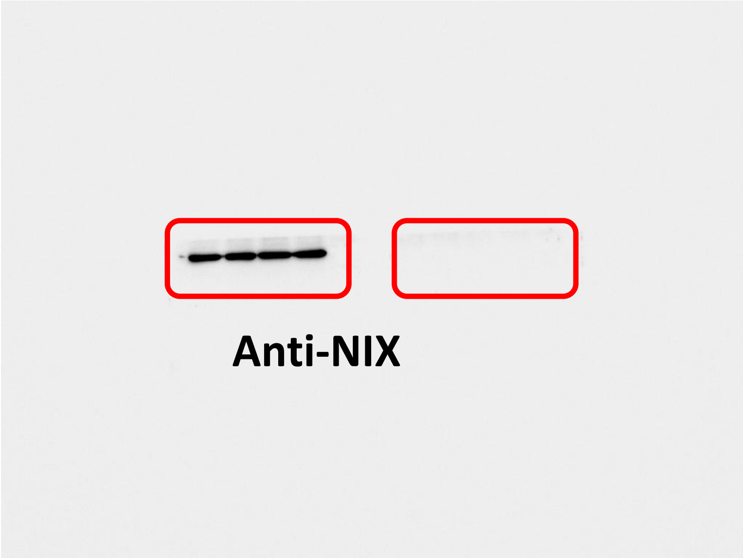
Anti-NIX

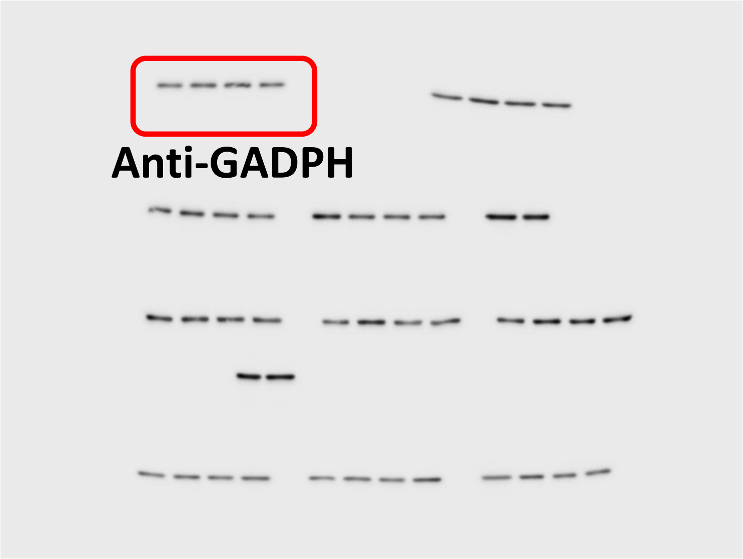
Anti-GADPH
