# Supplementary figures and images for: Ubiquitin ligase ITCH regulates life cycle of SARS-CoV-2 virus (part 3 of 4)
Source: eLife. 2026 May 29;14:RP105105. doi: 10.7554/eLife.105105 (PMC13221179; doi:10.7554/eLife.105105)

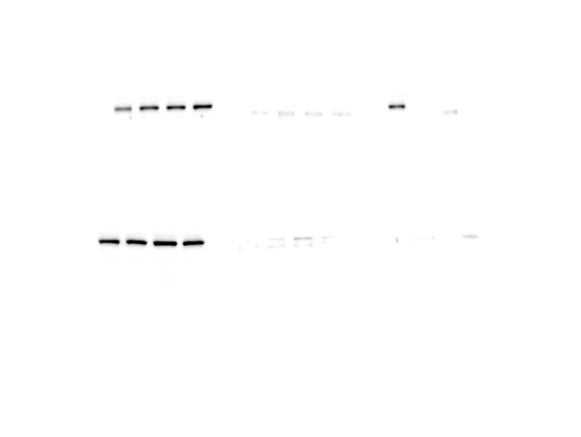

Supplement: Figure 3—figure supplement 1—source data 2. [file elife-105105-fig3-figsupp1-data2.zip › Figure 3-figure supplement 1C/p62.tif]

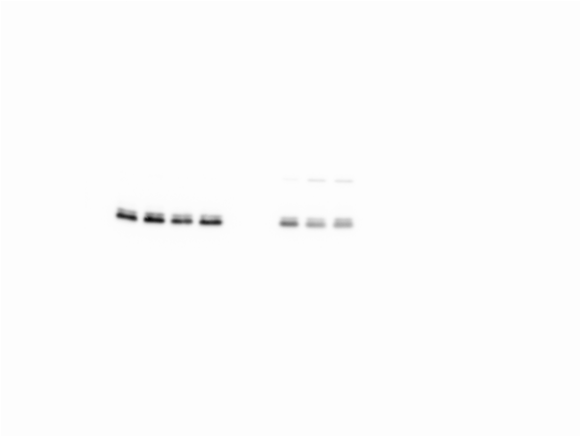

Supplement: Figure 3—figure supplement 1—source data 2. [file elife-105105-fig3-figsupp1-data2.zip › Figure 3-figure supplement 1C/RTN3.tif]

4B

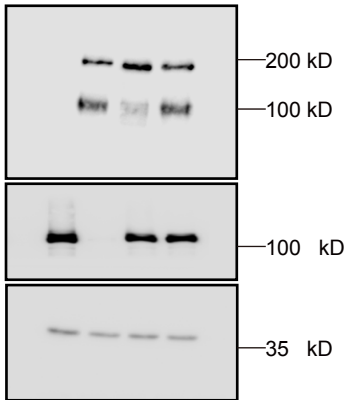

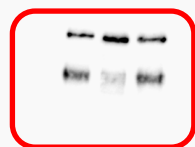

Flag spike

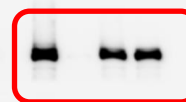

ITCH

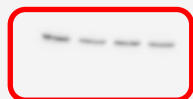

gapdh

Supplement: Figure 4—source data 1. [file elife-105105-fig4-data1.zip › Figure 4B.pdf]

4C

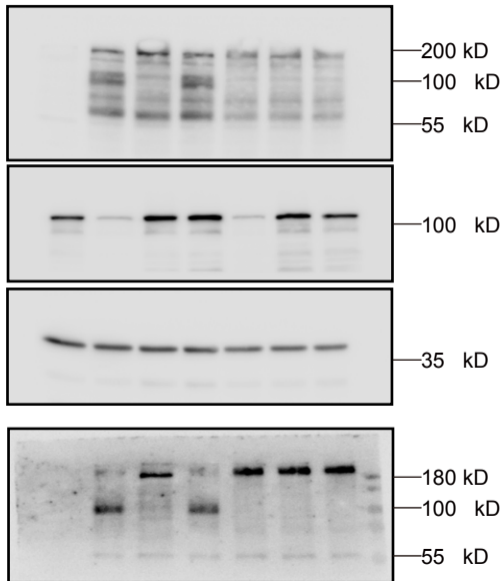

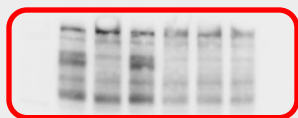

**Spike**

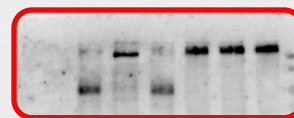

**Spike**

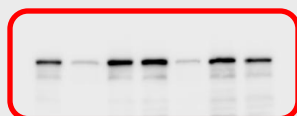

**ITCH**

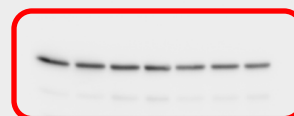

**GAPDH**

Supplement: Figure 4—source data 1. [file elife-105105-fig4-data1.zip › Figure 4C.pdf]

4A

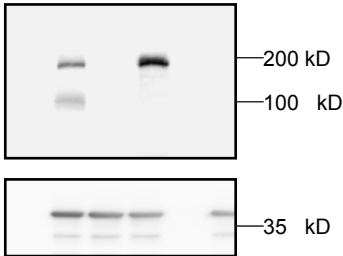

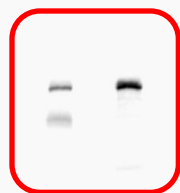

**Spike**

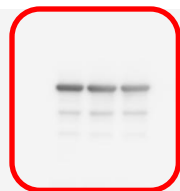

**GAPDH**

Supplement: Figure 4—source data 1. [file elife-105105-fig4-data1.zip › Figure 4A.pdf]

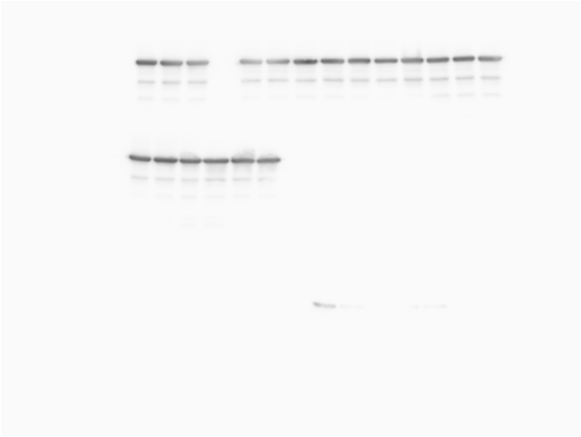

Supplement: Figure 4—source data 2. [file elife-105105-fig4-data2.zip › Figure 4A/gapdh.tif]

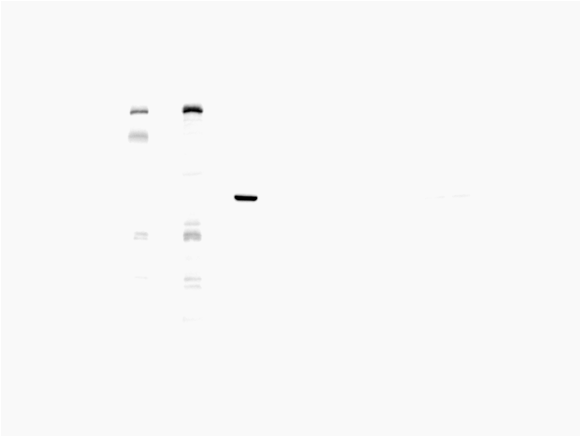

Supplement: Figure 4—source data 2. [file elife-105105-fig4-data2.zip › Figure 4A/spike.tif]

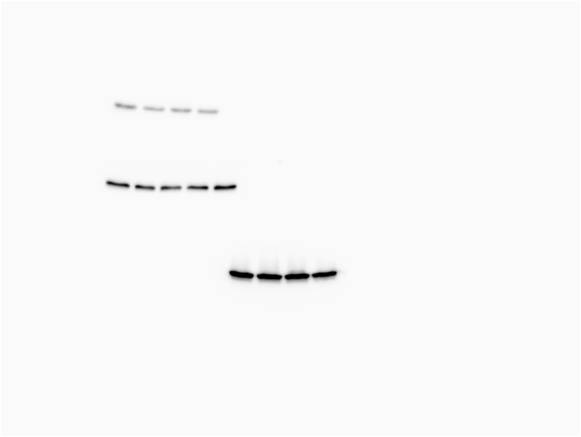

Supplement: Figure 4—source data 2. [file elife-105105-fig4-data2.zip › Figure 4B/gapdh.tif]

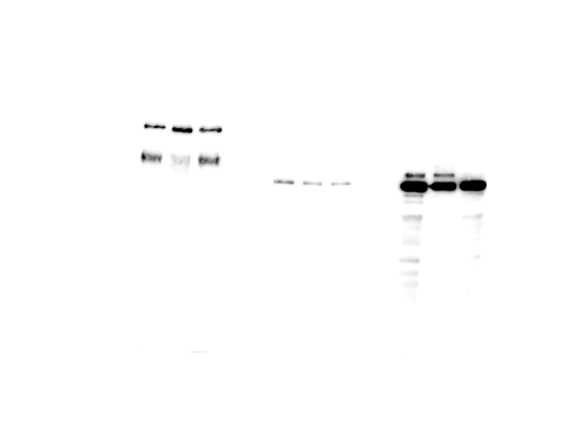

Supplement: Figure 4—source data 2. [file elife-105105-fig4-data2.zip › Figure 4B/spike.tif]

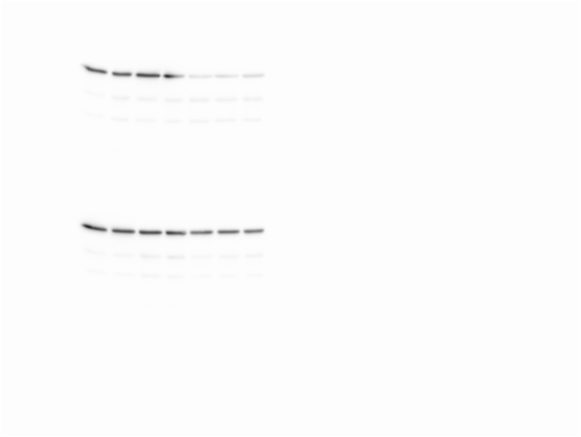

Supplement: Figure 4—source data 2. [file elife-105105-fig4-data2.zip › Figure 4C/gapdh.tif]

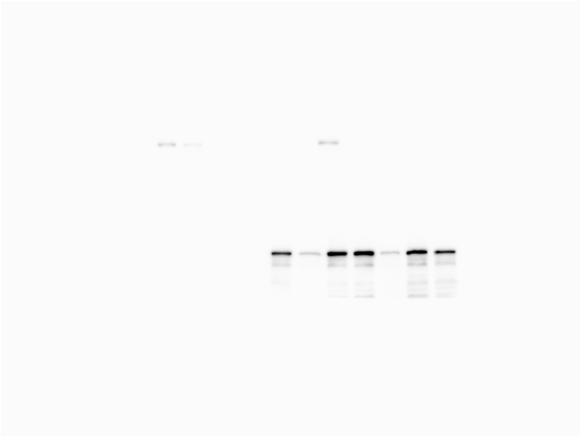

Supplement: Figure 4—source data 2. [file elife-105105-fig4-data2.zip › Figure 4C/ITCH.tif]

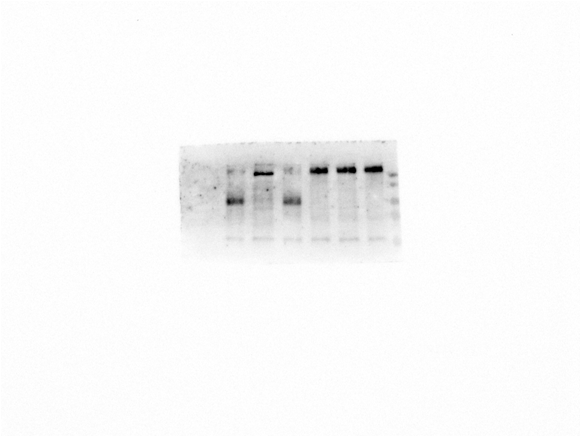

Supplement: Figure 4—source data 2. [file elife-105105-fig4-data2.zip › Figure 4C/spike medium.tif]

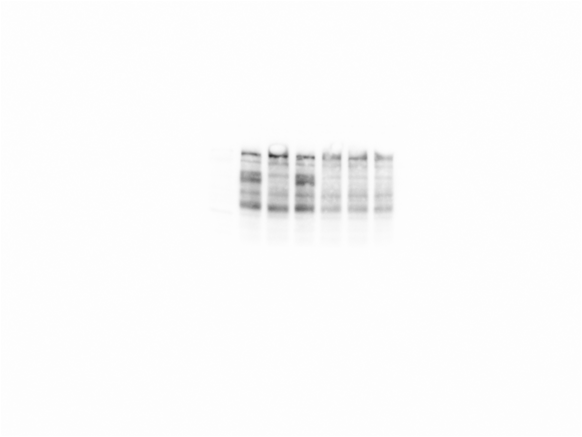

Supplement: Figure 4—source data 2. [file elife-105105-fig4-data2.zip › Figure 4C/spike.tif]

# S4A

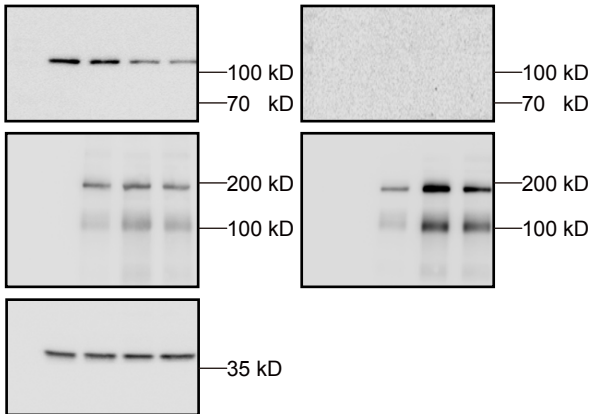

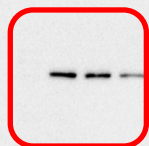

**ITCH**

**ITCH**

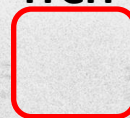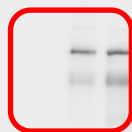

**spike**

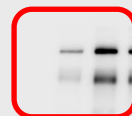

**spike**

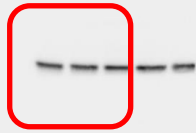

**GAPDH**

Supplement: Figure 4—figure supplement 1—source data 1. [file elife-105105-fig4-figsupp1-data1.zip › Figure 4-figure supplement 1A.pdf]

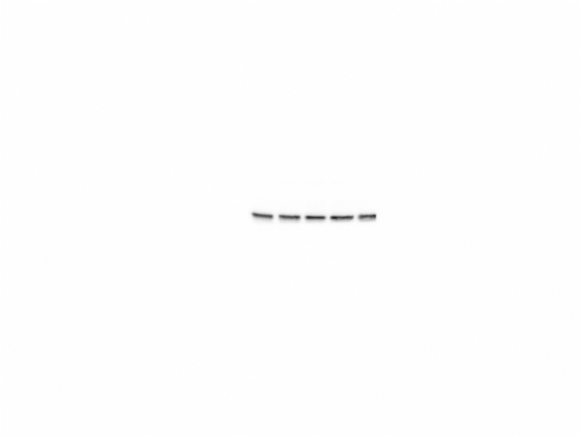

Supplement: Figure 4—figure supplement 1—source data 2. [file elife-105105-fig4-figsupp1-data2.zip › Figure 4-figure supplement 1A/gapdh.tif]

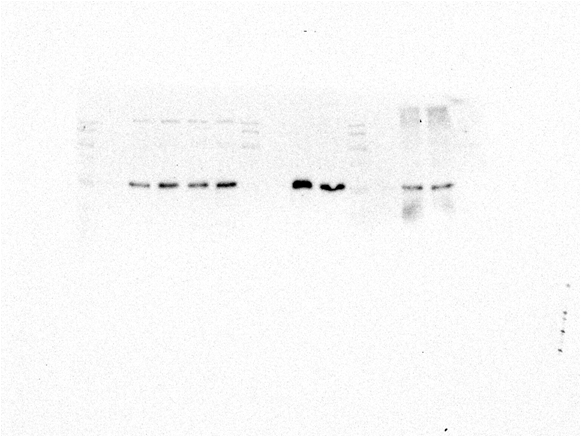

Supplement: Figure 4—figure supplement 1—source data 2. [file elife-105105-fig4-figsupp1-data2.zip › Figure 4-figure supplement 1A/itch ip.tif]

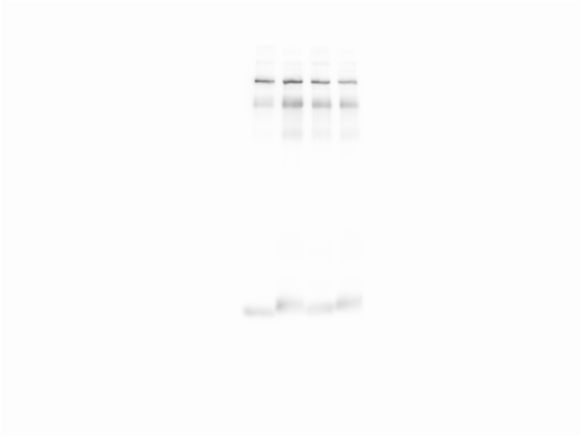

Supplement: Figure 4—figure supplement 1—source data 2. [file elife-105105-fig4-figsupp1-data2.zip › Figure 4-figure supplement 1A/spike input.tif]

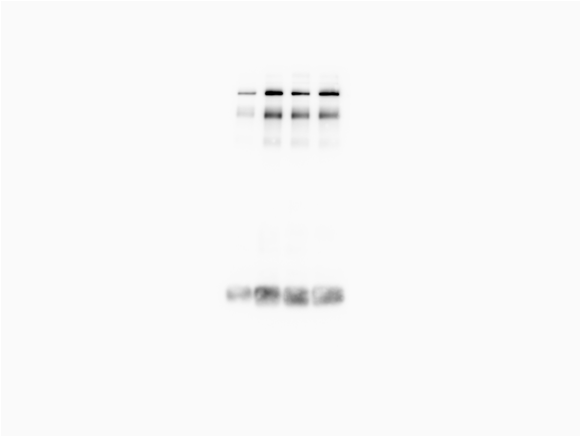

Supplement: Figure 4—figure supplement 1—source data 2. [file elife-105105-fig4-figsupp1-data2.zip › Figure 4-figure supplement 1A/spike ip.tif]

S5A

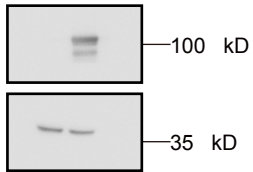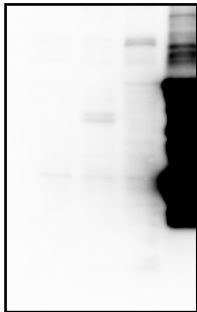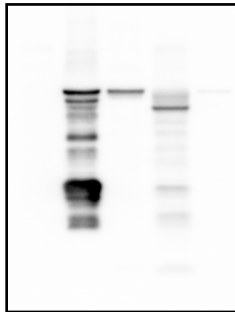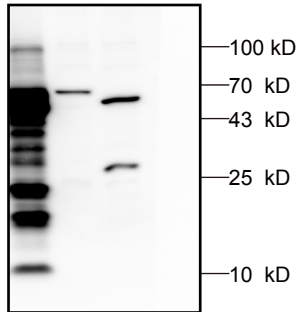

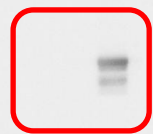

S tag

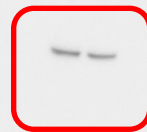

gapdh

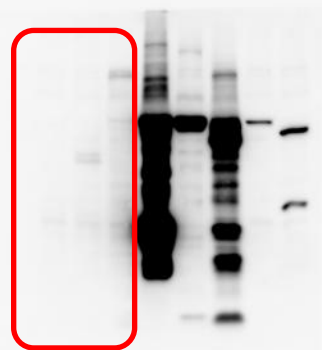

Flag

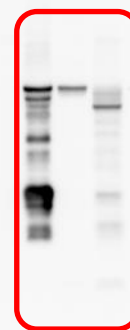

Flag

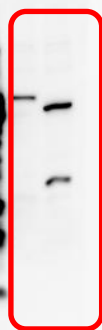

Flag

Supplement: Figure 4—figure supplement 2—source data 1. [file elife-105105-fig4-figsupp2-data1.zip › Figure 4-figure supplement 2A.pdf]

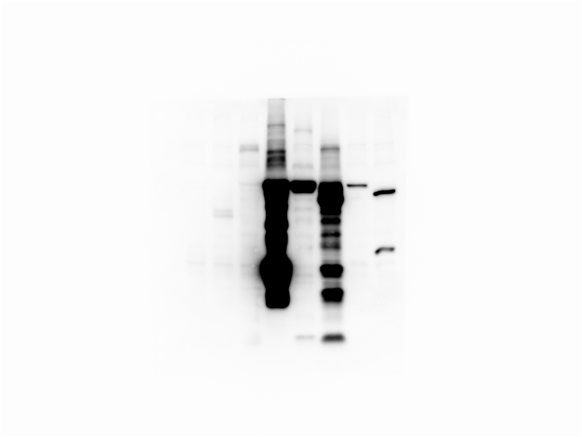

Supplement: Figure 4—figure supplement 2—source data 2. [file elife-105105-fig4-figsupp2-data2.zip › Figure 4-figure supplement 2A/flag S5A-1.tif]

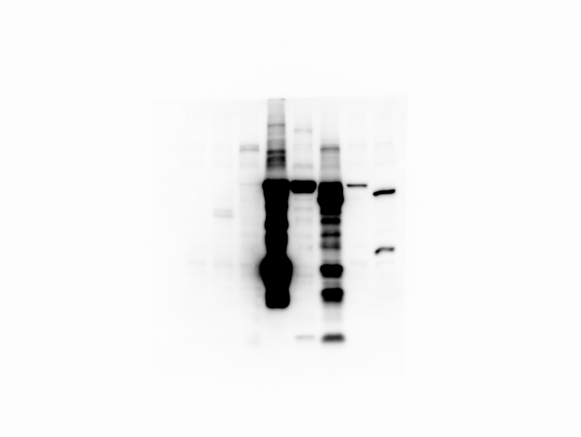

Supplement: Figure 4—figure supplement 2—source data 2. [file elife-105105-fig4-figsupp2-data2.zip › Figure 4-figure supplement 2A/flag S5A-2.tif]

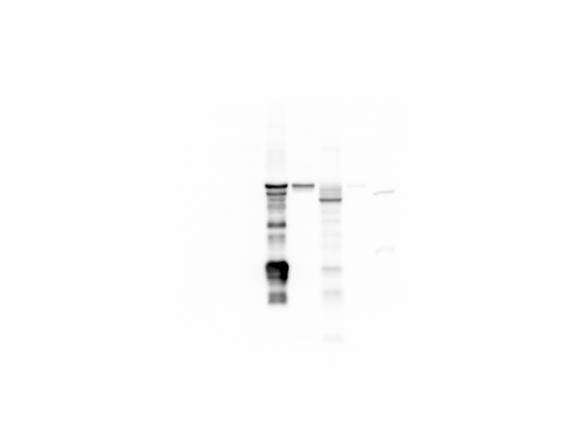

Supplement: Figure 4—figure supplement 2—source data 2. [file elife-105105-fig4-figsupp2-data2.zip › Figure 4-figure supplement 2A/flag S5A-3.tif]

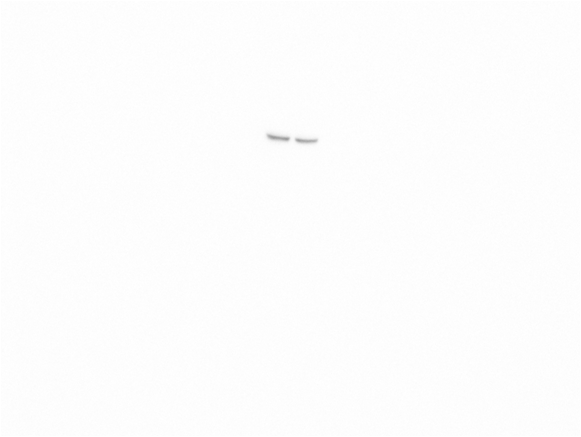

Supplement: Figure 4—figure supplement 2—source data 2. [file elife-105105-fig4-figsupp2-data2.zip › Figure 4-figure supplement 2A/gapdh.tif]

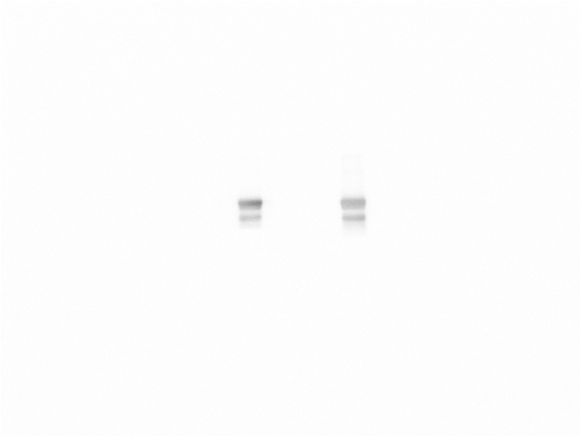

Supplement: Figure 4—figure supplement 2—source data 2. [file elife-105105-fig4-figsupp2-data2.zip › Figure 4-figure supplement 2A/S tag furin.tif]

# S6K

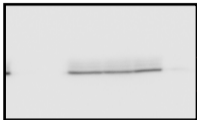

— 55 kD

— 43 kD

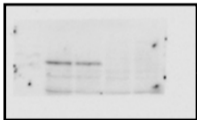

— 100 kD

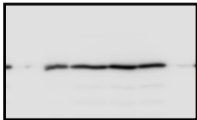

— 35 kD

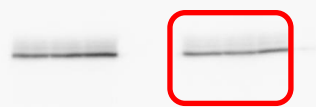

tmpr11d

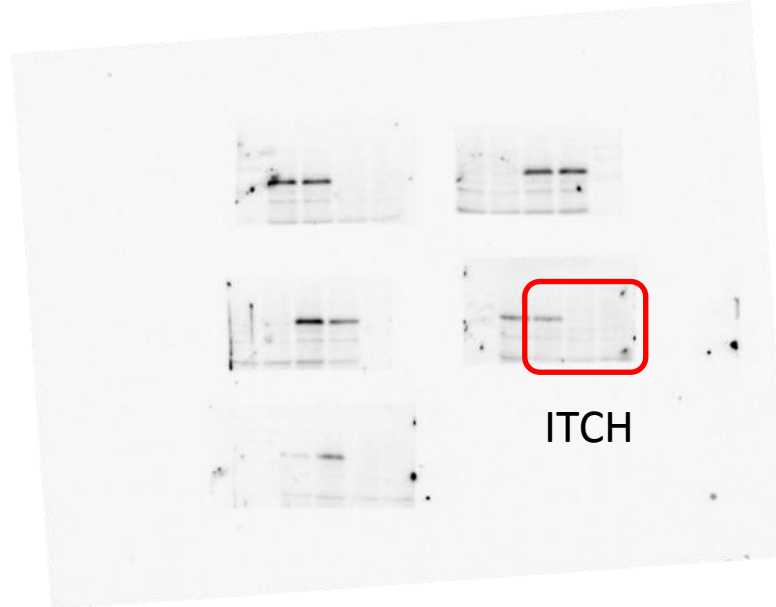

ITCH

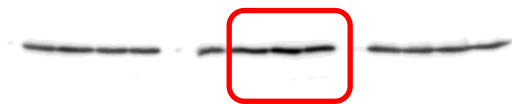

GAPDH

Supplement: Figure 4—figure supplement 3—source data 1. [file elife-105105-fig4-figsupp3-data1.zip › Figure 4-figure supplement 3K.pdf]

# S6L

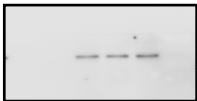

— 70 kD

— 55 kD

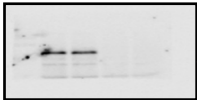

— 100 kD

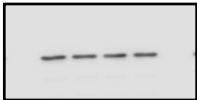

— 35 kD

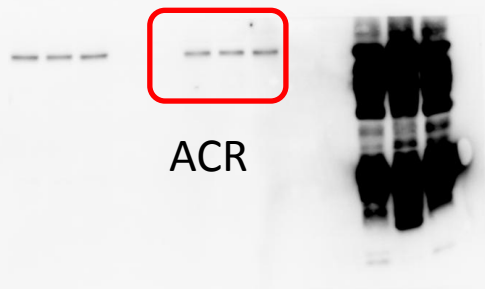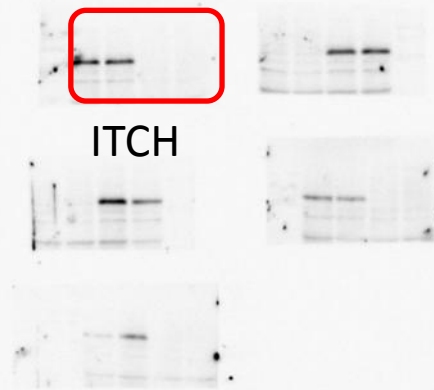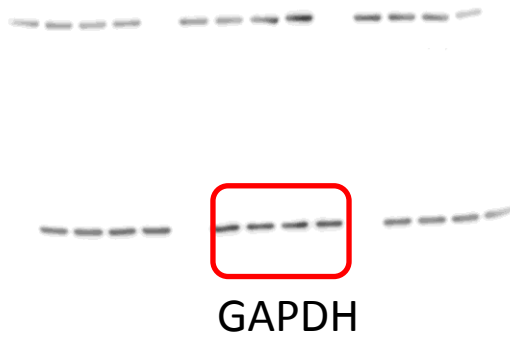

Supplement: Figure 4—figure supplement 3—source data 1. [file elife-105105-fig4-figsupp3-data1.zip › Figure 4-figure supplement 3L.pdf]

# S6M

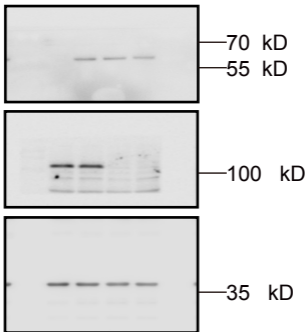

PLAT

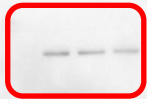

ITCH

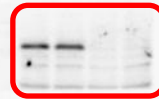

GAPDH

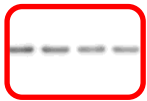

Supplement: Figure 4—figure supplement 3—source data 1. [file elife-105105-fig4-figsupp3-data1.zip › Figure 4-figure supplement 3M.pdf]

S6N

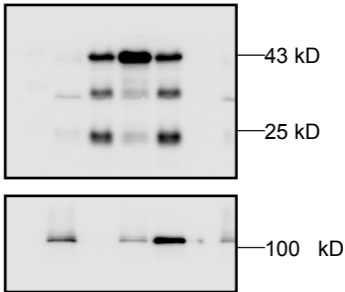

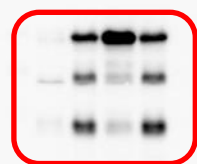

CTSL

ITCH

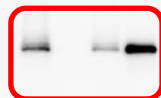

Supplement: Figure 4—figure supplement 3—source data 1. [file elife-105105-fig4-figsupp3-data1.zip › Figure 4-figure supplement 3N.pdf]

# S60

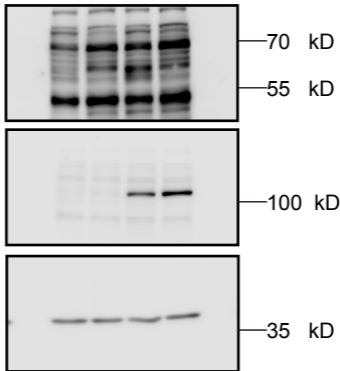

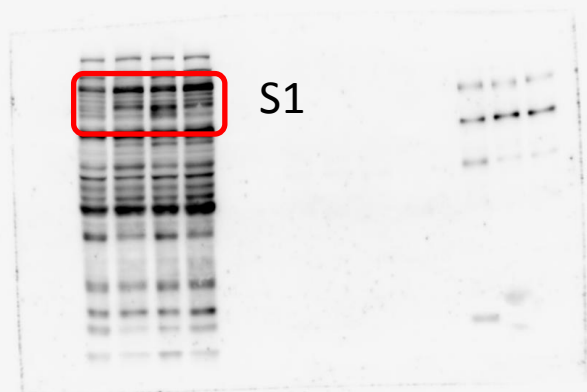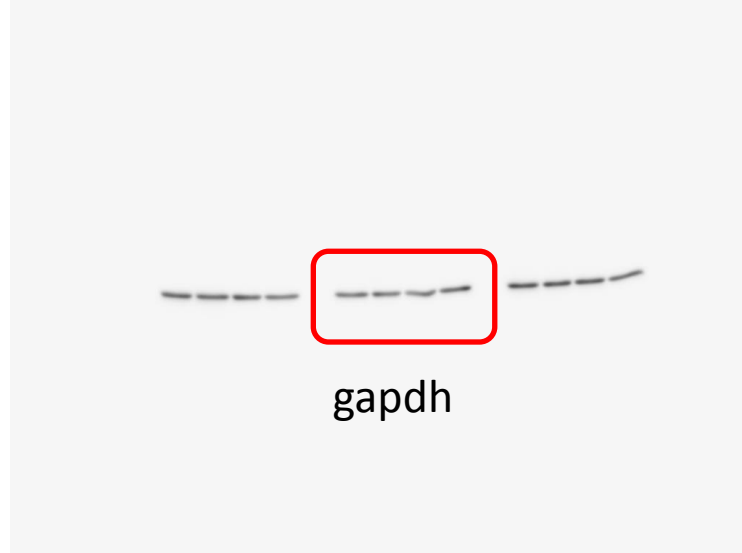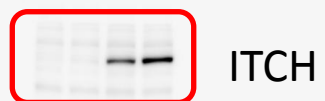

Supplement: Figure 4—figure supplement 3—source data 1. [file elife-105105-fig4-figsupp3-data1.zip › Figure 4-figure supplement 3O.pdf]

S6A

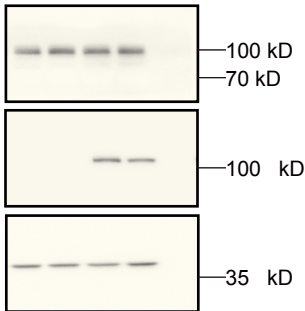

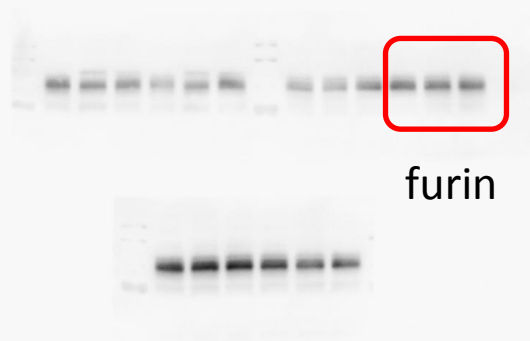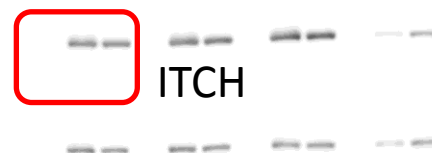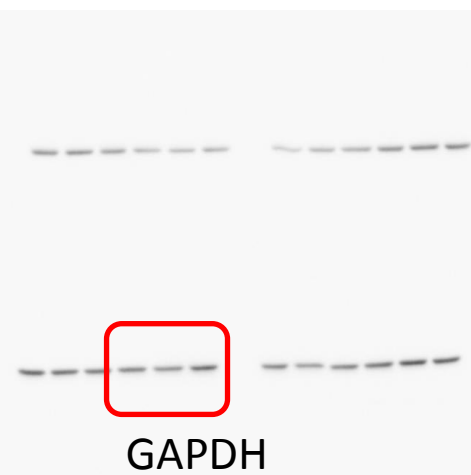

Supplement: Figure 4—figure supplement 3—source data 1. [file elife-105105-fig4-figsupp3-data1.zip › Figure 4-figure supplement 3A.pdf]

S6B

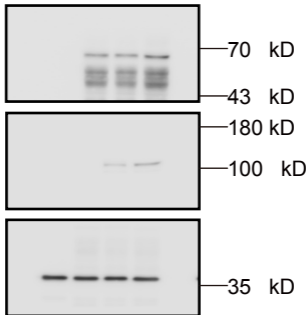

TMPRSS2

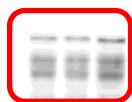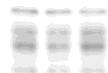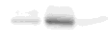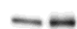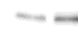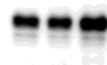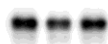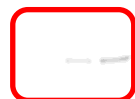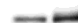

ITCH

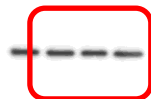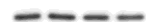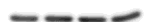

GAPDH

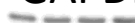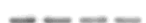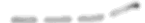

Supplement: Figure 4—figure supplement 3—source data 1. [file elife-105105-fig4-figsupp3-data1.zip › Figure 4-figure supplement 3B.pdf]

S6C

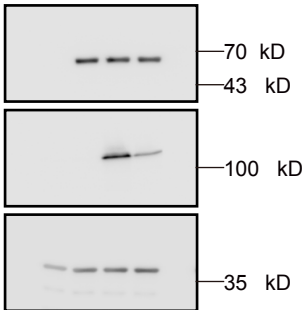

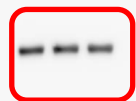

tmprss4

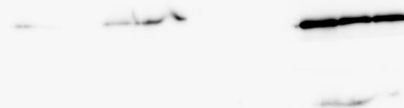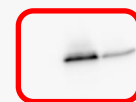

ITCH

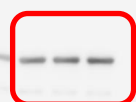

GAPDH

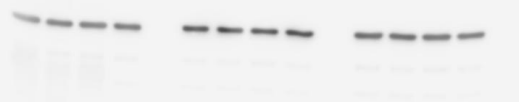

Supplement: Figure 4—figure supplement 3—source data 1. [file elife-105105-fig4-figsupp3-data1.zip › Figure 4-figure supplement 3C.pdf]

# S6D

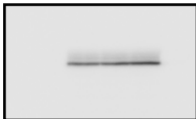

— 55 kD

— 43 kD

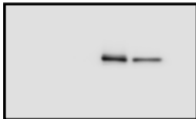

— 100 kD

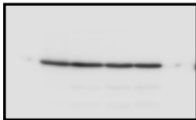

— 35 kD

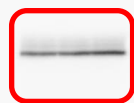

tmpr11d

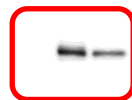

ITCH

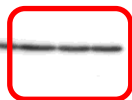

GAPDH

Supplement: Figure 4—figure supplement 3—source data 1. [file elife-105105-fig4-figsupp3-data1.zip › Figure 4-figure supplement 3D.pdf]

# S6E

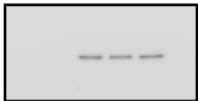

— 70 kD

— 55 kD

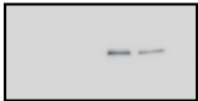

— 100 kD

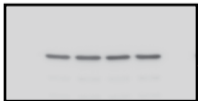

— 35 kD

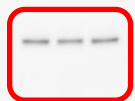

ACR

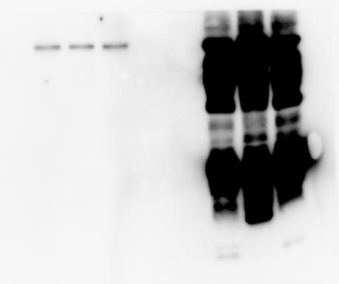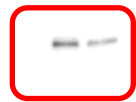

ITCH

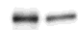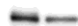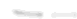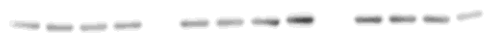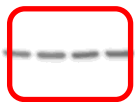

GAPDH

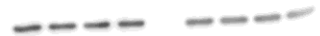

Supplement: Figure 4—figure supplement 3—source data 1. [file elife-105105-fig4-figsupp3-data1.zip › Figure 4-figure supplement 3E.pdf]

# S6F

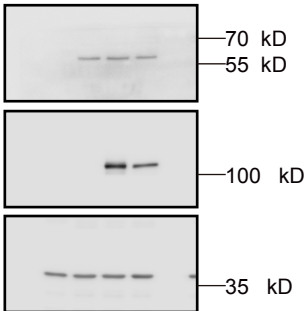

PLAT

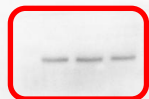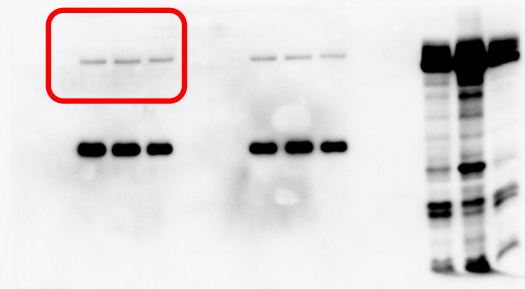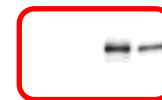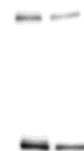

ITCH

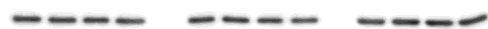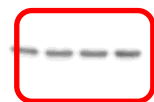

GAPDH

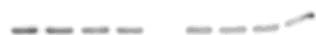

Supplement: Figure 4—figure supplement 3—source data 1. [file elife-105105-fig4-figsupp3-data1.zip › Figure 4-figure supplement 3F.pdf]

S6H

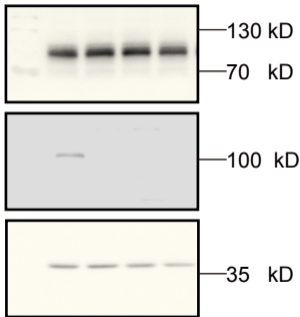

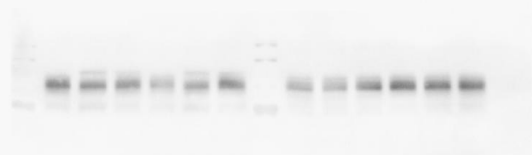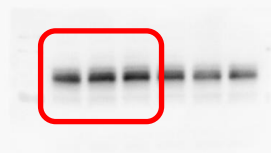

furin

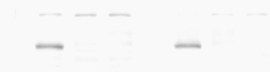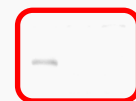

ITCH

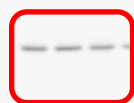

GAPDH

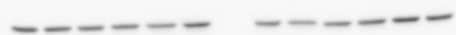

Supplement: Figure 4—figure supplement 3—source data 1. [file elife-105105-fig4-figsupp3-data1.zip › Figure 4-figure supplement 3H.pdf]

S6I

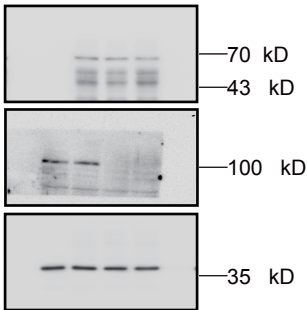

TMPRSS2

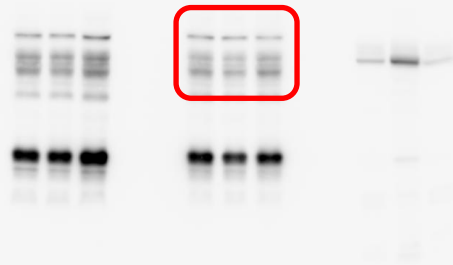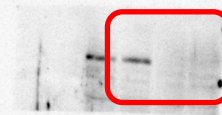

ITCH

GAPDH

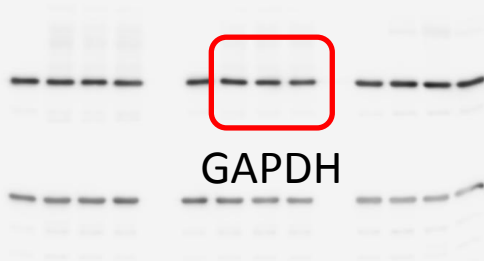

Supplement: Figure 4—figure supplement 3—source data 1. [file elife-105105-fig4-figsupp3-data1.zip › Figure 4-figure supplement 3I.pdf]

S6J

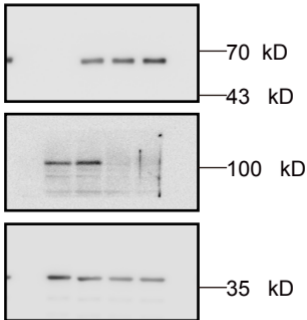

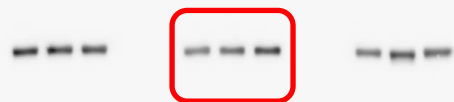

tmprss4

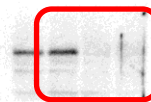

ITCH

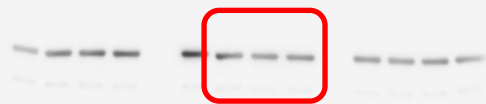

GAPDH

Supplement: Figure 4—figure supplement 3—source data 1. [file elife-105105-fig4-figsupp3-data1.zip › Figure 4-figure supplement 3J.pdf]

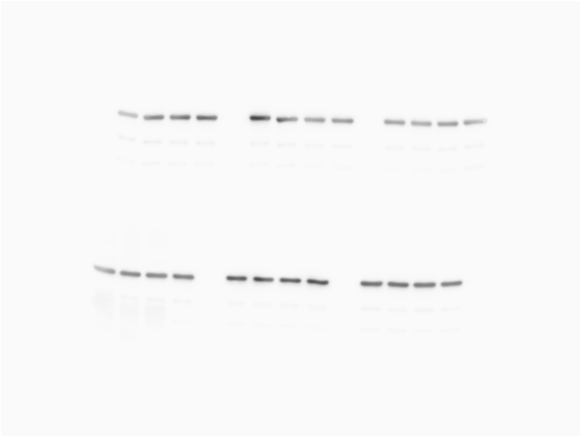

Supplement: Figure 4—figure supplement 3—source data 2. [file elife-105105-fig4-figsupp3-data2.zip › Figure 4-figure supplement 3C/gapdh.tif]

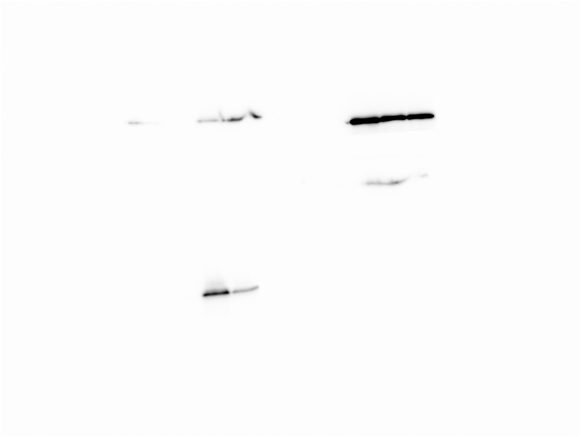

Supplement: Figure 4—figure supplement 3—source data 2. [file elife-105105-fig4-figsupp3-data2.zip › Figure 4-figure supplement 3C/itch.tif]

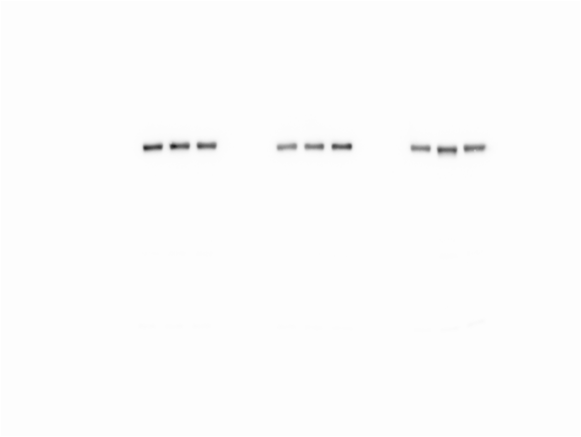

Supplement: Figure 4—figure supplement 3—source data 2. [file elife-105105-fig4-figsupp3-data2.zip › Figure 4-figure supplement 3C/tmprss4.tif]

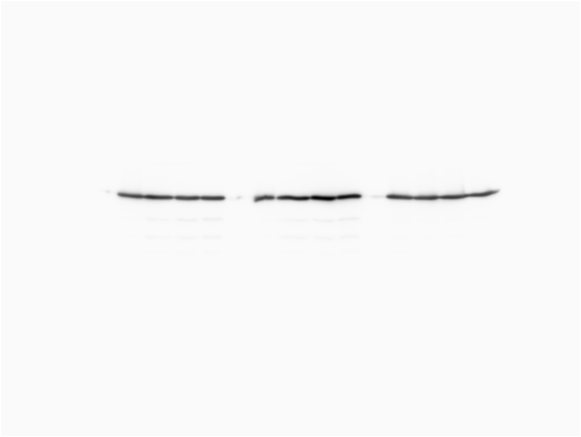

Supplement: Figure 4—figure supplement 3—source data 2. [file elife-105105-fig4-figsupp3-data2.zip › Figure 4-figure supplement 3D/gapdh.tif]

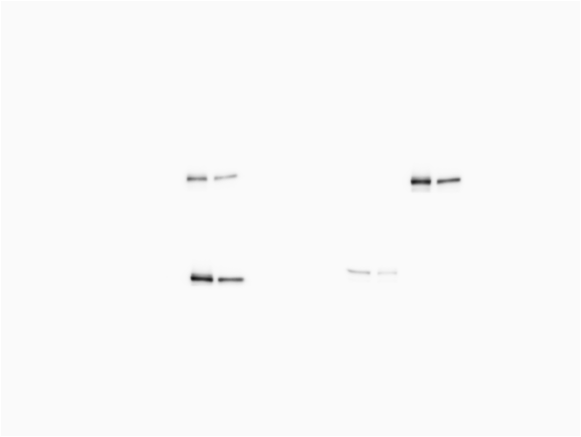

Supplement: Figure 4—figure supplement 3—source data 2. [file elife-105105-fig4-figsupp3-data2.zip › Figure 4-figure supplement 3D/itch.tif]

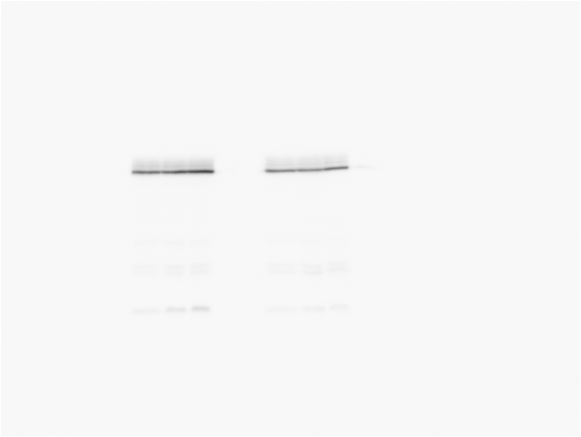

Supplement: Figure 4—figure supplement 3—source data 2. [file elife-105105-fig4-figsupp3-data2.zip › Figure 4-figure supplement 3D/tmpr11d.tif]

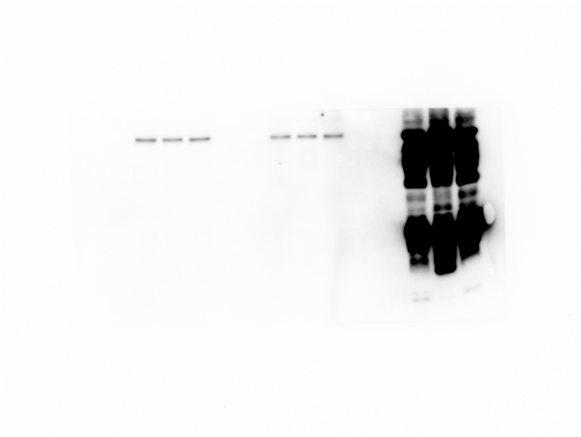

Supplement: Figure 4—figure supplement 3—source data 2. [file elife-105105-fig4-figsupp3-data2.zip › Figure 4-figure supplement 3E/ACR.tif]

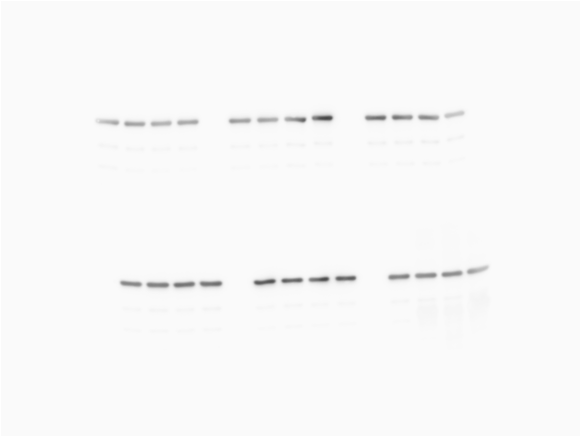

Supplement: Figure 4—figure supplement 3—source data 2. [file elife-105105-fig4-figsupp3-data2.zip › Figure 4-figure supplement 3E/gapdh.tif]

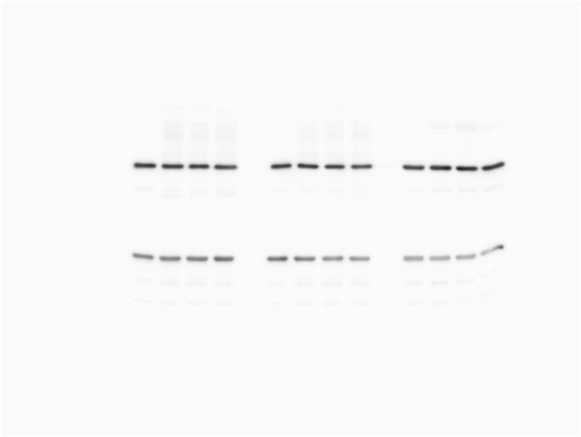

Supplement: Figure 4—figure supplement 3—source data 2. [file elife-105105-fig4-figsupp3-data2.zip › Figure 4-figure supplement 3F/gapdh.tif]

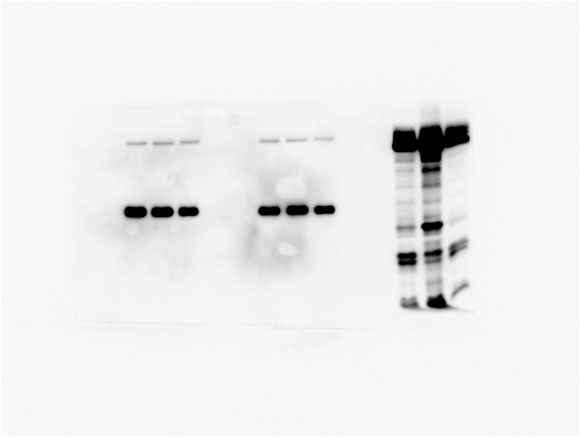

Supplement: Figure 4—figure supplement 3—source data 2. [file elife-105105-fig4-figsupp3-data2.zip › Figure 4-figure supplement 3F/plat.tif]

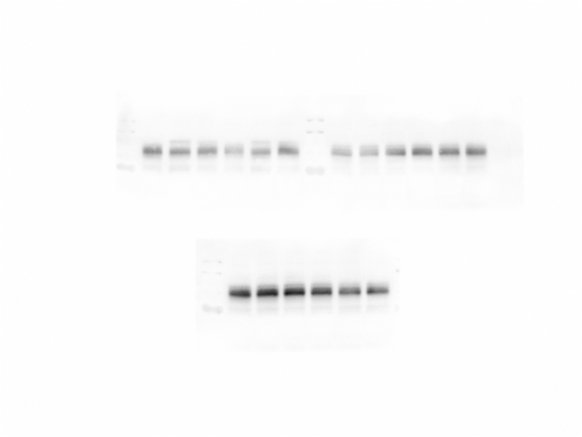

Supplement: Figure 4—figure supplement 3—source data 2. [file elife-105105-fig4-figsupp3-data2.zip › Figure 4-figure supplement 3H/furin A and H.tif]

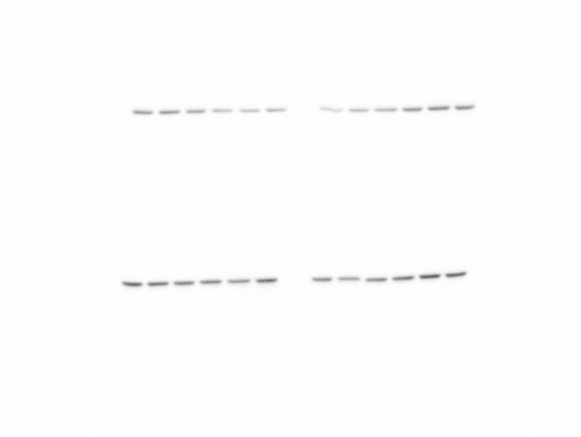

Supplement: Figure 4—figure supplement 3—source data 2. [file elife-105105-fig4-figsupp3-data2.zip › Figure 4-figure supplement 3H/gapdh.tif]

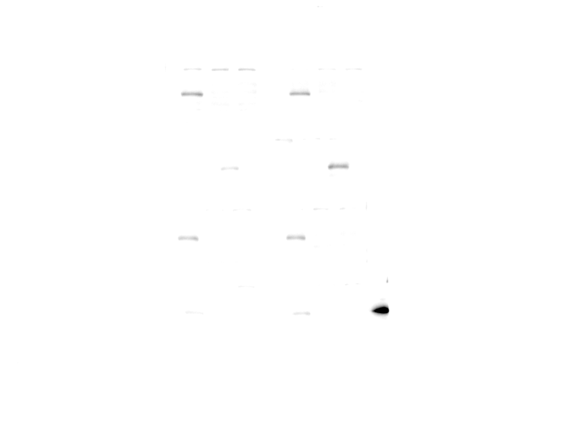

Supplement: Figure 4—figure supplement 3—source data 2. [file elife-105105-fig4-figsupp3-data2.zip › Figure 4-figure supplement 3H/itch.tif]

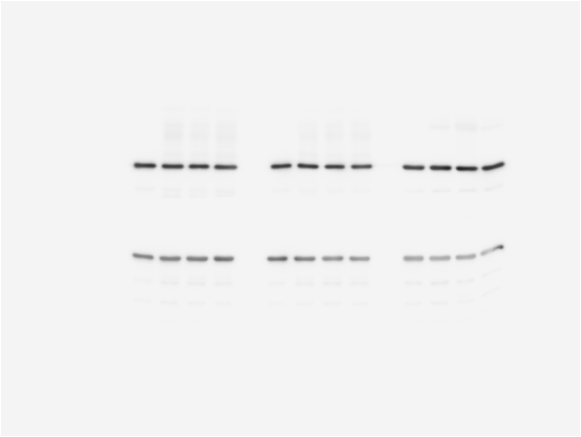

Supplement: Figure 4—figure supplement 3—source data 2. [file elife-105105-fig4-figsupp3-data2.zip › Figure 4-figure supplement 3I/gapdh.tif]

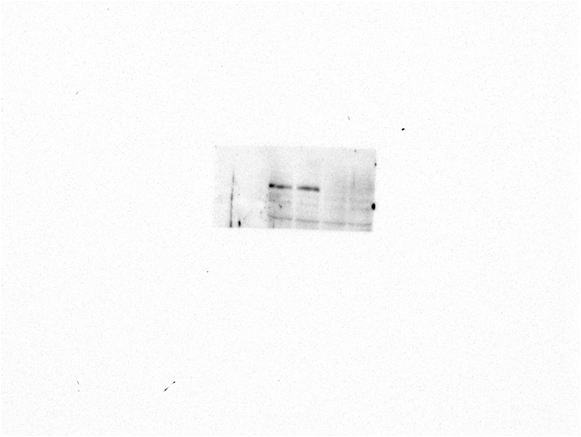

Supplement: Figure 4—figure supplement 3—source data 2. [file elife-105105-fig4-figsupp3-data2.zip › Figure 4-figure supplement 3I/itch.tif]

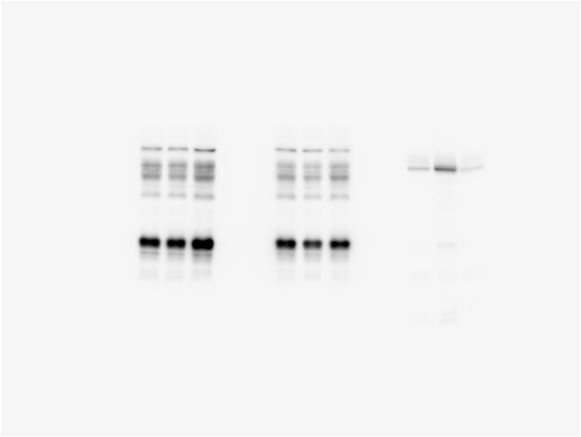

Supplement: Figure 4—figure supplement 3—source data 2. [file elife-105105-fig4-figsupp3-data2.zip › Figure 4-figure supplement 3I/tmprss2.tif]

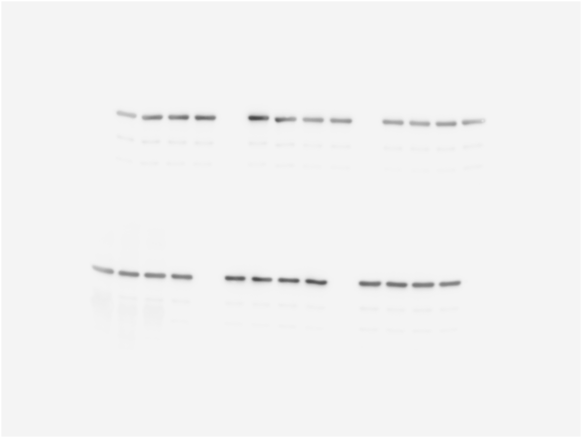

Supplement: Figure 4—figure supplement 3—source data 2. [file elife-105105-fig4-figsupp3-data2.zip › Figure 4-figure supplement 3J/gapdh.tif]

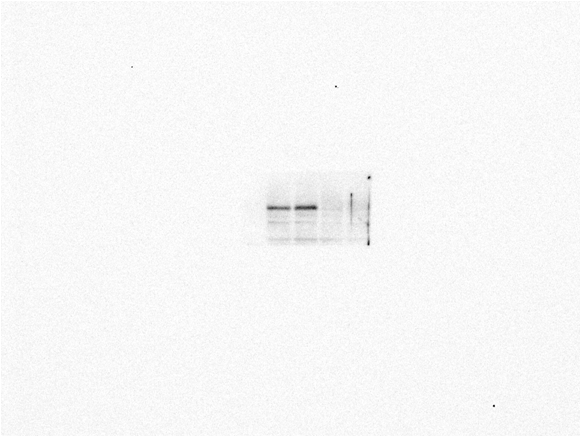

Supplement: Figure 4—figure supplement 3—source data 2. [file elife-105105-fig4-figsupp3-data2.zip › Figure 4-figure supplement 3J/itch.tif]

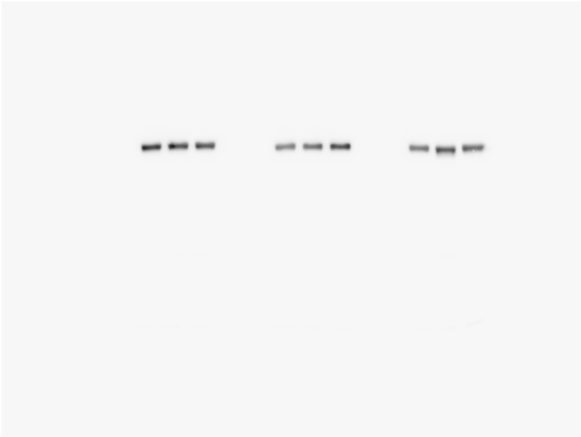

Supplement: Figure 4—figure supplement 3—source data 2. [file elife-105105-fig4-figsupp3-data2.zip › Figure 4-figure supplement 3J/tmprss4.tif]

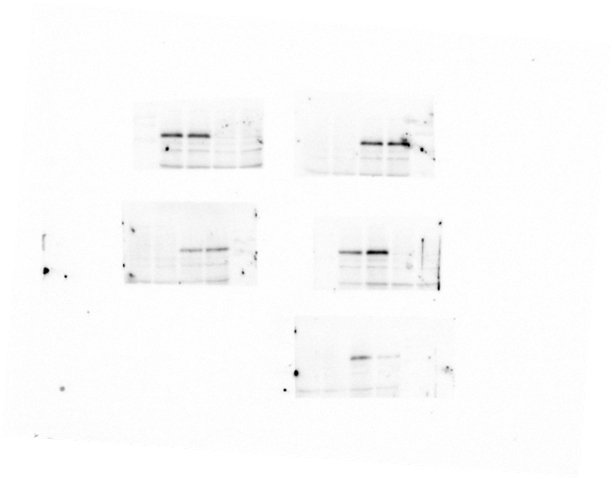

Supplement: Figure 4—figure supplement 3—source data 2. [file elife-105105-fig4-figsupp3-data2.zip › Figure 4-figure supplement 3K/itch.tif]

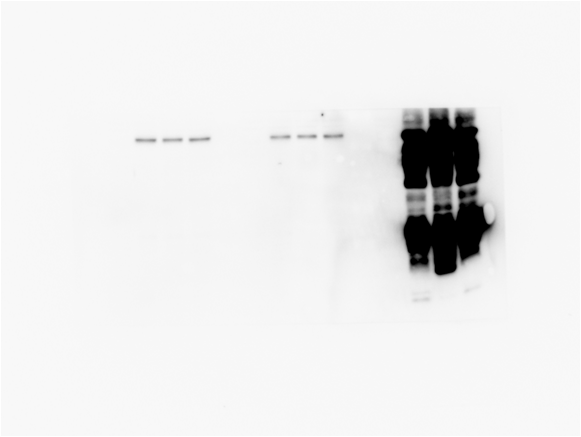

Supplement: Figure 4—figure supplement 3—source data 2. [file elife-105105-fig4-figsupp3-data2.zip › Figure 4-figure supplement 3L/ACR.tif]

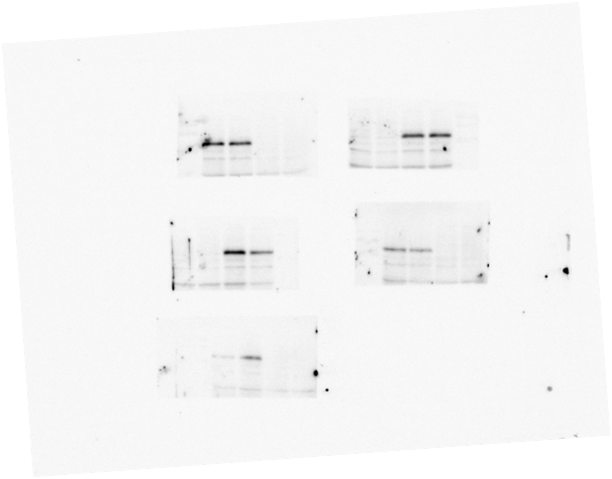

Supplement: Figure 4—figure supplement 3—source data 2. [file elife-105105-fig4-figsupp3-data2.zip › Figure 4-figure supplement 3L/itch.tif]

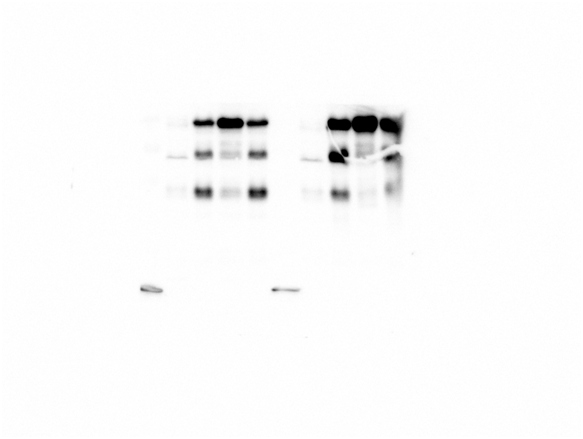

Supplement: Figure 4—figure supplement 3—source data 2. [file elife-105105-fig4-figsupp3-data2.zip › Figure 4-figure supplement 3N/CTSL.tif]

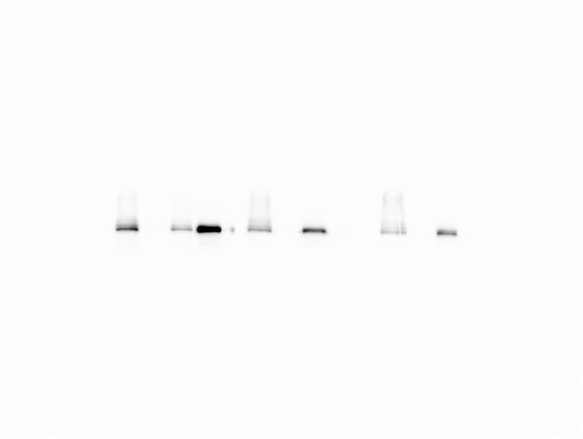

Supplement: Figure 4—figure supplement 3—source data 2. [file elife-105105-fig4-figsupp3-data2.zip › Figure 4-figure supplement 3N/itch.tif]

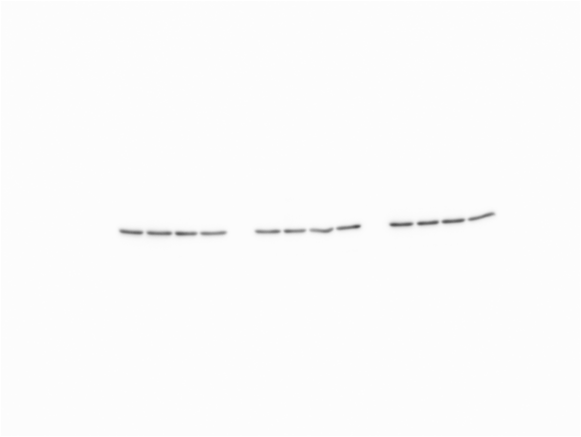

Supplement: Figure 4—figure supplement 3—source data 2. [file elife-105105-fig4-figsupp3-data2.zip › Figure 4-figure supplement 3O/gapdh.tif]

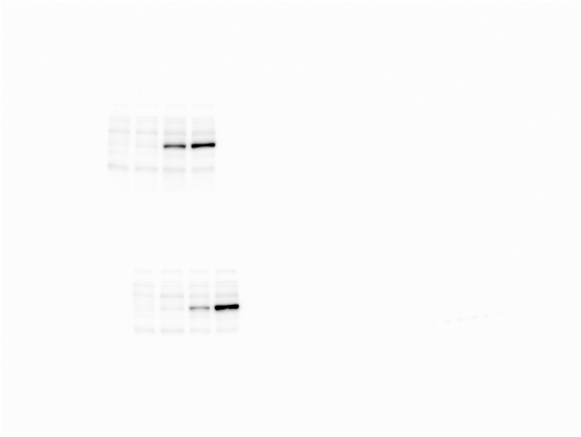

Supplement: Figure 4—figure supplement 3—source data 2. [file elife-105105-fig4-figsupp3-data2.zip › Figure 4-figure supplement 3O/itch.tif]

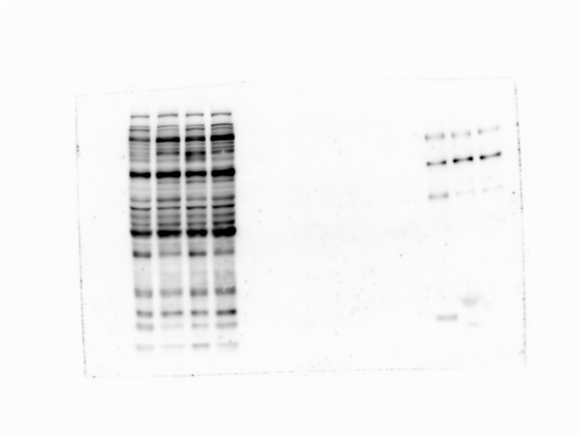

Supplement: Figure 4—figure supplement 3—source data 2. [file elife-105105-fig4-figsupp3-data2.zip › Figure 4-figure supplement 3O/S1.tif]

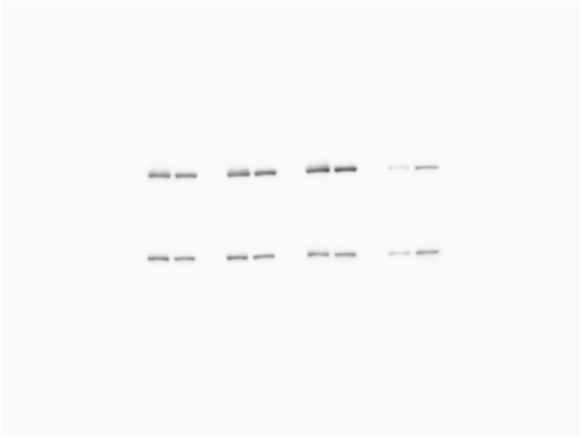

Supplement: Figure 4—figure supplement 3—source data 2. [file elife-105105-fig4-figsupp3-data2.zip › Figure 4-figure supplement 3A/ITCH.tif]

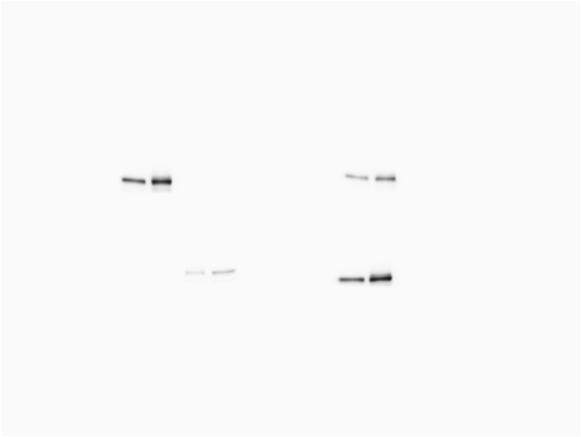

Supplement: Figure 4—figure supplement 3—source data 2. [file elife-105105-fig4-figsupp3-data2.zip › Figure 4-figure supplement 3B/itch.tif]

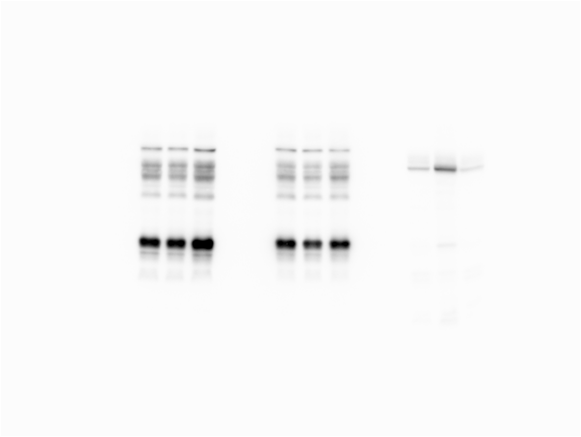

Supplement: Figure 4—figure supplement 3—source data 2. [file elife-105105-fig4-figsupp3-data2.zip › Figure 4-figure supplement 3B/TMPRSS2.tif]

5F

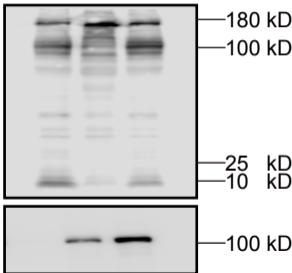

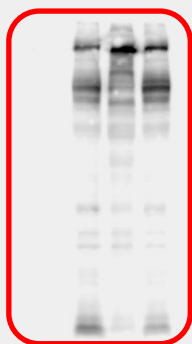

Spike

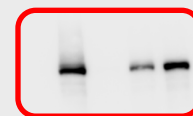

ITCH

Supplement: Figure 5—source data 1. [file elife-105105-fig5-data1.zip › Figure 5F.pdf]

5G

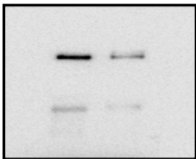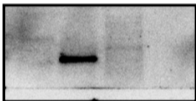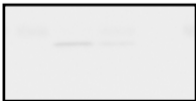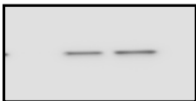

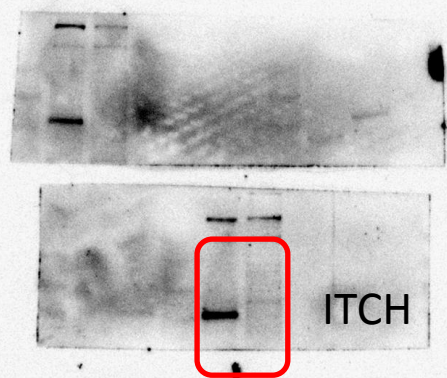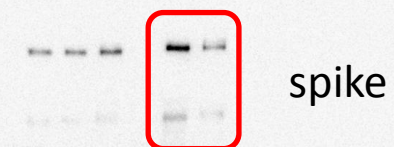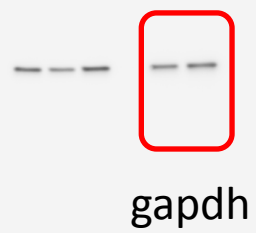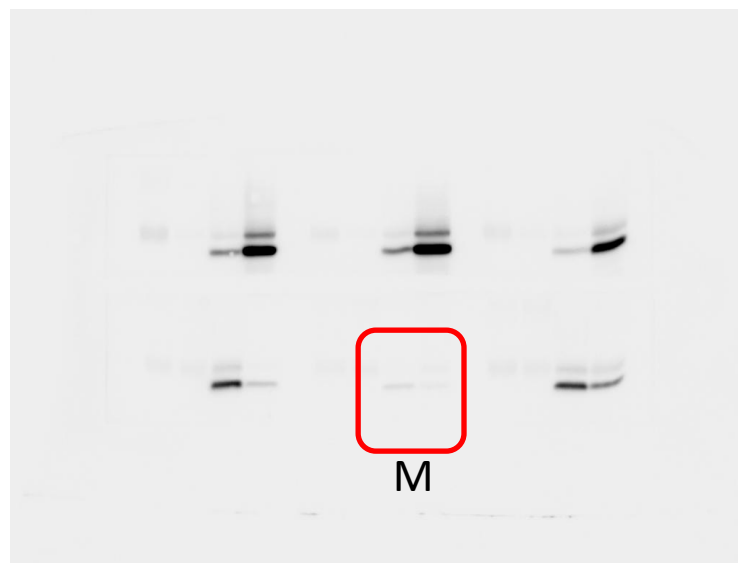

Supplement: Figure 5—source data 1. [file elife-105105-fig5-data1.zip › Figure 5G.pdf]

5B

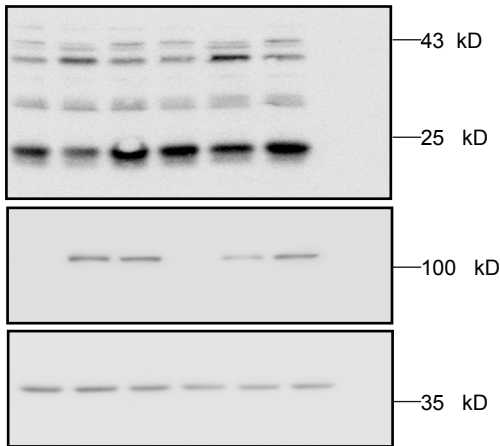

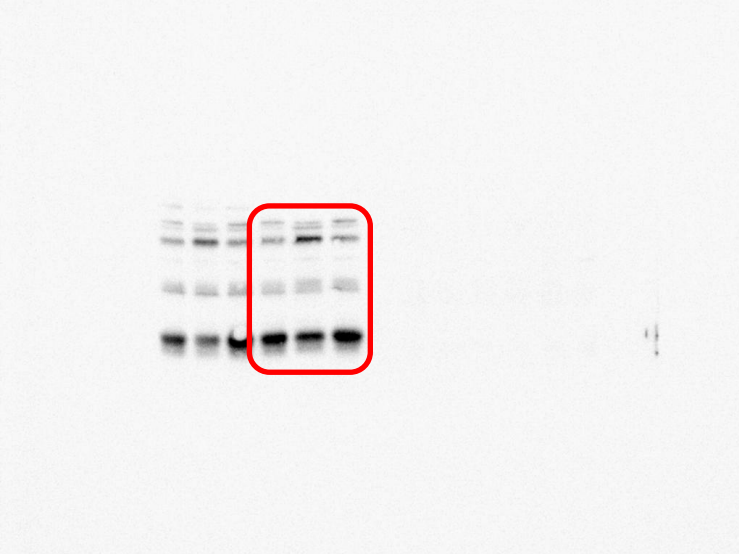

CTSL

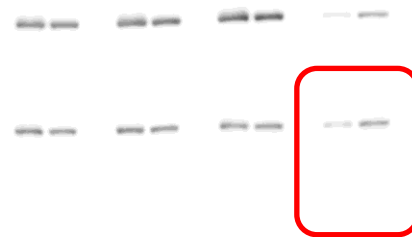

ITCH

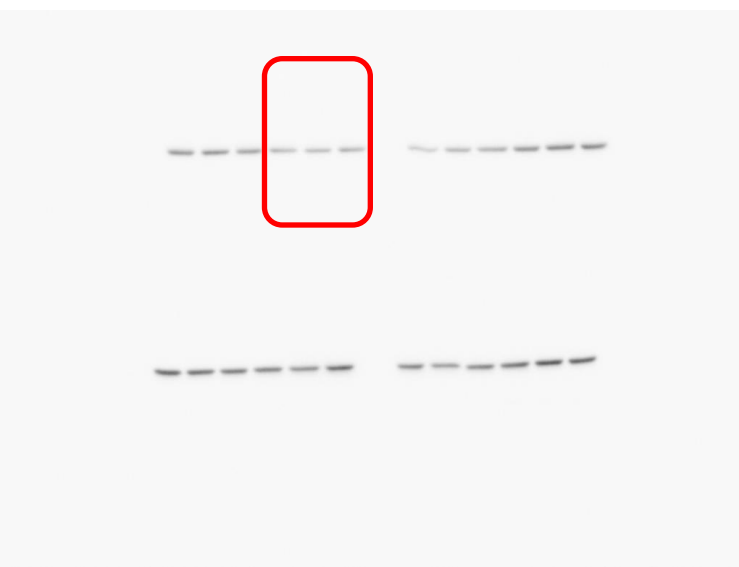

GAPDH

Supplement: Figure 5—source data 1. [file elife-105105-fig5-data1.zip › Figure 5B.pdf]

5C

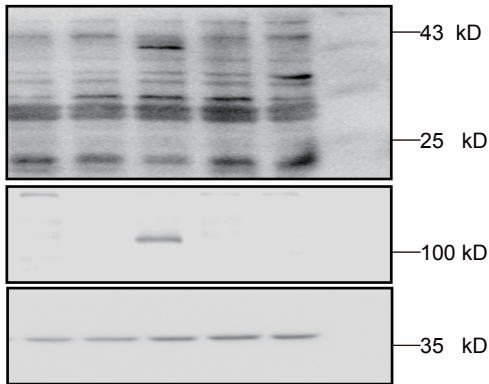

CTSL

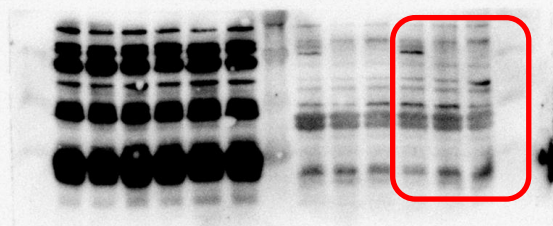

ITCH

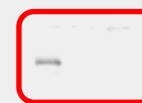

gapdh

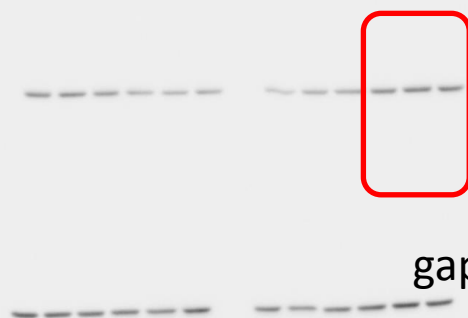

Supplement: Figure 5—source data 1. [file elife-105105-fig5-data1.zip › Figure 5C.pdf]

5D

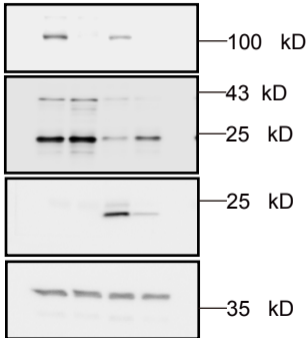

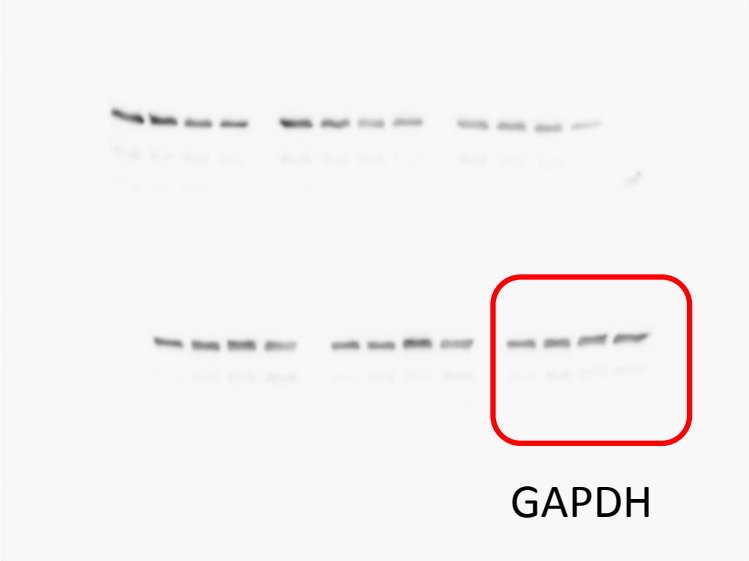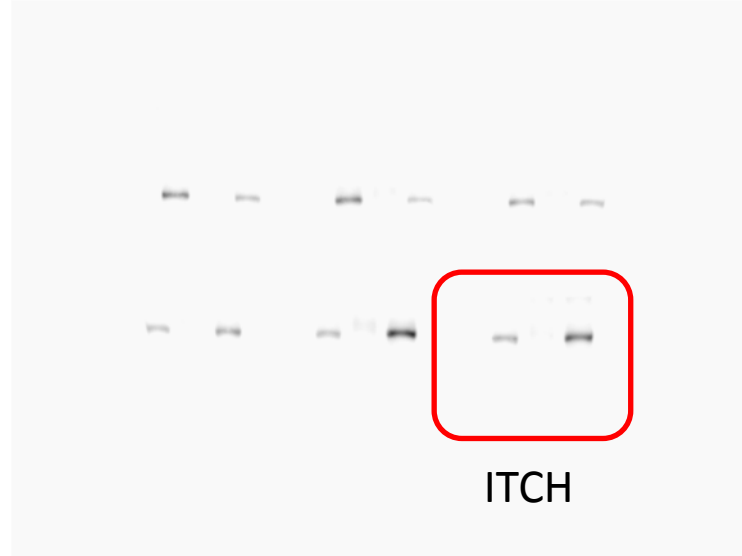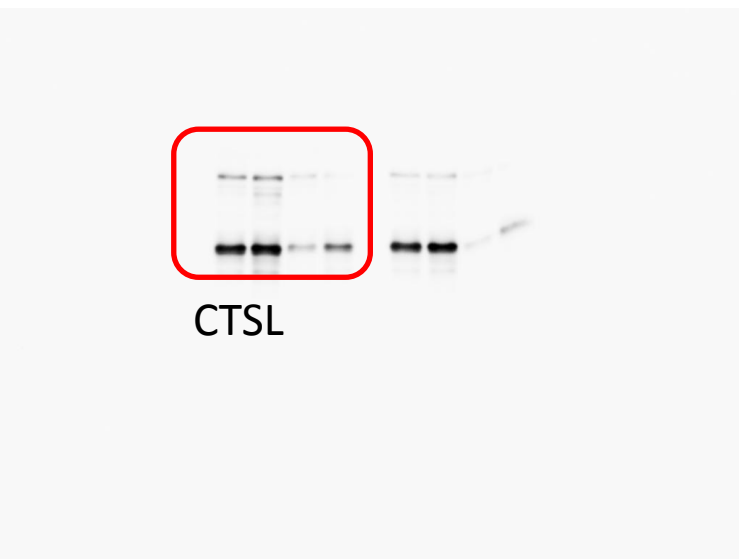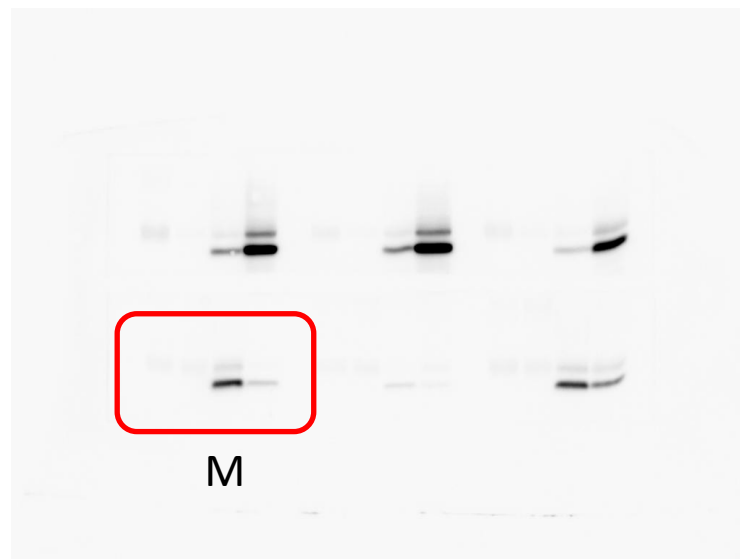

Supplement: Figure 5—source data 1. [file elife-105105-fig5-data1.zip › Figure 5D.pdf]

5E

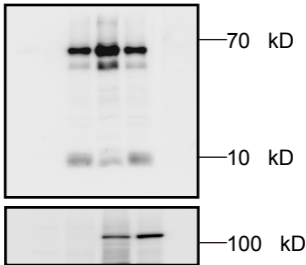

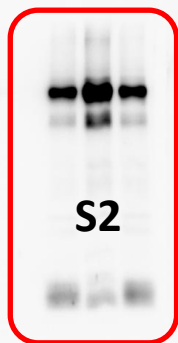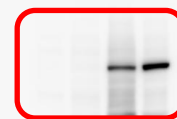

Supplement: Figure 5—source data 1. [file elife-105105-fig5-data1.zip › Figure 5E.pdf]

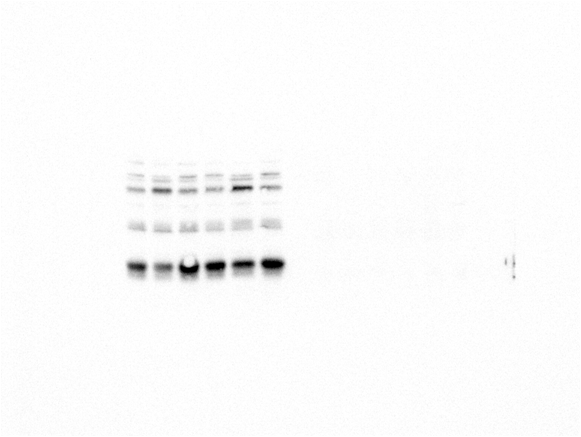

Supplement: Figure 5—source data 2. [file elife-105105-fig5-data2.zip › Figure 5B/CTSL.tif]

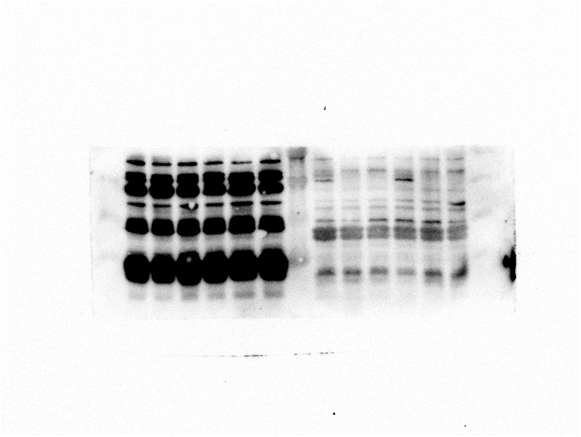

Supplement: Figure 5—source data 2. [file elife-105105-fig5-data2.zip › Figure 5C/ctsl.tif]

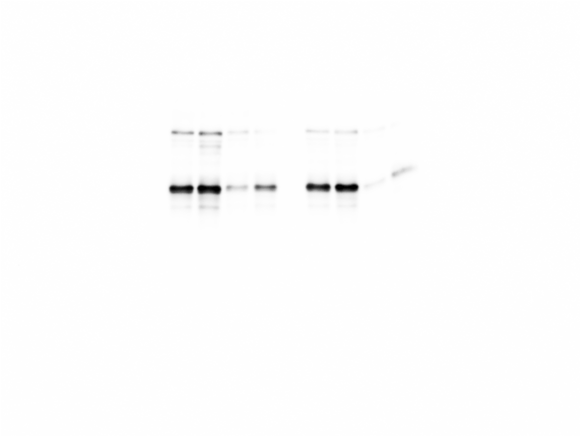

Supplement: Figure 5—source data 2. [file elife-105105-fig5-data2.zip › Figure 5D/CTSL.tif]

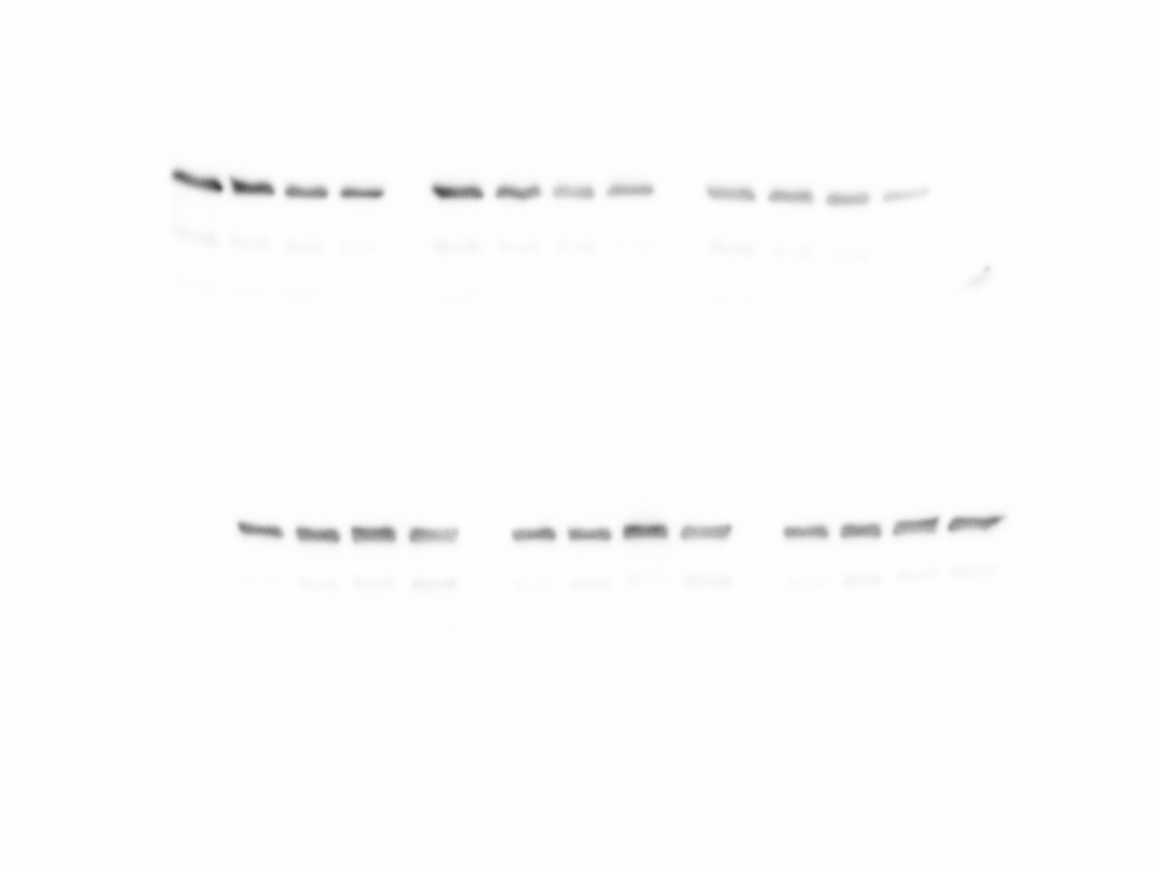

Supplement: Figure 5—source data 2. [file elife-105105-fig5-data2.zip › Figure 5D/GAPDH 5D.tif]

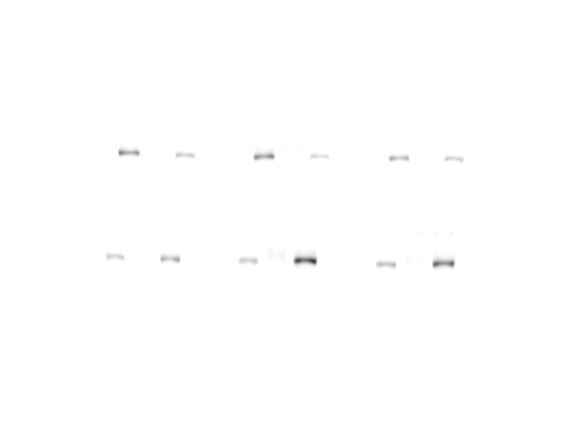

Supplement: Figure 5—source data 2. [file elife-105105-fig5-data2.zip › Figure 5D/ITCH 5D.tif]

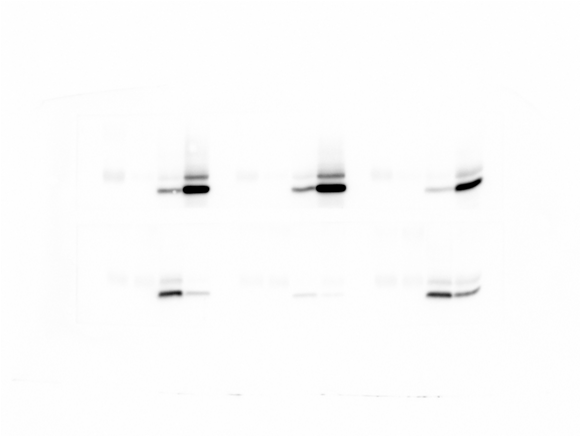

Supplement: Figure 5—source data 2. [file elife-105105-fig5-data2.zip › Figure 5D/M 5D.tif]

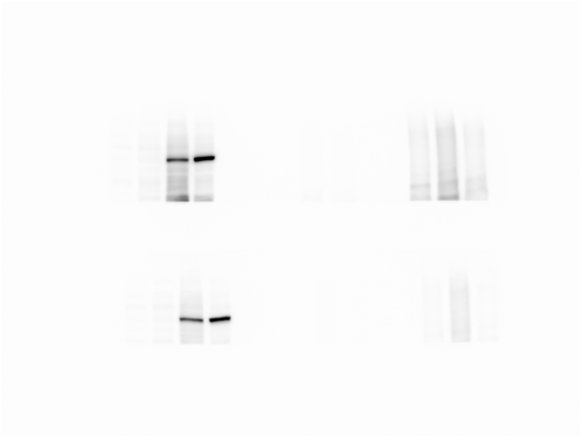

Supplement: Figure 5—source data 2. [file elife-105105-fig5-data2.zip › Figure 5E/ITCH.tif]

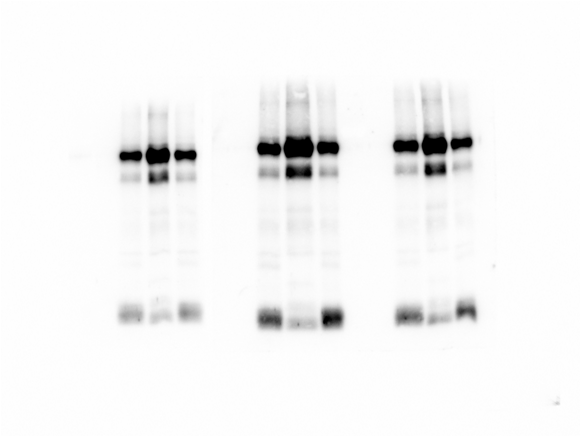

Supplement: Figure 5—source data 2. [file elife-105105-fig5-data2.zip › Figure 5E/S2.tif]

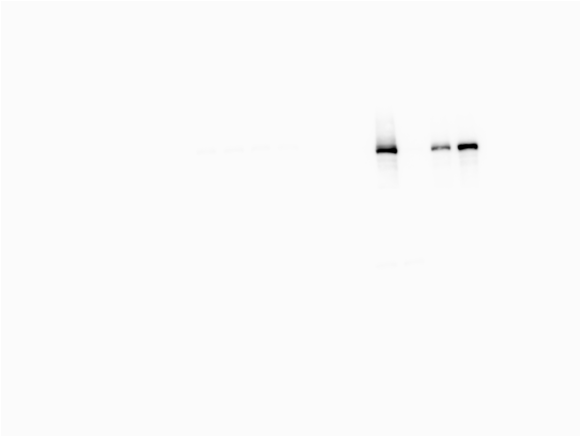

Supplement: Figure 5—source data 2. [file elife-105105-fig5-data2.zip › Figure 5F/itch 5F.tif]

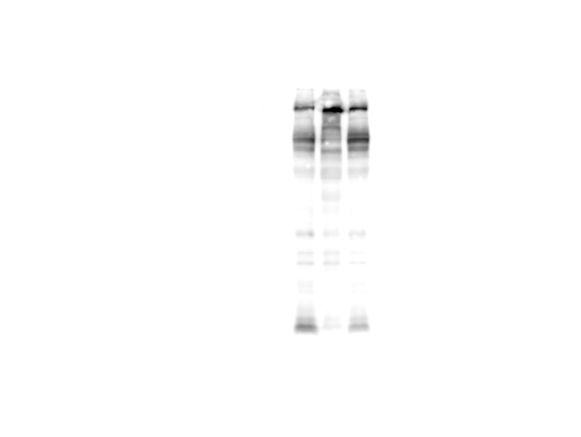

Supplement: Figure 5—source data 2. [file elife-105105-fig5-data2.zip › Figure 5F/spike 5F.tif]

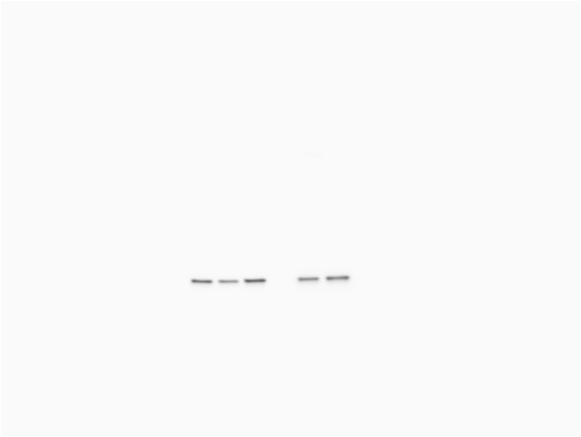

Supplement: Figure 5—source data 2. [file elife-105105-fig5-data2.zip › Figure 5G/gapdh.tif]

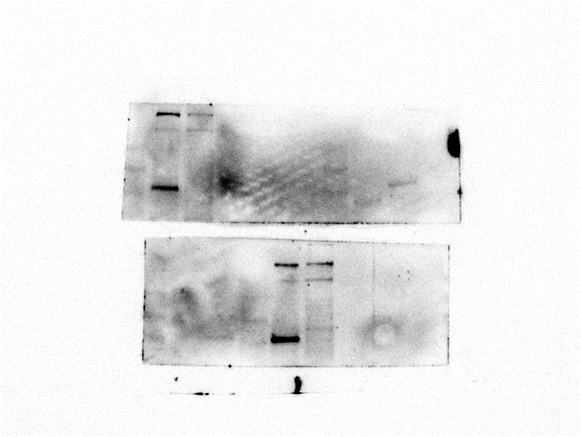

Supplement: Figure 5—source data 2. [file elife-105105-fig5-data2.zip › Figure 5G/itch.tif]

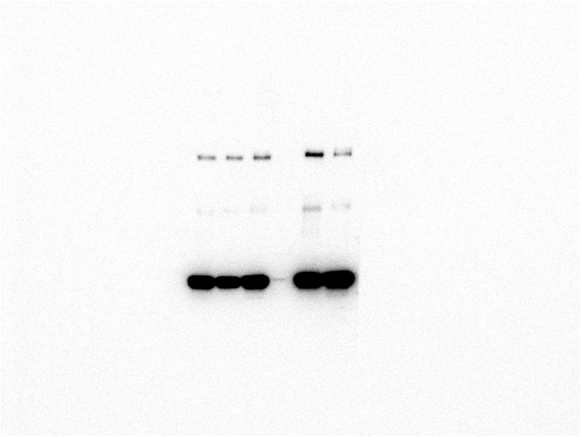

Supplement: Figure 5—source data 2. [file elife-105105-fig5-data2.zip › Figure 5G/spike.tif]

6L

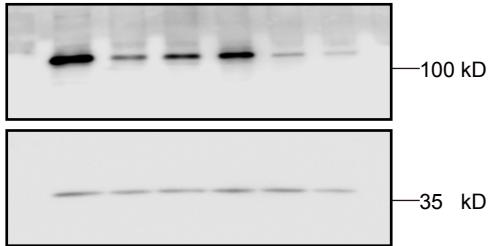

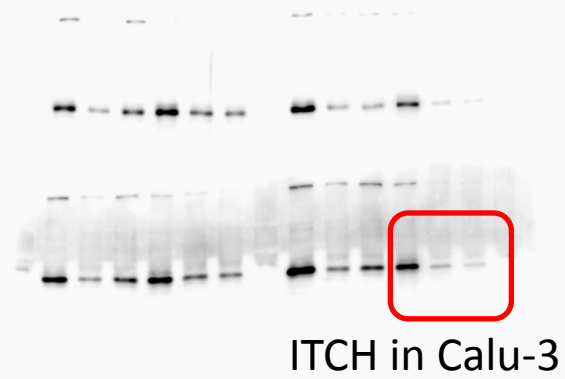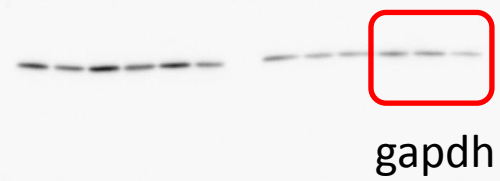

Supplement: Figure 6—source data 1. [file elife-105105-fig6-data1.zip › Figure 6L.pdf]

6C

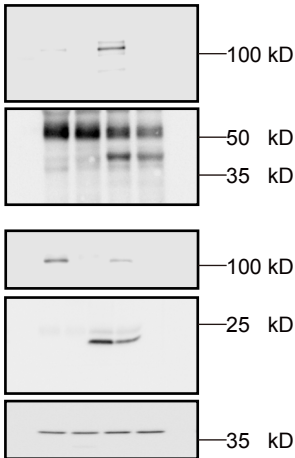

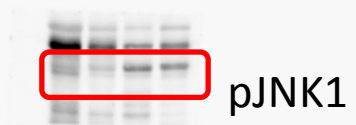

pJNK1

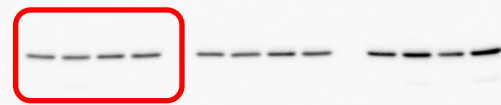

GAPDH

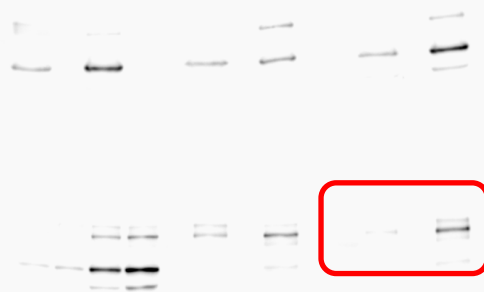

p-ITCH

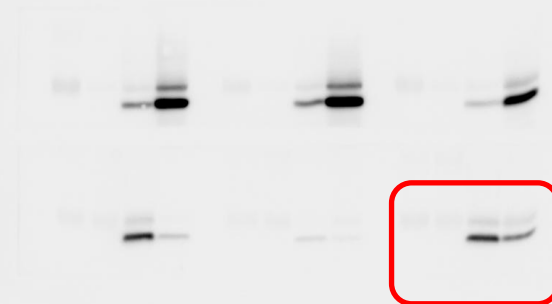

M

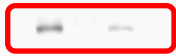

ITCH

Supplement: Figure 6—source data 1. [file elife-105105-fig6-data1.zip › Figure 6C.pdf]

6F

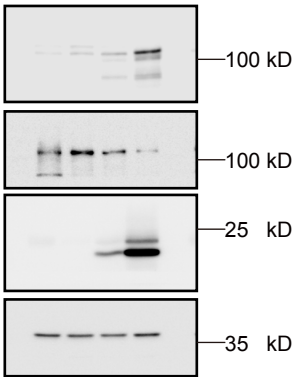

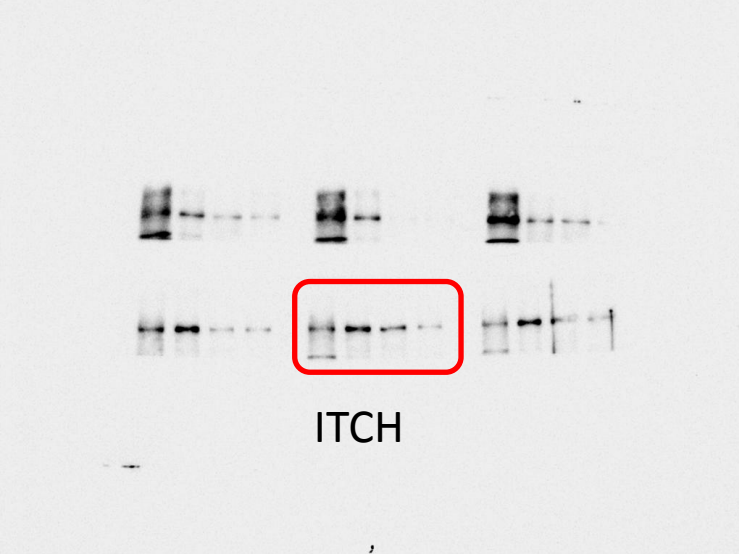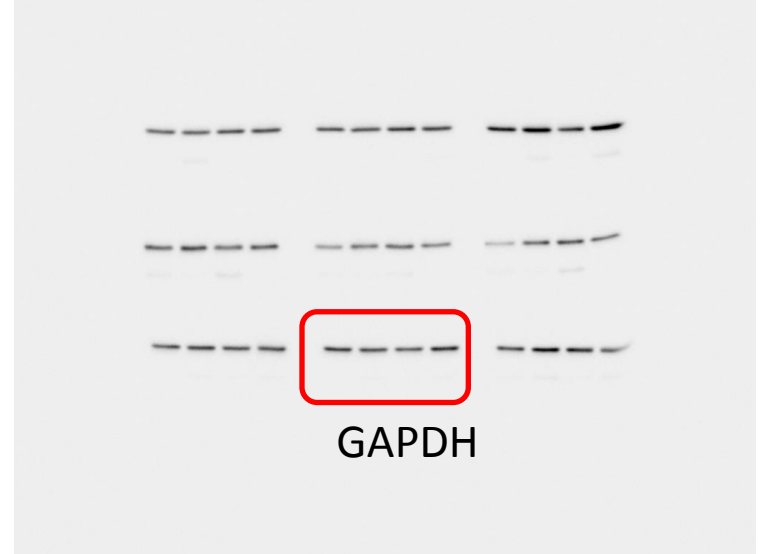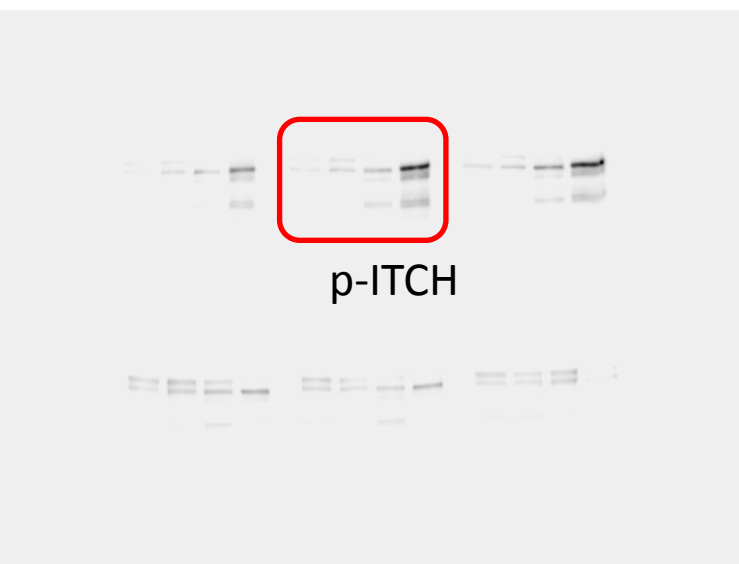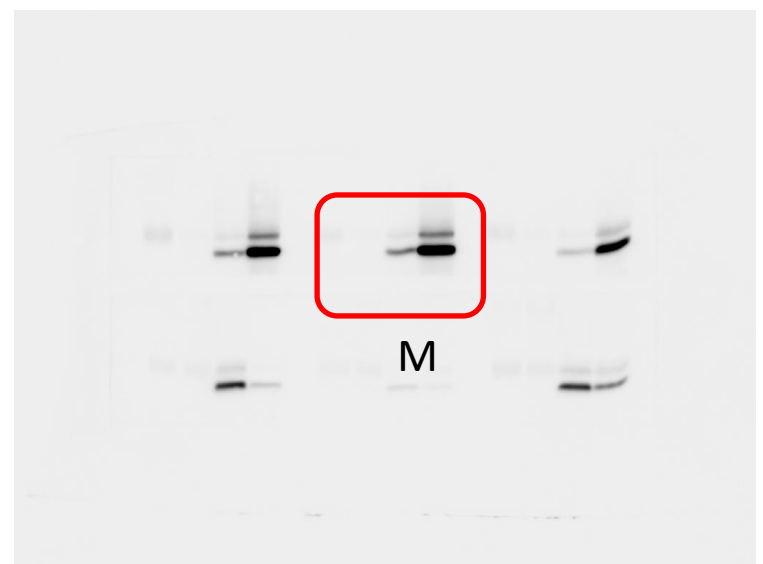

Supplement: Figure 6—source data 1. [file elife-105105-fig6-data1.zip › Figure 6F.pdf]

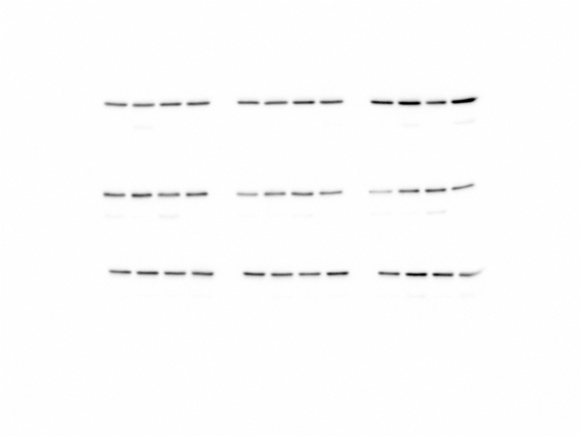

Supplement: Figure 6—source data 2. [file elife-105105-fig6-data2.zip › Figure 6C/GAPDH.tif]

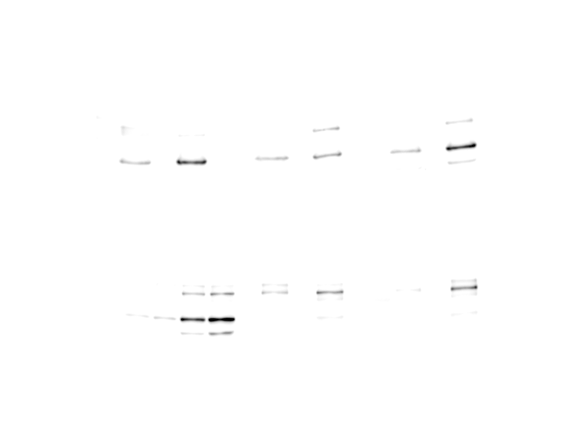

Supplement: Figure 6—source data 2. [file elife-105105-fig6-data2.zip › Figure 6C/p-ITCH 6C.tif]

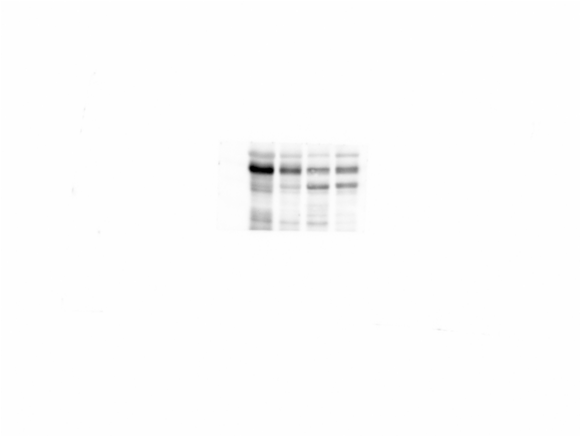

Supplement: Figure 6—source data 2. [file elife-105105-fig6-data2.zip › Figure 6C/pJNK.tif]

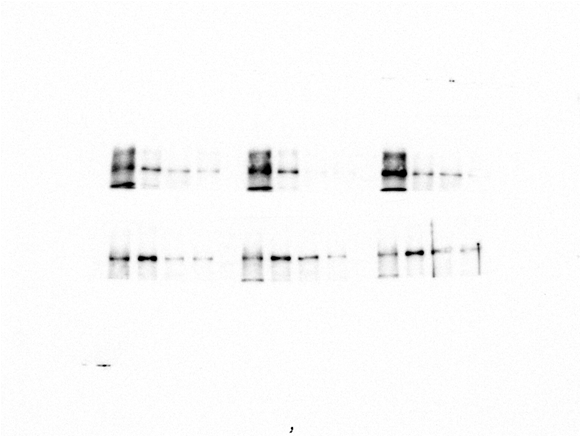

Supplement: Figure 6—source data 2. [file elife-105105-fig6-data2.zip › Figure 6F/ITCH.tif]

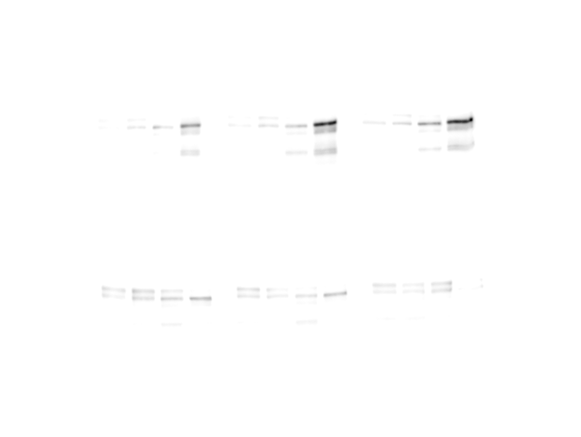

Supplement: Figure 6—source data 2. [file elife-105105-fig6-data2.zip › Figure 6F/p-ITCH 6F.tif]

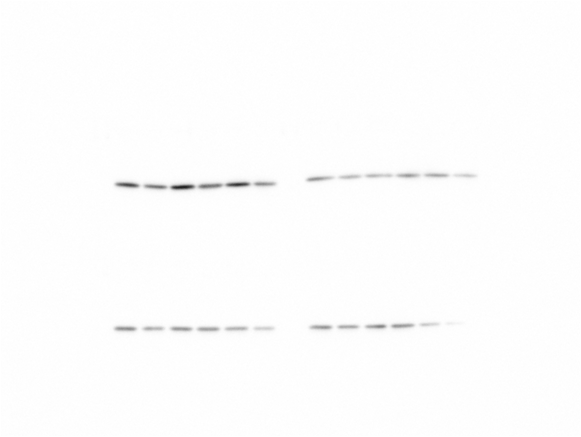

Supplement: Figure 6—source data 2. [file elife-105105-fig6-data2.zip › Figure 6L/gapdh.tif]

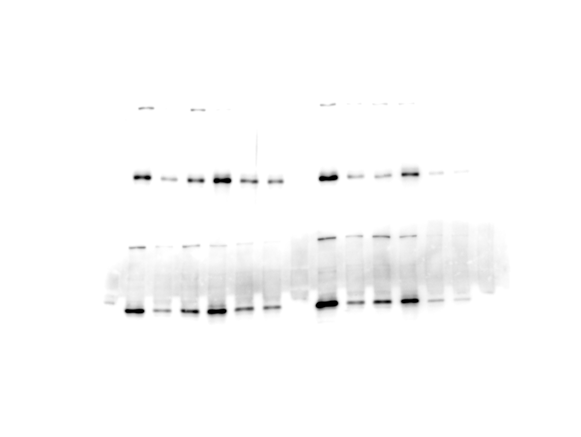

Supplement: Figure 6—source data 2. [file elife-105105-fig6-data2.zip › Figure 6L/itch.tif]

S7B

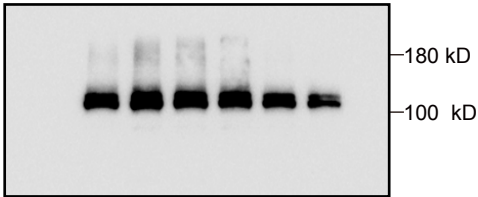

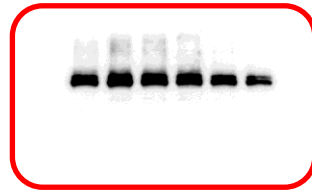

**ITCH**

Supplement: Figure 6—figure supplement 1—source data 1. [file elife-105105-fig6-figsupp1-data1.zip › Figure 6-figure supplement 1B.pdf]

S7A

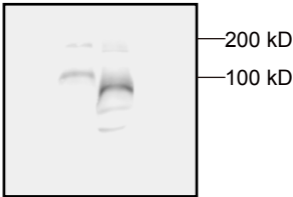

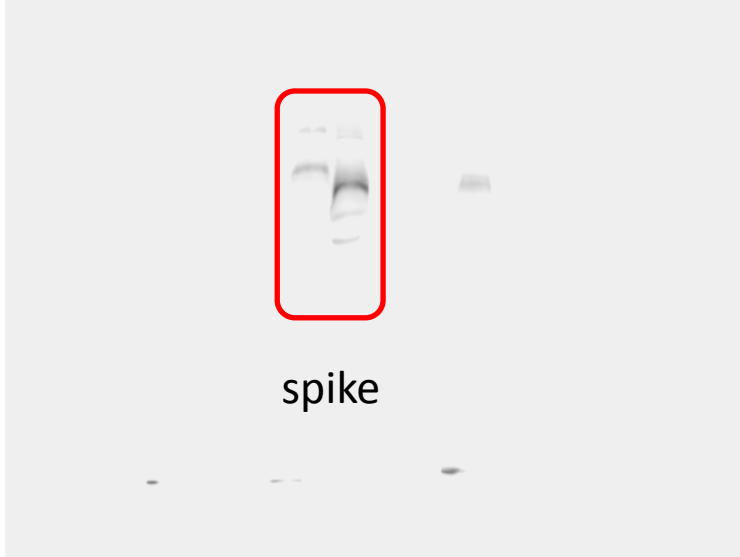

Supplement: Figure 6—figure supplement 1—source data 1. [file elife-105105-fig6-figsupp1-data1.zip › Figure 6-figure supplement 1A.pdf]

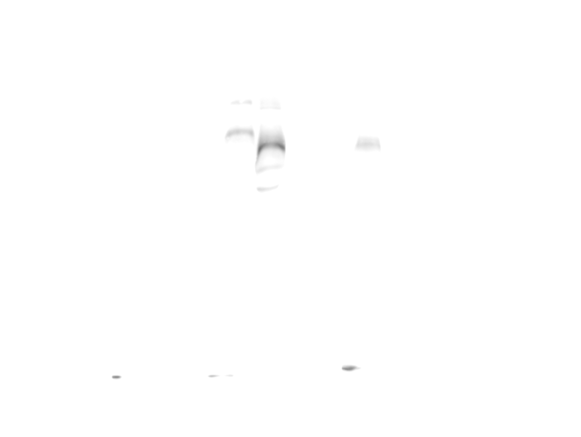

Supplement: Figure 6—figure supplement 1—source data 2. [file elife-105105-fig6-figsupp1-data2.zip › Figure 6-figure supplement 1A/S7A spike.tif]
