# Supplementary figures and images for: Ubiquitin ligase ITCH regulates life cycle of SARS-CoV-2 virus (part 4 of 4)
Source: eLife. 2026 May 29;14:RP105105. doi: 10.7554/eLife.105105 (PMC13221179; doi:10.7554/eLife.105105)

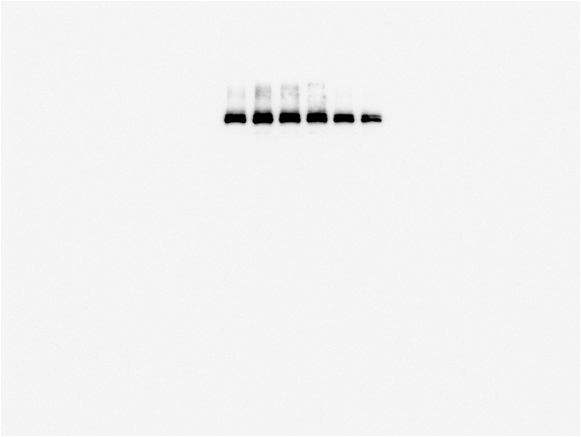

Supplement: Figure 6—figure supplement 1—source data 2. [file elife-105105-fig6-figsupp1-data2.zip › Figure 6-figure supplement 1B/itch.tif]
